# Supplementary material for: WIPI2b recruitment to phagophores and ATG16L1 binding are regulated by ULK1 phosphorylation
Source: EMBO Rep. 2024 Aug 16;25(9):8. doi: 10.1038/s44319-024-00215-5 (PMC11387628; doi:10.1038/s44319-024-00215-5)
Supplement: Supplementary file 3 — Source data Fig. 1 [file 44319_2024_215_MOESM3_ESM.zip › Figure 1/1D/F1D.pdf]

Figure 1D

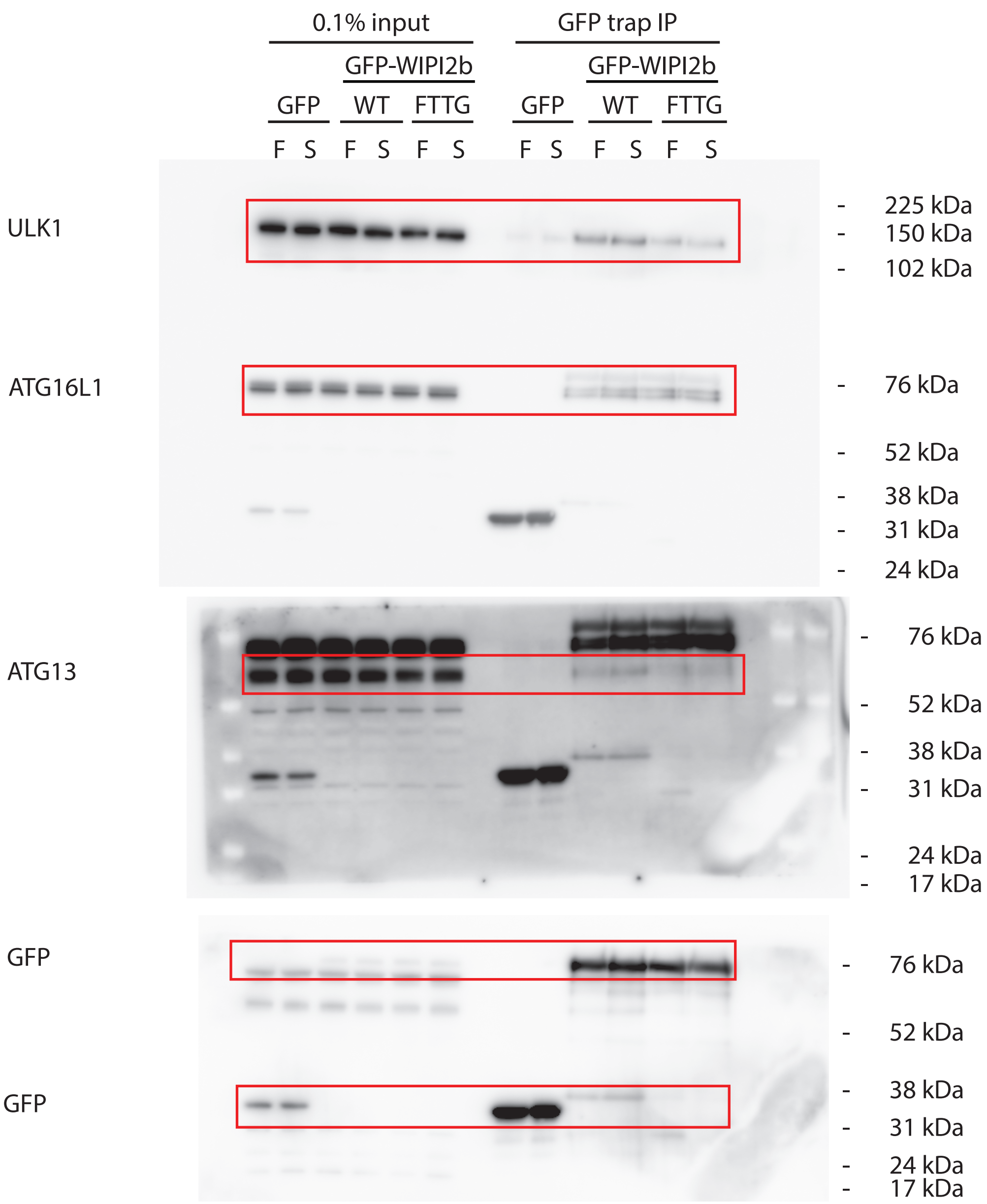

the inputs of all three replicates were re-run and blotted for LC3B

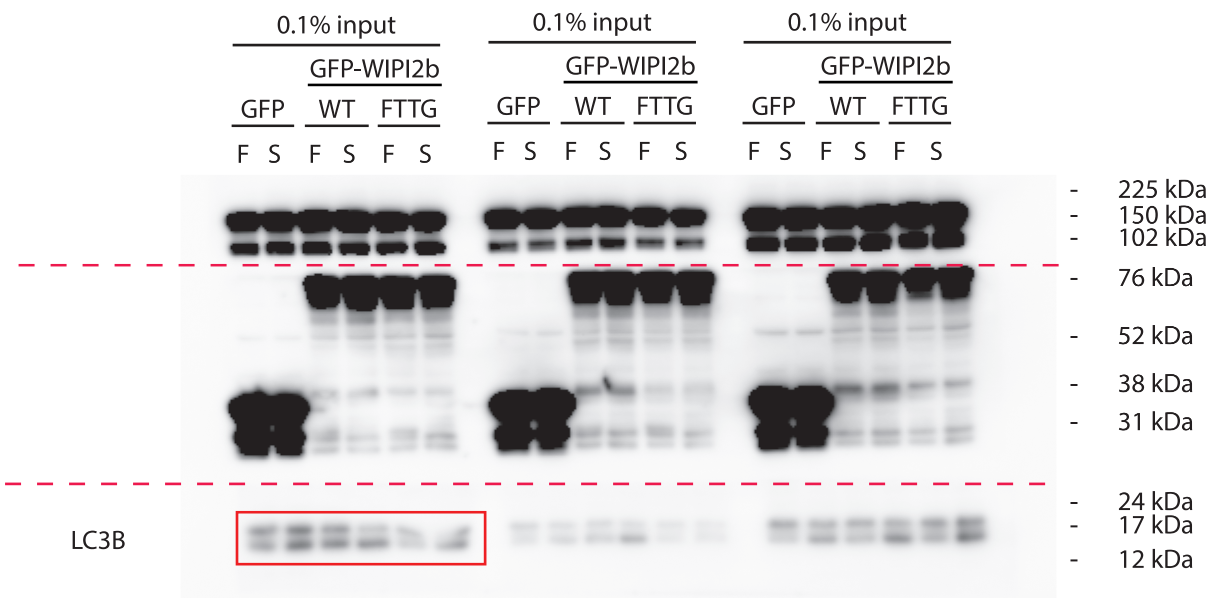

dashed red line shows where the membrane was cut
