# Supplementary figures and images for: WIPI2b recruitment to phagophores and ATG16L1 binding are regulated by ULK1 phosphorylation (part 1 of 2)
Source: EMBO Rep. 2024 Aug 16;25(9):8. doi: 10.1038/s44319-024-00215-5 (PMC11387628; doi:10.1038/s44319-024-00215-5)

Figure 1F

dashed red line shows where the membrane was cut

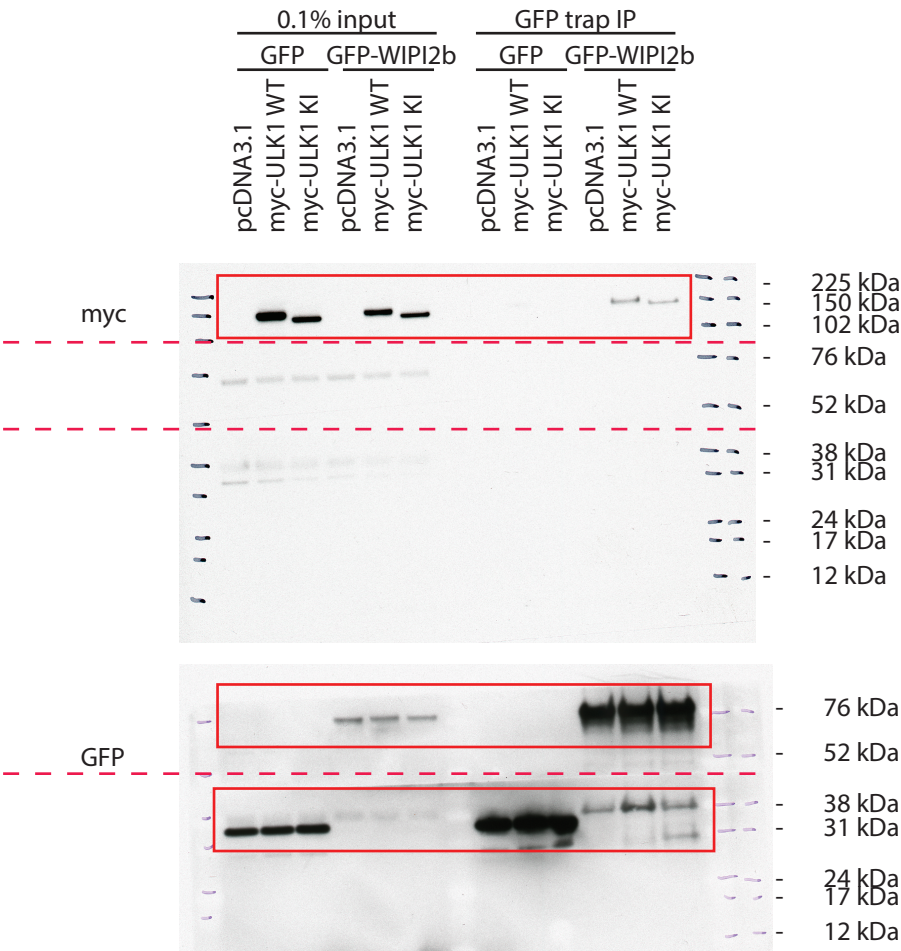

Supplement: Supplementary file 3 — Source data Fig. 1 [file 44319_2024_215_MOESM3_ESM.zip › Figure 1/1F/F1F.pdf]

Figure 1A

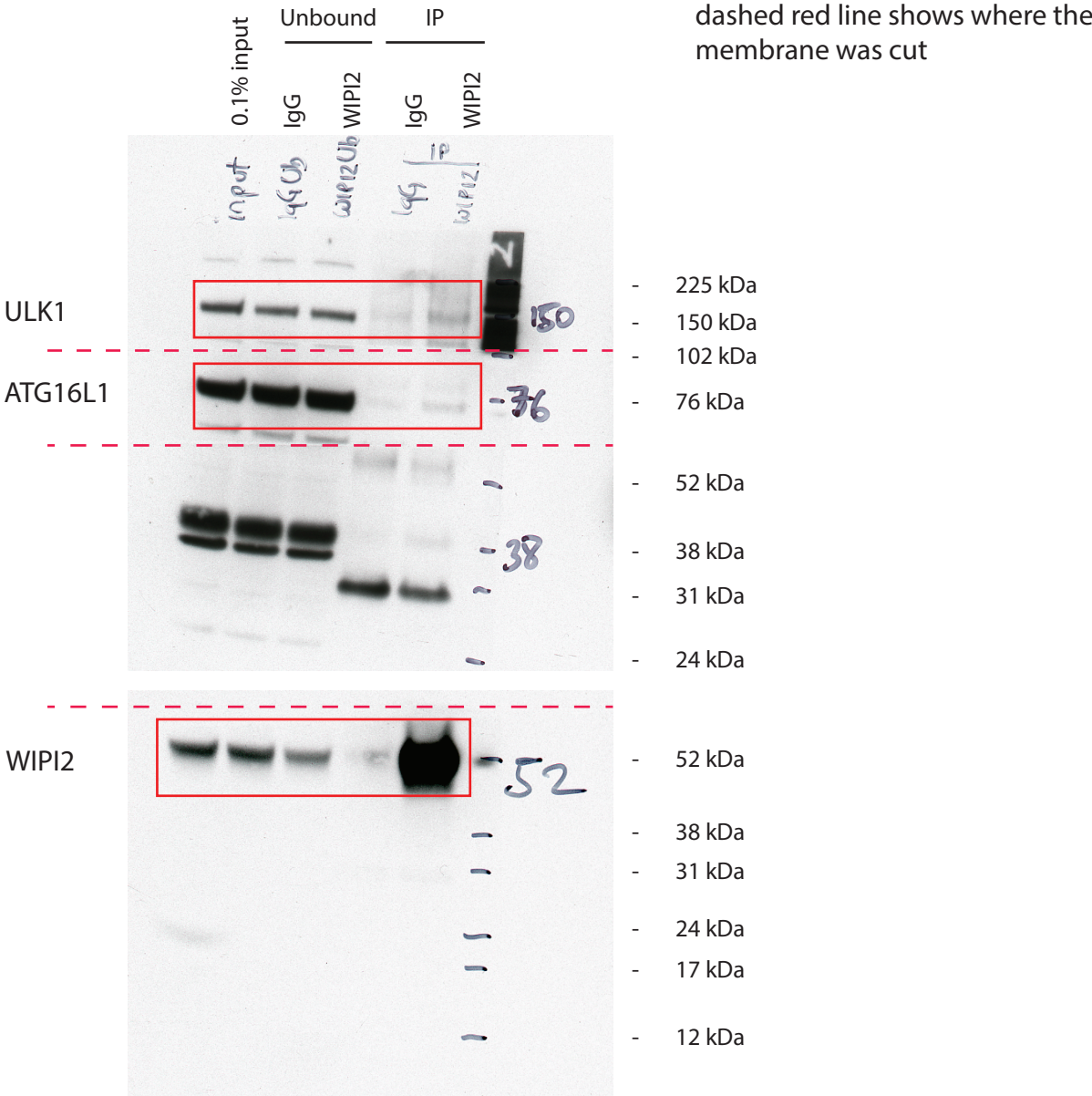

Supplement: Supplementary file 3 — Source data Fig. 1 [file 44319_2024_215_MOESM3_ESM.zip › Figure 1/1A/F1A.pdf]

Figure 1B

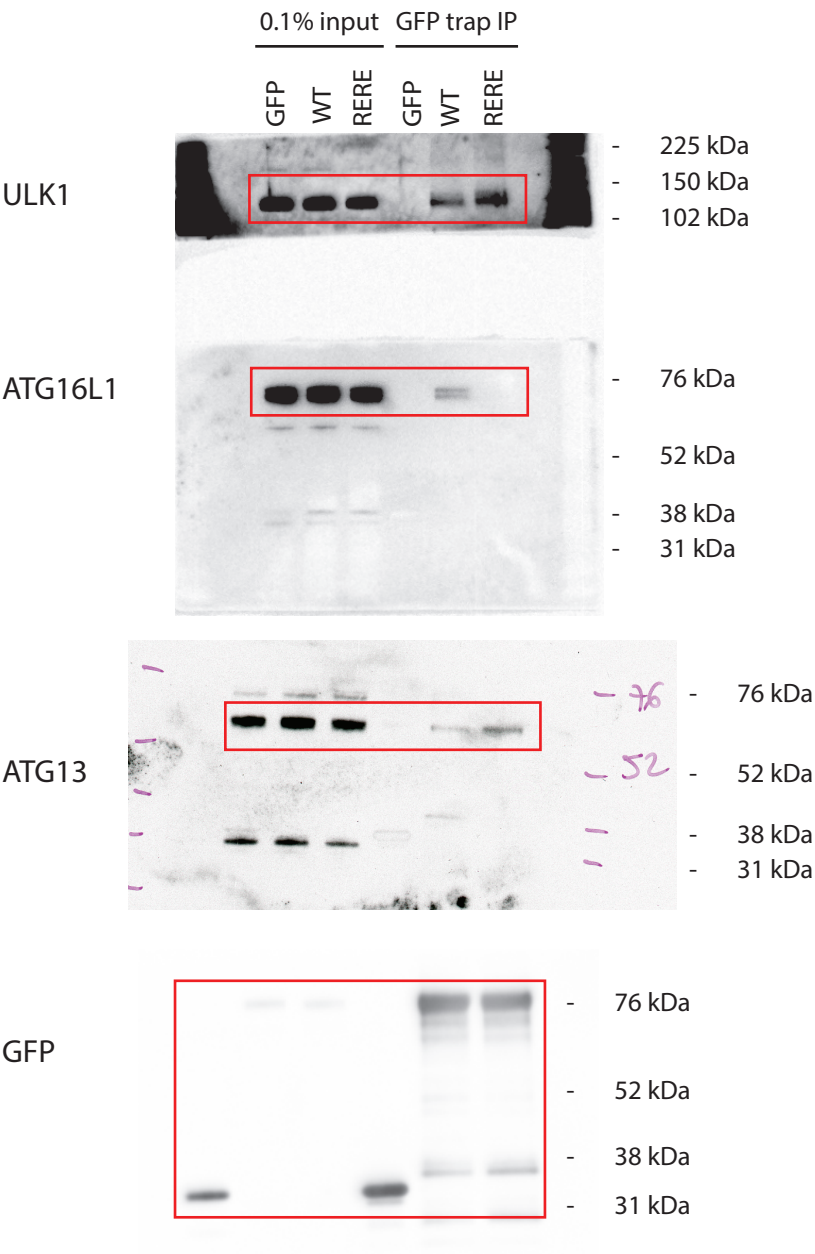

Supplement: Supplementary file 3 — Source data Fig. 1 [file 44319_2024_215_MOESM3_ESM.zip › Figure 1/1B/F1B.pdf]

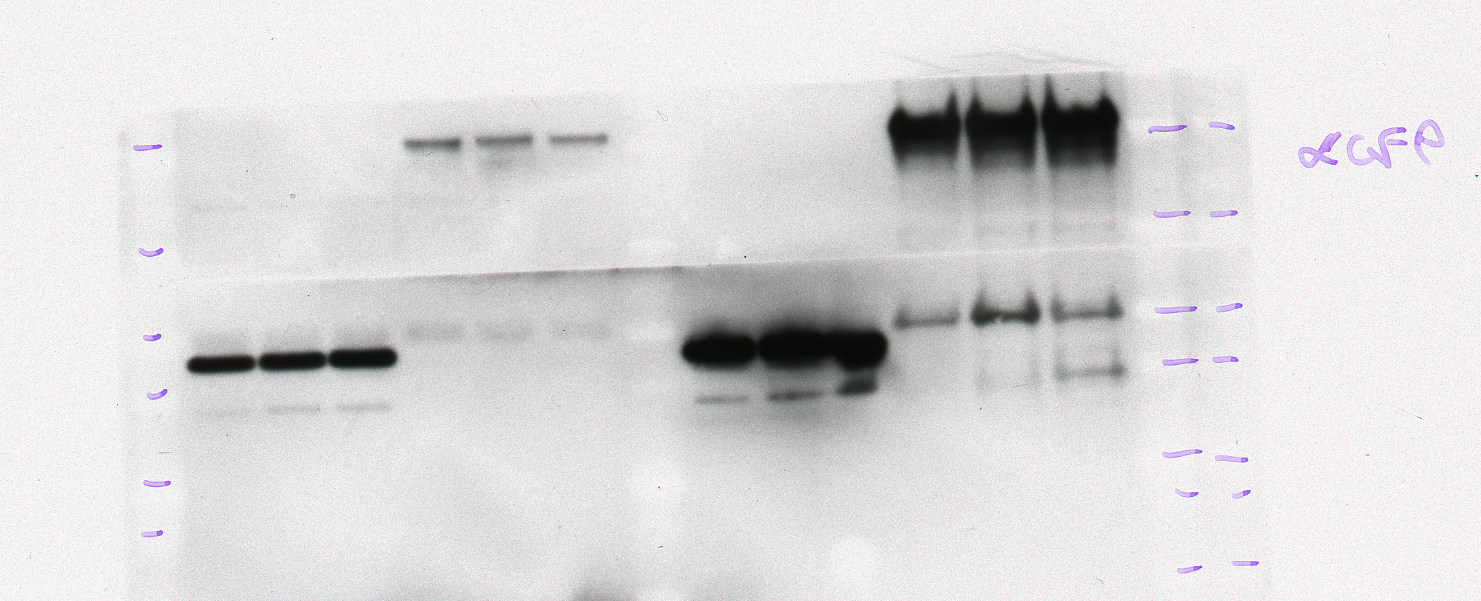

Supplement: Supplementary file 3 — Source data Fig. 1 [file 44319_2024_215_MOESM3_ESM.zip › Figure 1/1F/Images/western GFP.tif]

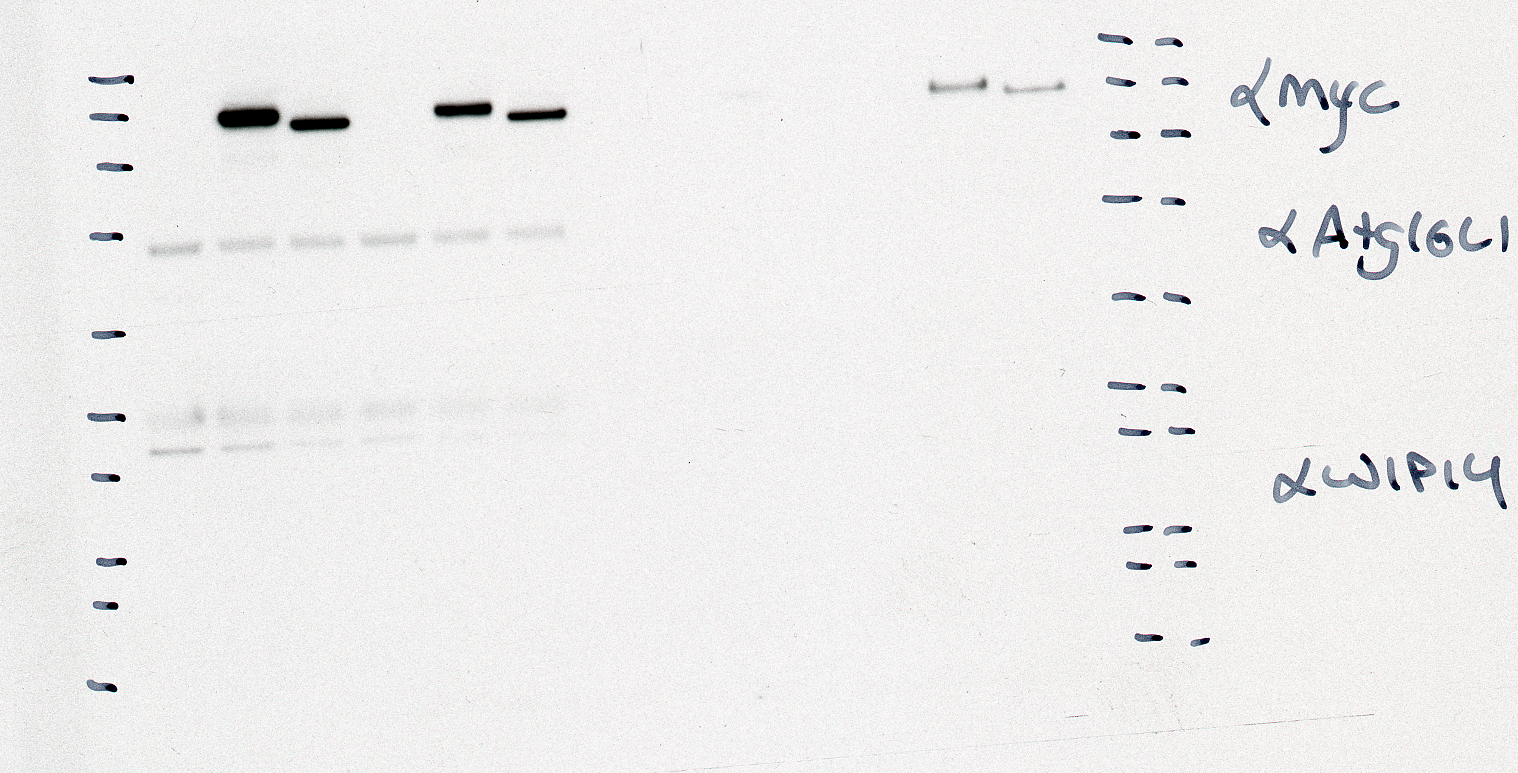

Supplement: Supplementary file 3 — Source data Fig. 1 [file 44319_2024_215_MOESM3_ESM.zip › Figure 1/1F/Images/western myc.tif]

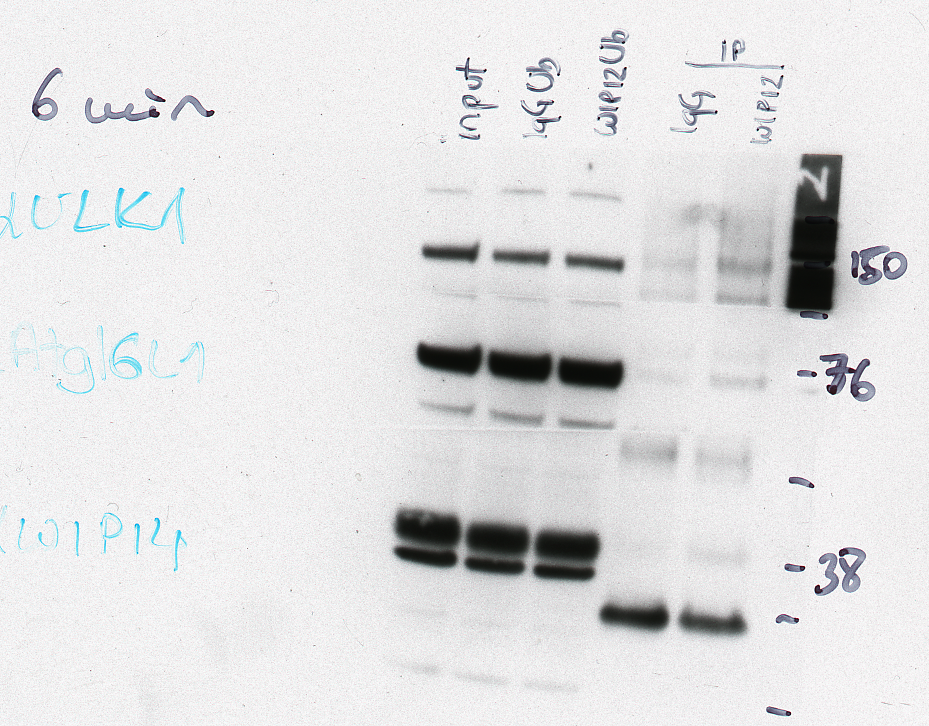

Supplement: Supplementary file 3 — Source data Fig. 1 [file 44319_2024_215_MOESM3_ESM.zip › Figure 1/1A/Images/western ULK1 ATG16L1.tif]

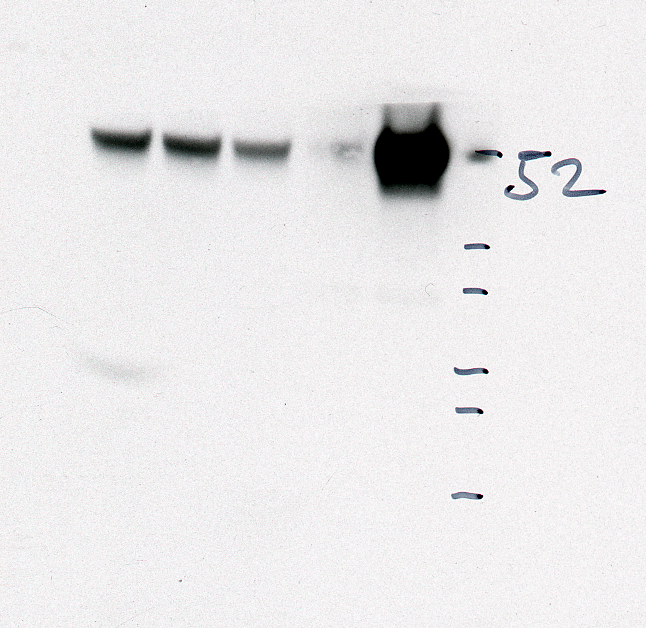

Supplement: Supplementary file 3 — Source data Fig. 1 [file 44319_2024_215_MOESM3_ESM.zip › Figure 1/1A/Images/western WIPI2.tif]

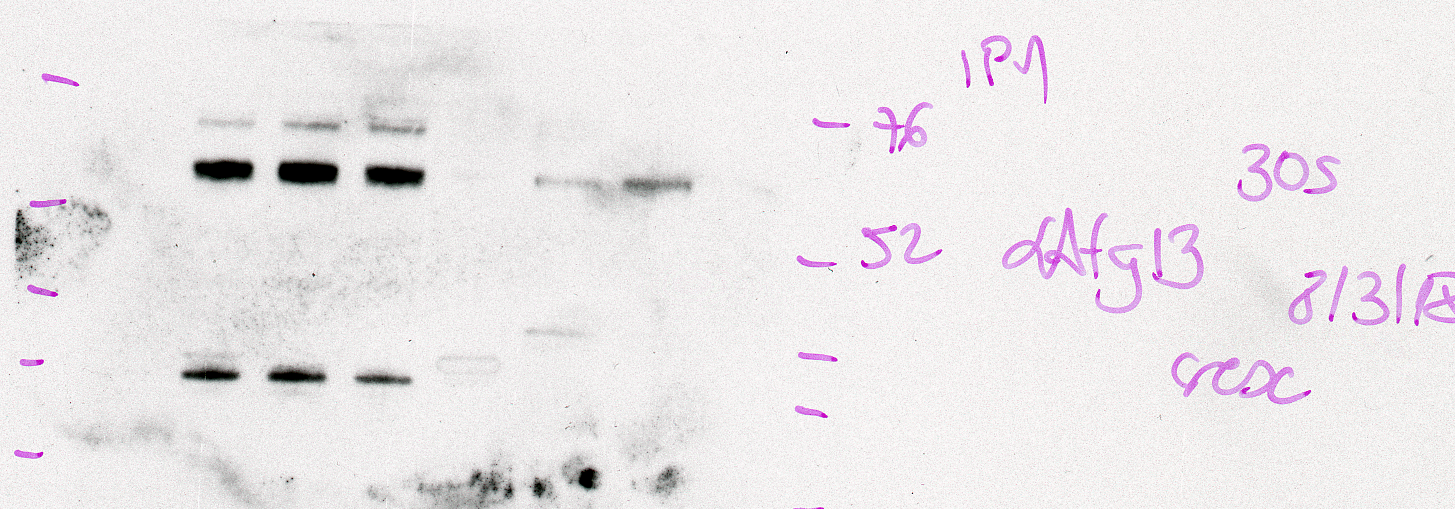

Supplement: Supplementary file 3 — Source data Fig. 1 [file 44319_2024_215_MOESM3_ESM.zip › Figure 1/1B/Images/western ATG13.tif]

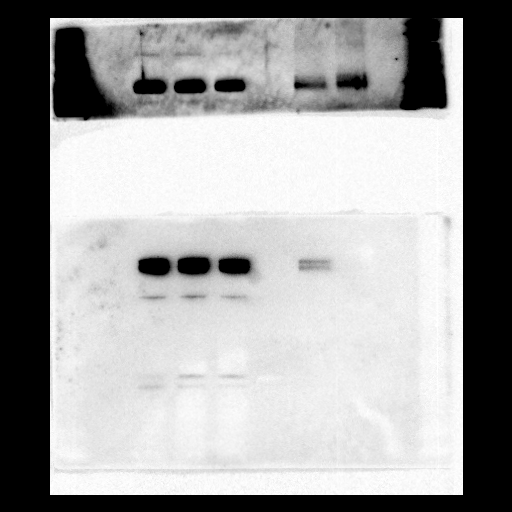

Supplement: Supplementary file 3 — Source data Fig. 1 [file 44319_2024_215_MOESM3_ESM.zip › Figure 1/1B/Images/western ULK1_ATG16L1.tif]

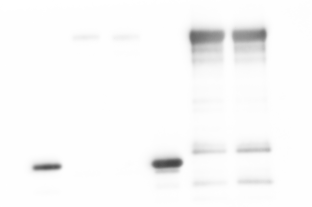

Supplement: Supplementary file 3 — Source data Fig. 1 [file 44319_2024_215_MOESM3_ESM.zip › Figure 1/1B/Images/western GFP.tiff]

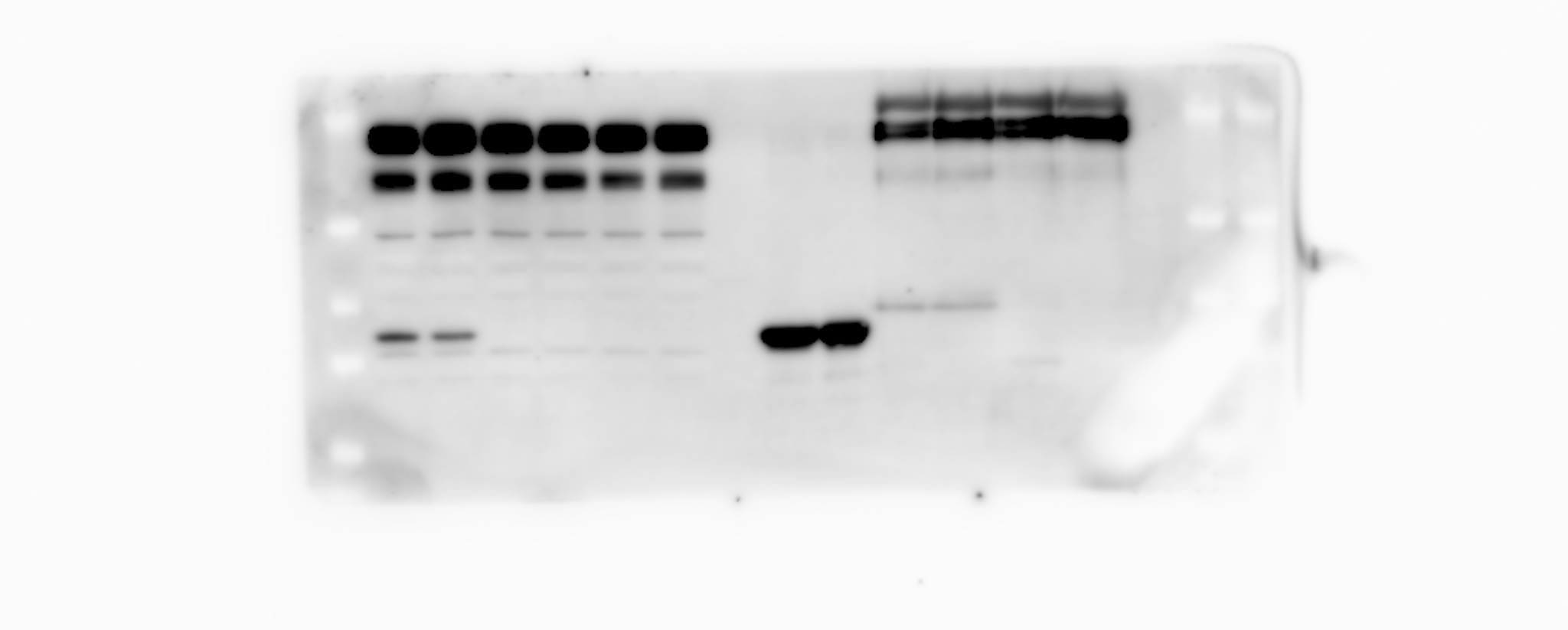

Supplement: Supplementary file 3 — Source data Fig. 1 [file 44319_2024_215_MOESM3_ESM.zip › Figure 1/1D/Images/western ATG13.tif]

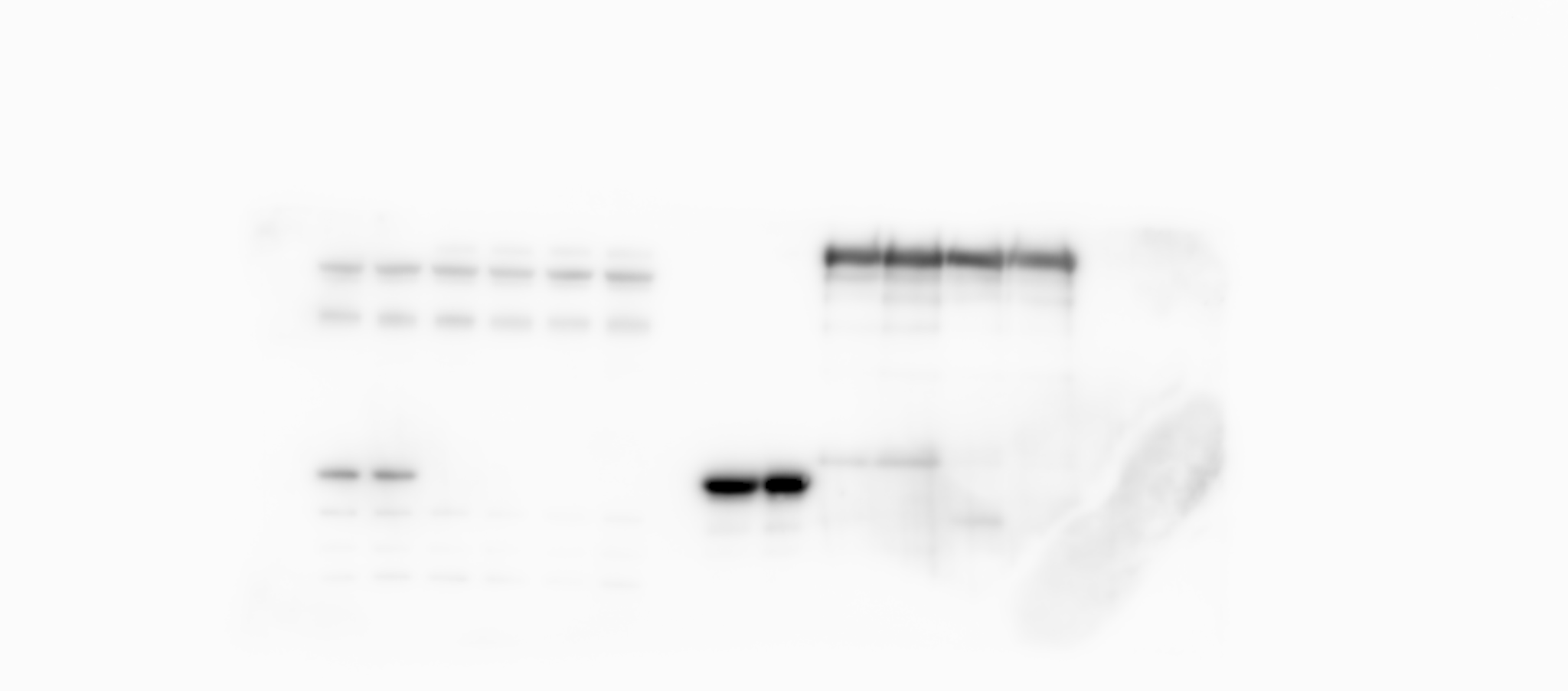

Supplement: Supplementary file 3 — Source data Fig. 1 [file 44319_2024_215_MOESM3_ESM.zip › Figure 1/1D/Images/western GFP.tif]

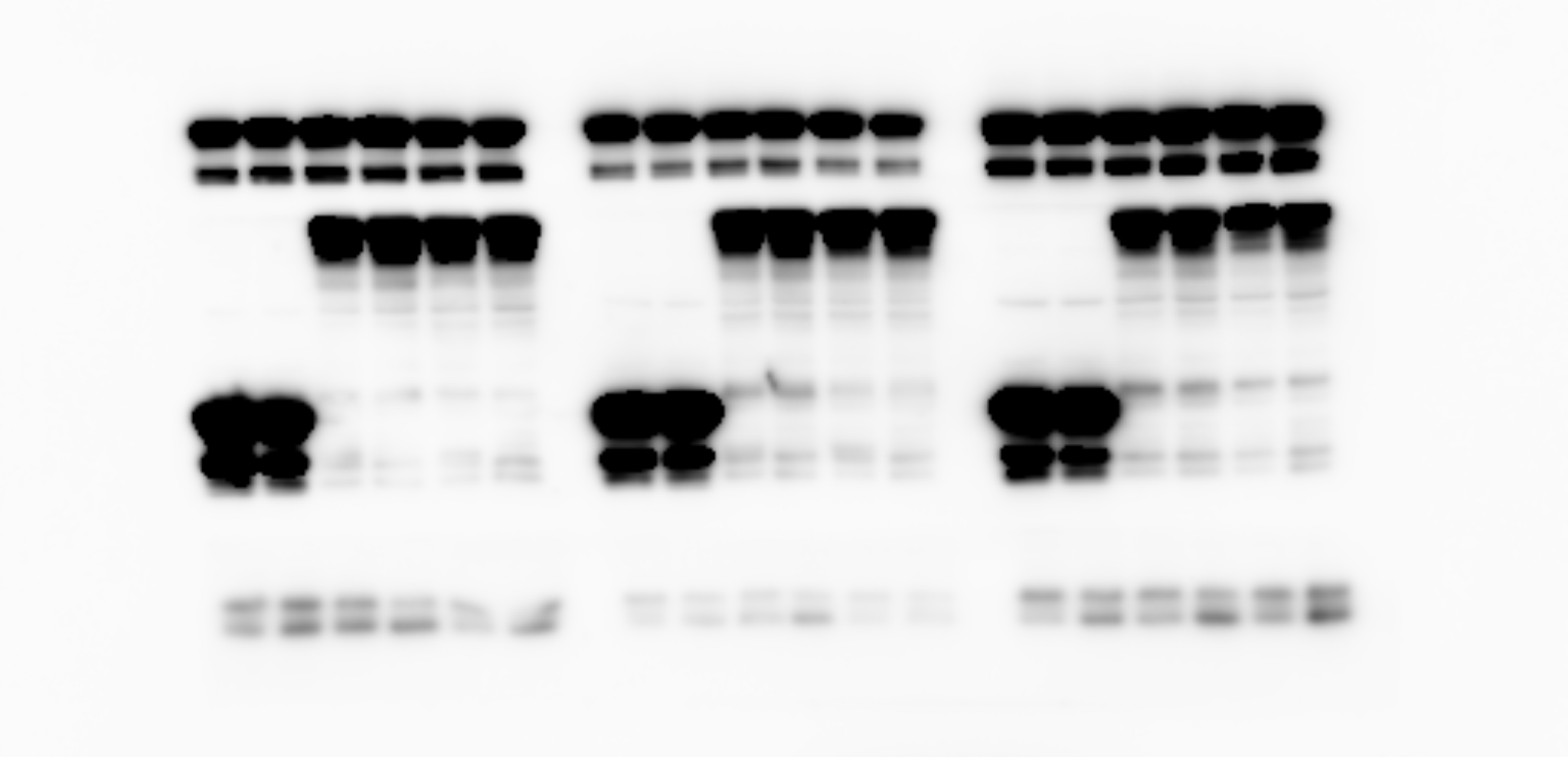

Supplement: Supplementary file 3 — Source data Fig. 1 [file 44319_2024_215_MOESM3_ESM.zip › Figure 1/1D/Images/western LC3.tif]

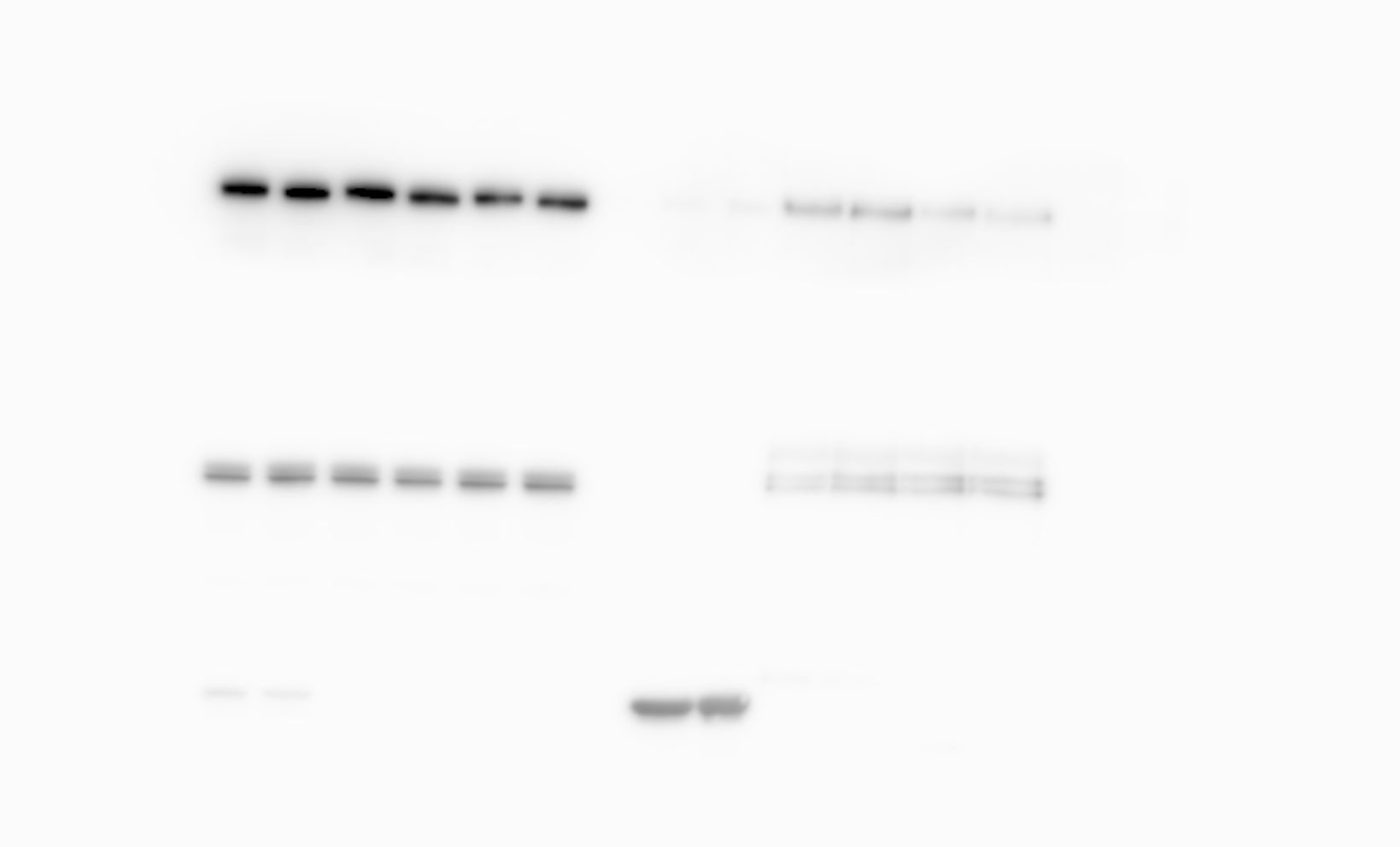

Supplement: Supplementary file 3 — Source data Fig. 1 [file 44319_2024_215_MOESM3_ESM.zip › Figure 1/1D/Images/western ULK1.tif]

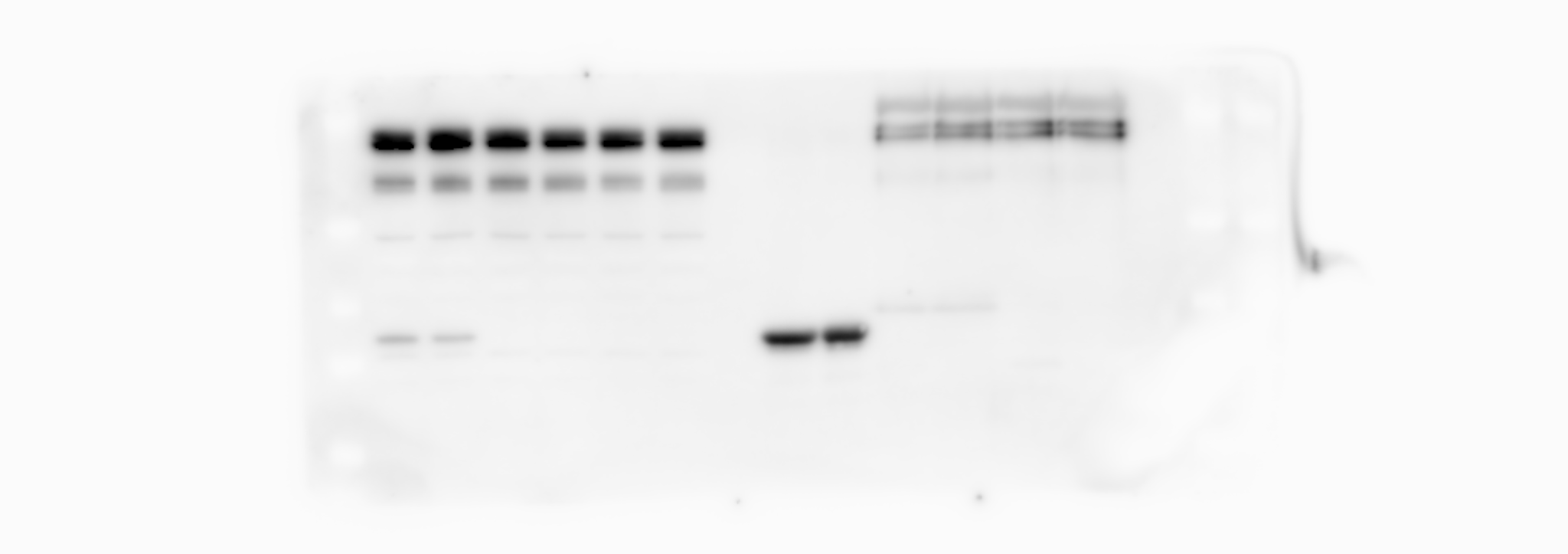

Supplement: Supplementary file 3 — Source data Fig. 1 [file 44319_2024_215_MOESM3_ESM.zip › Figure 1/1D/Images/western ATG16L1.tif]

Figure 2G

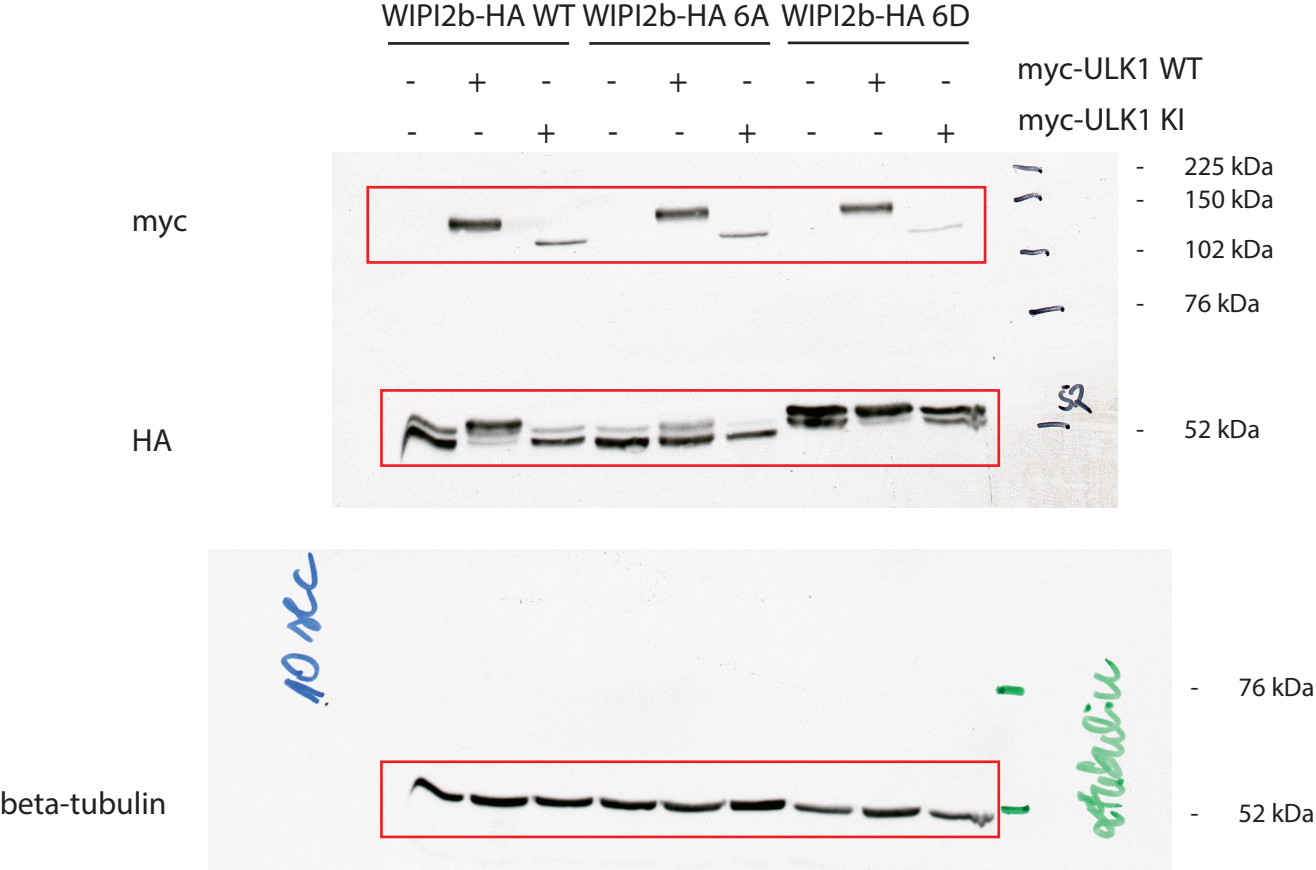

Supplement: Supplementary file 4 — Source data Fig. 2 [file 44319_2024_215_MOESM4_ESM.zip › Figure 2/2G/F2G.pdf]

Figure 2F

|   |   |   |   |   |   |   |   |              |
|---|---|---|---|---|---|---|---|--------------|
| + | + | - | - | + | - | - | - | WIPI2b-HA WT |
| - | - | + | + | - | + | - | - | WIPI2b-HA 6A |
| + | - | + | - | - | - | + | - | myc-ULK1 KI  |
| - | + | - | + | - | - | - | + | myc-ULK1 WT  |

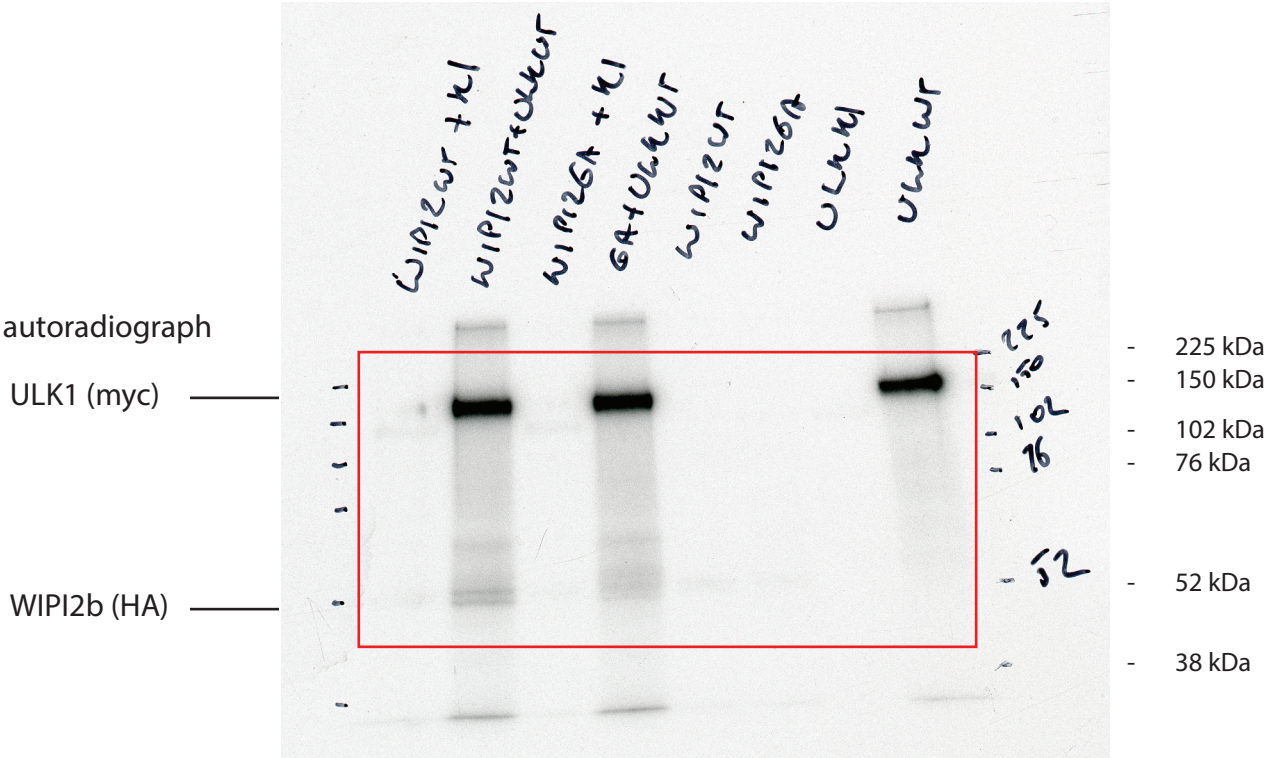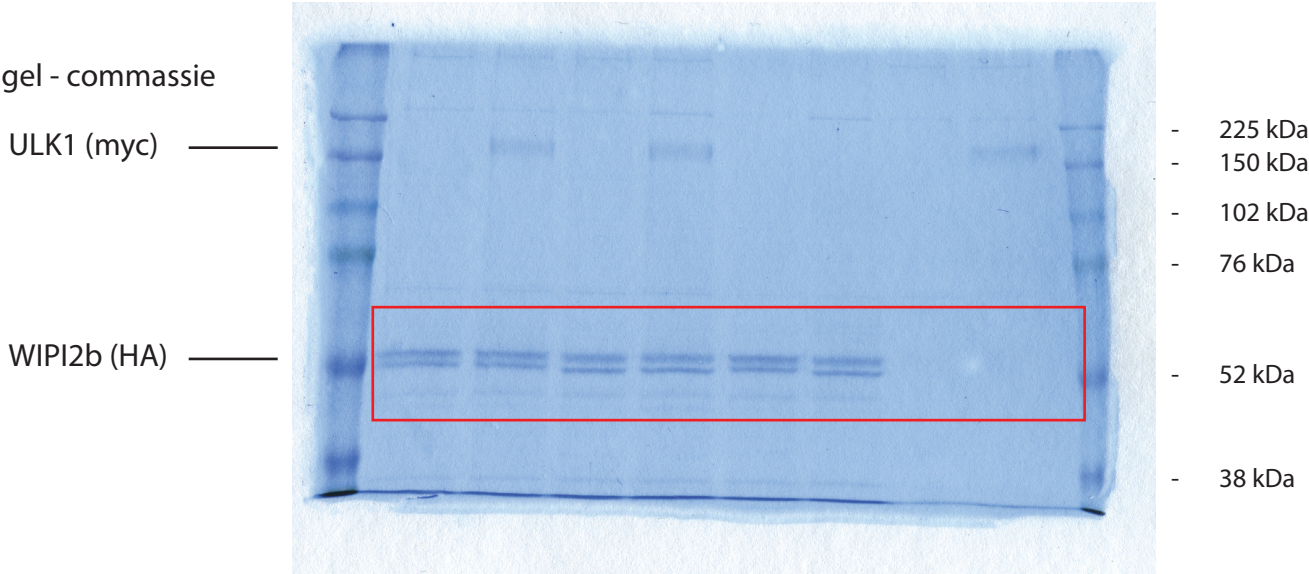

Supplement: Supplementary file 4 — Source data Fig. 2 [file 44319_2024_215_MOESM4_ESM.zip › Figure 2/2H/F2H.pdf]

Figure 2A

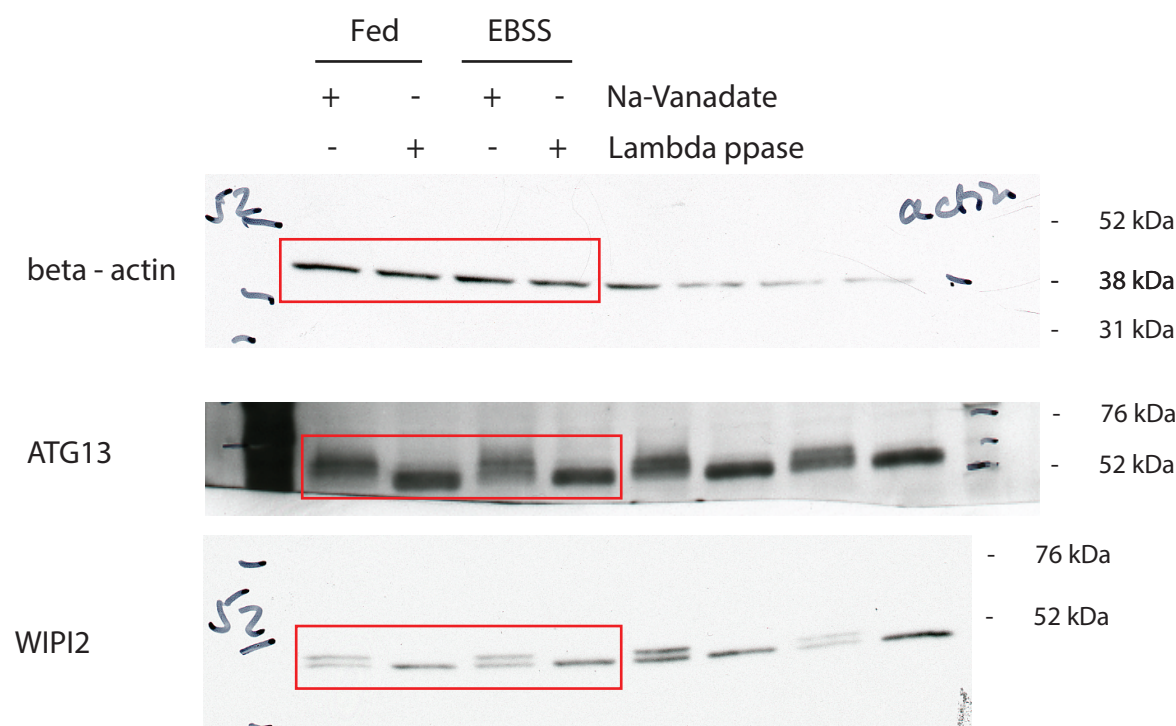

Supplement: Supplementary file 4 — Source data Fig. 2 [file 44319_2024_215_MOESM4_ESM.zip › Figure 2/2A/F2A.pdf]

Figure 2D

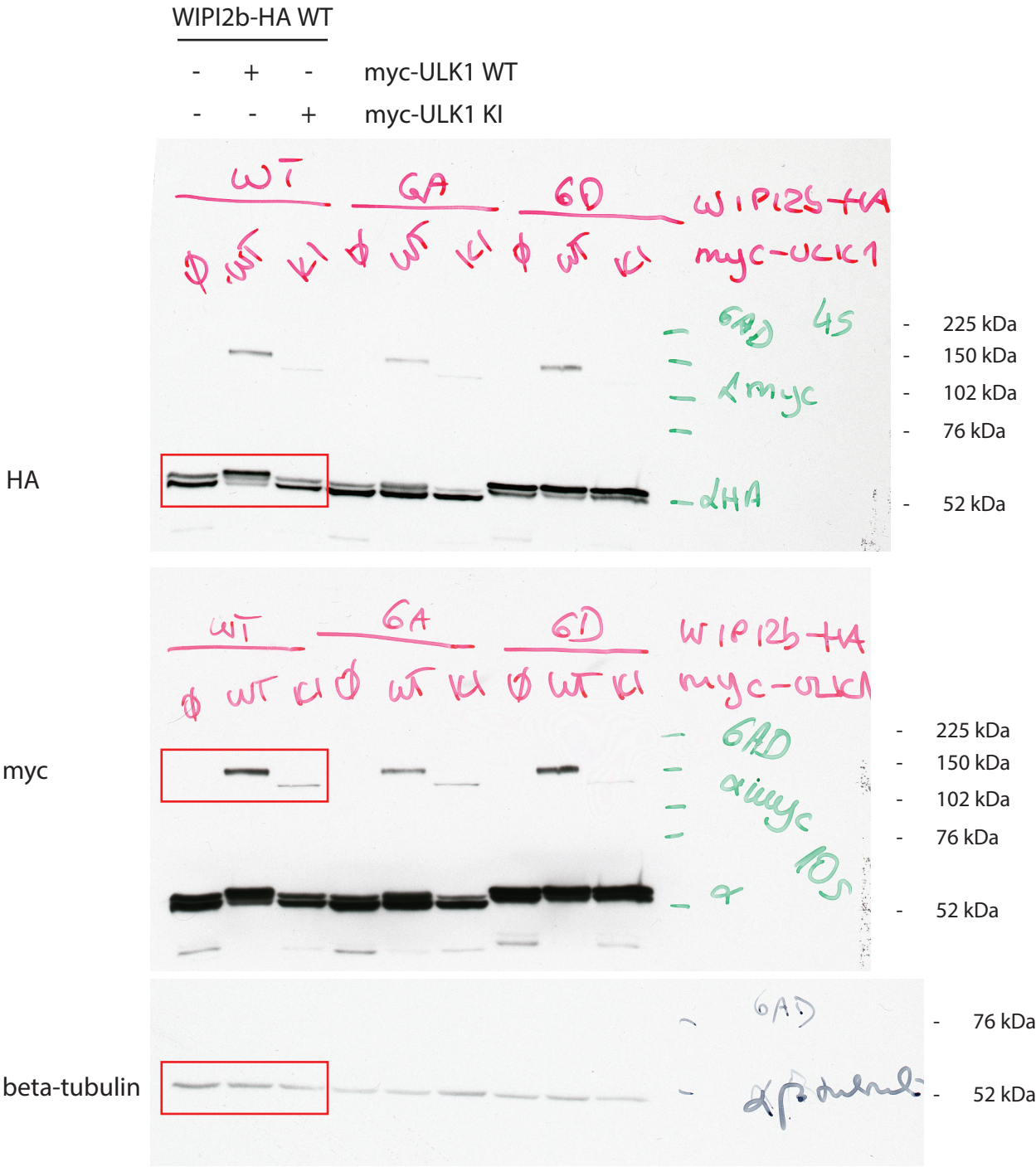

Supplement: Supplementary file 4 — Source data Fig. 2 [file 44319_2024_215_MOESM4_ESM.zip › Figure 2/2D/F2D.pdf]

Figure 2E

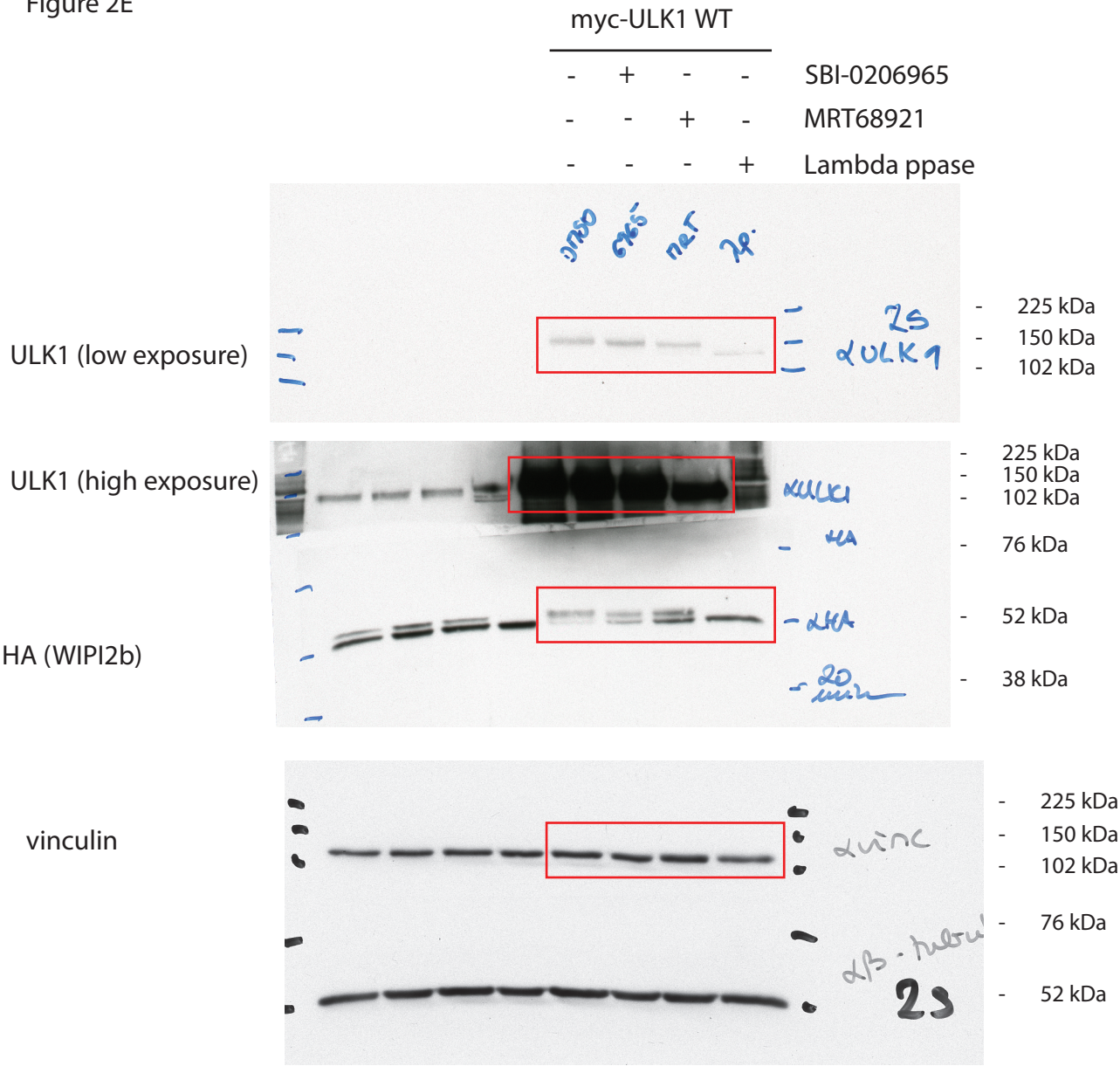

Supplement: Supplementary file 4 — Source data Fig. 2 [file 44319_2024_215_MOESM4_ESM.zip › Figure 2/2E/F2E.pdf]

Figure 2B

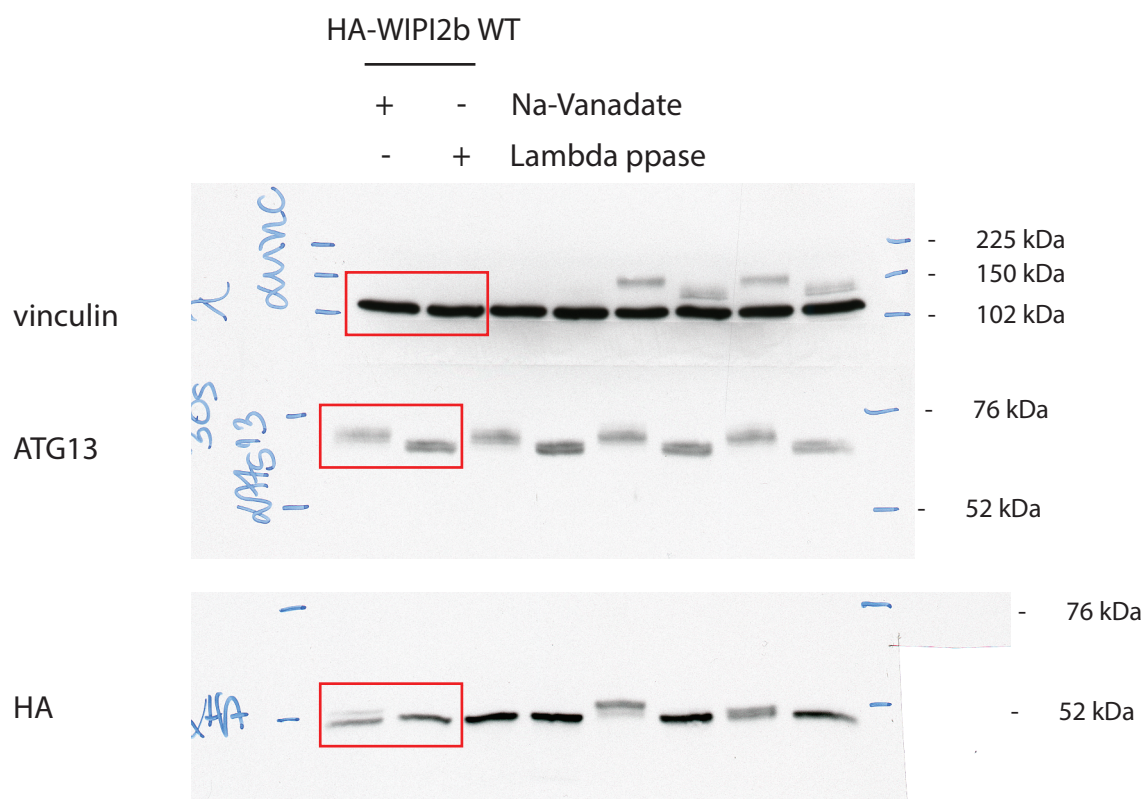

Supplement: Supplementary file 4 — Source data Fig. 2 [file 44319_2024_215_MOESM4_ESM.zip › Figure 2/2B/F2B.pdf]

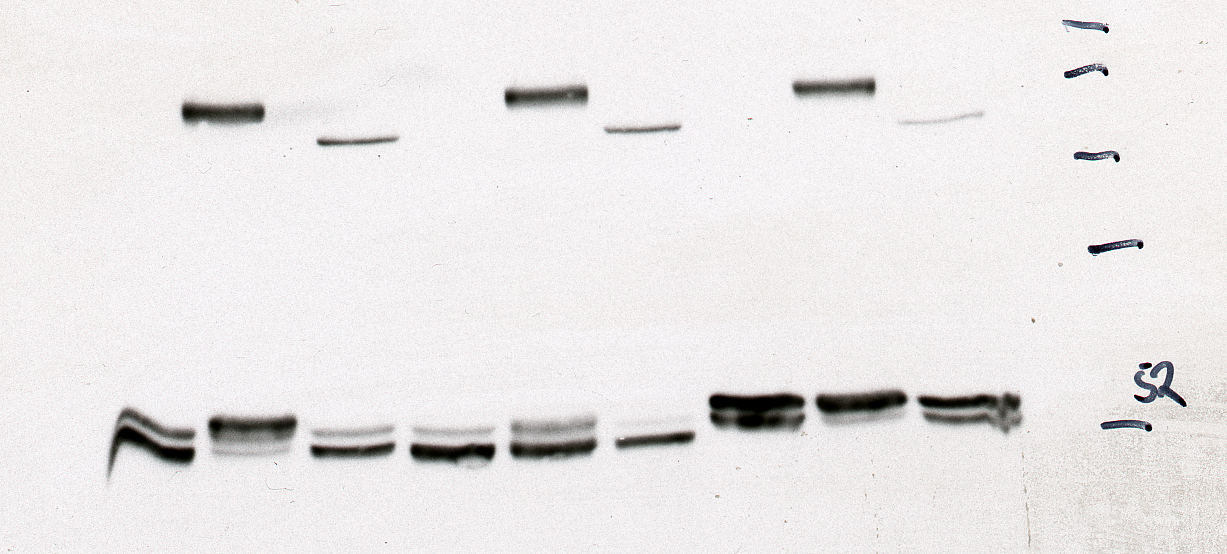

Supplement: Supplementary file 4 — Source data Fig. 2 [file 44319_2024_215_MOESM4_ESM.zip › Figure 2/2G/Images/western myc_HA.tif]

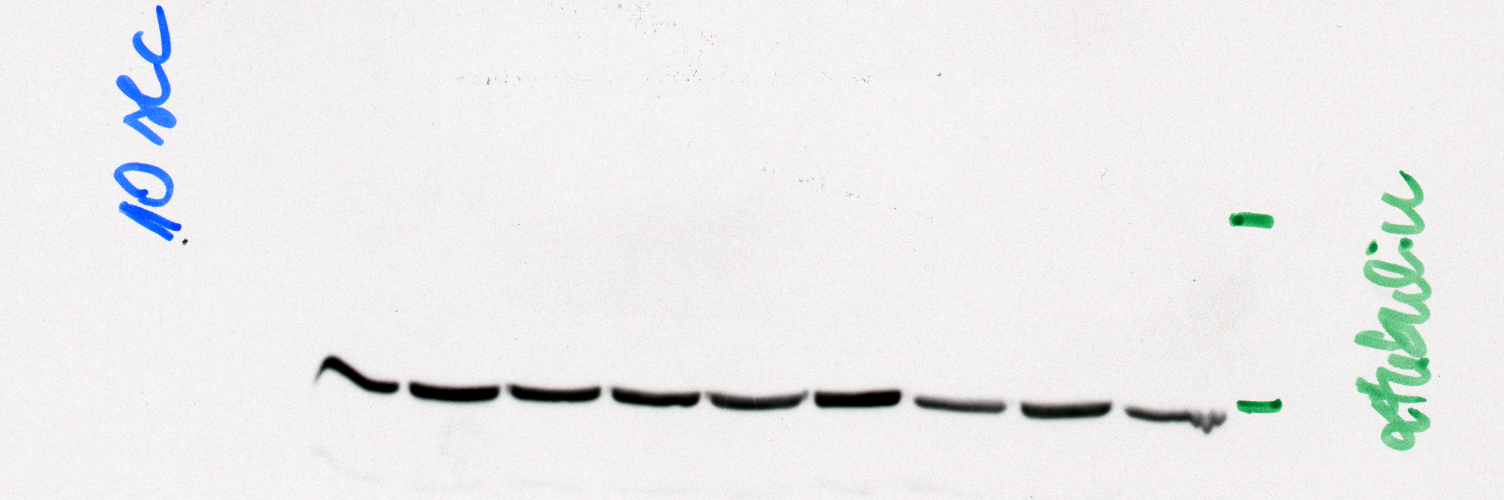

Supplement: Supplementary file 4 — Source data Fig. 2 [file 44319_2024_215_MOESM4_ESM.zip › Figure 2/2G/Images/western beta tubulin.tif]

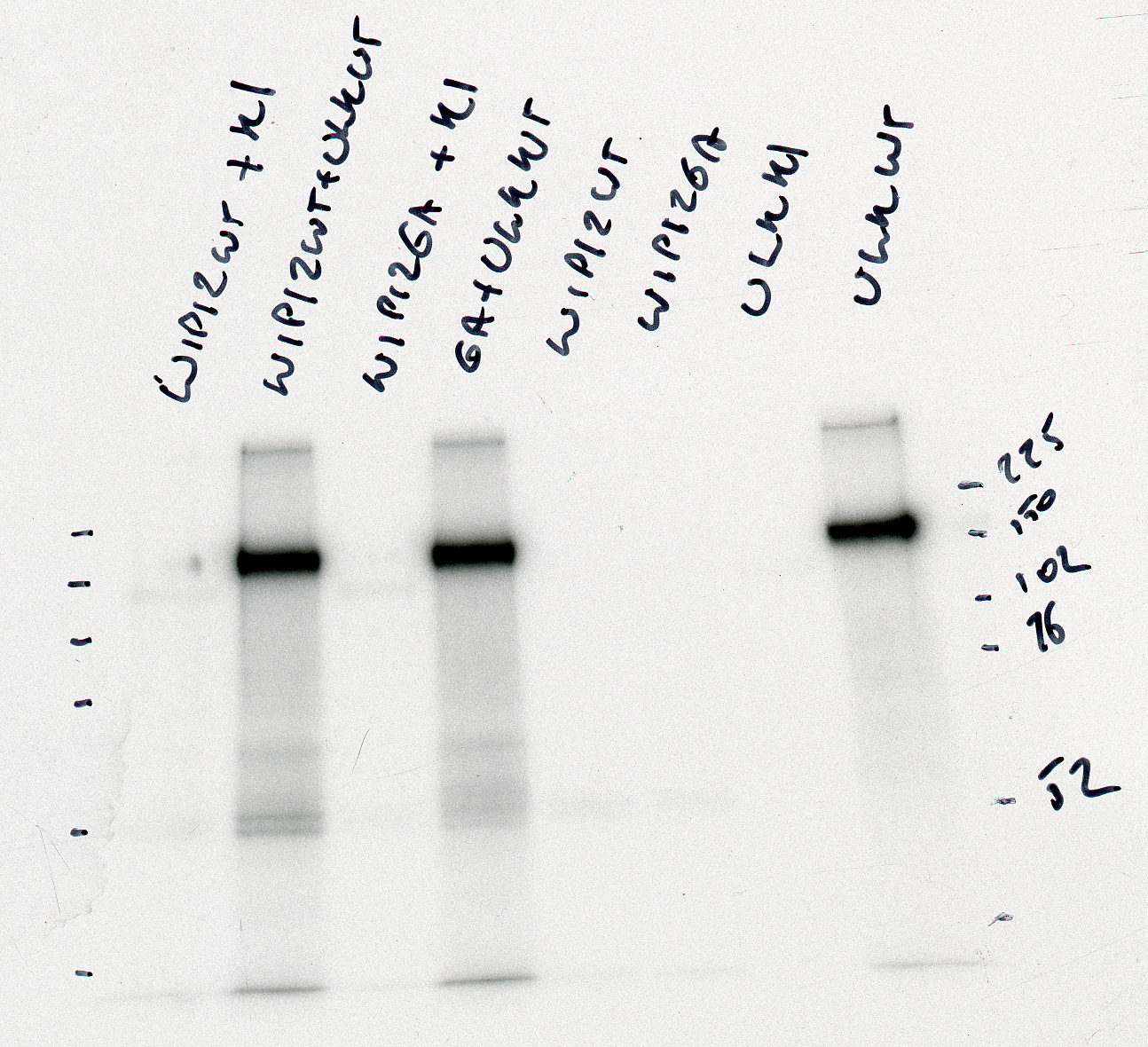

Supplement: Supplementary file 4 — Source data Fig. 2 [file 44319_2024_215_MOESM4_ESM.zip › Figure 2/2H/Images/autoradiograph.tif]

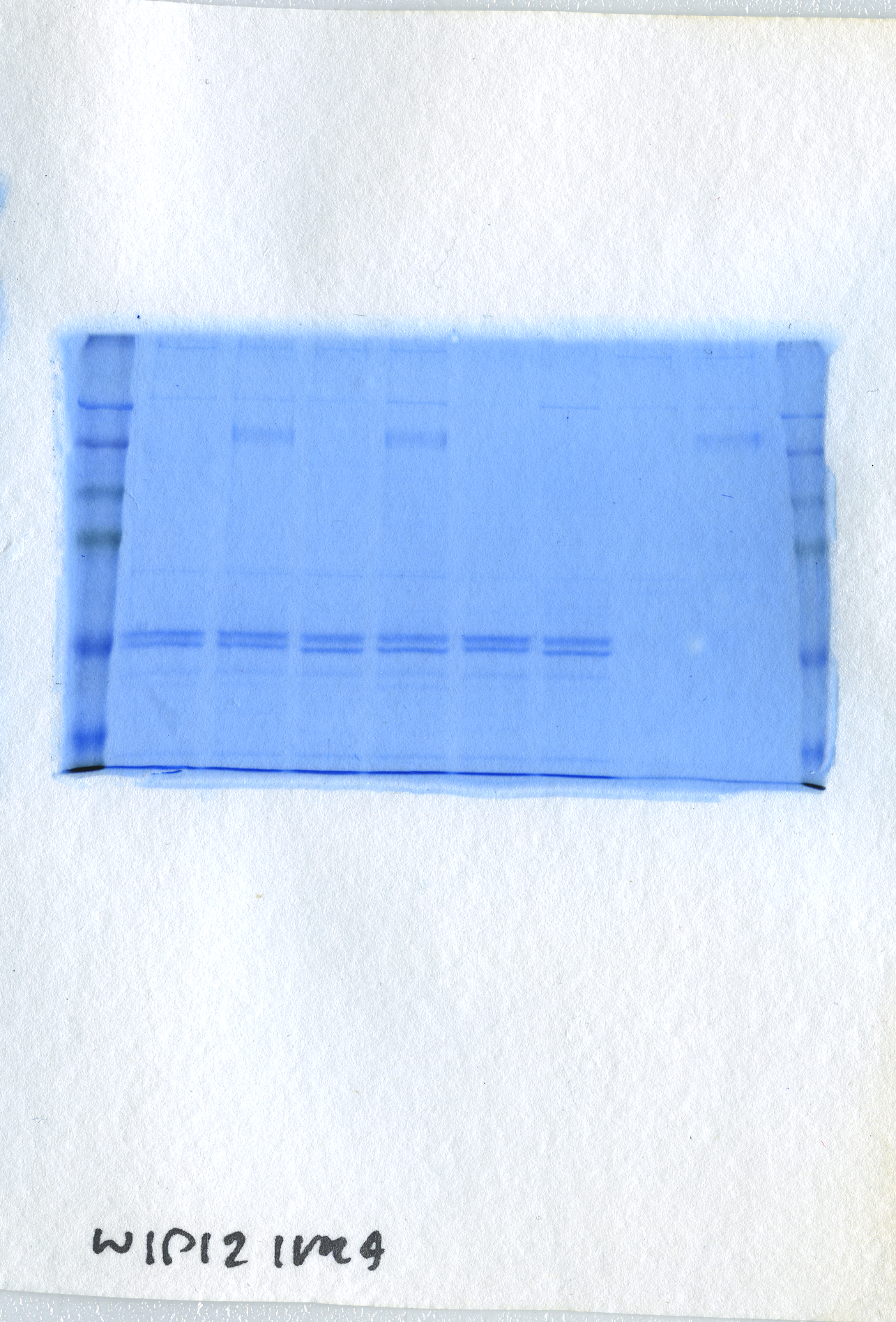

Supplement: Supplementary file 4 — Source data Fig. 2 [file 44319_2024_215_MOESM4_ESM.zip › Figure 2/2H/Images/gel.tif]

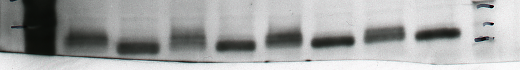

Supplement: Supplementary file 4 — Source data Fig. 2 [file 44319_2024_215_MOESM4_ESM.zip › Figure 2/2A/Images/western ATG13.tif]

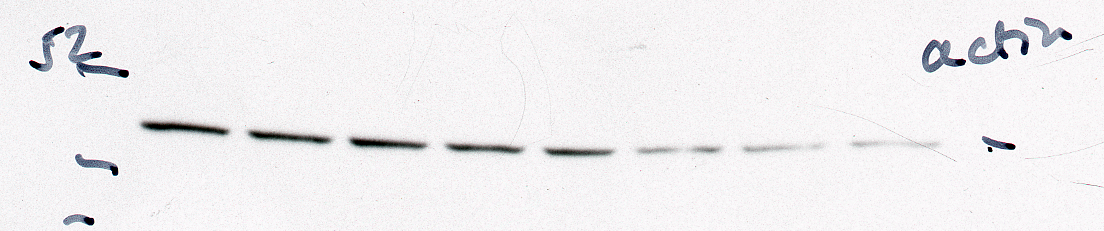

Supplement: Supplementary file 4 — Source data Fig. 2 [file 44319_2024_215_MOESM4_ESM.zip › Figure 2/2A/Images/western actin.tif]

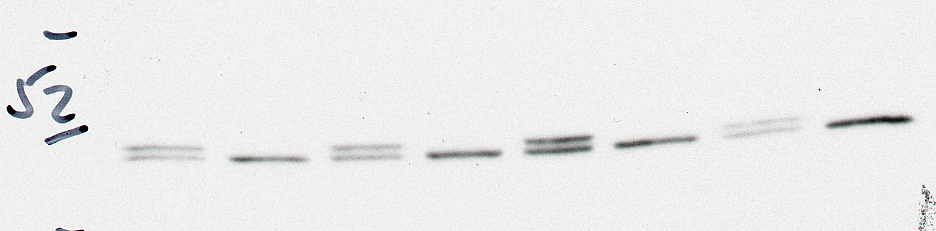

Supplement: Supplementary file 4 — Source data Fig. 2 [file 44319_2024_215_MOESM4_ESM.zip › Figure 2/2A/Images/western wipi2.tif]

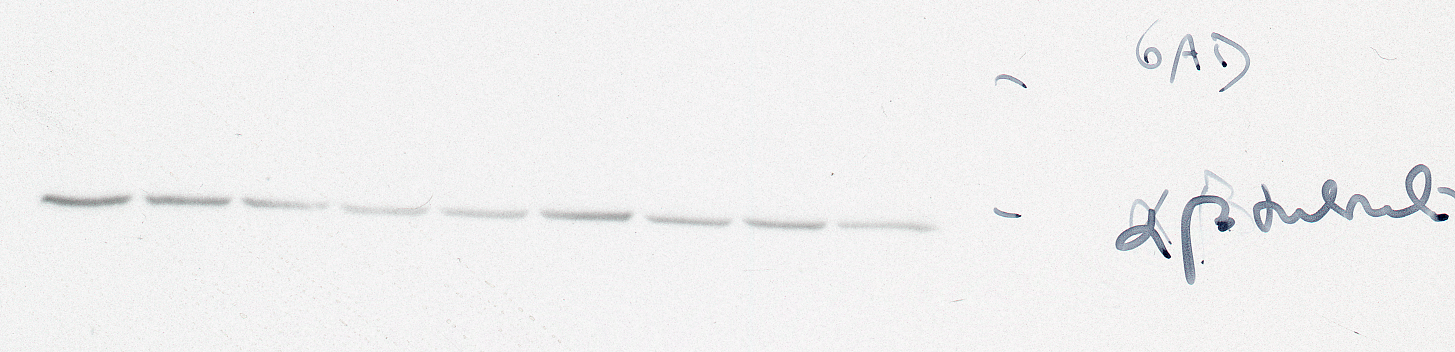

Supplement: Supplementary file 4 — Source data Fig. 2 [file 44319_2024_215_MOESM4_ESM.zip › Figure 2/2D/Images/western tubulin.tif]

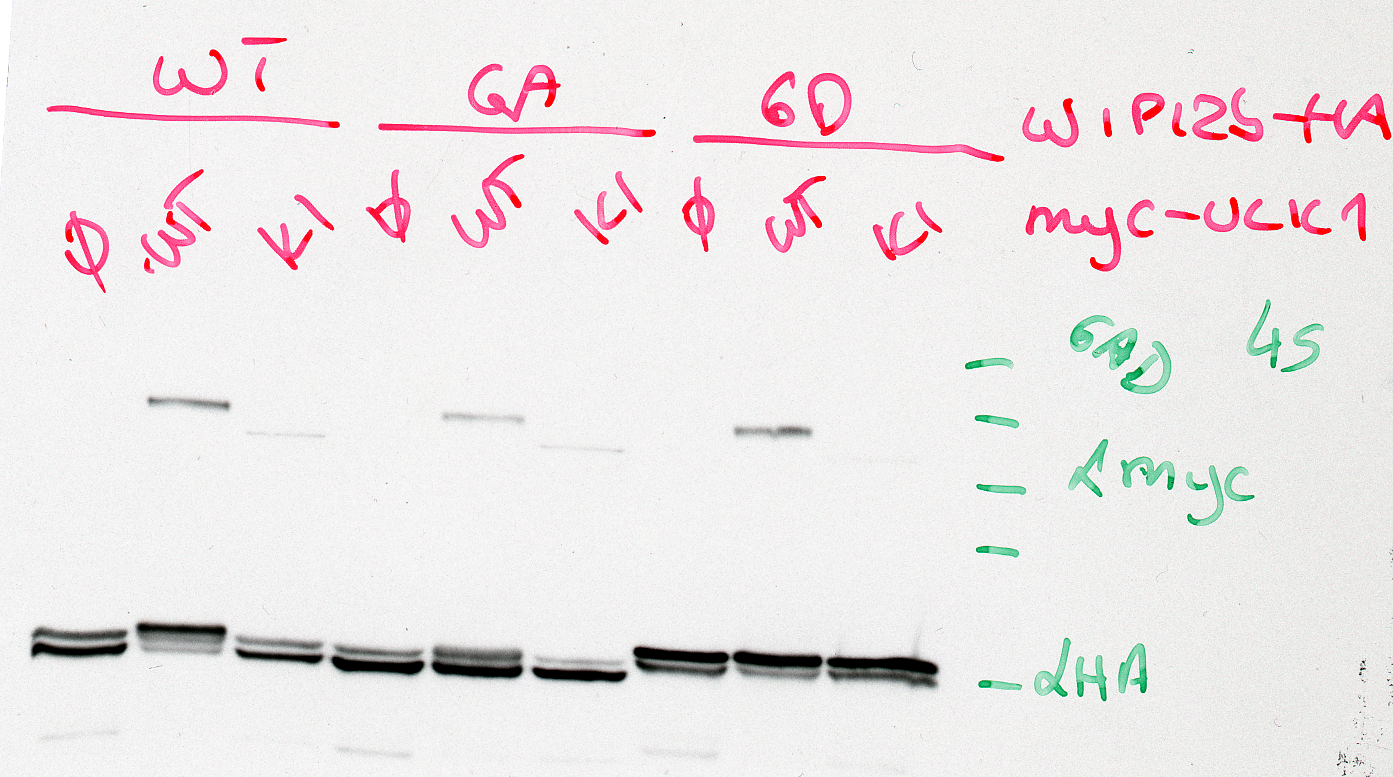

Supplement: Supplementary file 4 — Source data Fig. 2 [file 44319_2024_215_MOESM4_ESM.zip › Figure 2/2D/Images/western HA.tif]

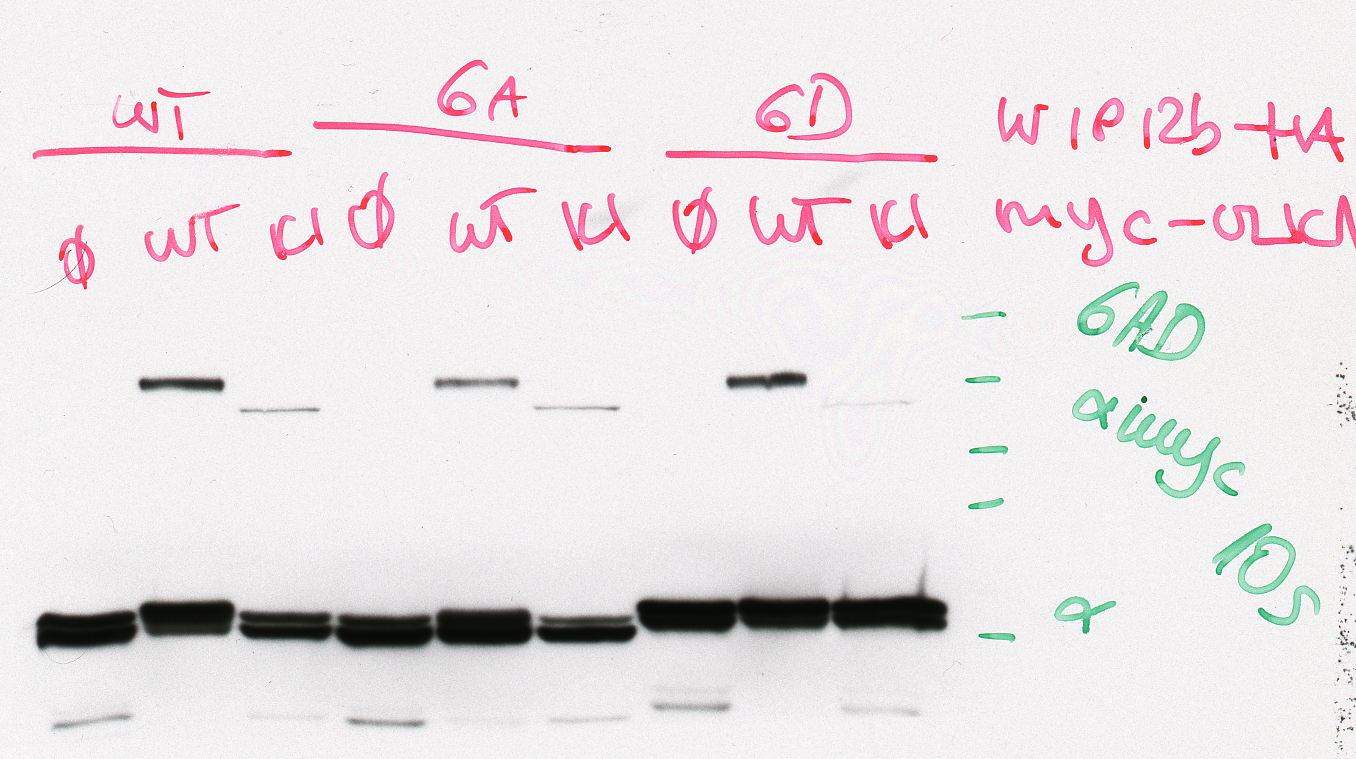

Supplement: Supplementary file 4 — Source data Fig. 2 [file 44319_2024_215_MOESM4_ESM.zip › Figure 2/2D/Images/western myc.tif]

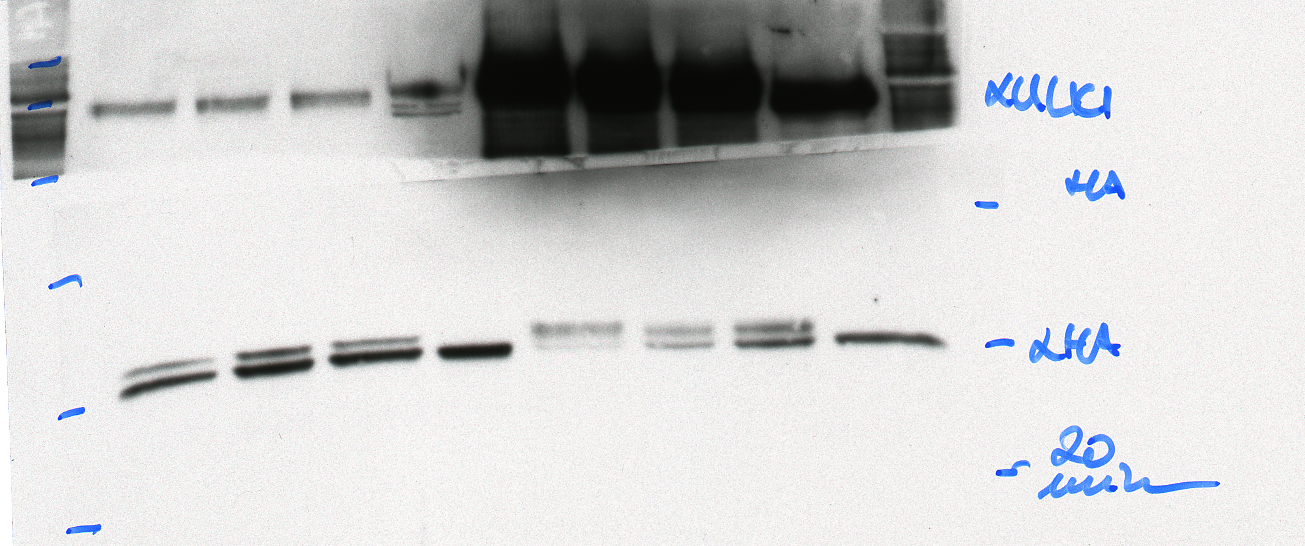

Supplement: Supplementary file 4 — Source data Fig. 2 [file 44319_2024_215_MOESM4_ESM.zip › Figure 2/2E/Images/western HA_ULK1_high.tif]

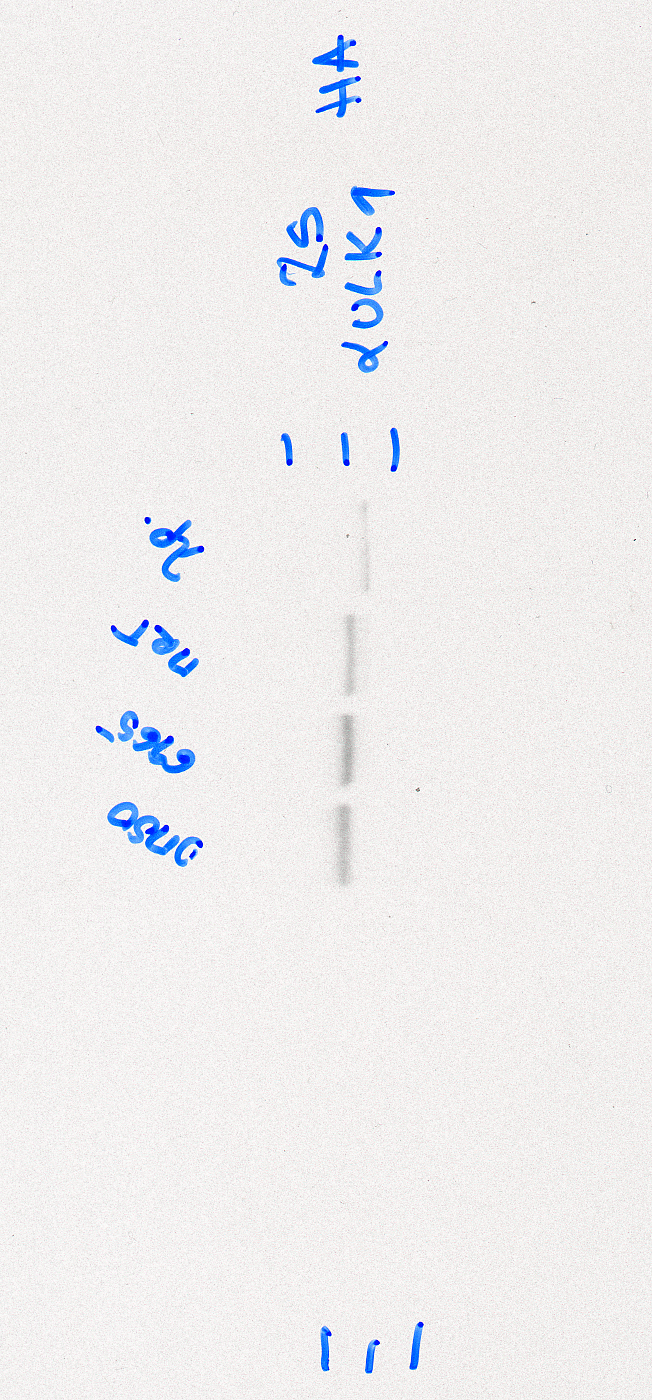

Supplement: Supplementary file 4 — Source data Fig. 2 [file 44319_2024_215_MOESM4_ESM.zip › Figure 2/2E/Images/western ULK1_low.tif]

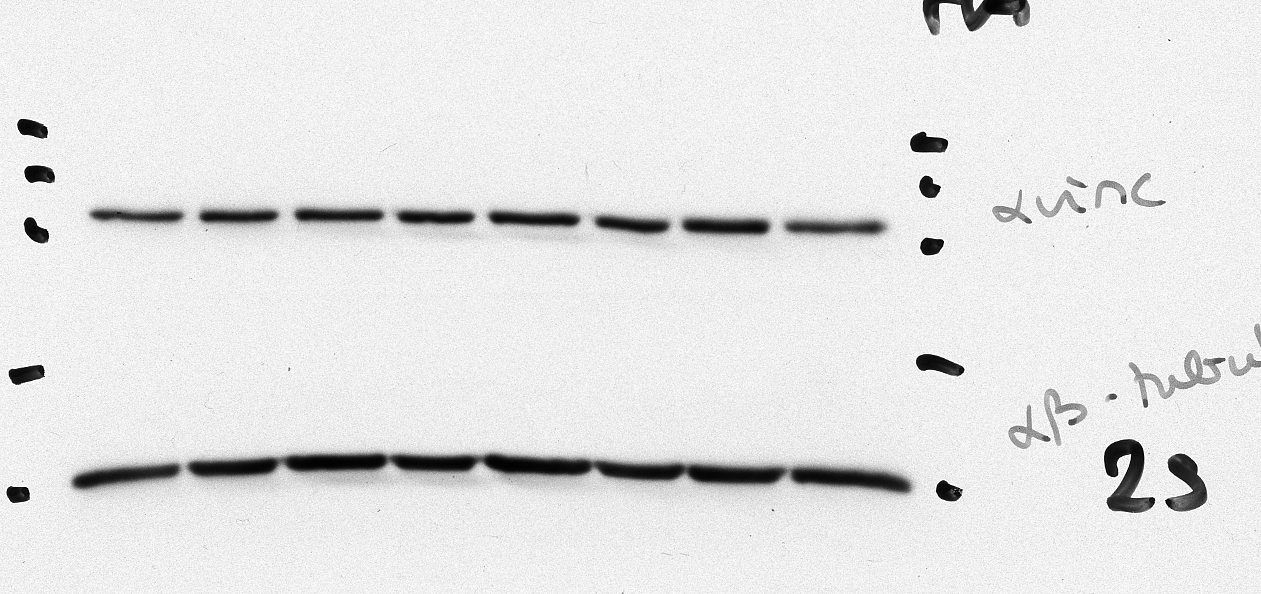

Supplement: Supplementary file 4 — Source data Fig. 2 [file 44319_2024_215_MOESM4_ESM.zip › Figure 2/2E/Images/western vinculin.tif]

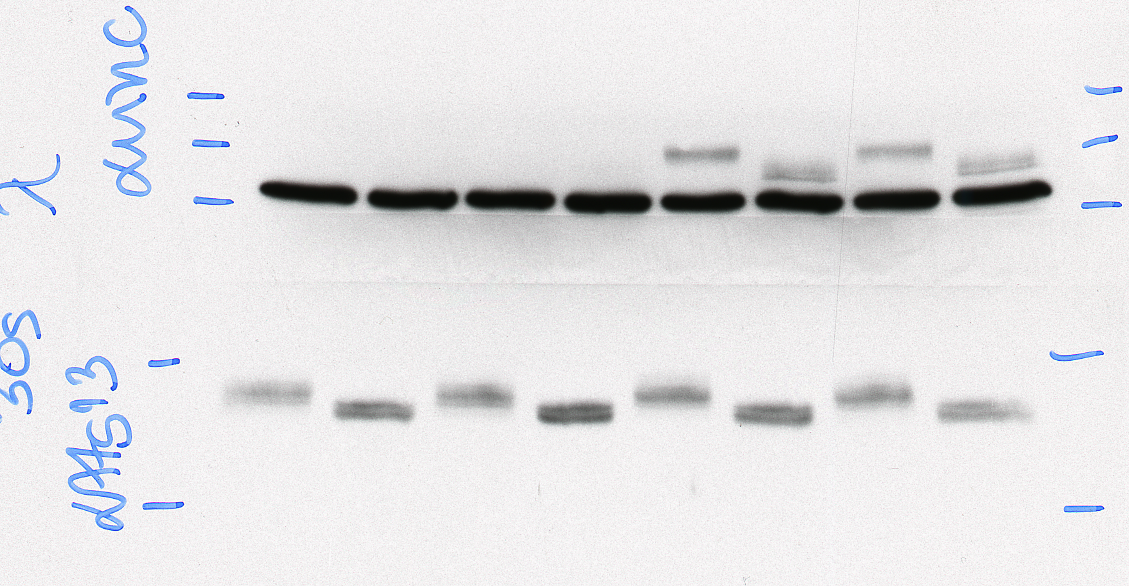

Supplement: Supplementary file 4 — Source data Fig. 2 [file 44319_2024_215_MOESM4_ESM.zip › Figure 2/2B/Images/western vinculin ATG13.tif]

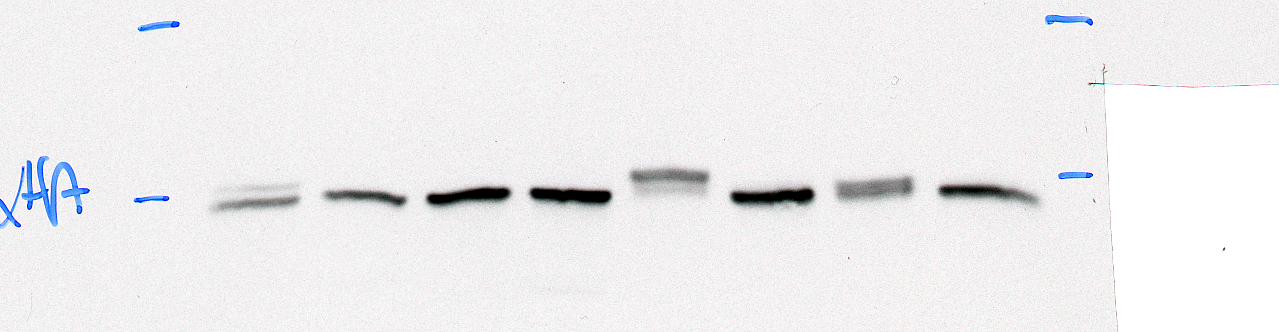

Supplement: Supplementary file 4 — Source data Fig. 2 [file 44319_2024_215_MOESM4_ESM.zip › Figure 2/2B/Images/western HA.tif]

Fed

Starved

WT

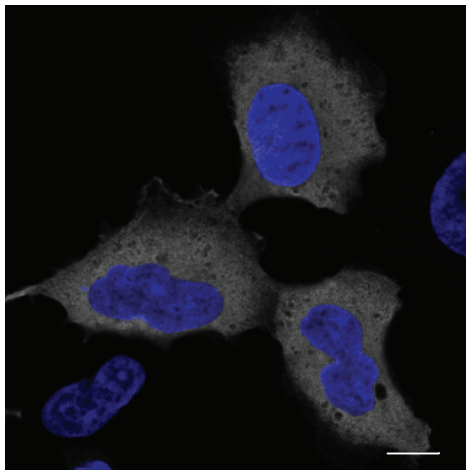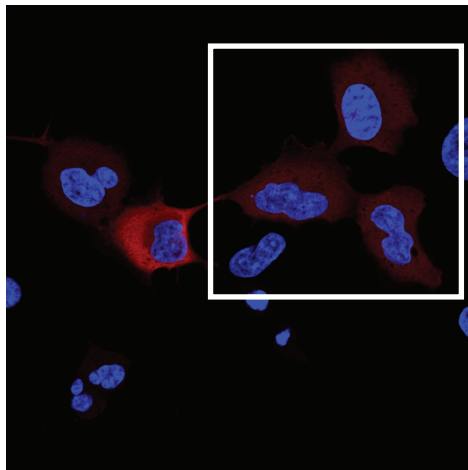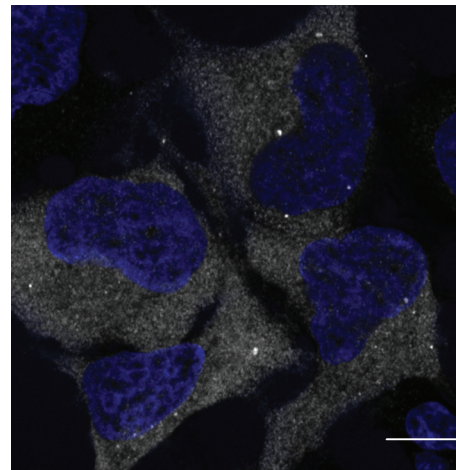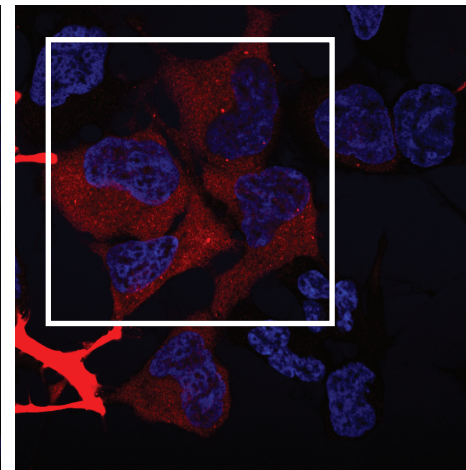

6A

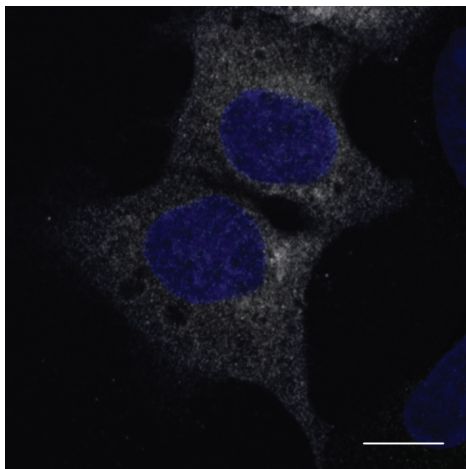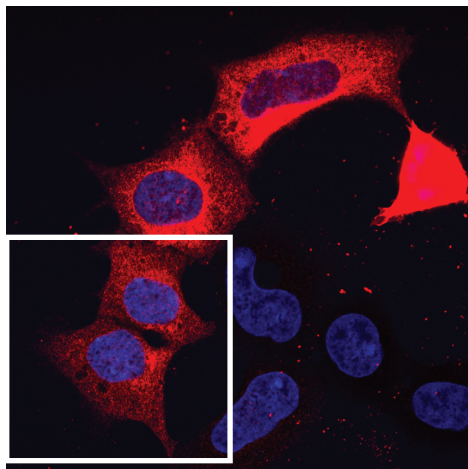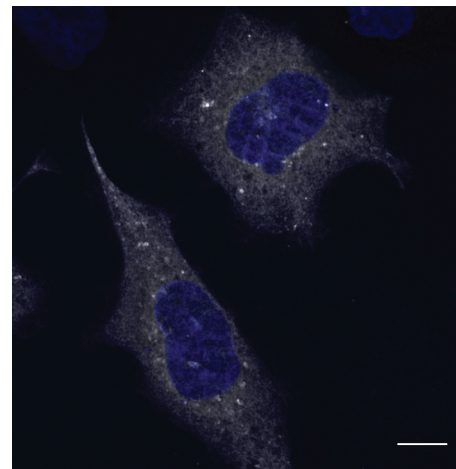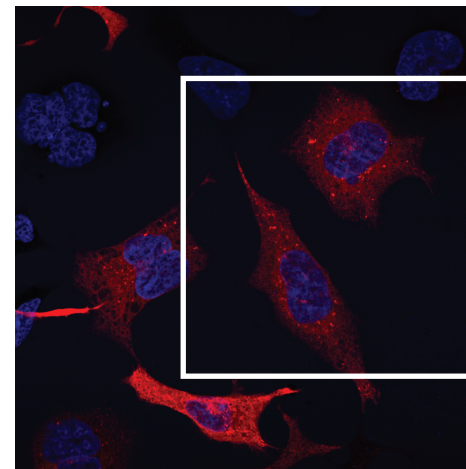

6D

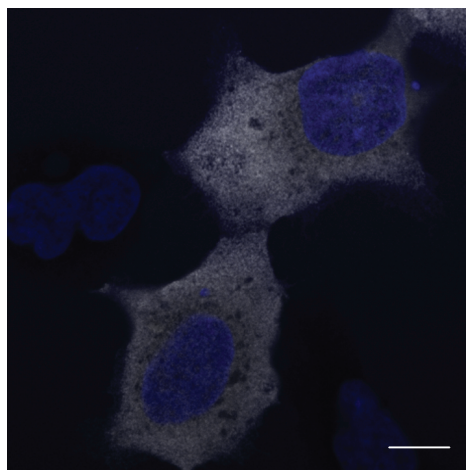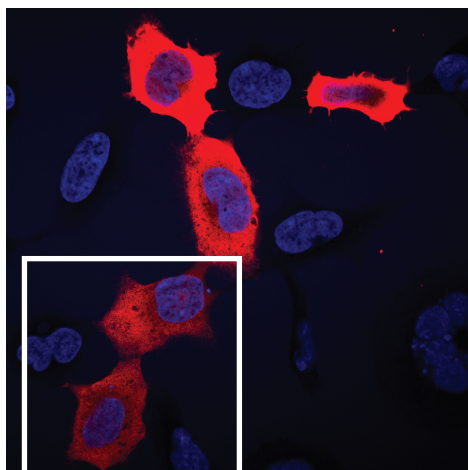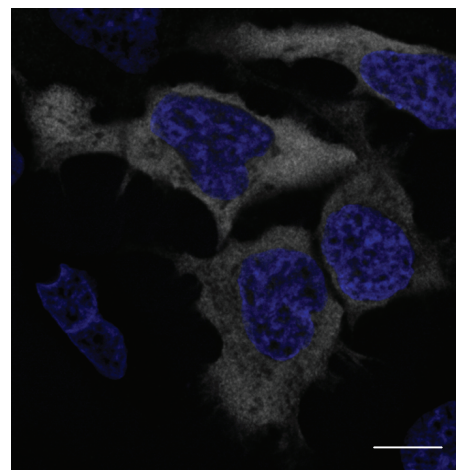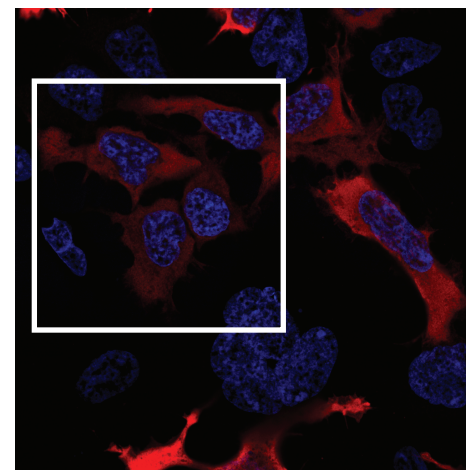

Supplement: Supplementary file 5 — Source data Fig. 3 [file 44319_2024_215_MOESM5_ESM.zip › Figure 3/3C/F3C.pdf]

Figure 3A

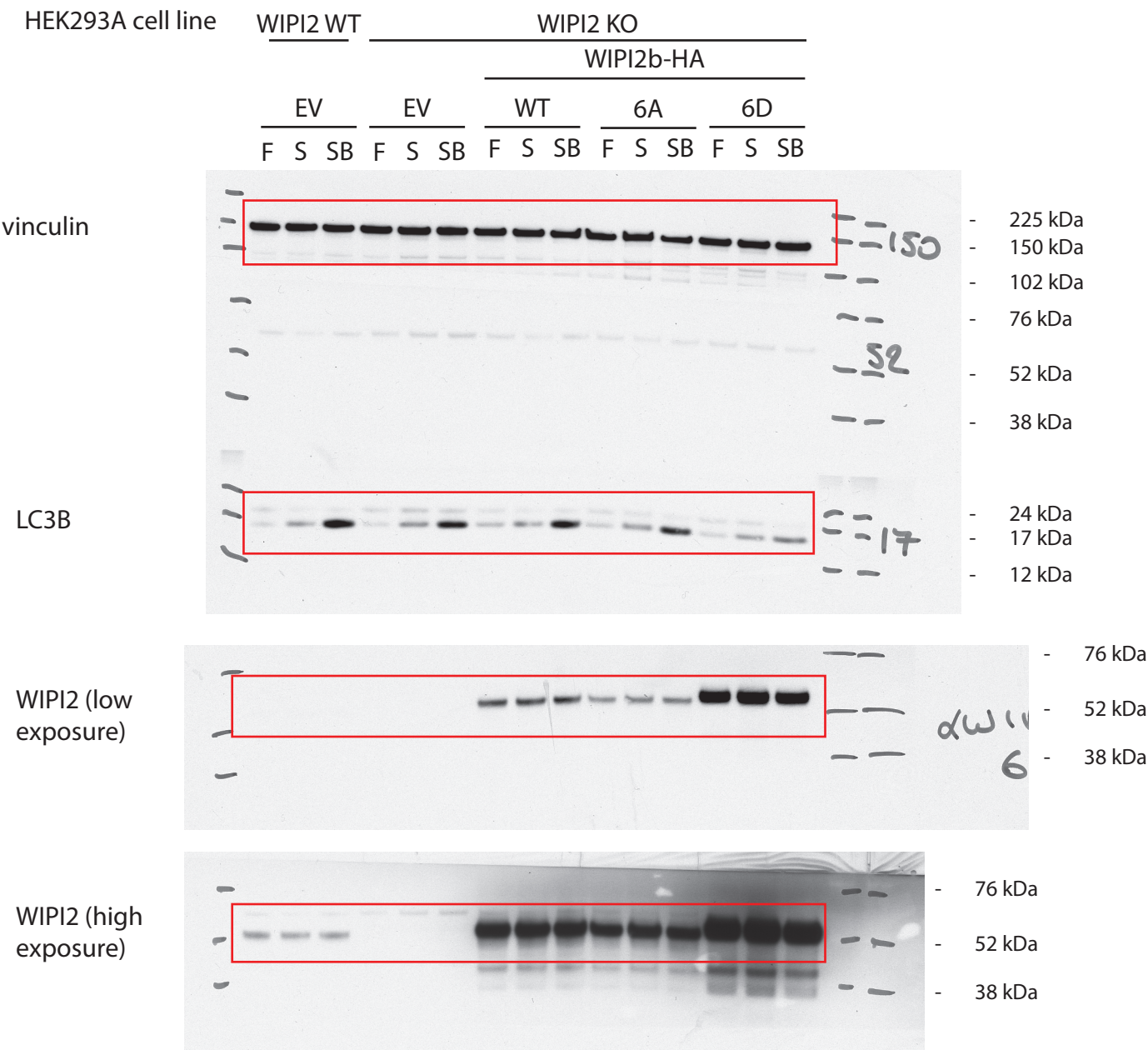

Supplement: Supplementary file 5 — Source data Fig. 3 [file 44319_2024_215_MOESM5_ESM.zip › Figure 3/3A/F3A.pdf]

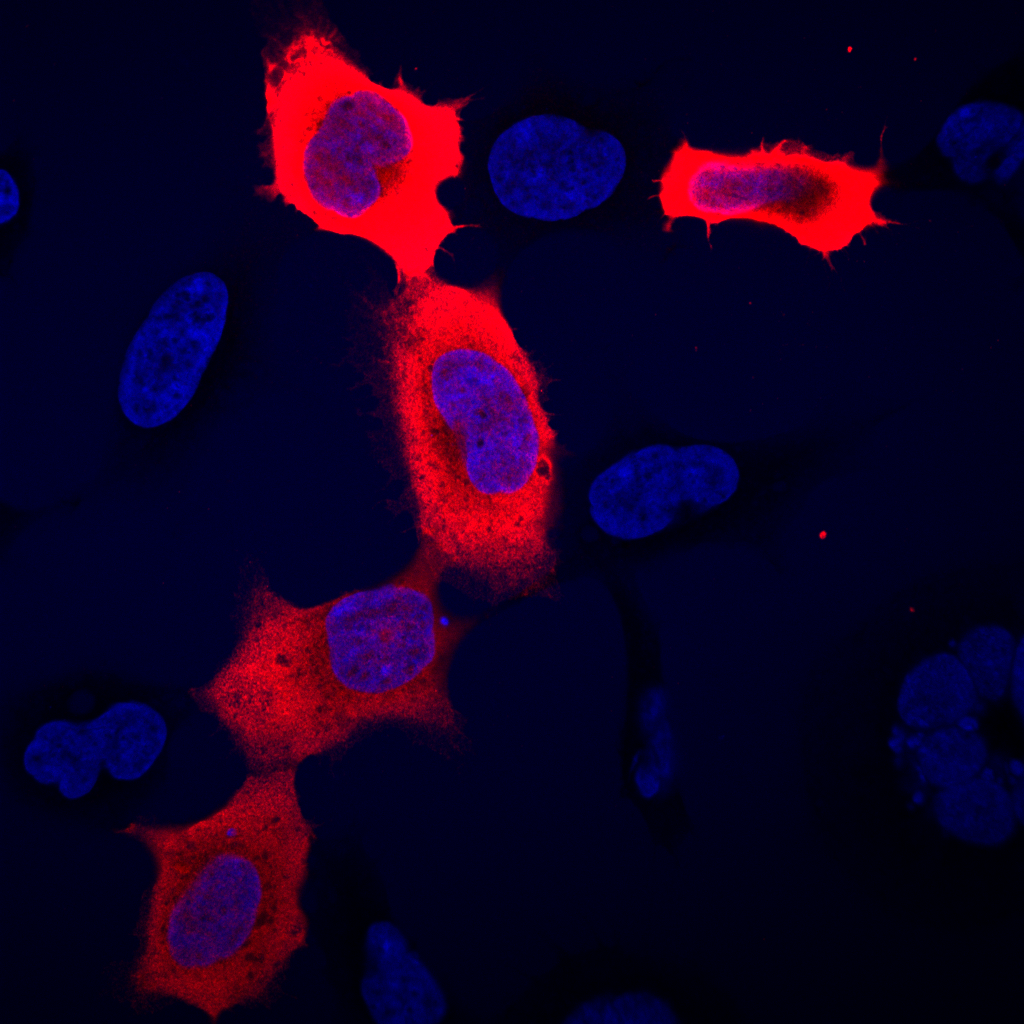

Supplement: Supplementary file 5 — Source data Fig. 3 [file 44319_2024_215_MOESM5_ESM.zip › Figure 3/3C/Fed/merge_6D.tif]

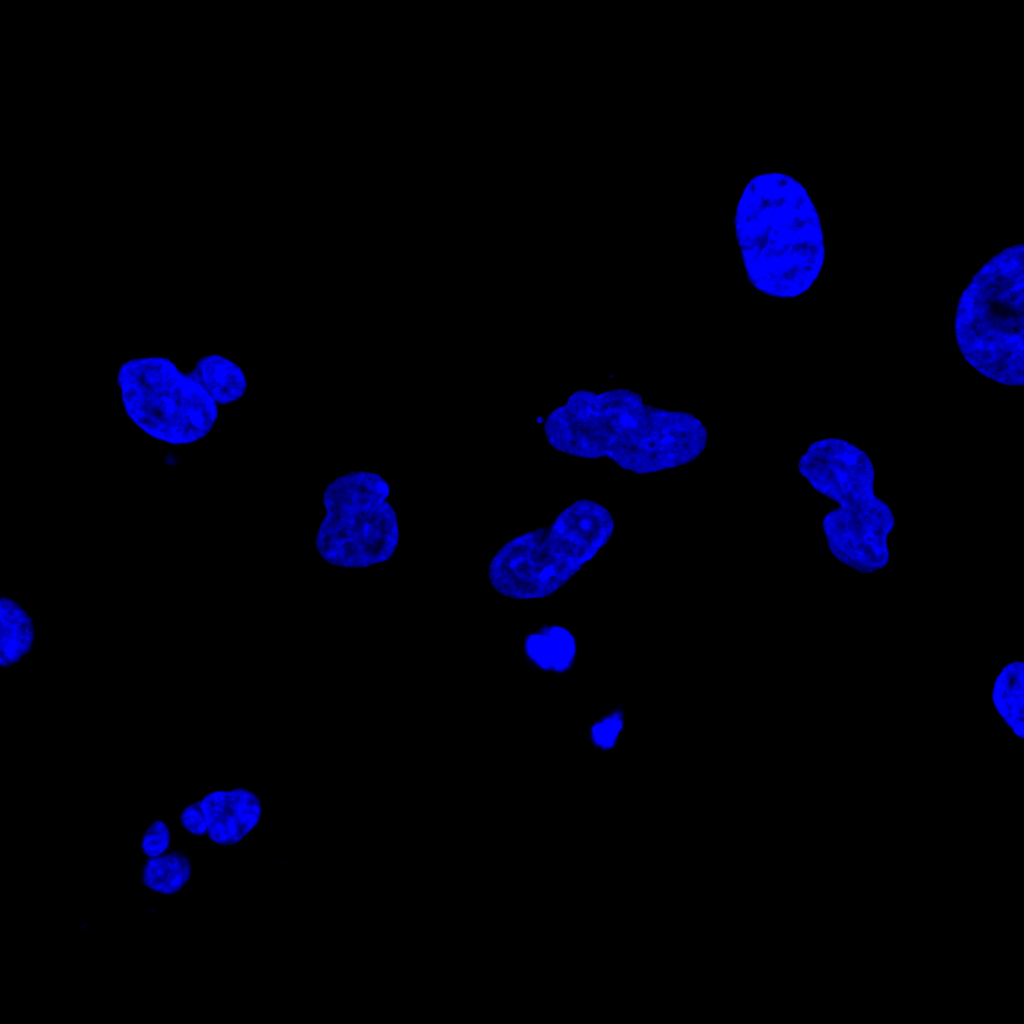

Supplement: Supplementary file 5 — Source data Fig. 3 [file 44319_2024_215_MOESM5_ESM.zip › Figure 3/3C/Fed/hoechst_WT.tif]

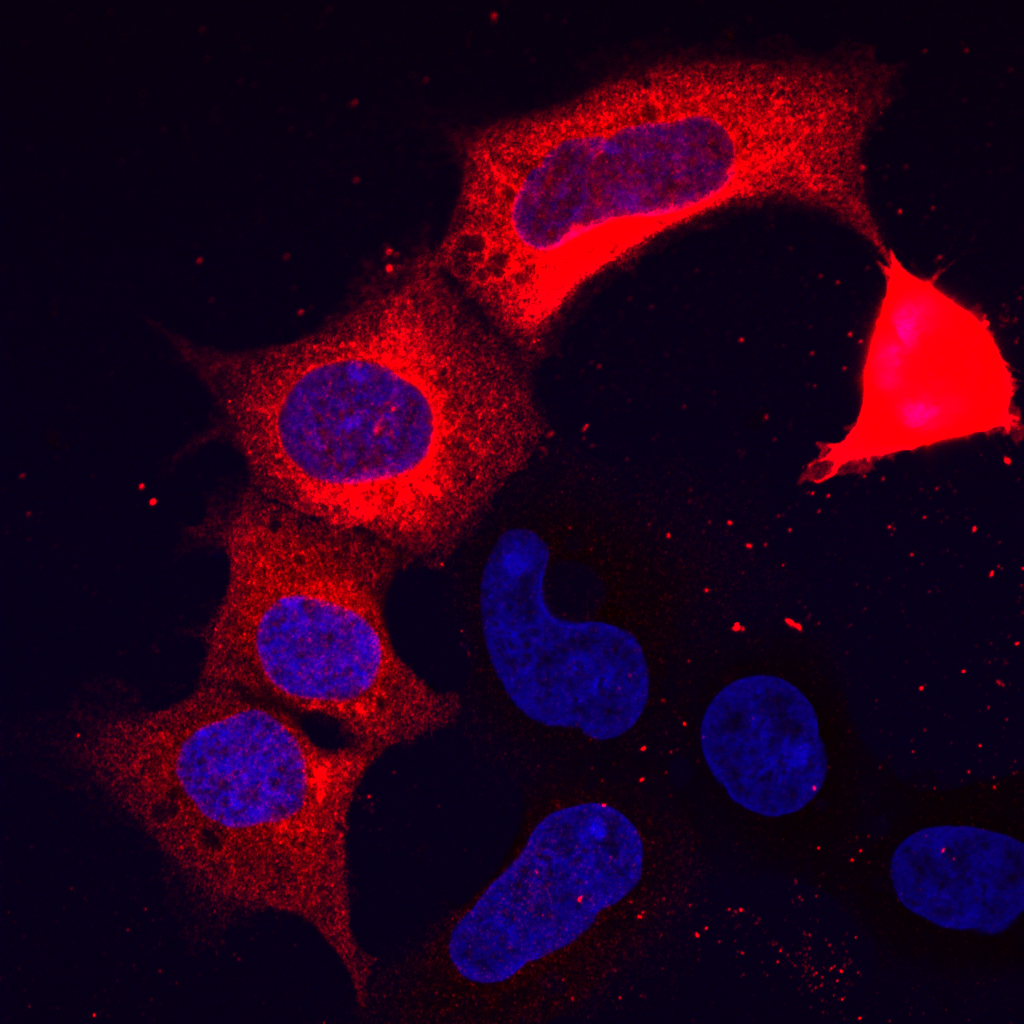

Supplement: Supplementary file 5 — Source data Fig. 3 [file 44319_2024_215_MOESM5_ESM.zip › Figure 3/3C/Fed/merge_6A.tif]

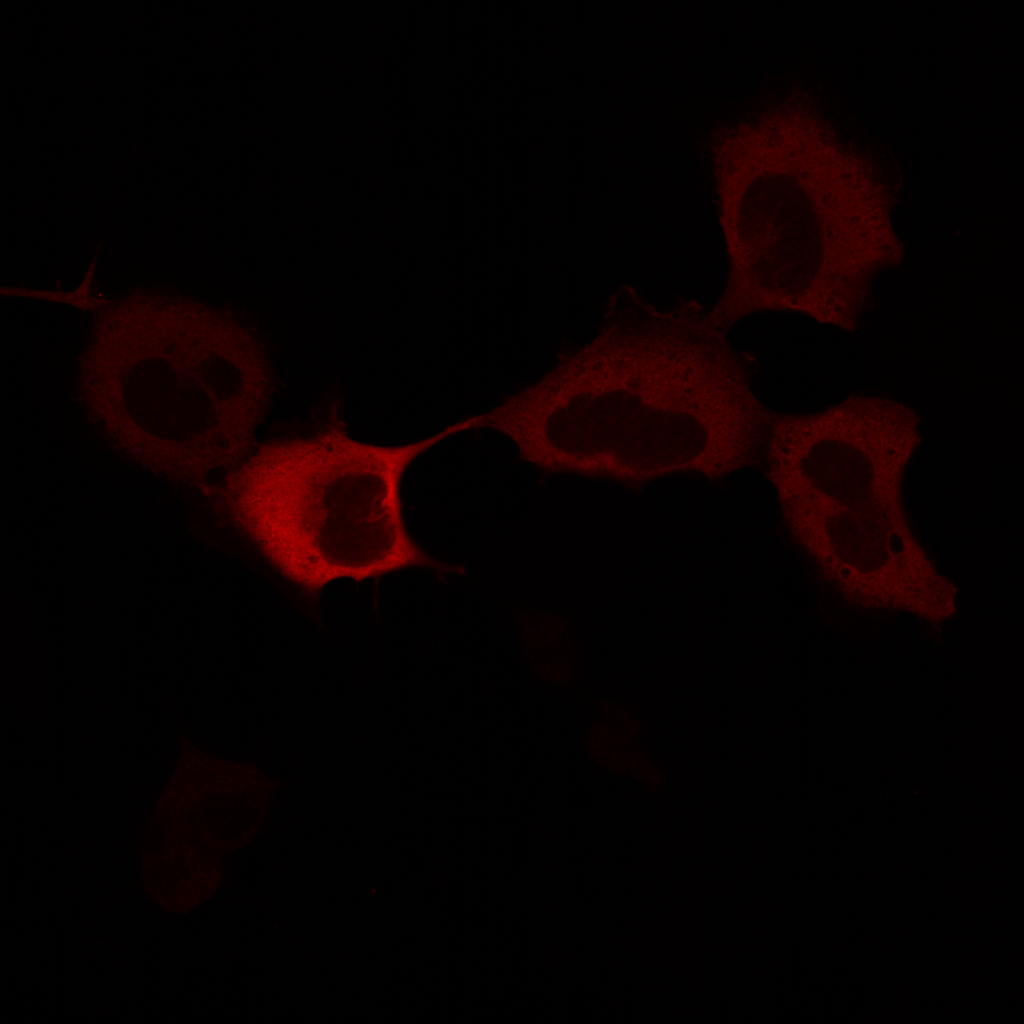

Supplement: Supplementary file 5 — Source data Fig. 3 [file 44319_2024_215_MOESM5_ESM.zip › Figure 3/3C/Fed/HA_WT.tif]

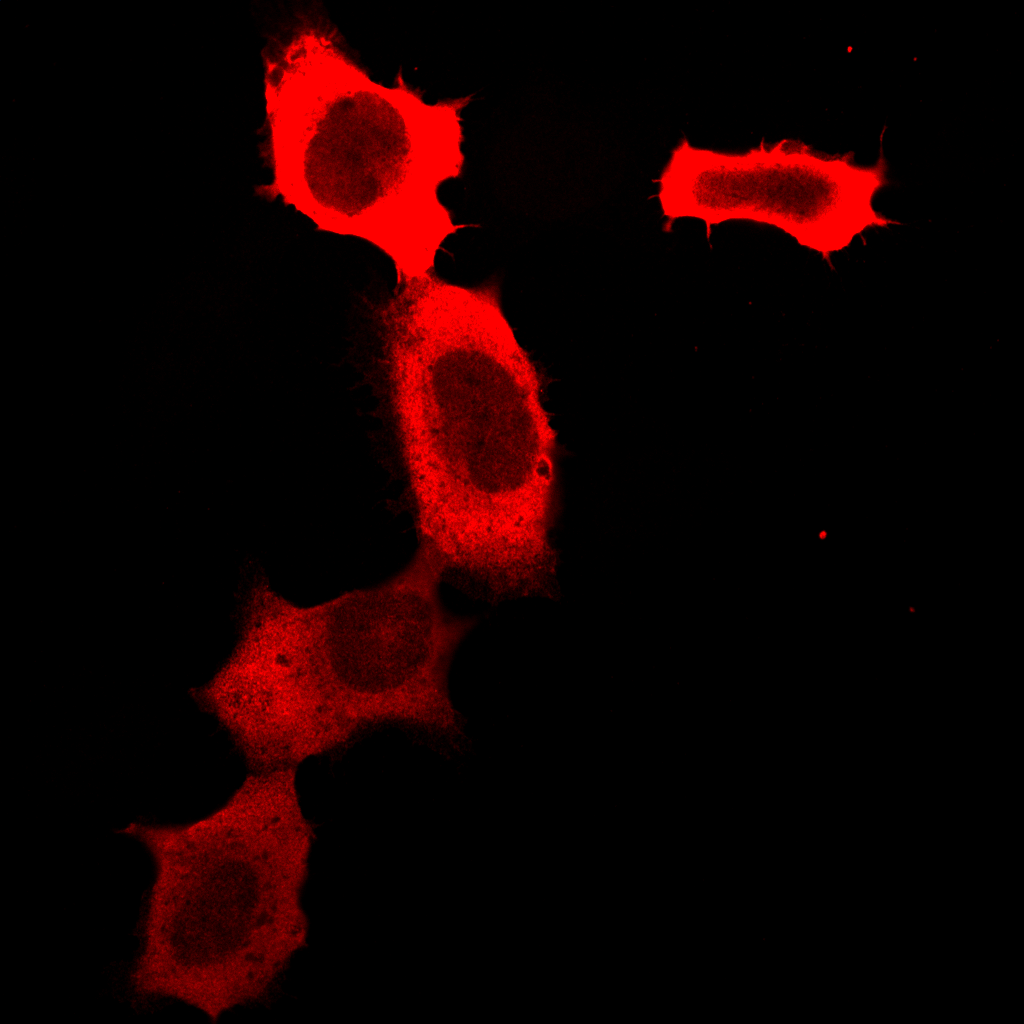

Supplement: Supplementary file 5 — Source data Fig. 3 [file 44319_2024_215_MOESM5_ESM.zip › Figure 3/3C/Fed/HA_6D.tif]

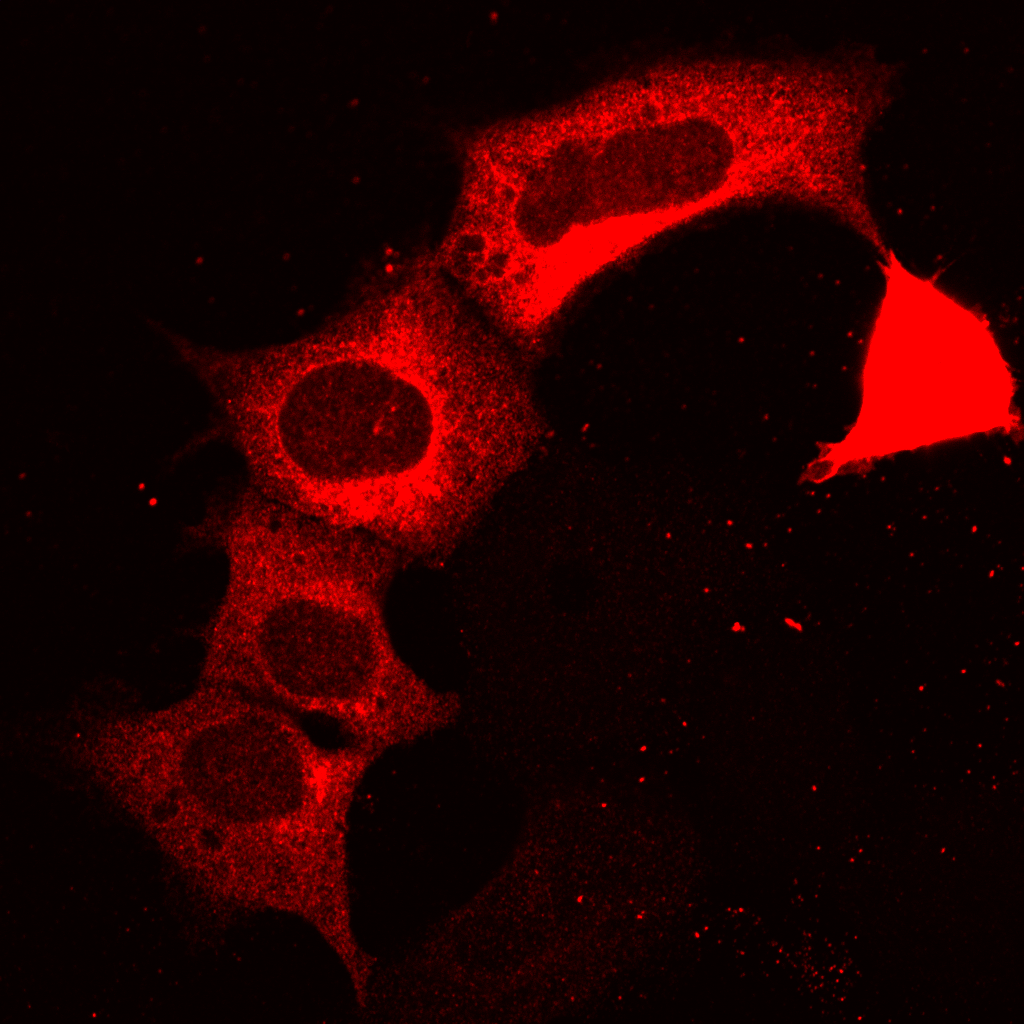

Supplement: Supplementary file 5 — Source data Fig. 3 [file 44319_2024_215_MOESM5_ESM.zip › Figure 3/3C/Fed/HA_6A.tif]

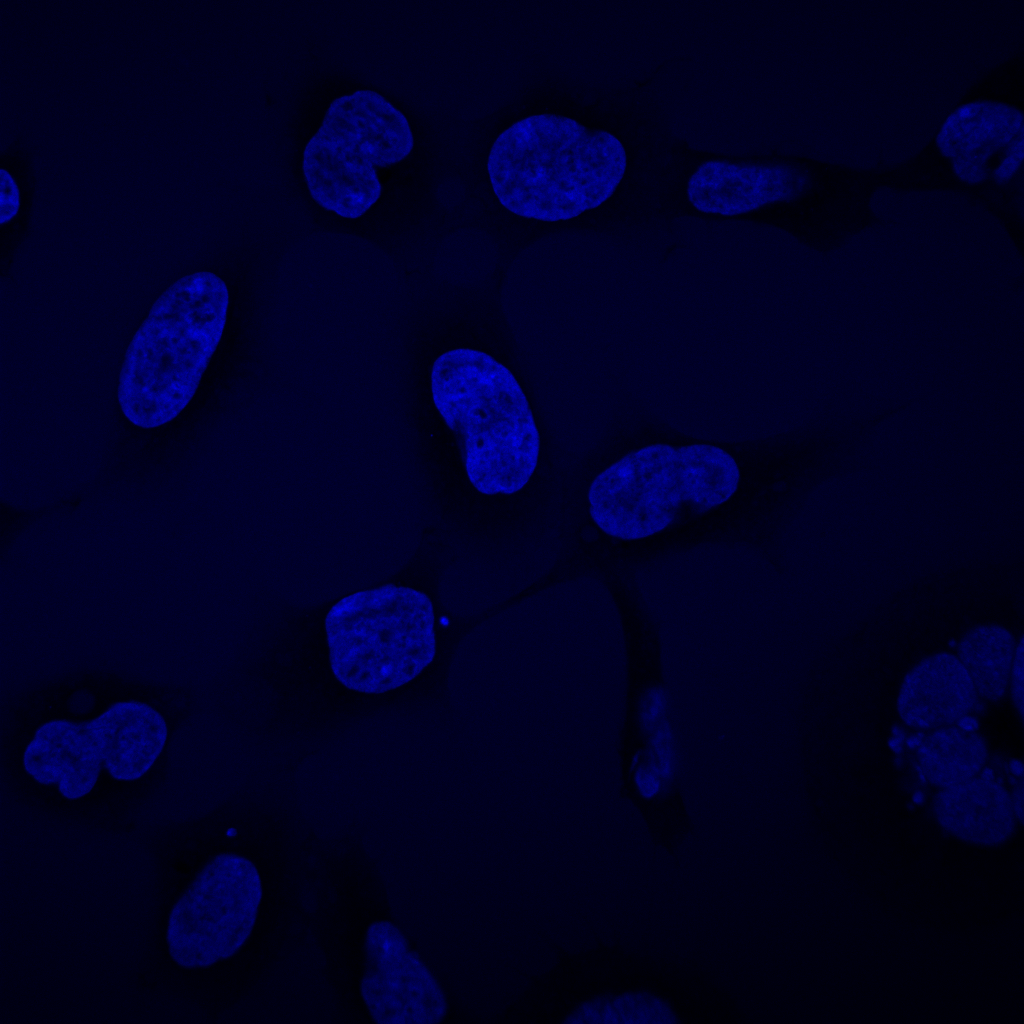

Supplement: Supplementary file 5 — Source data Fig. 3 [file 44319_2024_215_MOESM5_ESM.zip › Figure 3/3C/Fed/hoechst_6D.tif]

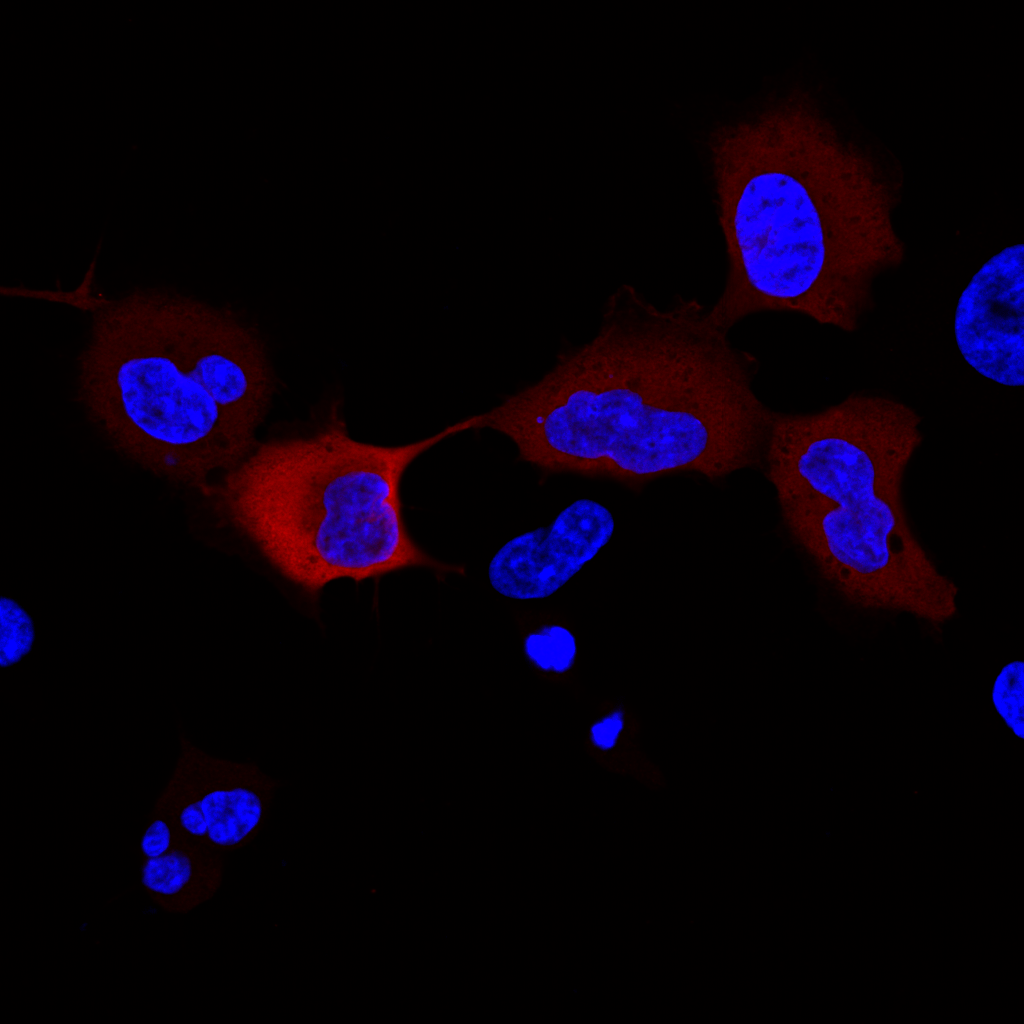

Supplement: Supplementary file 5 — Source data Fig. 3 [file 44319_2024_215_MOESM5_ESM.zip › Figure 3/3C/Fed/merge_WT.tif]

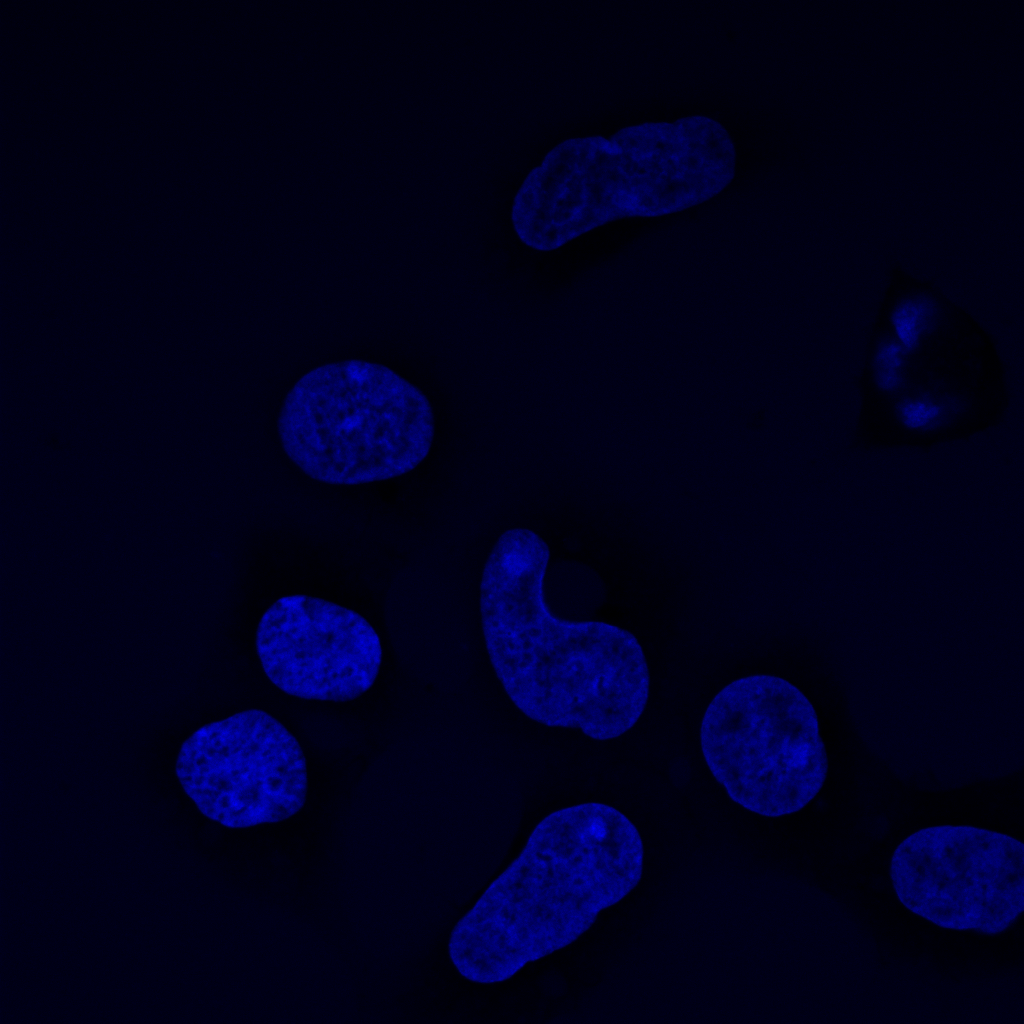

Supplement: Supplementary file 5 — Source data Fig. 3 [file 44319_2024_215_MOESM5_ESM.zip › Figure 3/3C/Fed/hoechst_6A.tif]

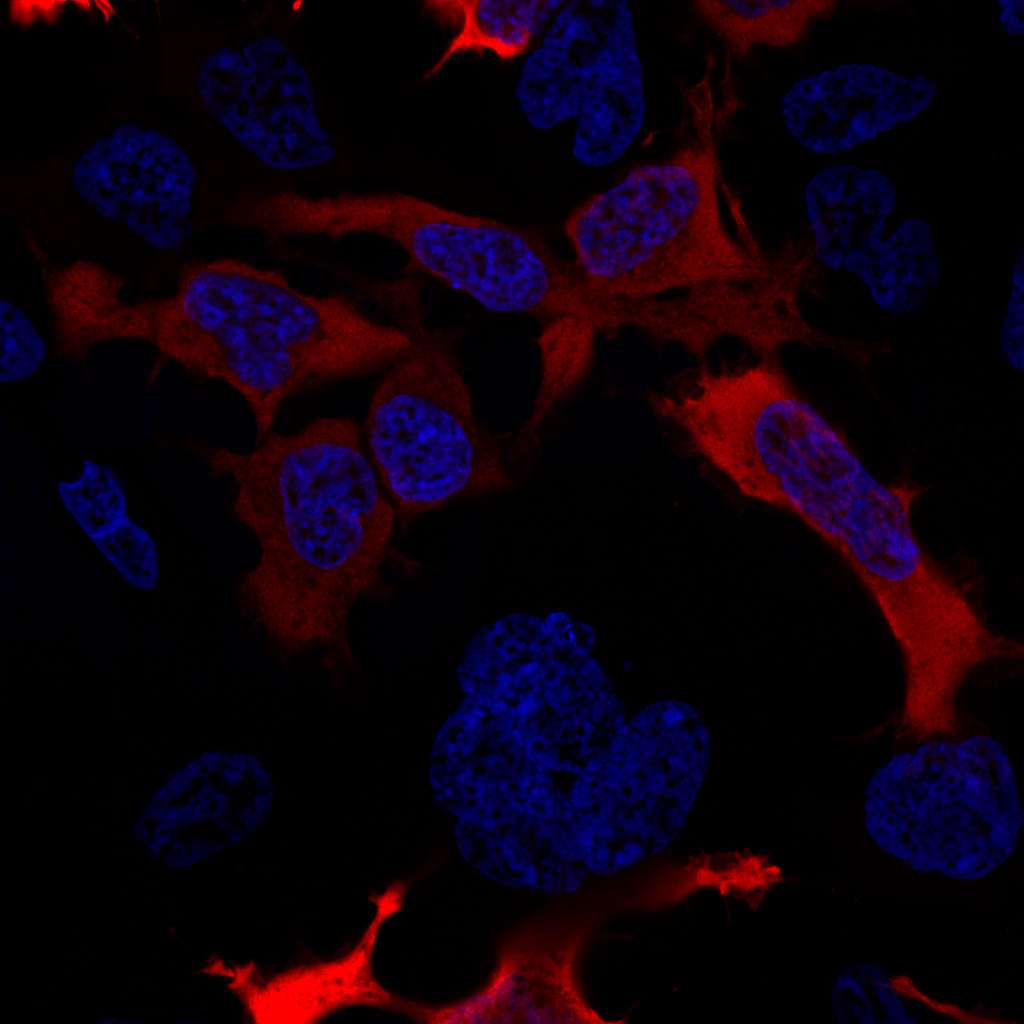

Supplement: Supplementary file 5 — Source data Fig. 3 [file 44319_2024_215_MOESM5_ESM.zip › Figure 3/3C/Starved/merge_6D.tif]

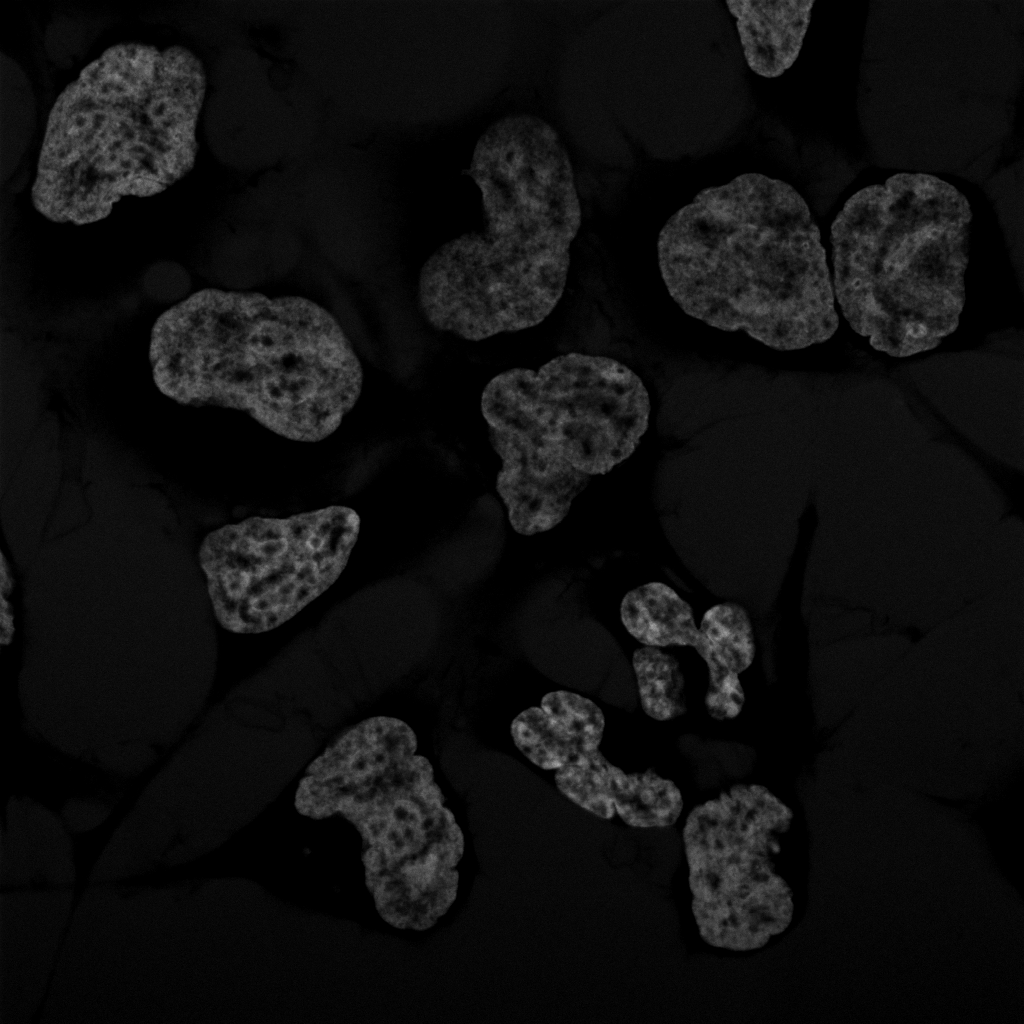

Supplement: Supplementary file 5 — Source data Fig. 3 [file 44319_2024_215_MOESM5_ESM.zip › Figure 3/3C/Starved/hoechst_WT.tif]

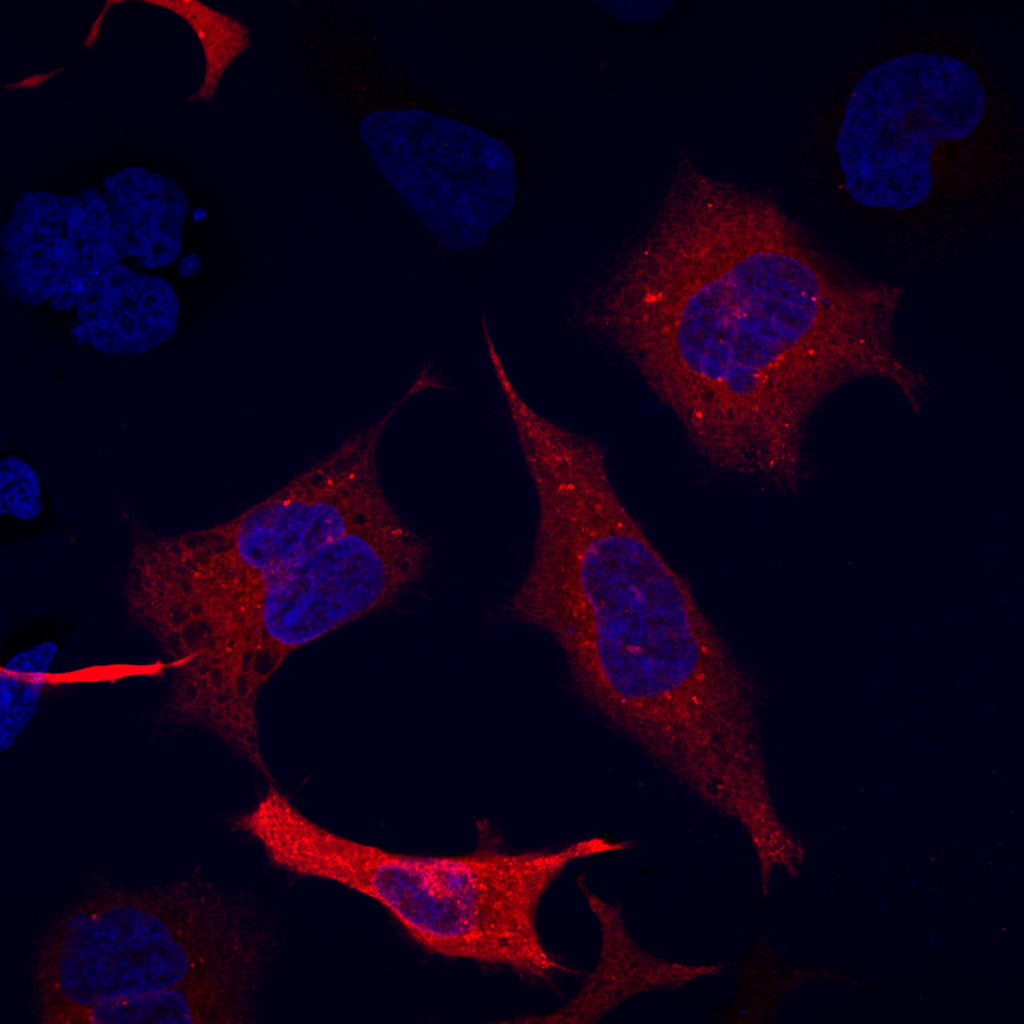

Supplement: Supplementary file 5 — Source data Fig. 3 [file 44319_2024_215_MOESM5_ESM.zip › Figure 3/3C/Starved/merge_6A.tif]

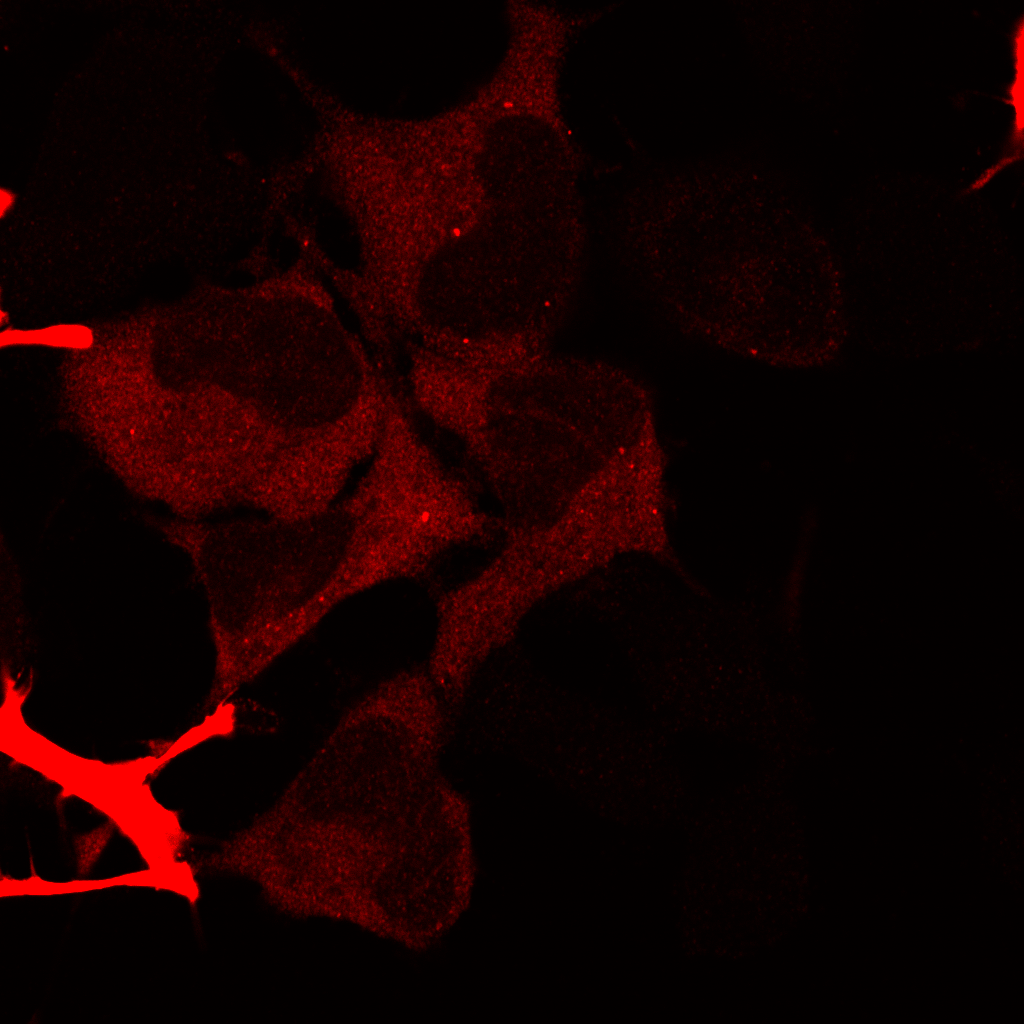

Supplement: Supplementary file 5 — Source data Fig. 3 [file 44319_2024_215_MOESM5_ESM.zip › Figure 3/3C/Starved/HA_WT.tif]

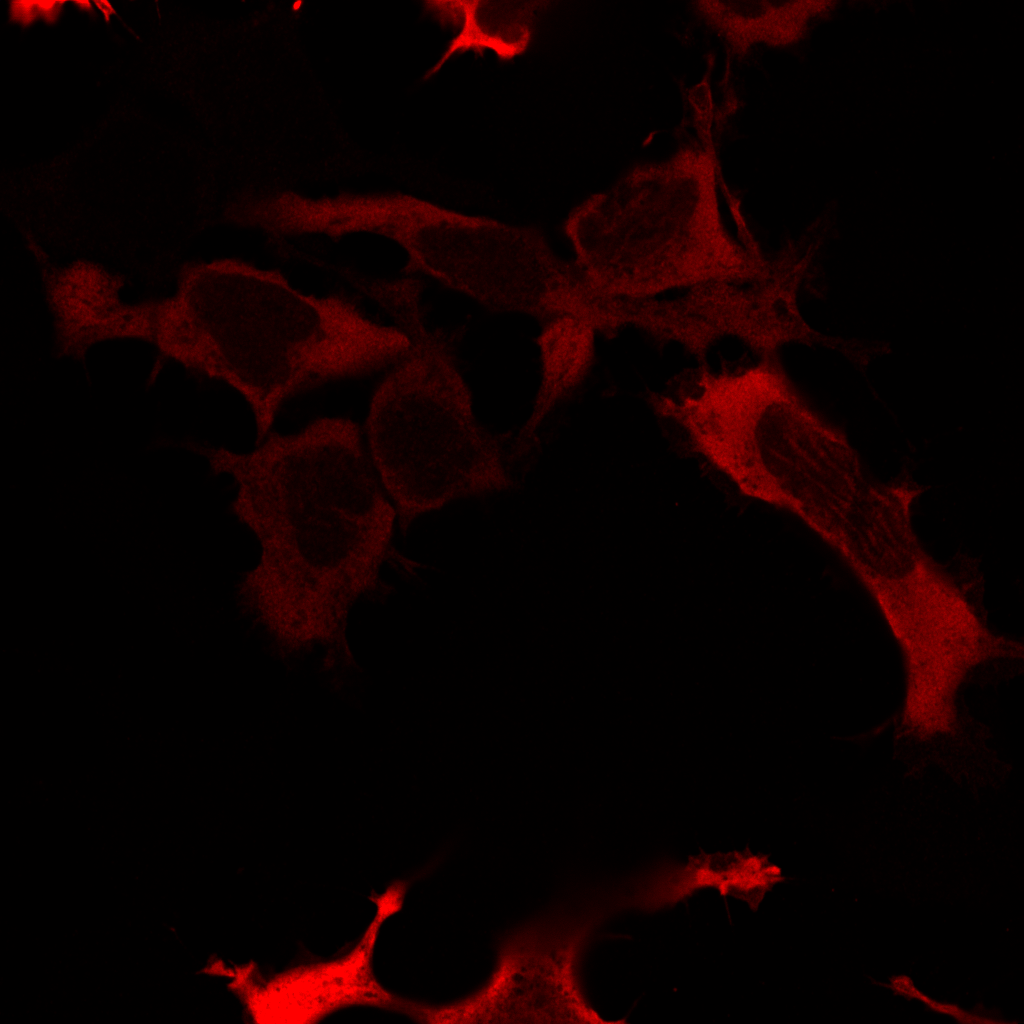

Supplement: Supplementary file 5 — Source data Fig. 3 [file 44319_2024_215_MOESM5_ESM.zip › Figure 3/3C/Starved/HA_6D.tif]

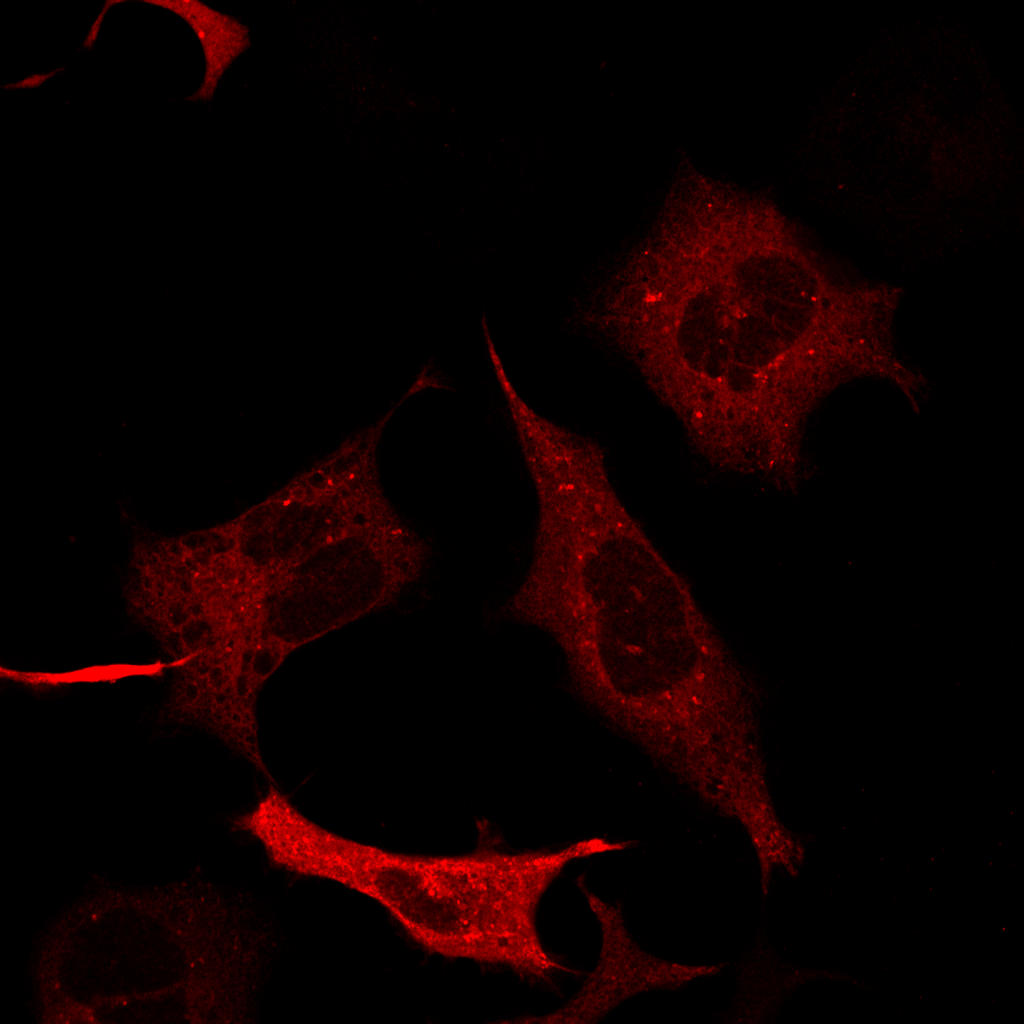

Supplement: Supplementary file 5 — Source data Fig. 3 [file 44319_2024_215_MOESM5_ESM.zip › Figure 3/3C/Starved/HA_6A.tif]

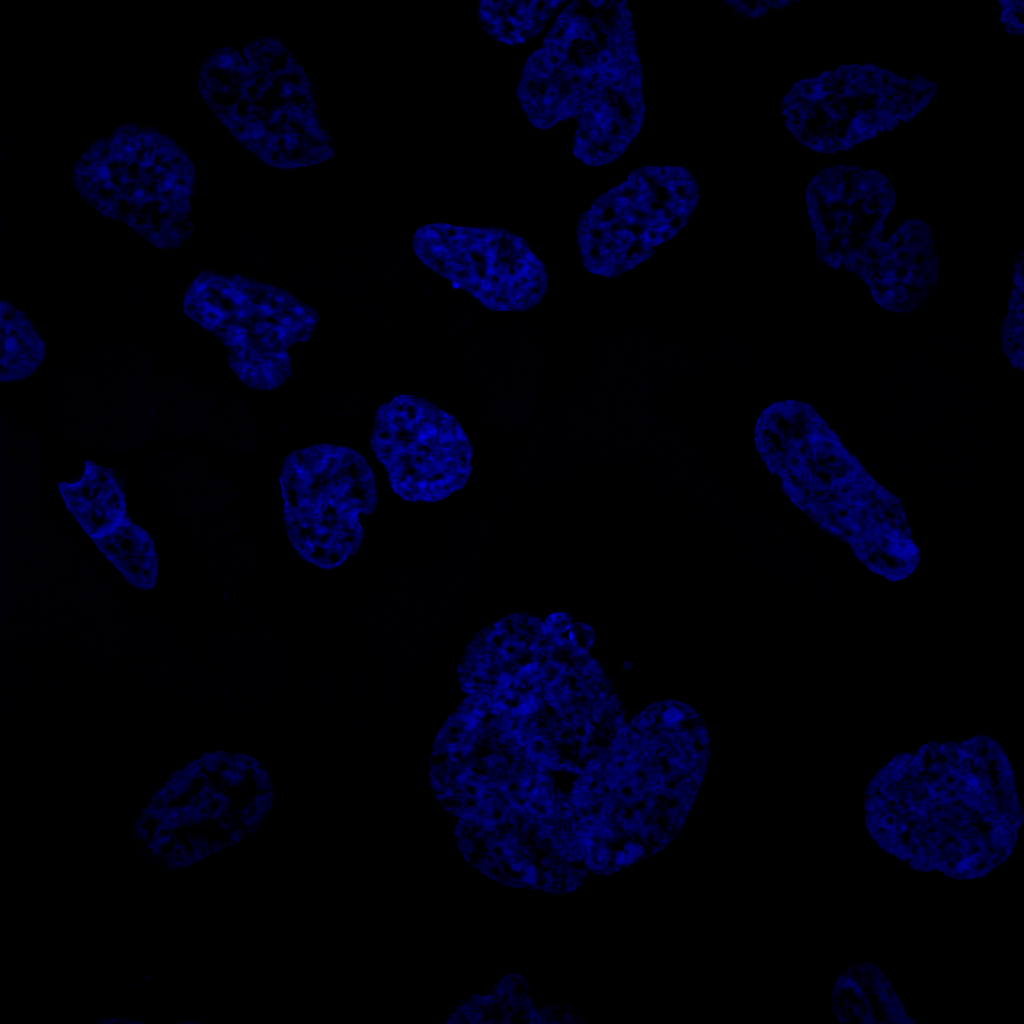

Supplement: Supplementary file 5 — Source data Fig. 3 [file 44319_2024_215_MOESM5_ESM.zip › Figure 3/3C/Starved/hoechst_6D.tif]

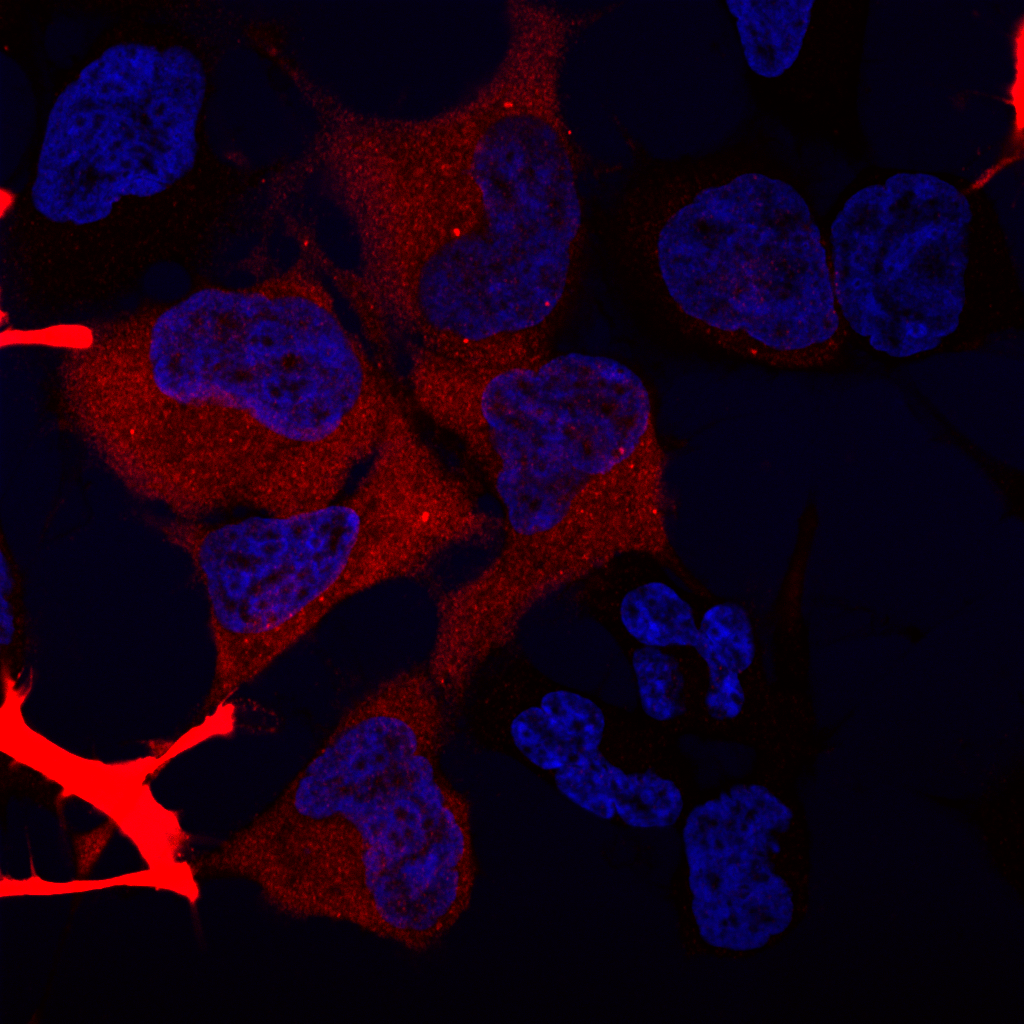

Supplement: Supplementary file 5 — Source data Fig. 3 [file 44319_2024_215_MOESM5_ESM.zip › Figure 3/3C/Starved/merge_WT.tif]

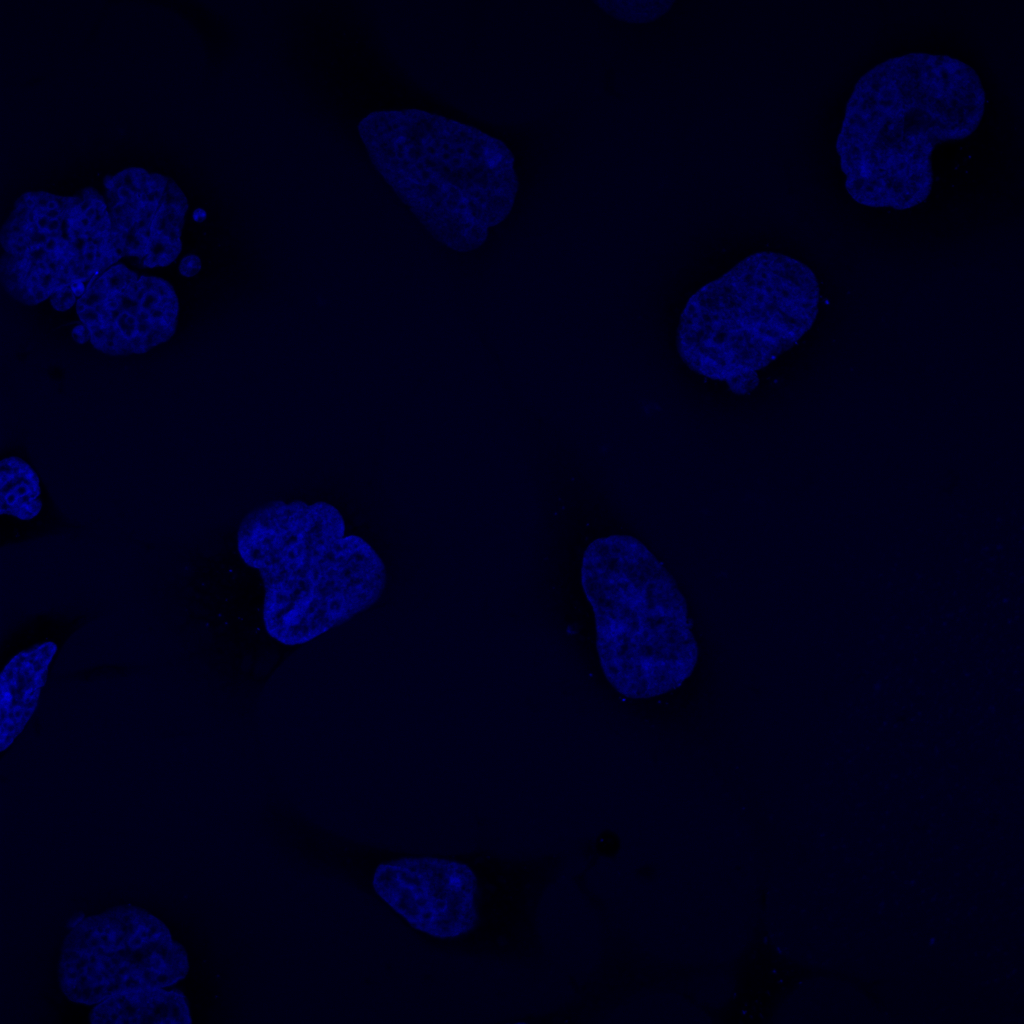

Supplement: Supplementary file 5 — Source data Fig. 3 [file 44319_2024_215_MOESM5_ESM.zip › Figure 3/3C/Starved/hoechst_6A.tif]

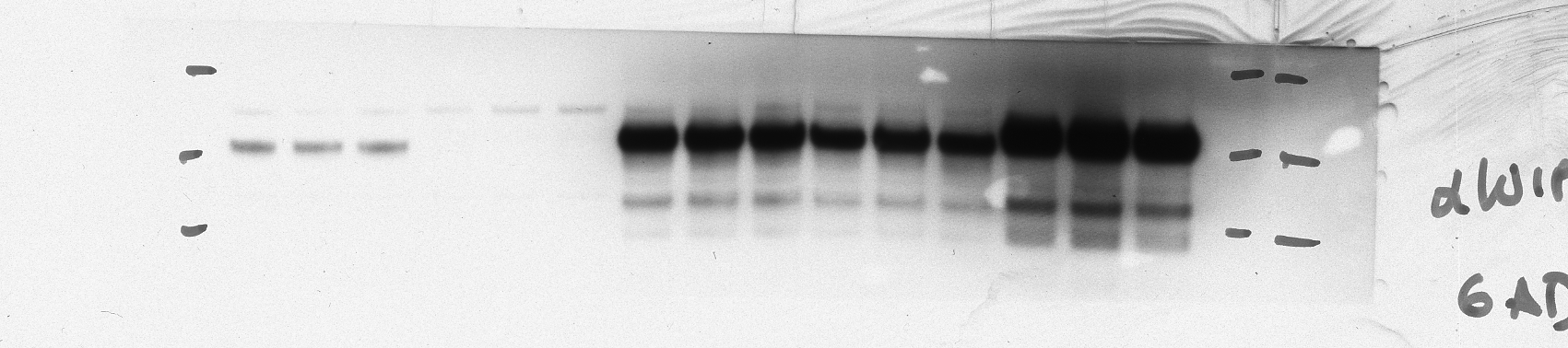

Supplement: Supplementary file 5 — Source data Fig. 3 [file 44319_2024_215_MOESM5_ESM.zip › Figure 3/3A/Images/western_WIPI2_high.tif]

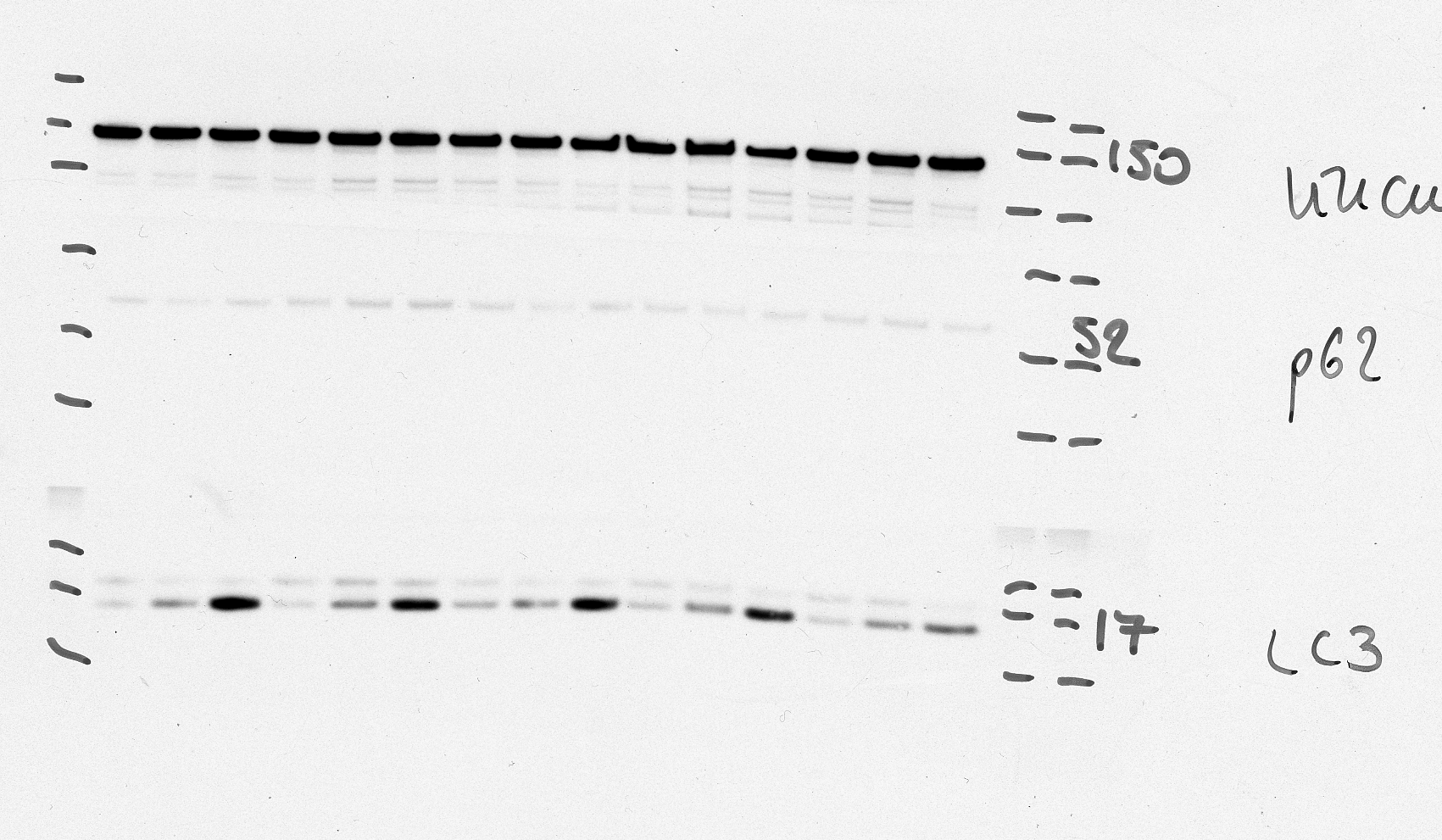

Supplement: Supplementary file 5 — Source data Fig. 3 [file 44319_2024_215_MOESM5_ESM.zip › Figure 3/3A/Images/western vinculin_LC3B.tif]

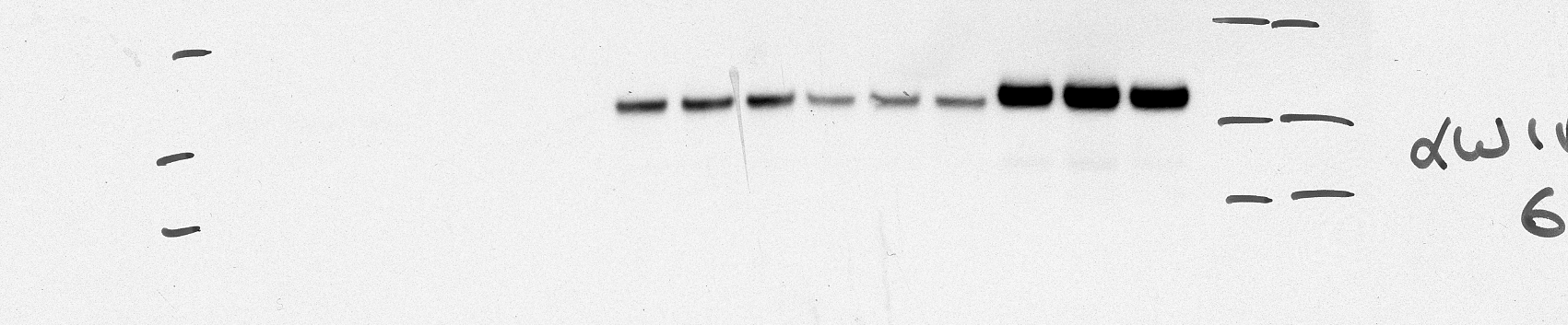

Supplement: Supplementary file 5 — Source data Fig. 3 [file 44319_2024_215_MOESM5_ESM.zip › Figure 3/3A/Images/western_WIPI2_low.tif]

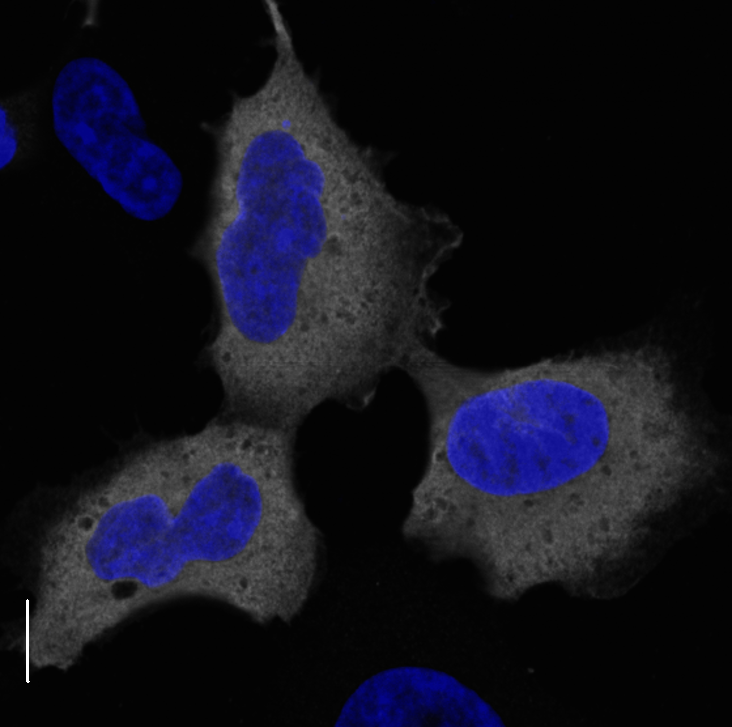

Supplement: Supplementary file 5 — Source data Fig. 3 [file 44319_2024_215_MOESM5_ESM.zip › Figure 3/3C/Fed/Greyscale merge/HA_WT.tif]

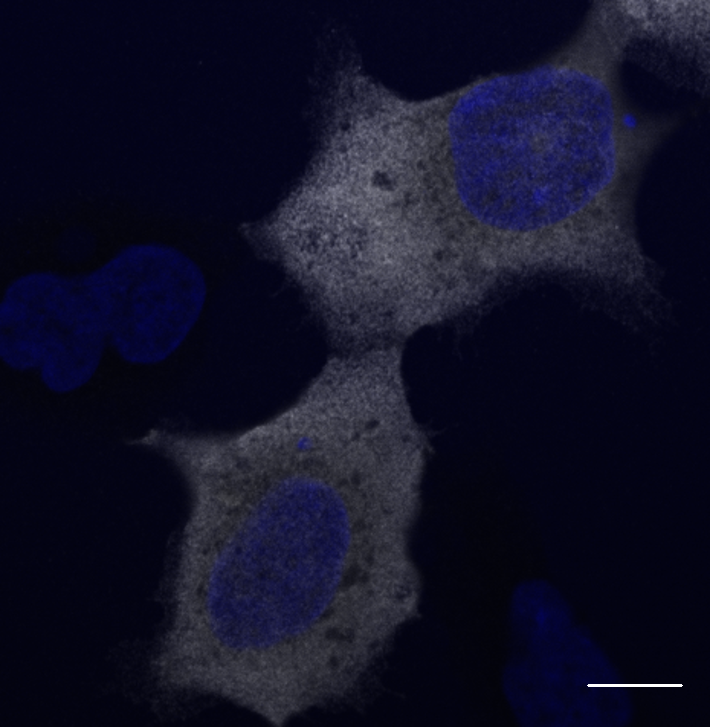

Supplement: Supplementary file 5 — Source data Fig. 3 [file 44319_2024_215_MOESM5_ESM.zip › Figure 3/3C/Fed/Greyscale merge/HA_6D.tif]

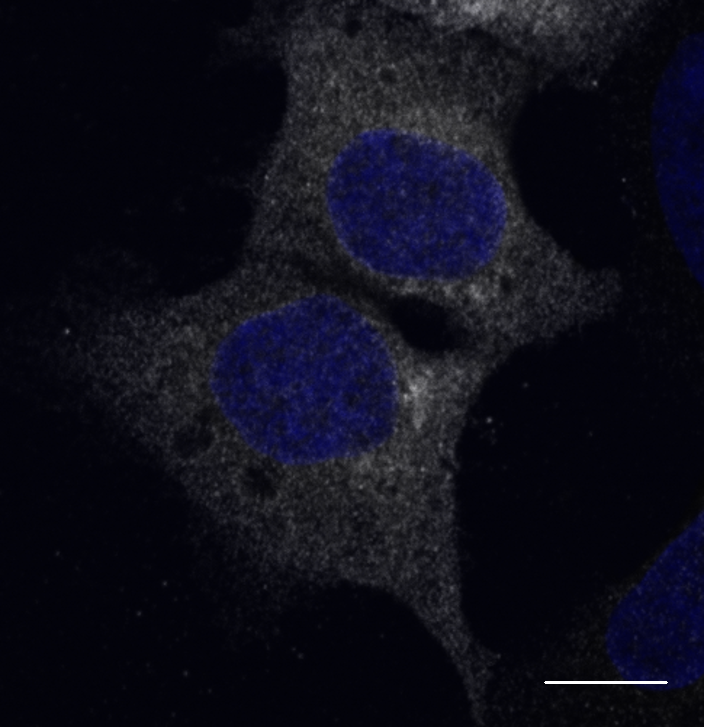

Supplement: Supplementary file 5 — Source data Fig. 3 [file 44319_2024_215_MOESM5_ESM.zip › Figure 3/3C/Fed/Greyscale merge/HA_6A.tif]

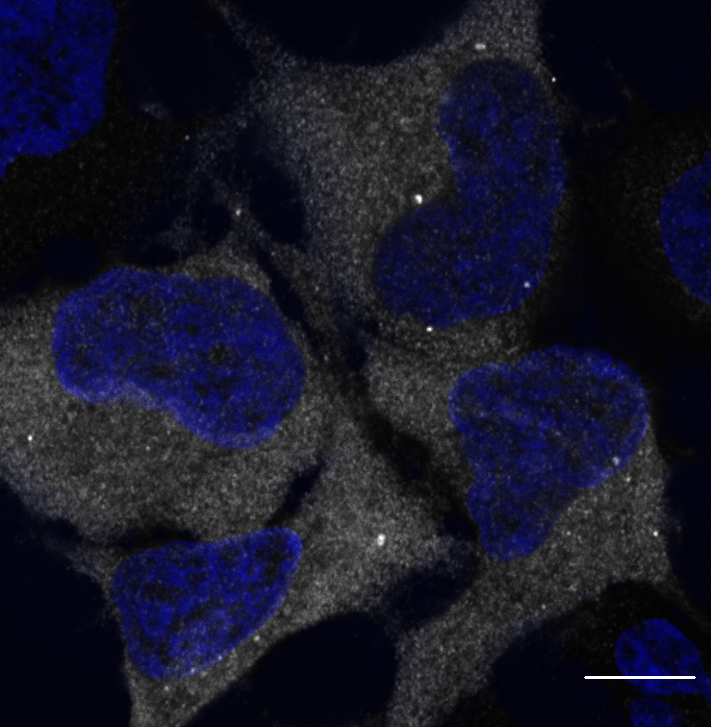

Supplement: Supplementary file 5 — Source data Fig. 3 [file 44319_2024_215_MOESM5_ESM.zip › Figure 3/3C/Starved/Greyscale merge/HA_WT.tif]

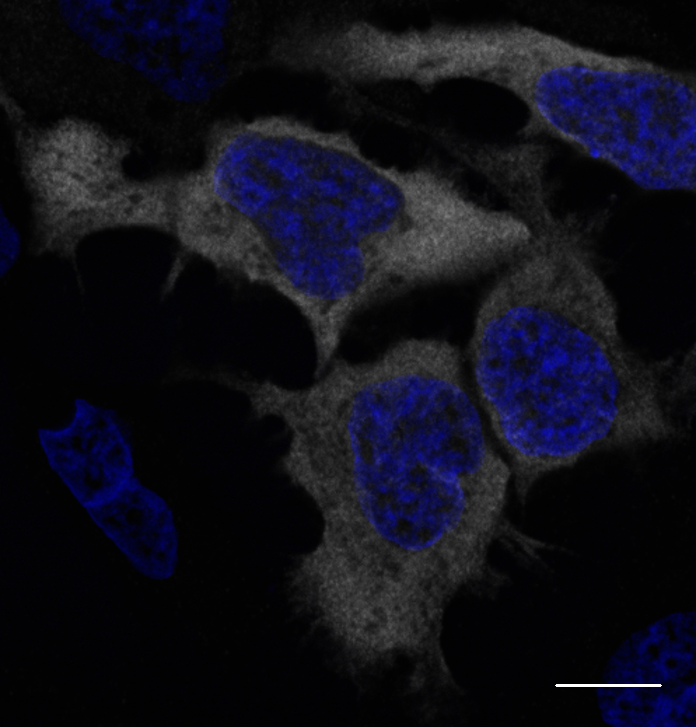

Supplement: Supplementary file 5 — Source data Fig. 3 [file 44319_2024_215_MOESM5_ESM.zip › Figure 3/3C/Starved/Greyscale merge/HA_6D.tif]

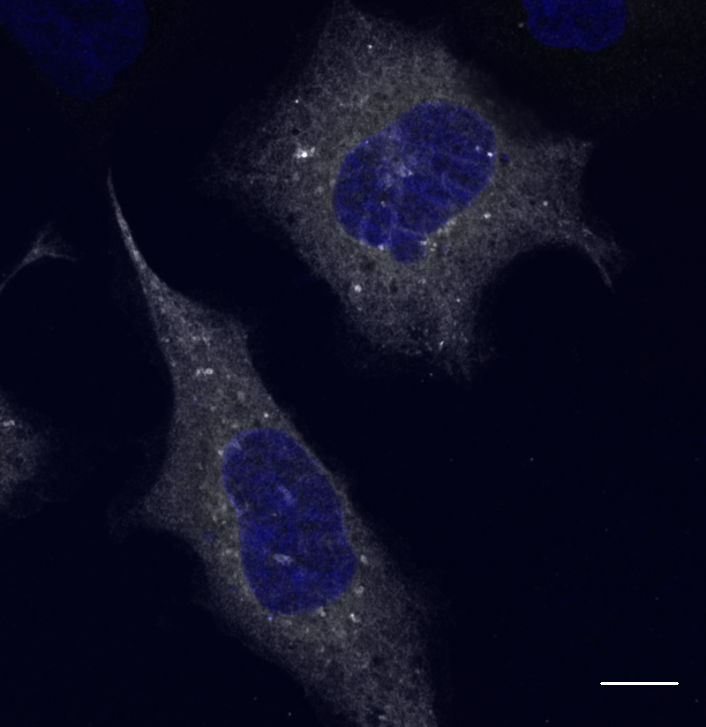

Supplement: Supplementary file 5 — Source data Fig. 3 [file 44319_2024_215_MOESM5_ESM.zip › Figure 3/3C/Starved/Greyscale merge/HA_6A.tif]

# WIP12 CRISPR KO

# WIP12 CRISPR WT

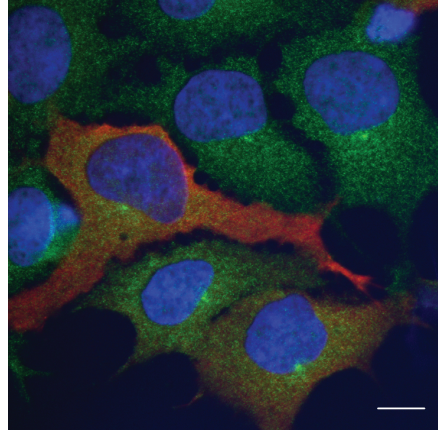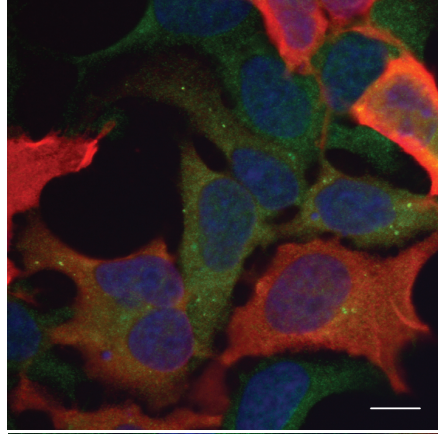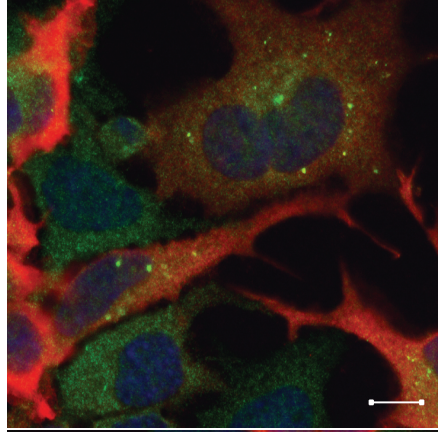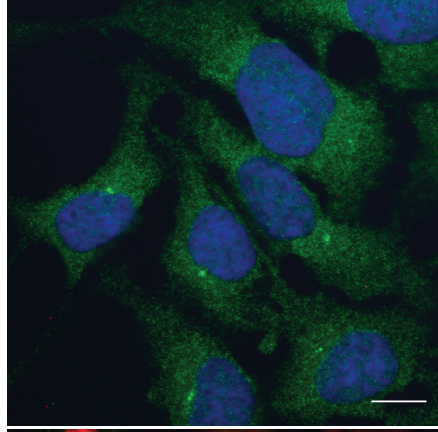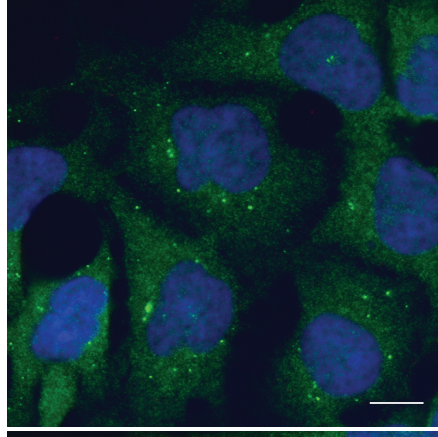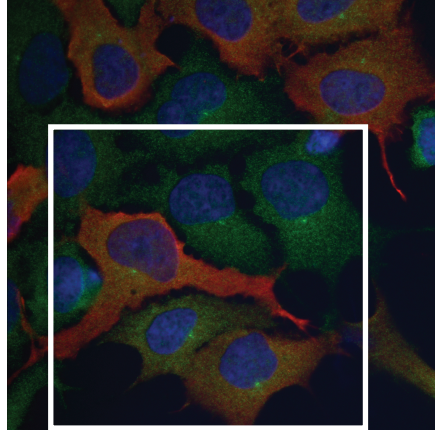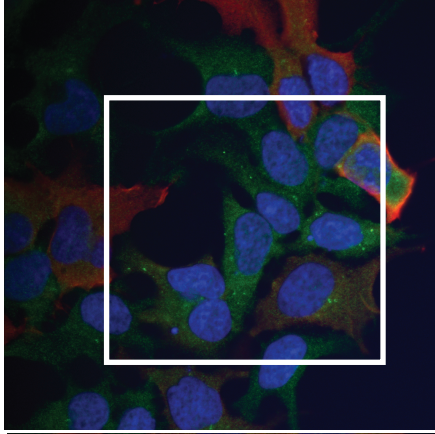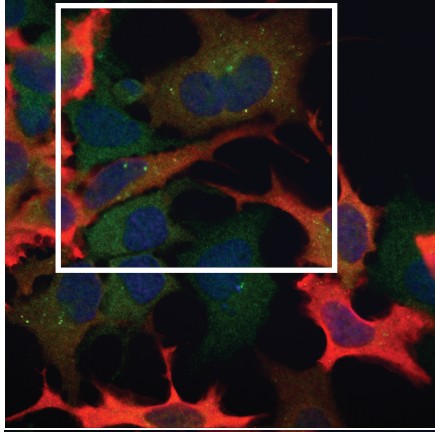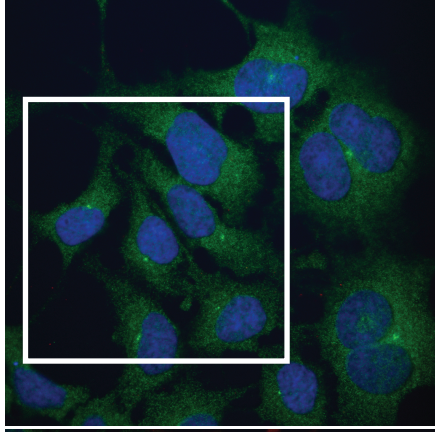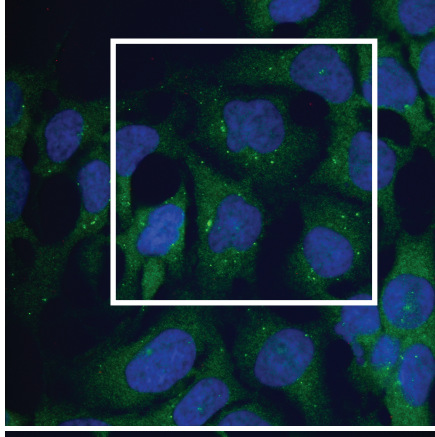

WIP12b S68D

WIP12b S68A

WIP12b WT

pcDNA

pcDNA

Supplement: Supplementary file 6 — Source data Fig. 4 [file 44319_2024_215_MOESM6_ESM.zip › Figure 4/4E/F4E.pdf]

Figure 4B

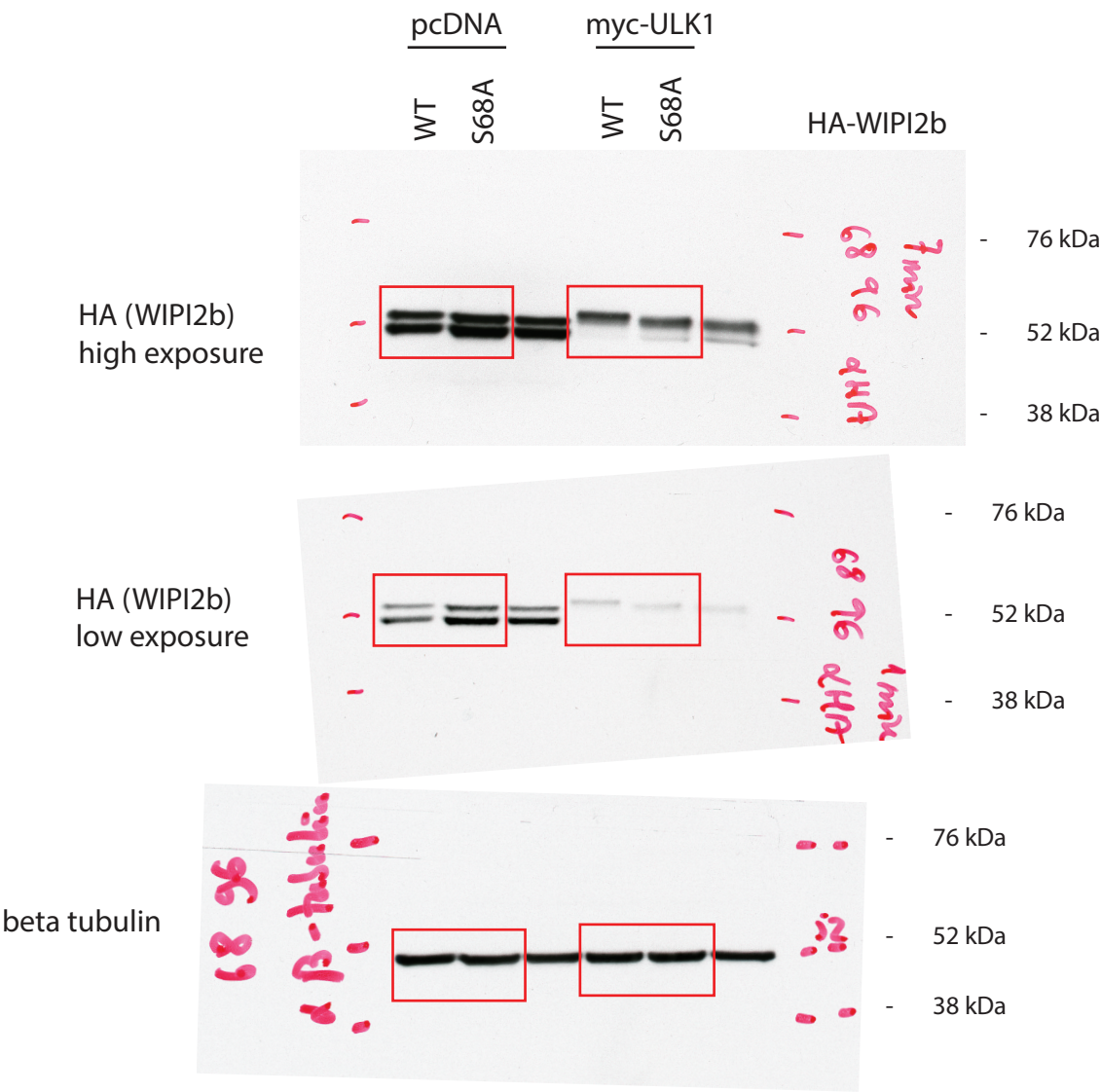

Supplement: Supplementary file 6 — Source data Fig. 4 [file 44319_2024_215_MOESM6_ESM.zip › Figure 4/4B/F4B.pdf]

Figure 4C

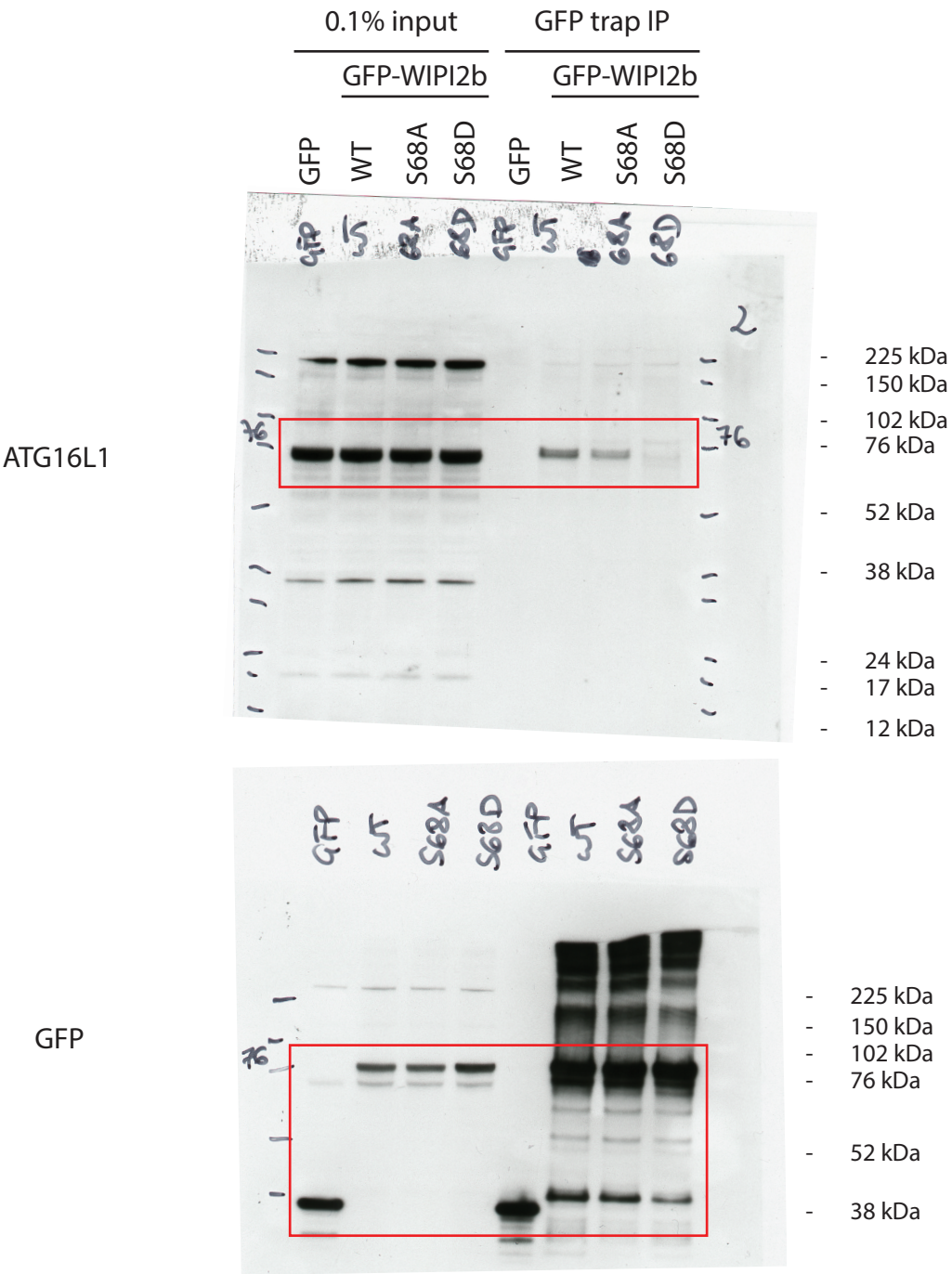

Supplement: Supplementary file 6 — Source data Fig. 4 [file 44319_2024_215_MOESM6_ESM.zip › Figure 4/4C/F4C.pdf]

Figure 4H

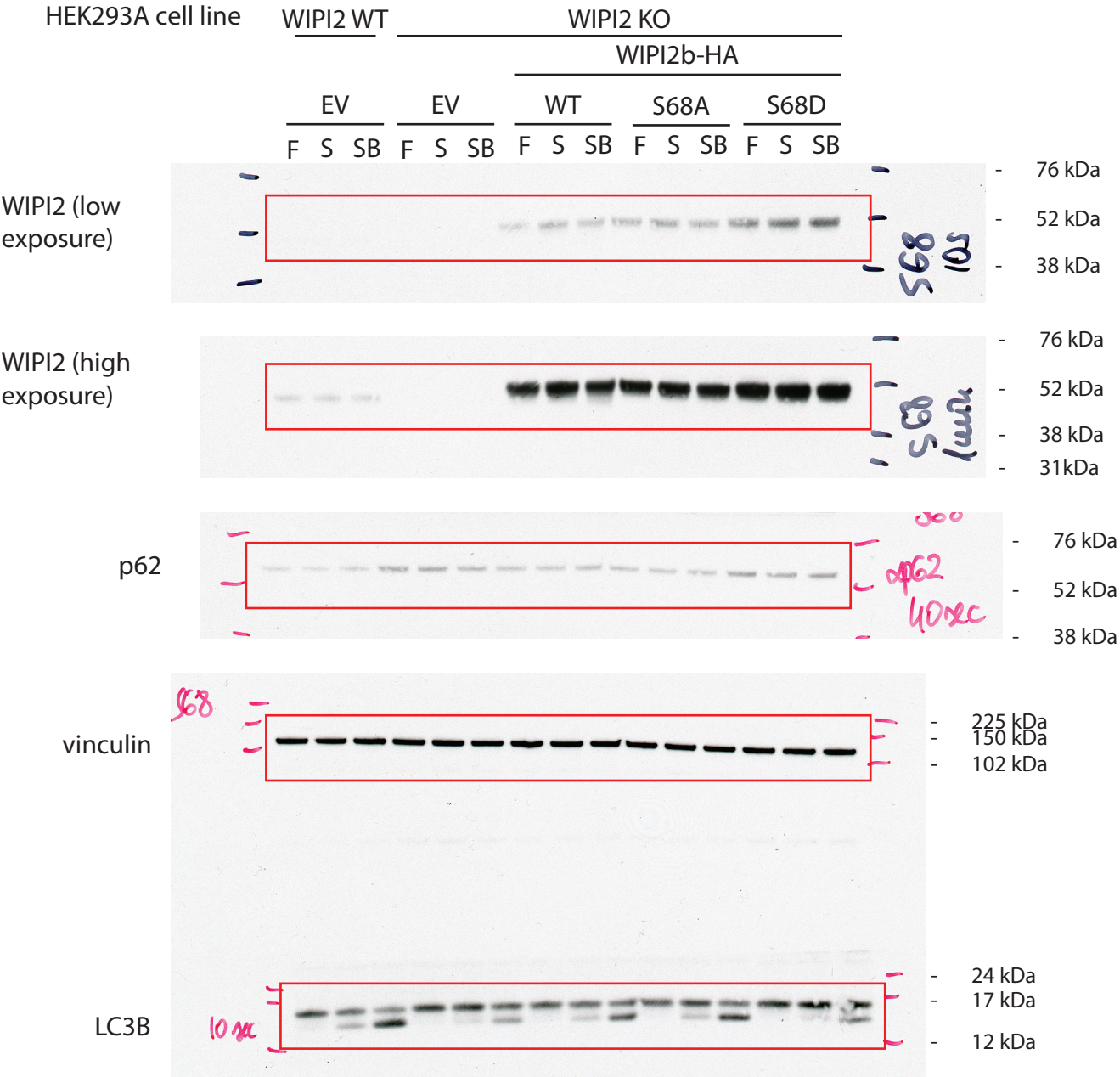

Supplement: Supplementary file 6 — Source data Fig. 4 [file 44319_2024_215_MOESM6_ESM.zip › Figure 4/4H/F4H.pdf]

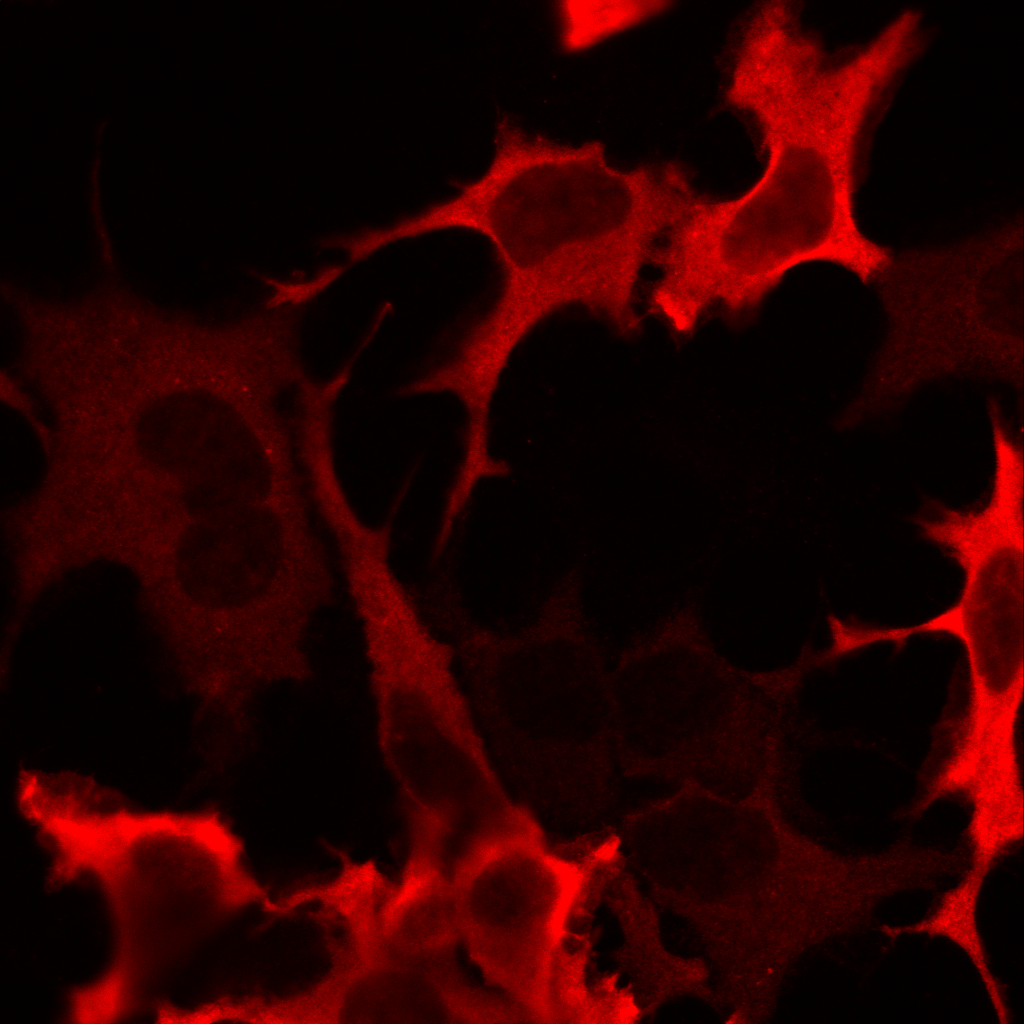

Supplement: Supplementary file 6 — Source data Fig. 4 [file 44319_2024_215_MOESM6_ESM.zip › Figure 4/4E/Images/KO_WT_HA.tif]

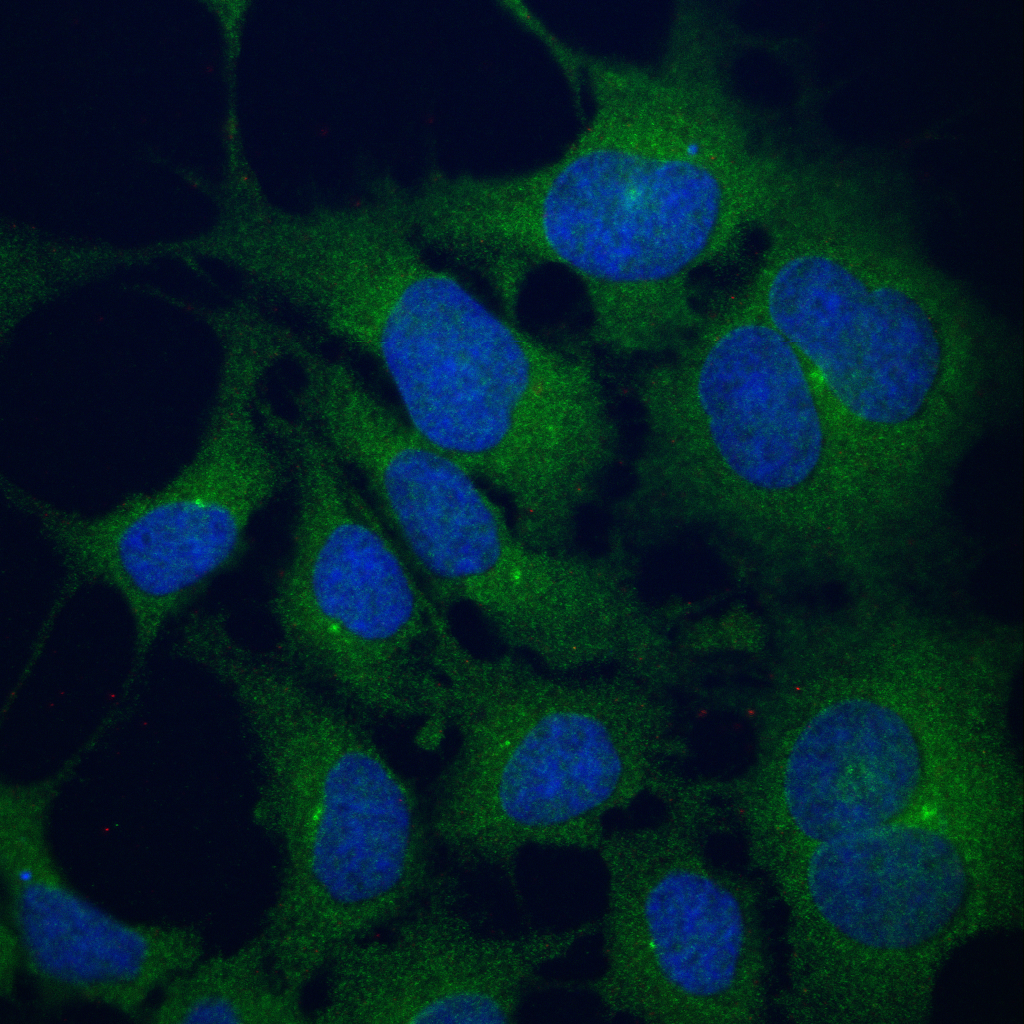

Supplement: Supplementary file 6 — Source data Fig. 4 [file 44319_2024_215_MOESM6_ESM.zip › Figure 4/4E/Images/KO_empty_merge.tif]

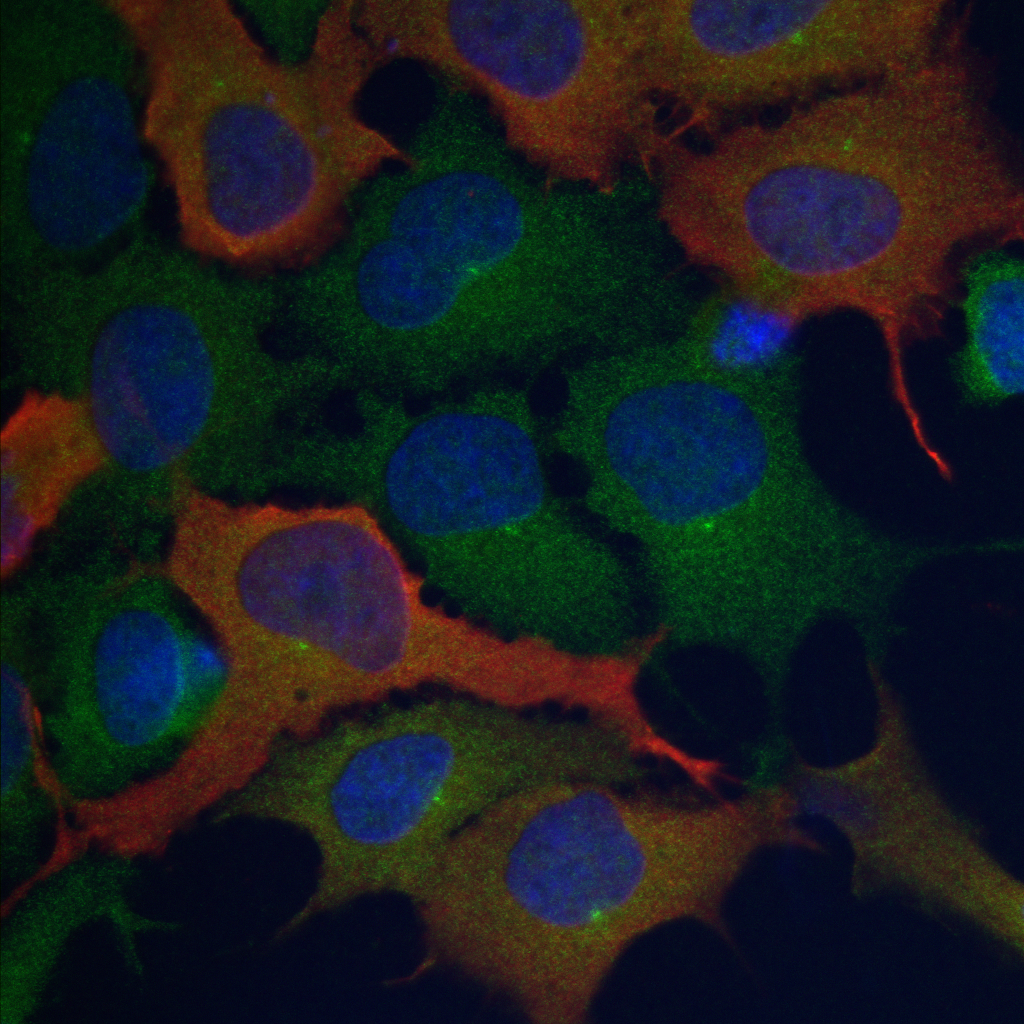

Supplement: Supplementary file 6 — Source data Fig. 4 [file 44319_2024_215_MOESM6_ESM.zip › Figure 4/4E/Images/KO_S68D_merge.tif]

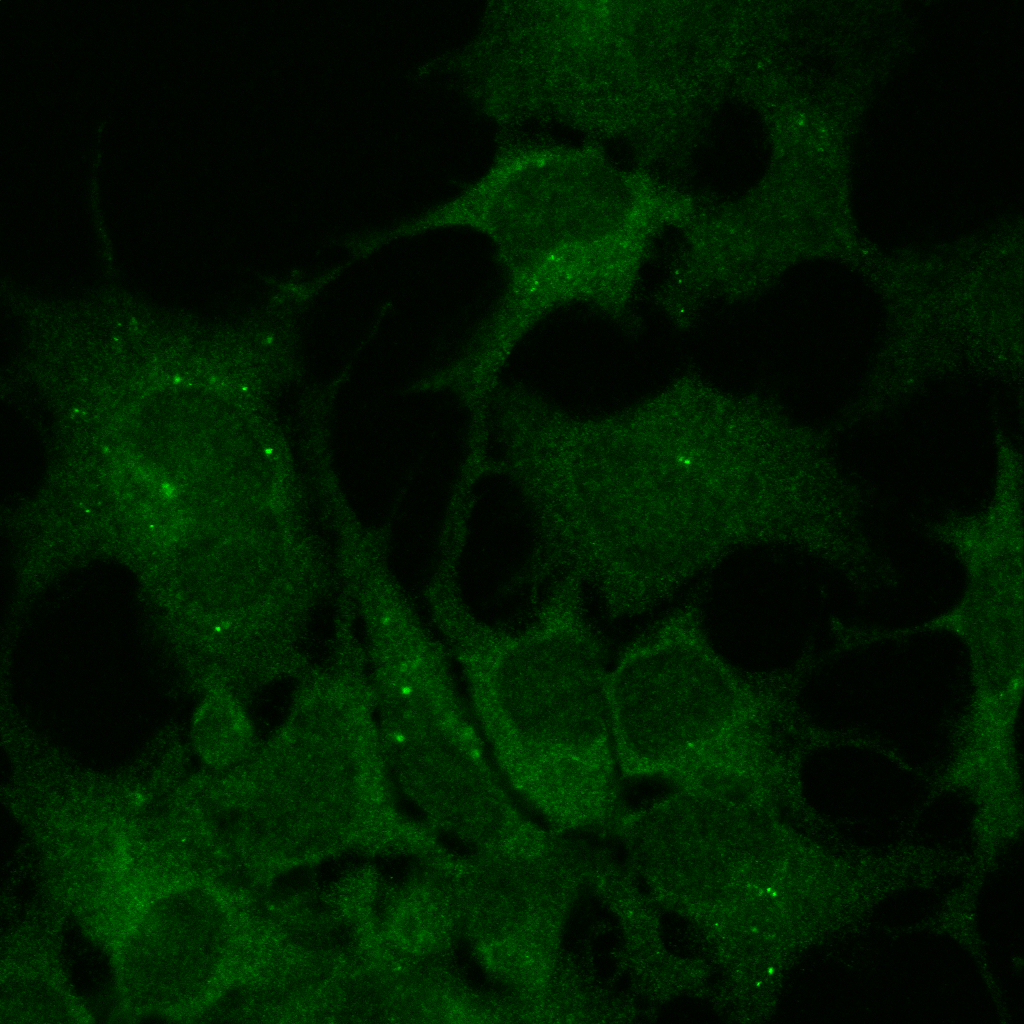

Supplement: Supplementary file 6 — Source data Fig. 4 [file 44319_2024_215_MOESM6_ESM.zip › Figure 4/4E/Images/KO_WT_ATG16L1.tif]

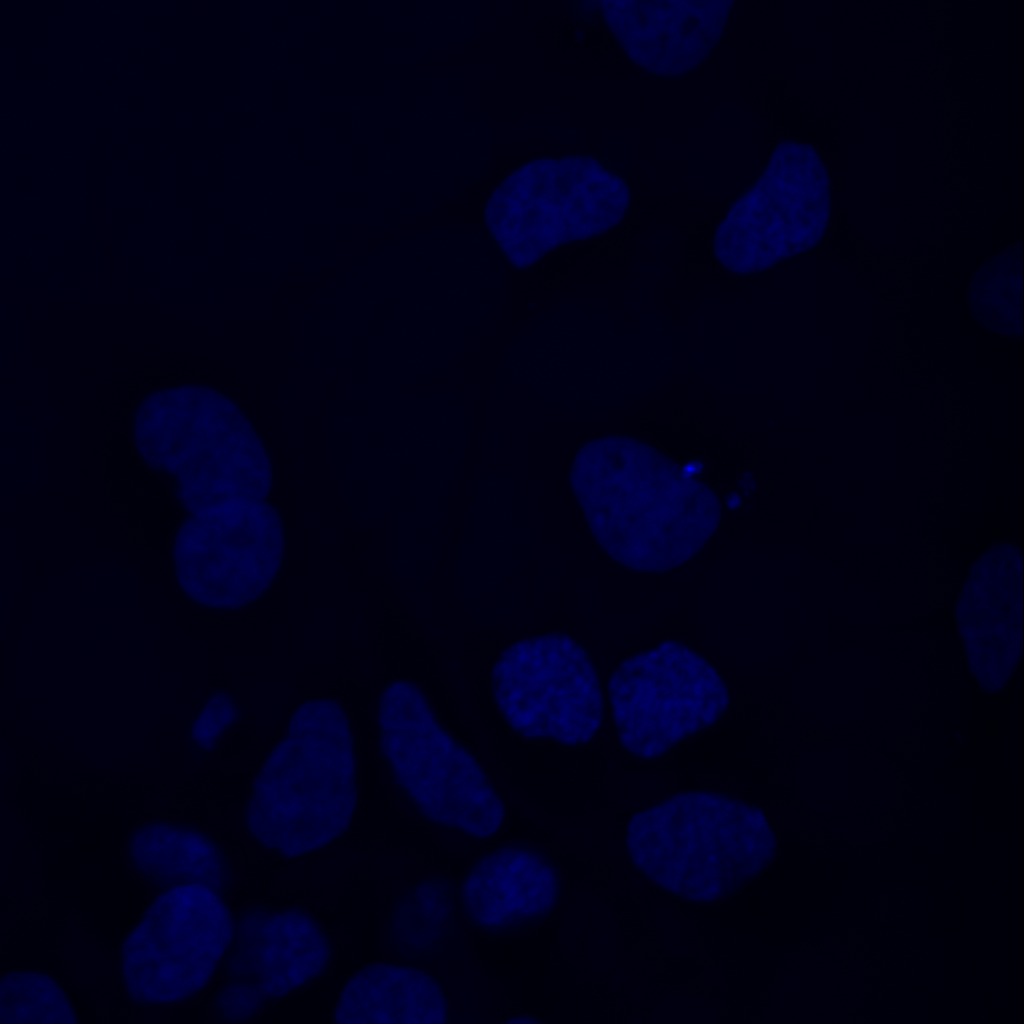

Supplement: Supplementary file 6 — Source data Fig. 4 [file 44319_2024_215_MOESM6_ESM.zip › Figure 4/4E/Images/KO_WT_hoechst.tif]

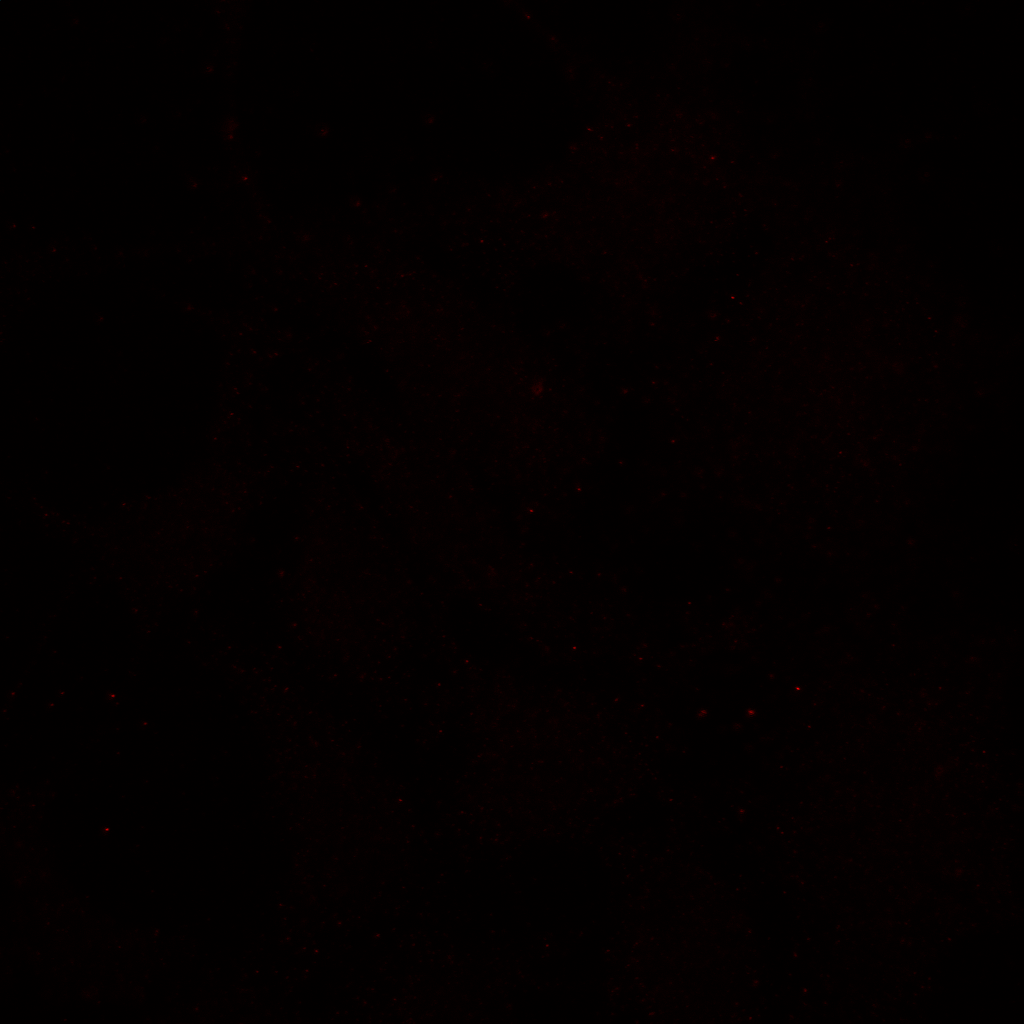

Supplement: Supplementary file 6 — Source data Fig. 4 [file 44319_2024_215_MOESM6_ESM.zip › Figure 4/4E/Images/KO_empty_HA.tif]

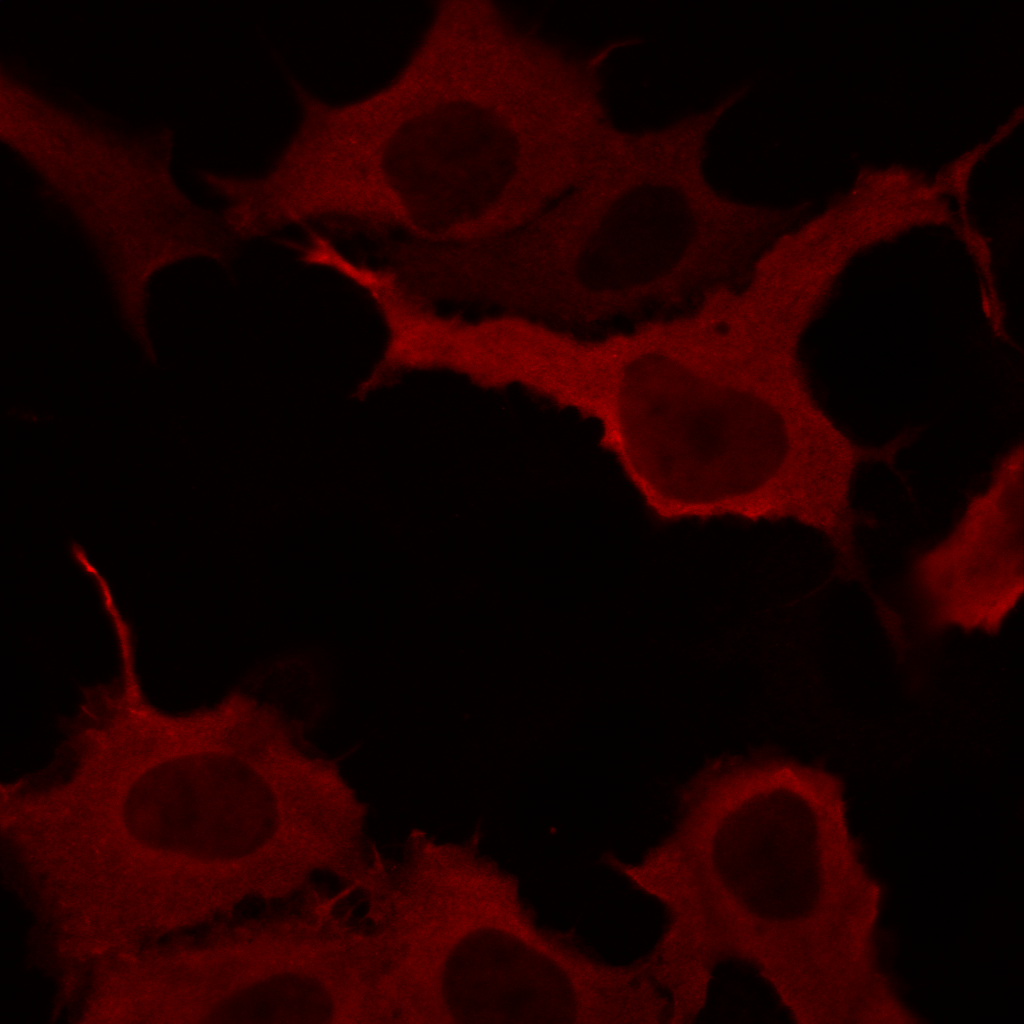

Supplement: Supplementary file 6 — Source data Fig. 4 [file 44319_2024_215_MOESM6_ESM.zip › Figure 4/4E/Images/KO_S68D_HA.tif]

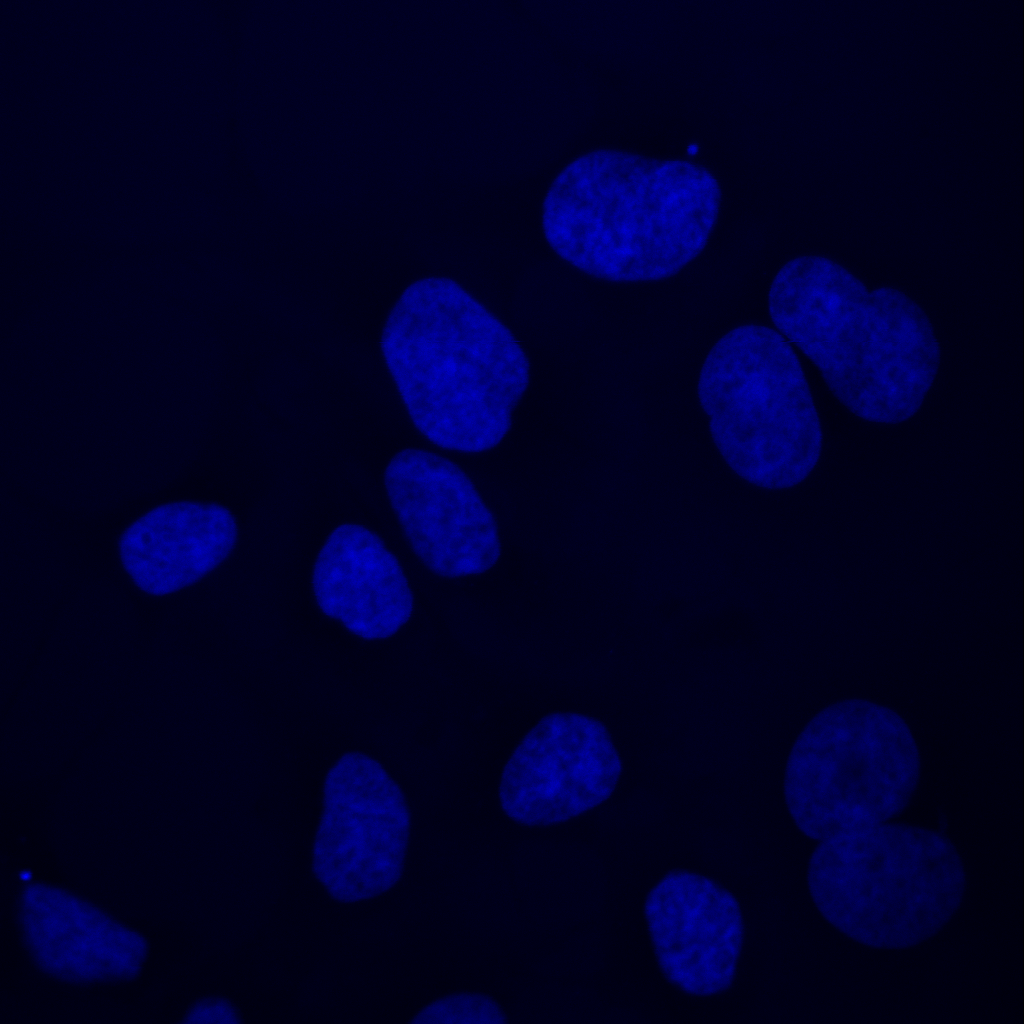

Supplement: Supplementary file 6 — Source data Fig. 4 [file 44319_2024_215_MOESM6_ESM.zip › Figure 4/4E/Images/KO_empty_hoechst.tif]

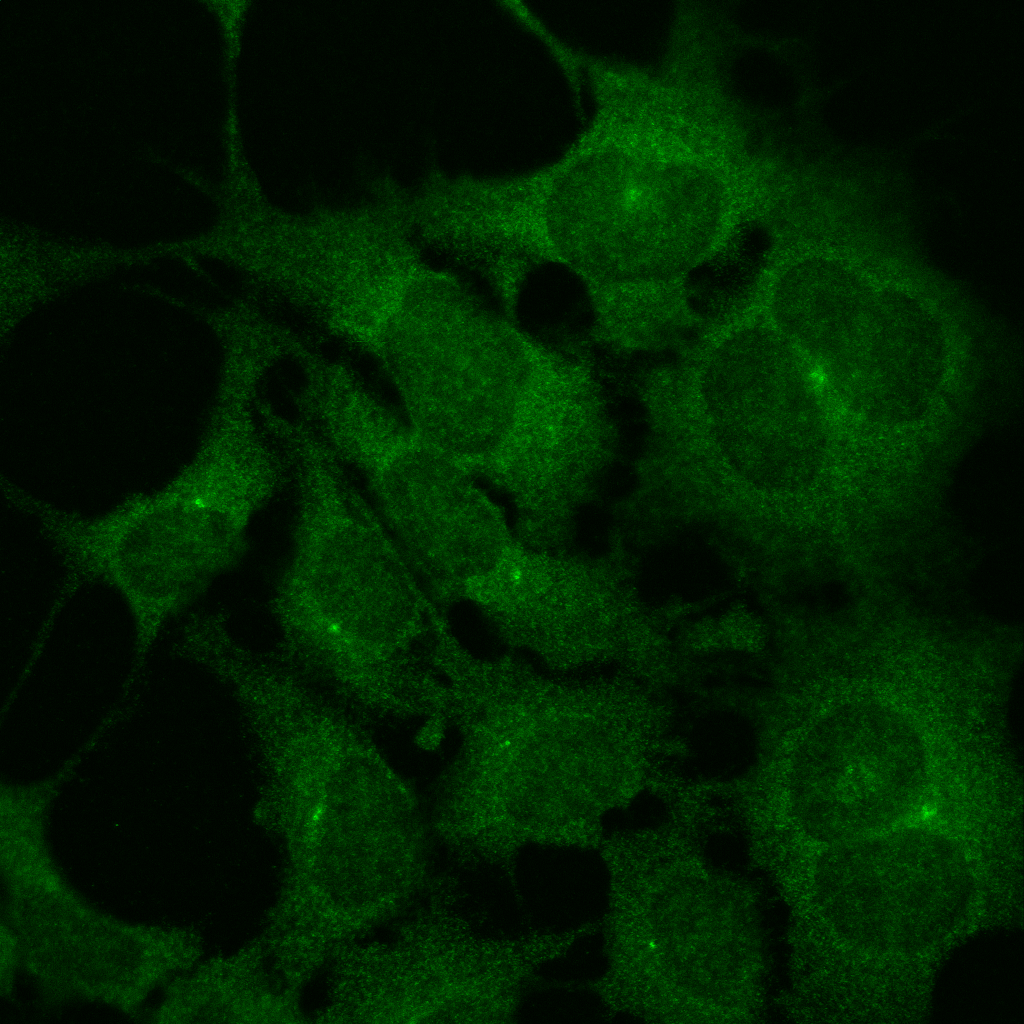

Supplement: Supplementary file 6 — Source data Fig. 4 [file 44319_2024_215_MOESM6_ESM.zip › Figure 4/4E/Images/KO_empty_ATG16L1.tif]

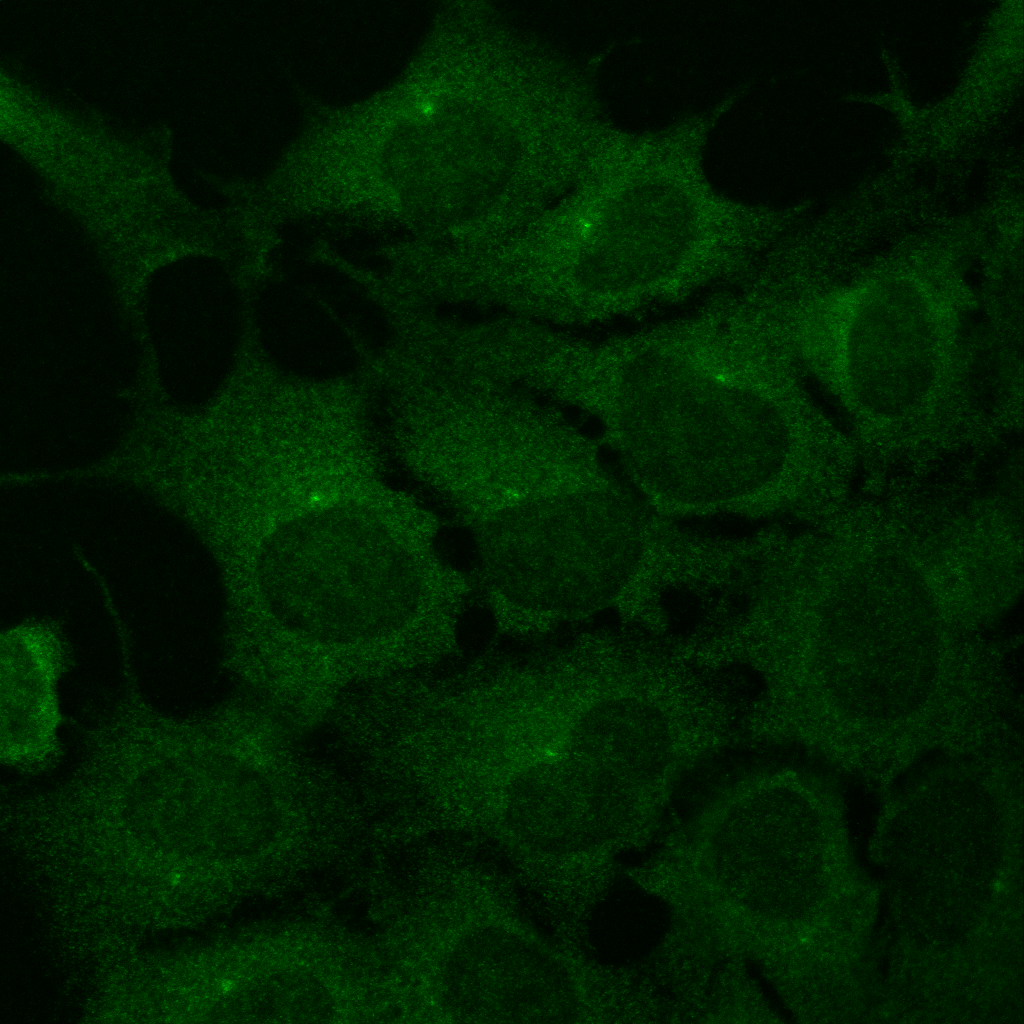

Supplement: Supplementary file 6 — Source data Fig. 4 [file 44319_2024_215_MOESM6_ESM.zip › Figure 4/4E/Images/KO_S68D_ATG16L1.tif]

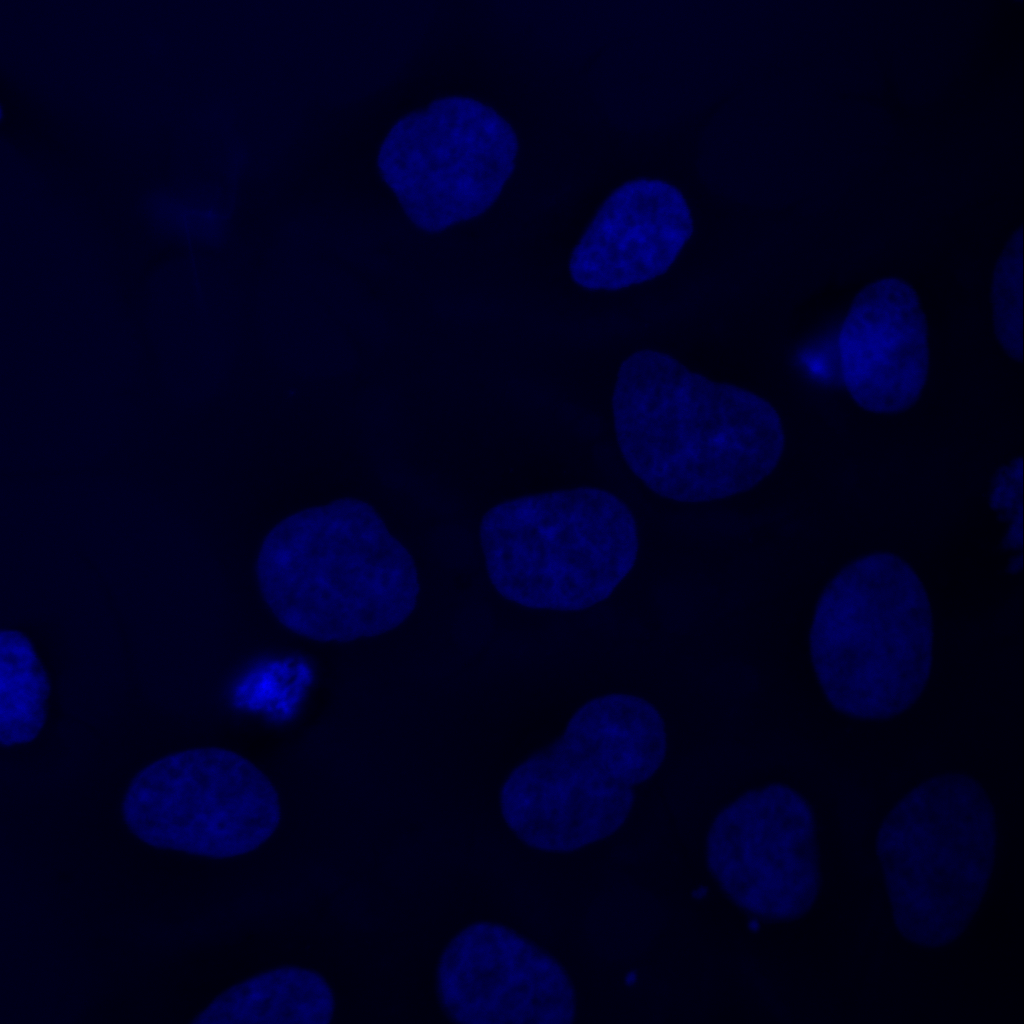

Supplement: Supplementary file 6 — Source data Fig. 4 [file 44319_2024_215_MOESM6_ESM.zip › Figure 4/4E/Images/KO_S68D_hoechst.tif]

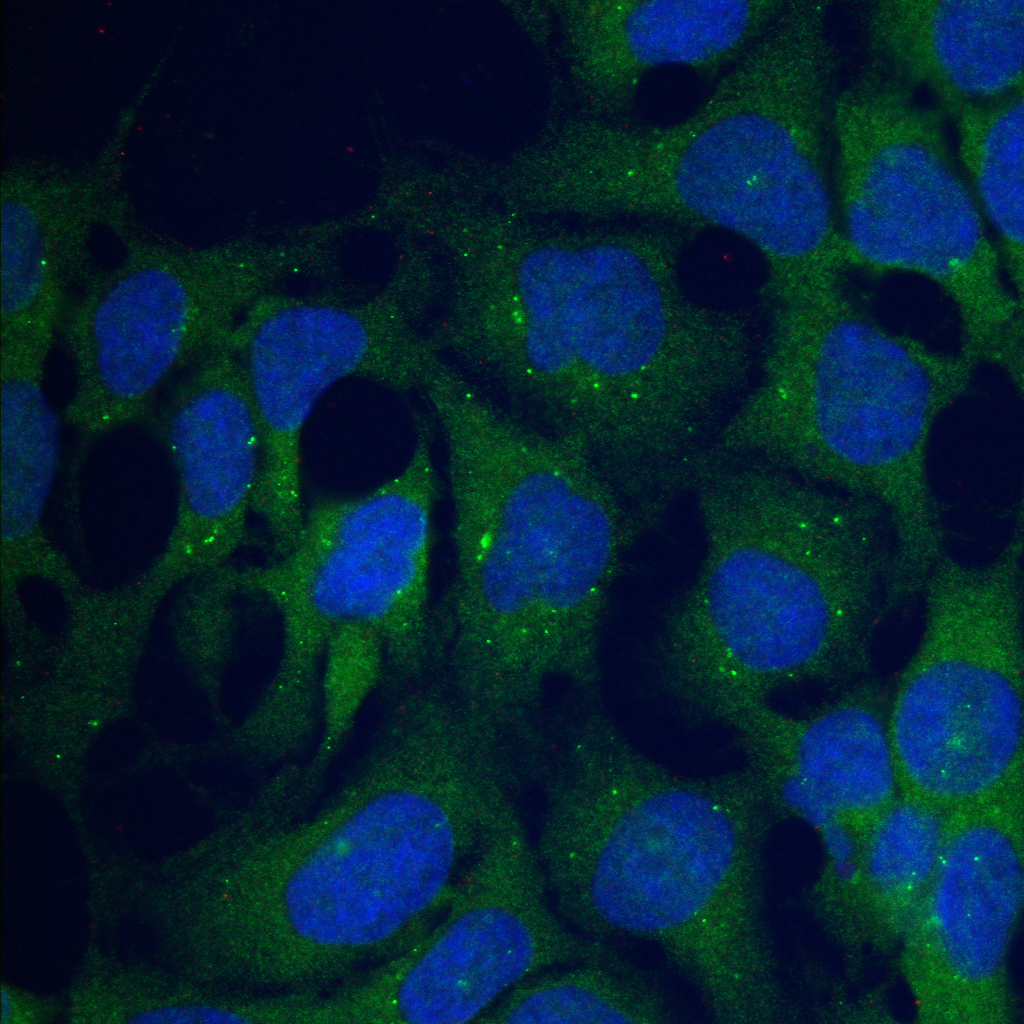

Supplement: Supplementary file 6 — Source data Fig. 4 [file 44319_2024_215_MOESM6_ESM.zip › Figure 4/4E/Images/WT_empty_merge.tif]

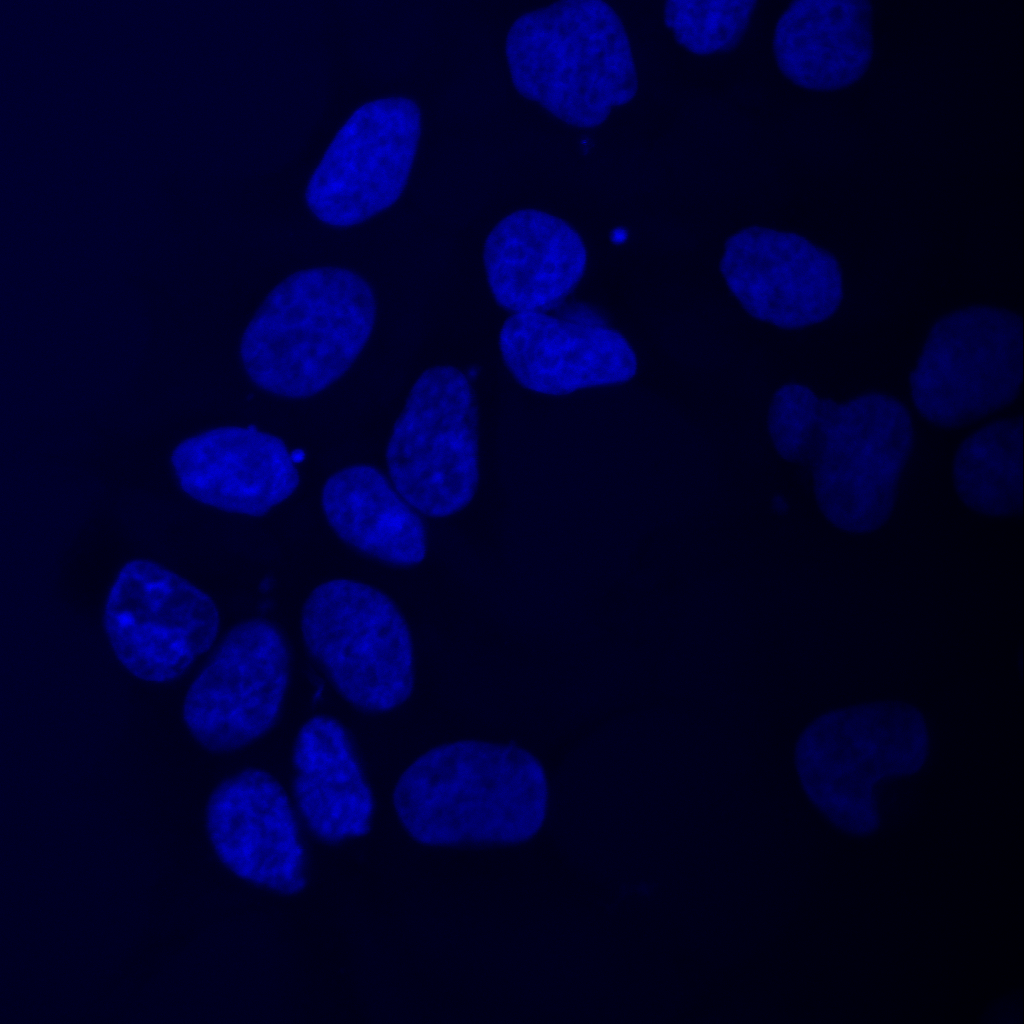

Supplement: Supplementary file 6 — Source data Fig. 4 [file 44319_2024_215_MOESM6_ESM.zip › Figure 4/4E/Images/KO_S68A_hoechst.tif]

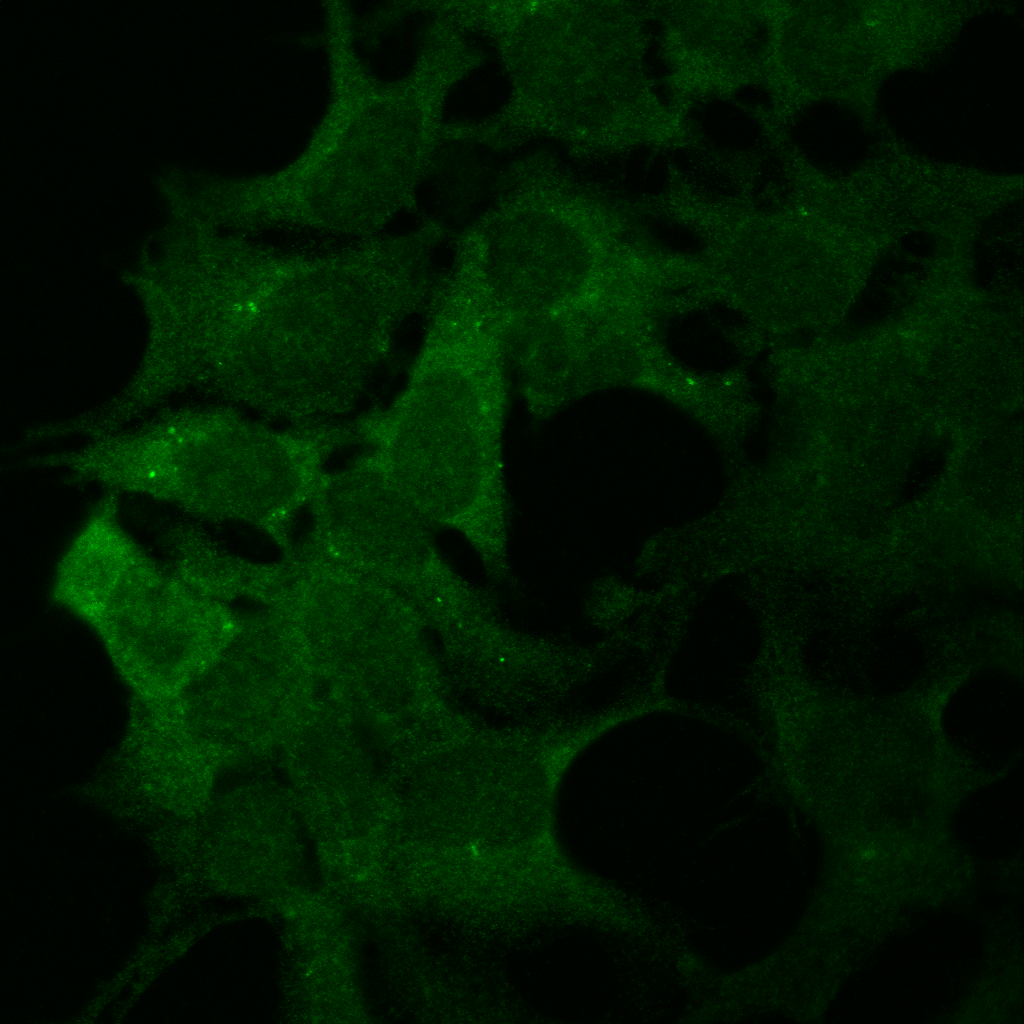

Supplement: Supplementary file 6 — Source data Fig. 4 [file 44319_2024_215_MOESM6_ESM.zip › Figure 4/4E/Images/KO_S68A_ATG16L1.tif]

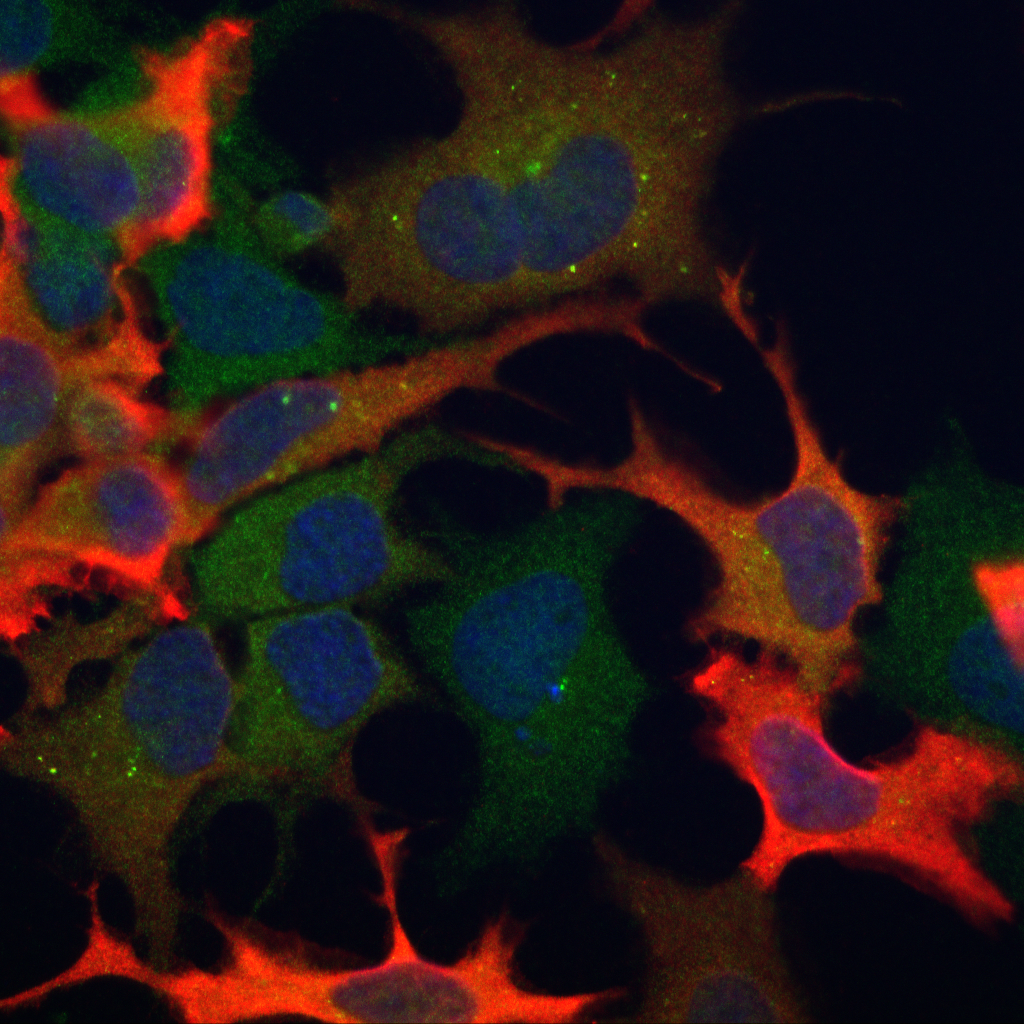

Supplement: Supplementary file 6 — Source data Fig. 4 [file 44319_2024_215_MOESM6_ESM.zip › Figure 4/4E/Images/KO_WT_merge.tif]

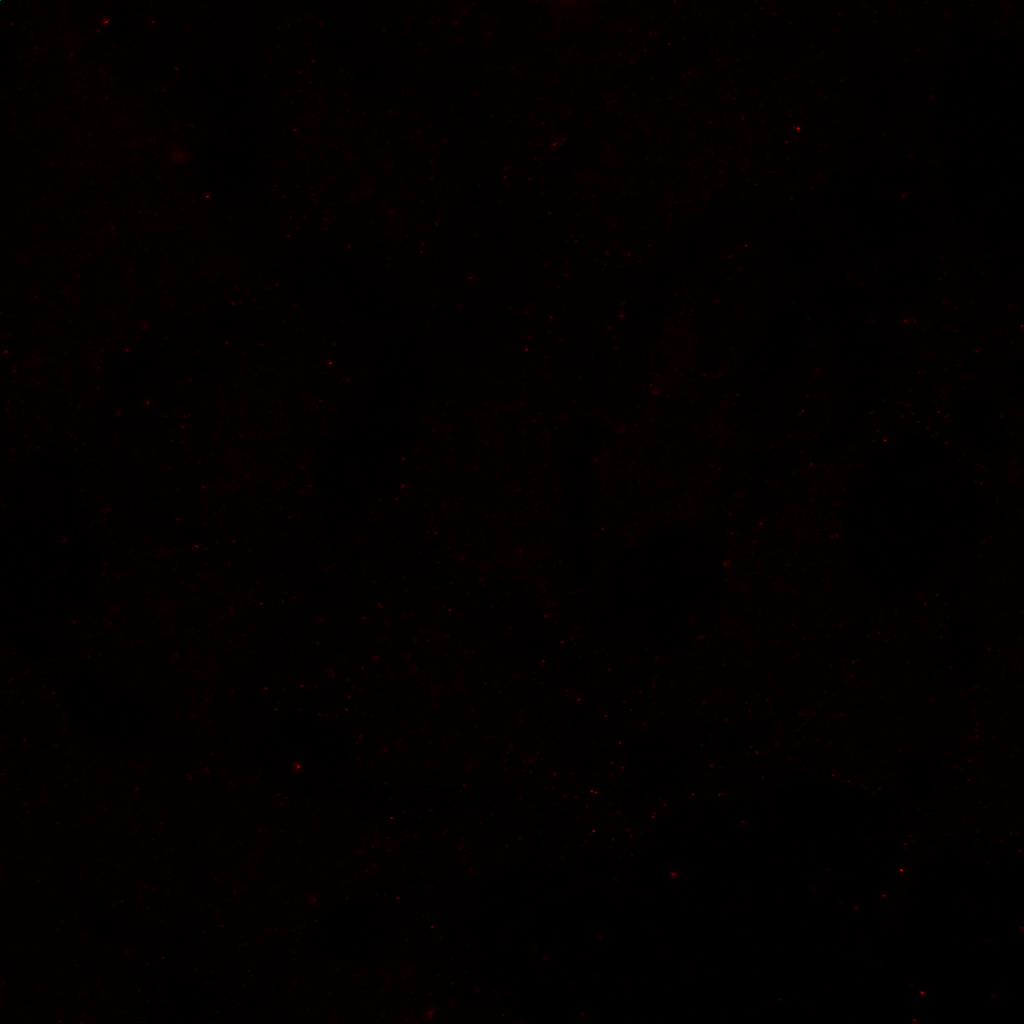

Supplement: Supplementary file 6 — Source data Fig. 4 [file 44319_2024_215_MOESM6_ESM.zip › Figure 4/4E/Images/WT_empty_HA.tif]

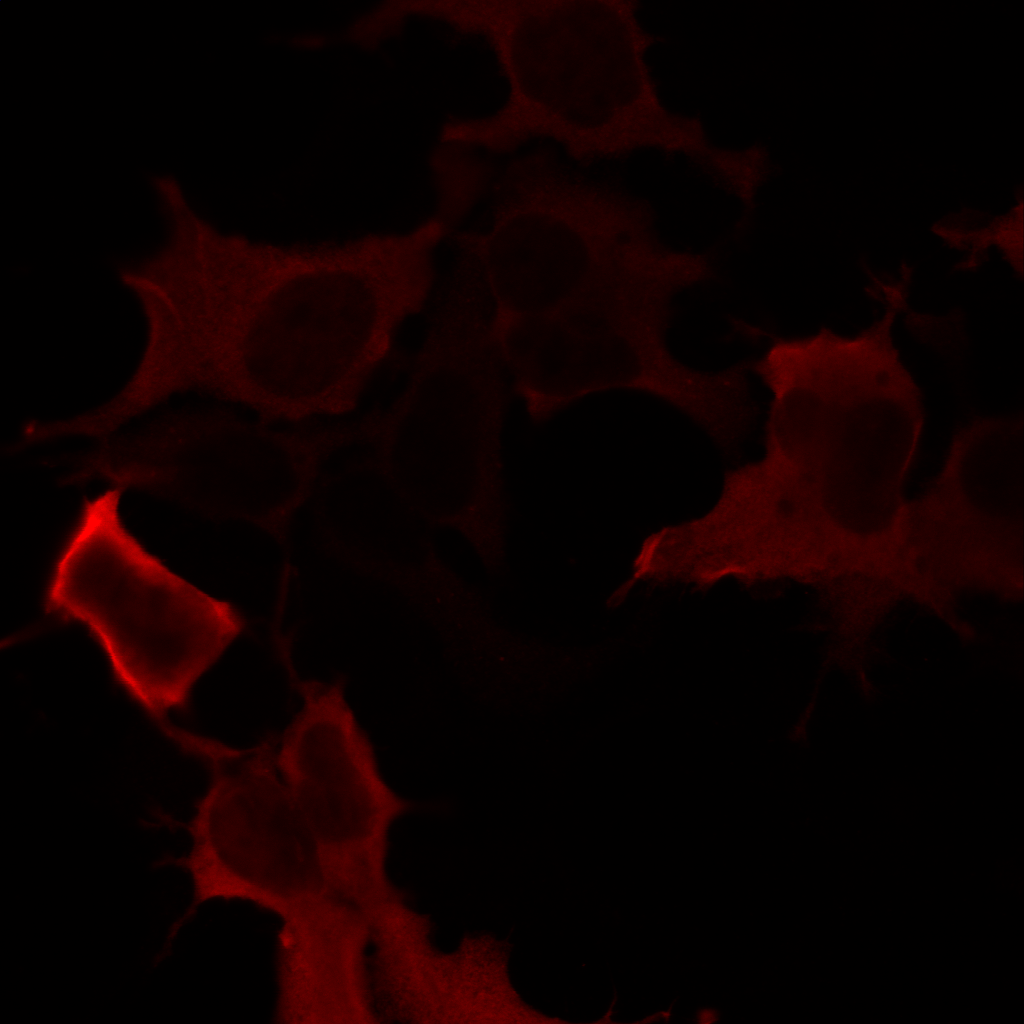

Supplement: Supplementary file 6 — Source data Fig. 4 [file 44319_2024_215_MOESM6_ESM.zip › Figure 4/4E/Images/KO_S68A_HA.tif]

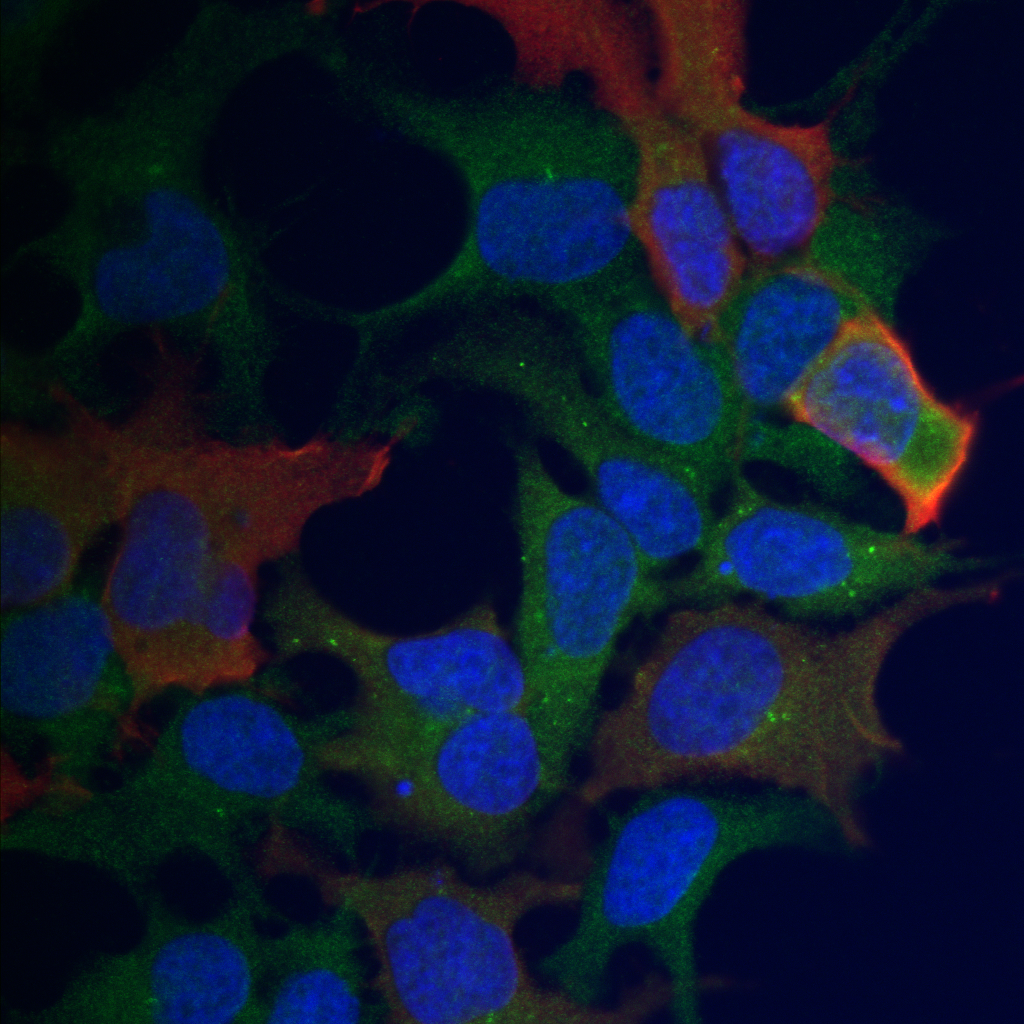

Supplement: Supplementary file 6 — Source data Fig. 4 [file 44319_2024_215_MOESM6_ESM.zip › Figure 4/4E/Images/KO_S68A_merge.tif]

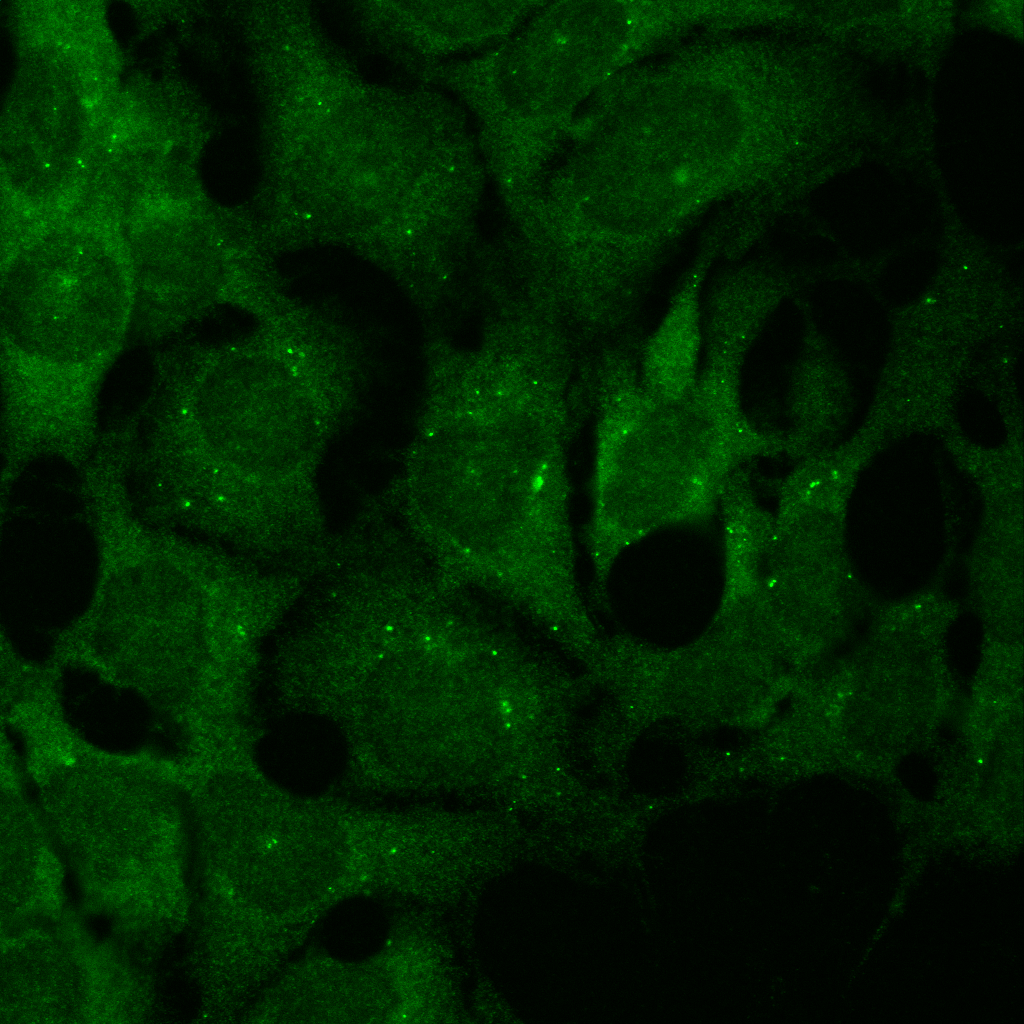

Supplement: Supplementary file 6 — Source data Fig. 4 [file 44319_2024_215_MOESM6_ESM.zip › Figure 4/4E/Images/WT_empty_ATG16L1.tif]

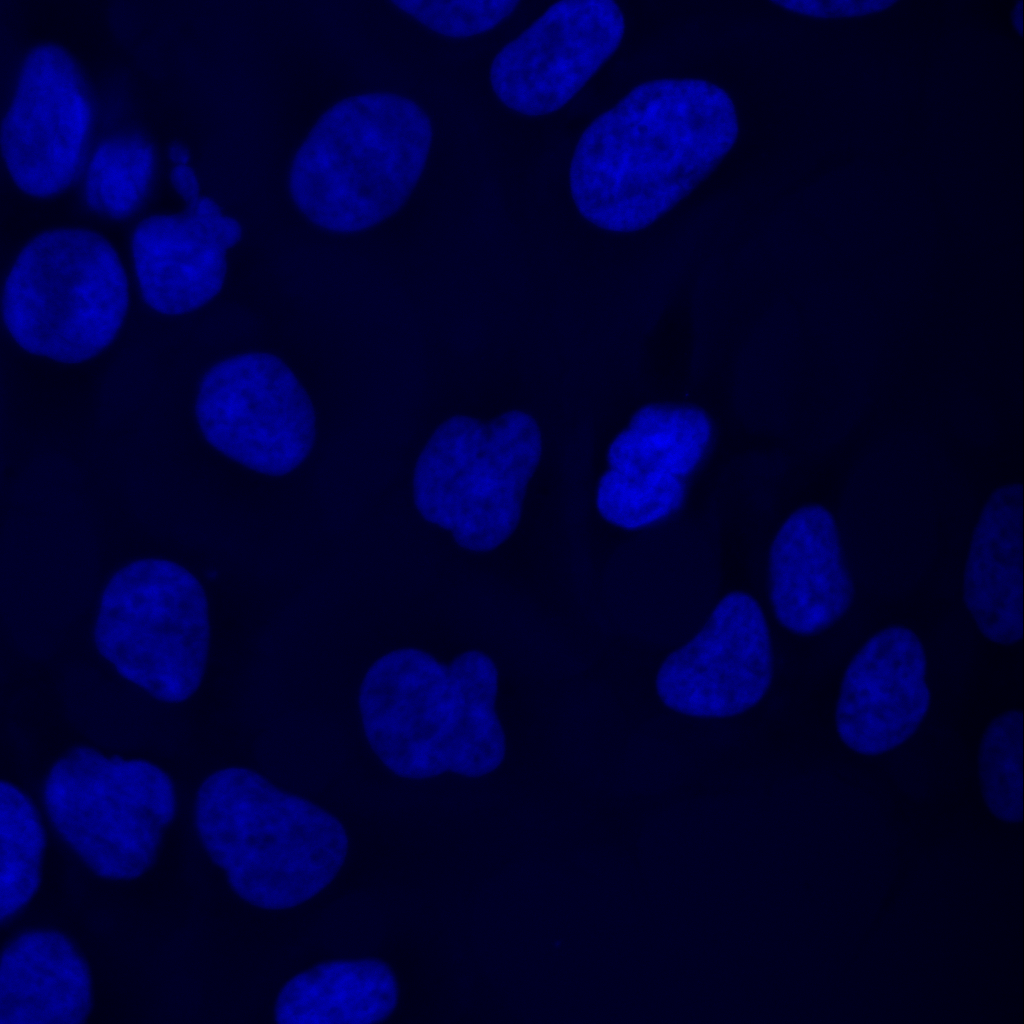

Supplement: Supplementary file 6 — Source data Fig. 4 [file 44319_2024_215_MOESM6_ESM.zip › Figure 4/4E/Images/WT_empty_hoechst.tif]

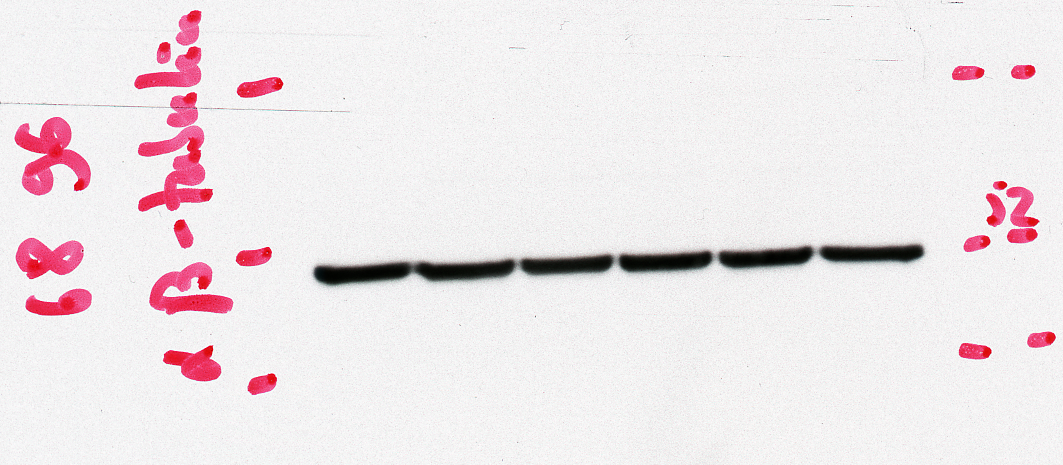

Supplement: Supplementary file 6 — Source data Fig. 4 [file 44319_2024_215_MOESM6_ESM.zip › Figure 4/4B/Images/western tubulin.tif]

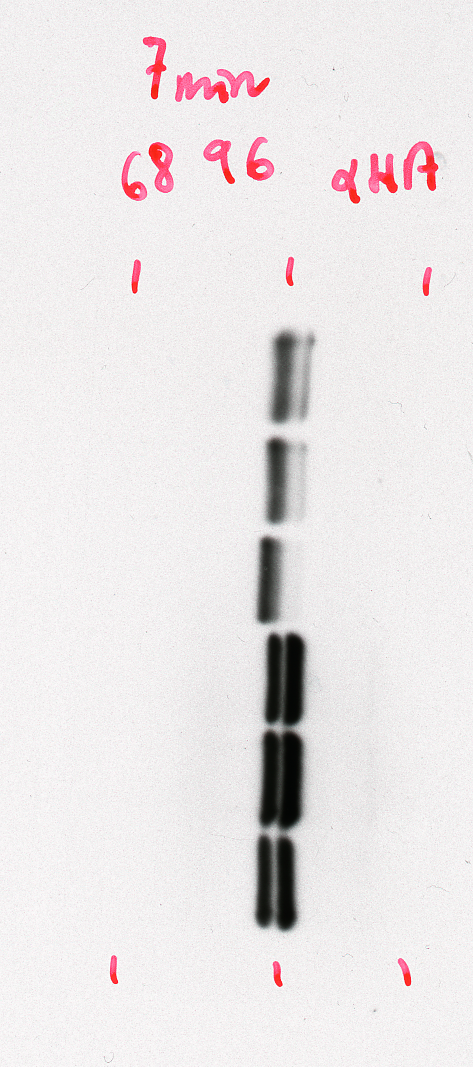

Supplement: Supplementary file 6 — Source data Fig. 4 [file 44319_2024_215_MOESM6_ESM.zip › Figure 4/4B/Images/western HA high.tif]

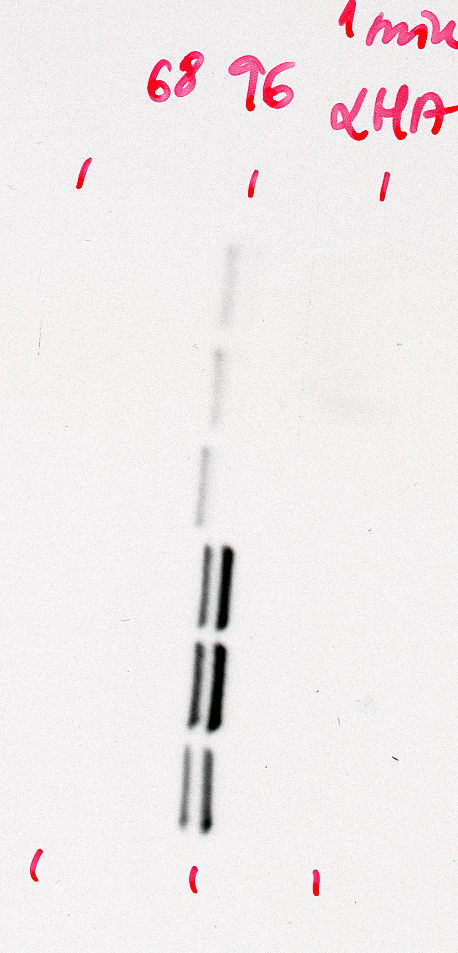

Supplement: Supplementary file 6 — Source data Fig. 4 [file 44319_2024_215_MOESM6_ESM.zip › Figure 4/4B/Images/western HA low.tif]

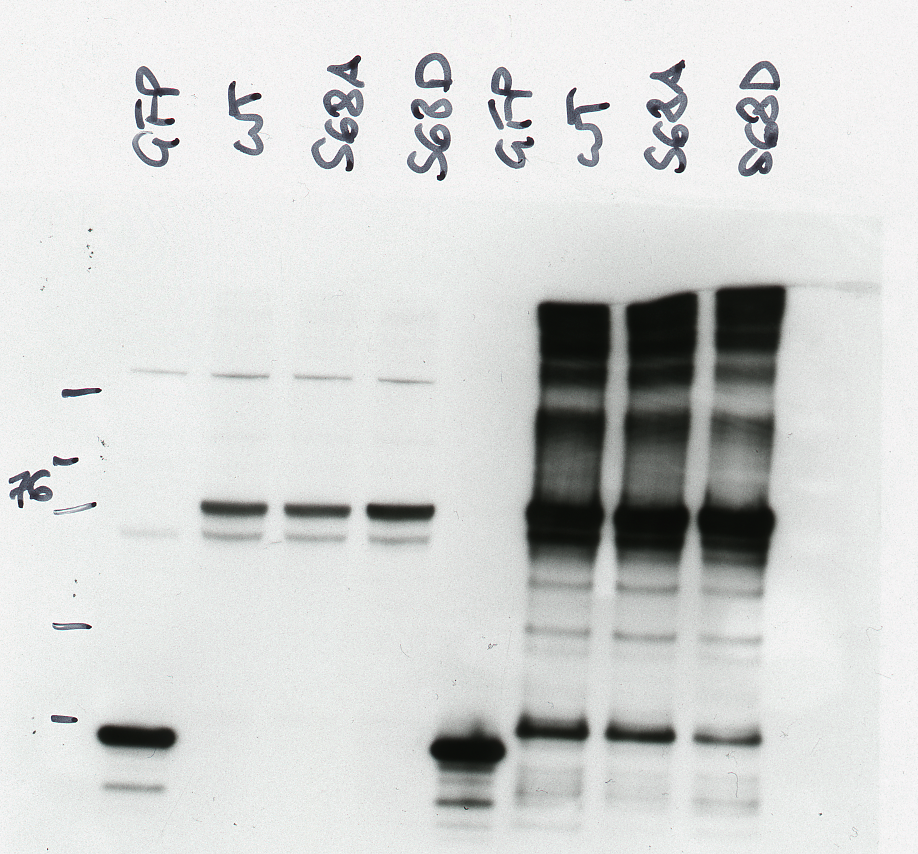

Supplement: Supplementary file 6 — Source data Fig. 4 [file 44319_2024_215_MOESM6_ESM.zip › Figure 4/4C/Images/western GFP.tif]

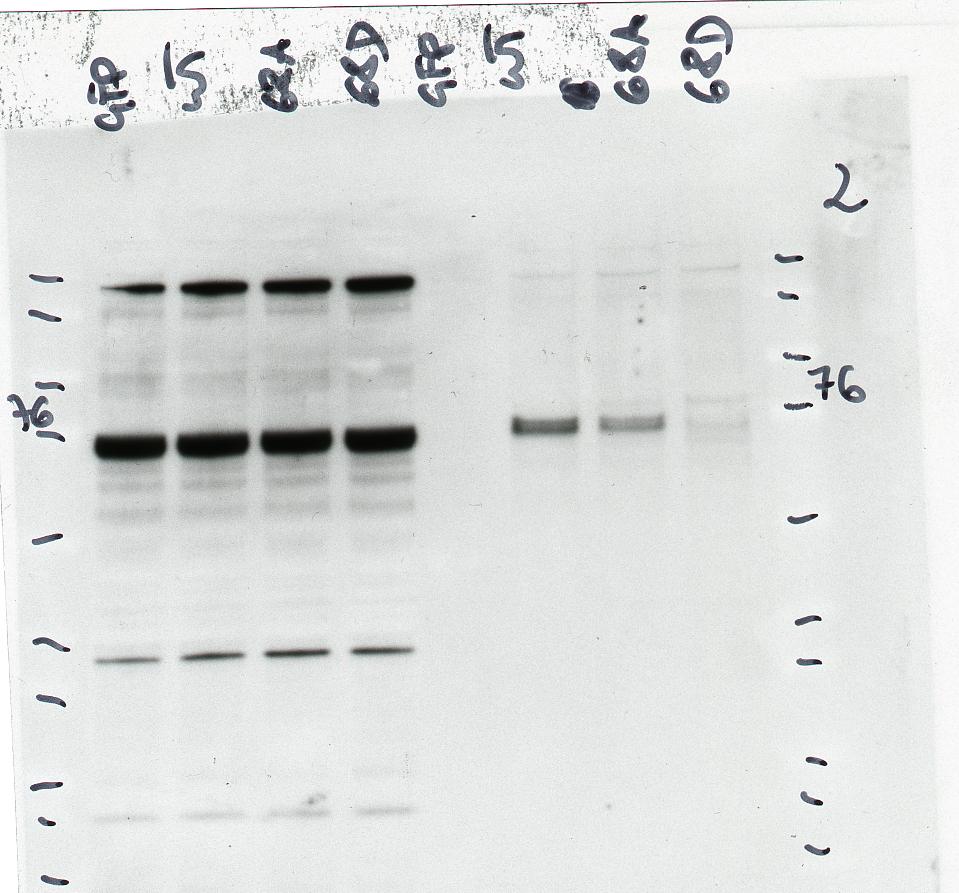

Supplement: Supplementary file 6 — Source data Fig. 4 [file 44319_2024_215_MOESM6_ESM.zip › Figure 4/4C/Images/western ATG16L1.tif]

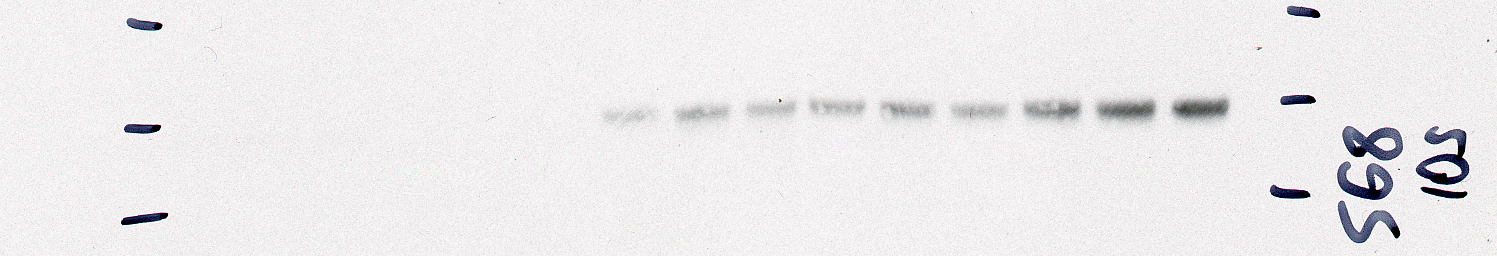

Supplement: Supplementary file 6 — Source data Fig. 4 [file 44319_2024_215_MOESM6_ESM.zip › Figure 4/4H/Images/western WIPI2 low.tif]

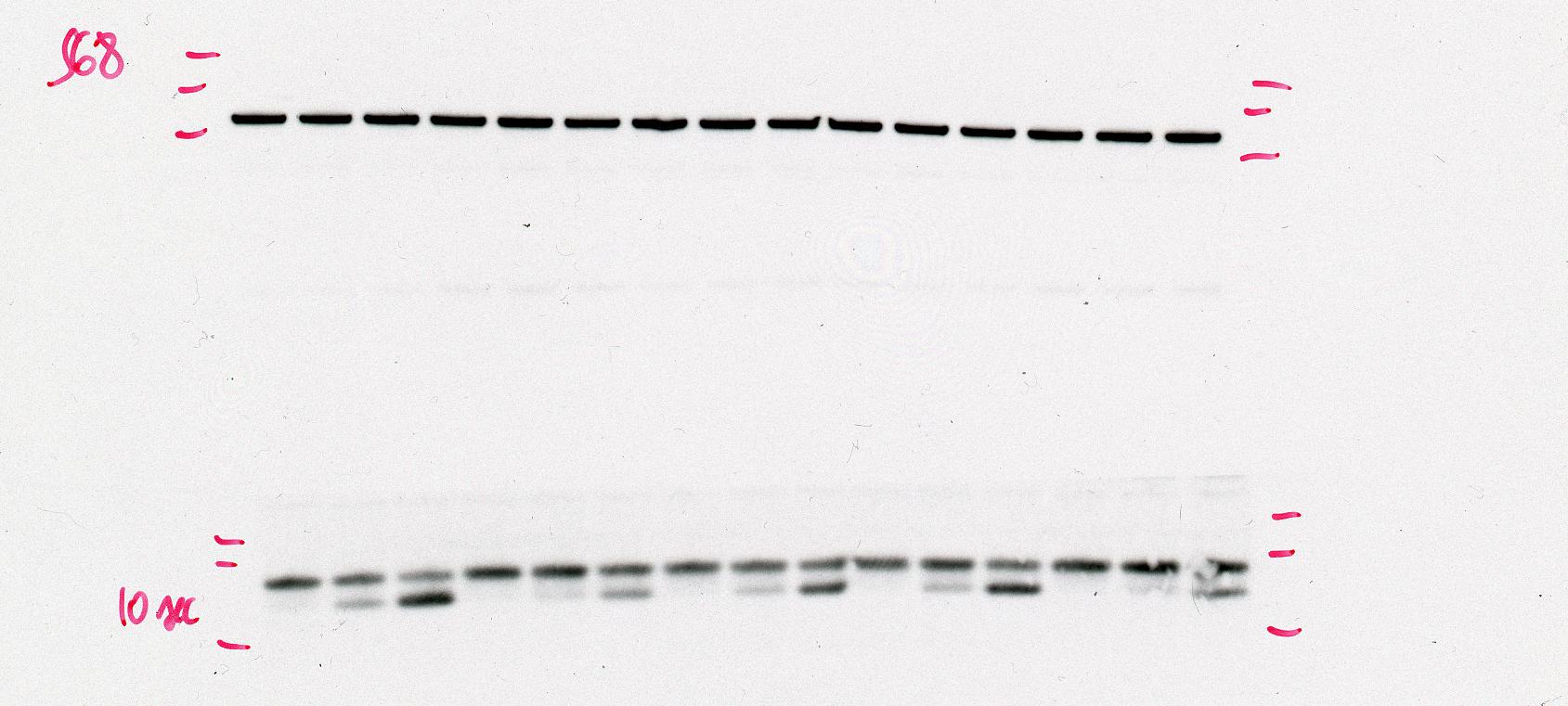

Supplement: Supplementary file 6 — Source data Fig. 4 [file 44319_2024_215_MOESM6_ESM.zip › Figure 4/4H/Images/western vinculin_LC3.tif]

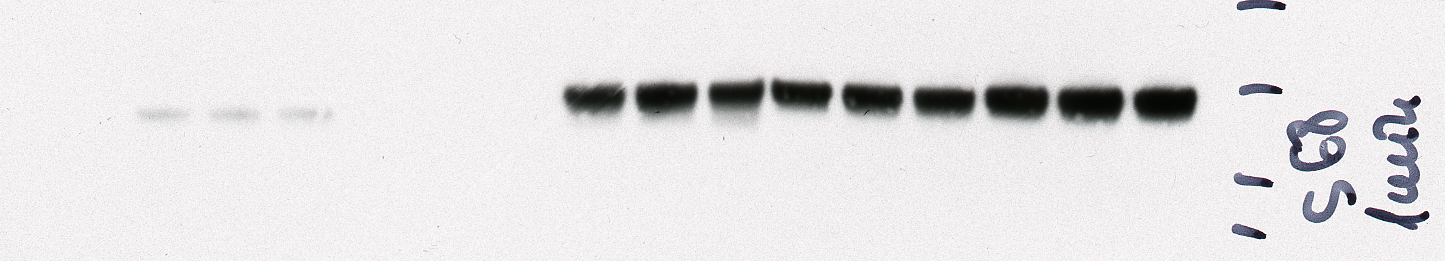

Supplement: Supplementary file 6 — Source data Fig. 4 [file 44319_2024_215_MOESM6_ESM.zip › Figure 4/4H/Images/western WIPI2.tif]

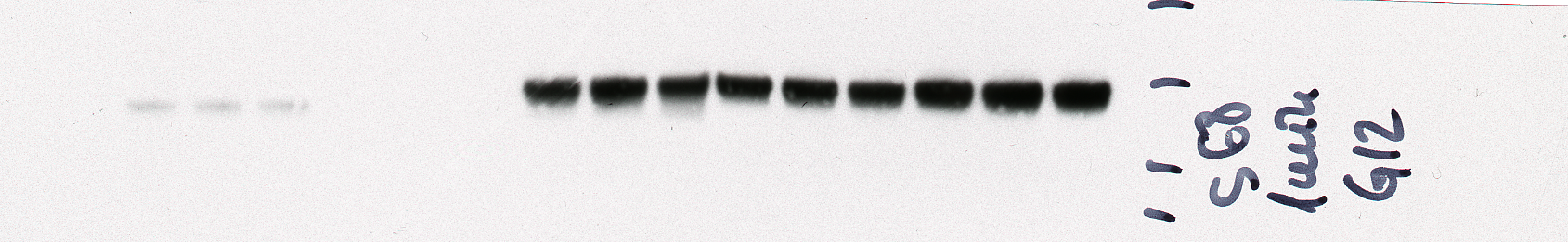

Supplement: Supplementary file 6 — Source data Fig. 4 [file 44319_2024_215_MOESM6_ESM.zip › Figure 4/4H/Images/western WIPI2 high.tif]

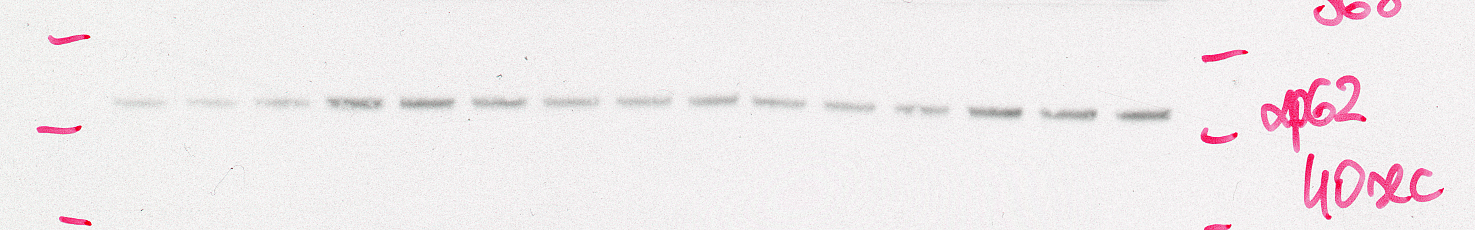

Supplement: Supplementary file 6 — Source data Fig. 4 [file 44319_2024_215_MOESM6_ESM.zip › Figure 4/4H/Images/western p62.tif]

Figure 5A

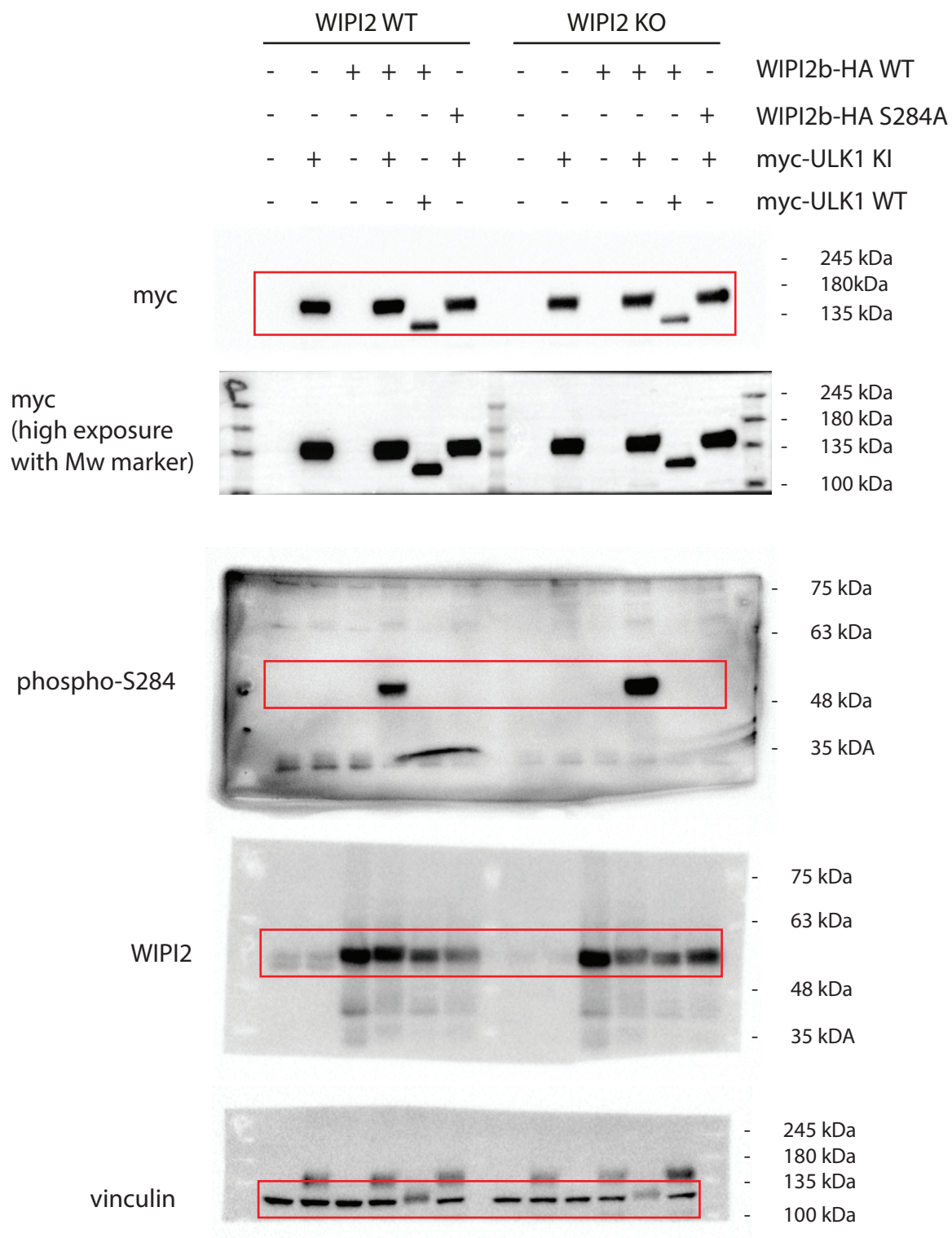

Supplement: Supplementary file 7 — Source data Fig. 5 [file 44319_2024_215_MOESM7_ESM.zip › Figure 5/5A/F5A.pdf]
