# Supplementary material for: WIPI2b recruitment to phagophores and ATG16L1 binding are regulated by ULK1 phosphorylation
Source: EMBO Rep. 2024 Aug 16;25(9):8. doi: 10.1038/s44319-024-00215-5 (PMC11387628; doi:10.1038/s44319-024-00215-5)
Supplement: Supplementary file 5 — Source data Fig. 3 [file 44319_2024_215_MOESM5_ESM.zip › Figure 3/3C/README.rtf]

the red panel was converted to grayscale and brightness and contrast were slightly changed for display purposes.images chosen for the figure panel were cropped from the originals included. the cropped area is shown in the accompanying pdf file, where unedited ‘merge’ panels were listed.
