# Supplementary material for: WIPI2b recruitment to phagophores and ATG16L1 binding are regulated by ULK1 phosphorylation
Source: EMBO Rep. 2024 Aug 16;25(9):8. doi: 10.1038/s44319-024-00215-5 (PMC11387628; doi:10.1038/s44319-024-00215-5)
Supplement: Supplementary file 7 — Source data Fig. 5 [file 44319_2024_215_MOESM7_ESM.zip › Figure 5/5C/README.rtf]

Image presented in greyscale in the figure.
