# Supplementary figures and images for: WIPI2b recruitment to phagophores and ATG16L1 binding are regulated by ULK1 phosphorylation (part 2 of 2)
Source: EMBO Rep. 2024 Aug 16;25(9):8. doi: 10.1038/s44319-024-00215-5 (PMC11387628; doi:10.1038/s44319-024-00215-5)

WIPI2b-HA WT

WIPI2b-HA S284A

WIPI2b-HA S284D

Fed

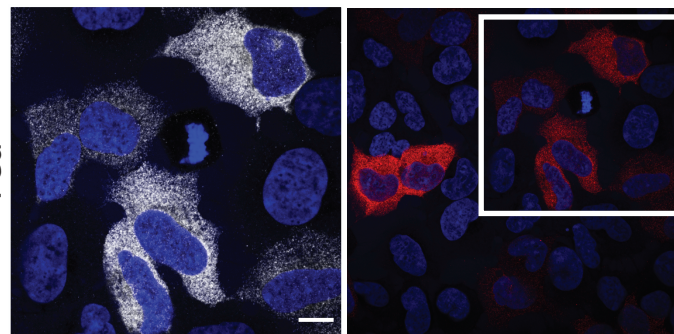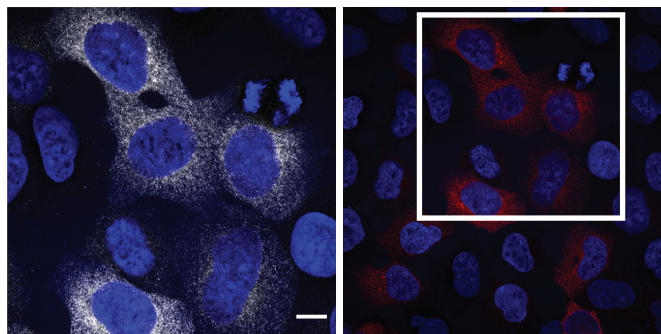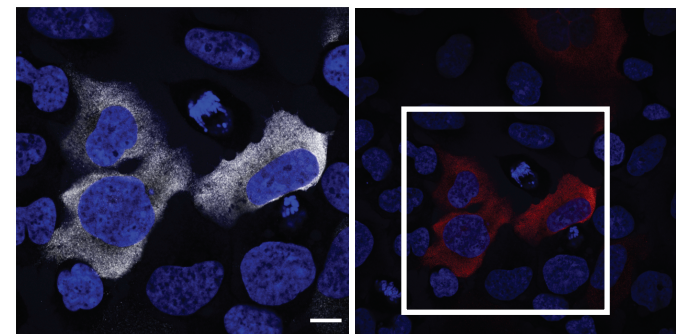

Starved

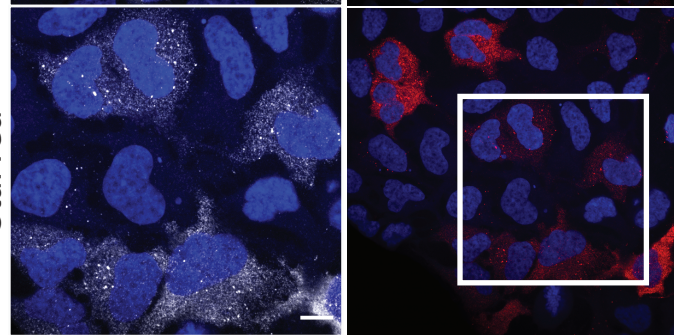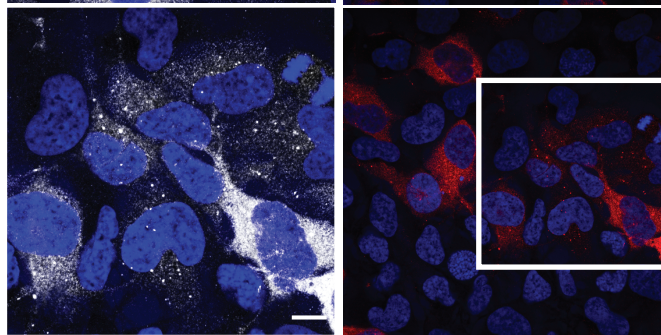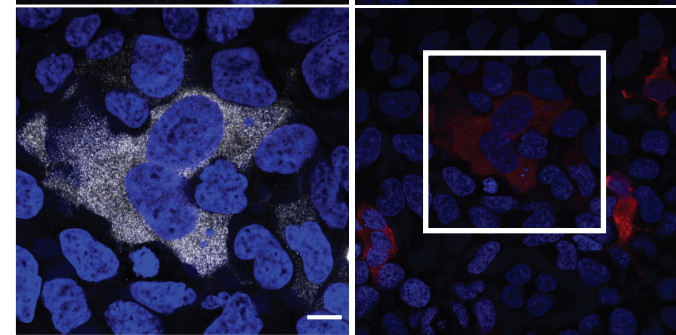

Starved + BafA1

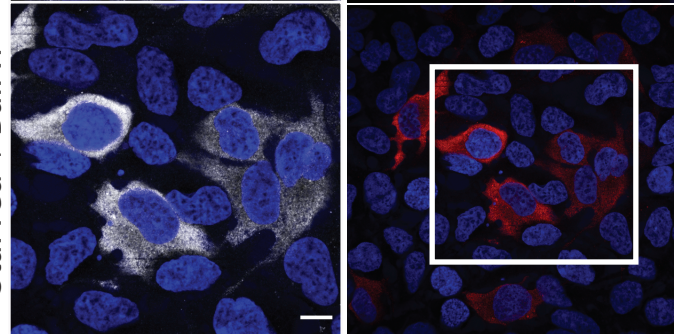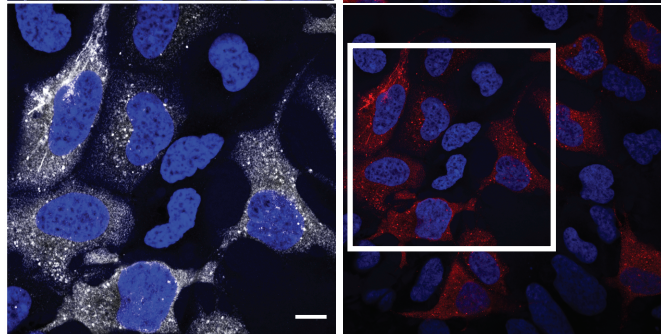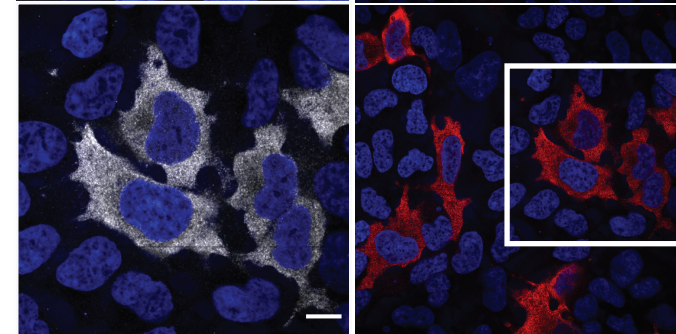

Supplement: Supplementary file 7 — Source data Fig. 5 [file 44319_2024_215_MOESM7_ESM.zip › Figure 5/5F/F5F.pdf]

Figure 5C

dashed line shows where the gel was cut

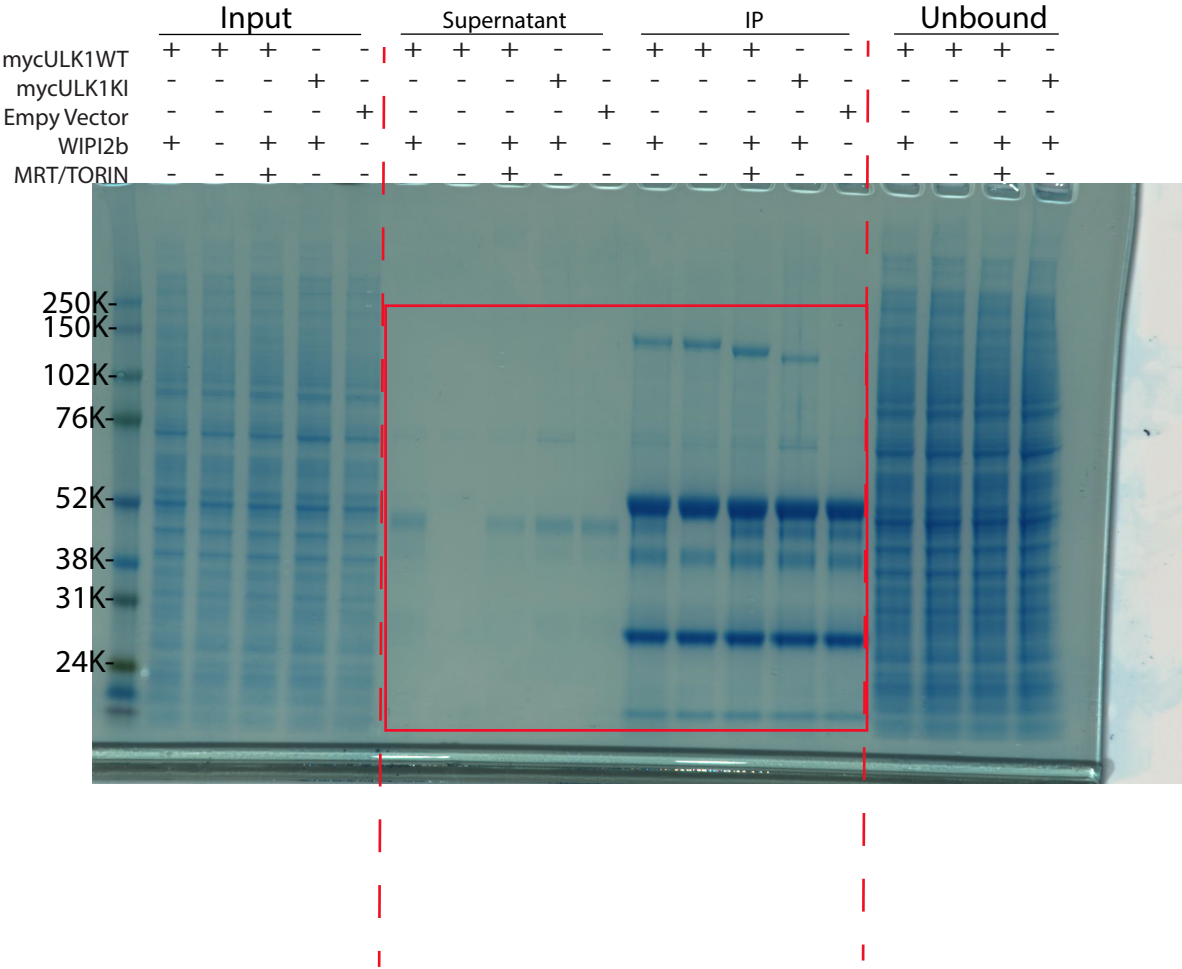

Supplement: Supplementary file 7 — Source data Fig. 5 [file 44319_2024_215_MOESM7_ESM.zip › Figure 5/5C/F5C.pdf]

Figure 5D

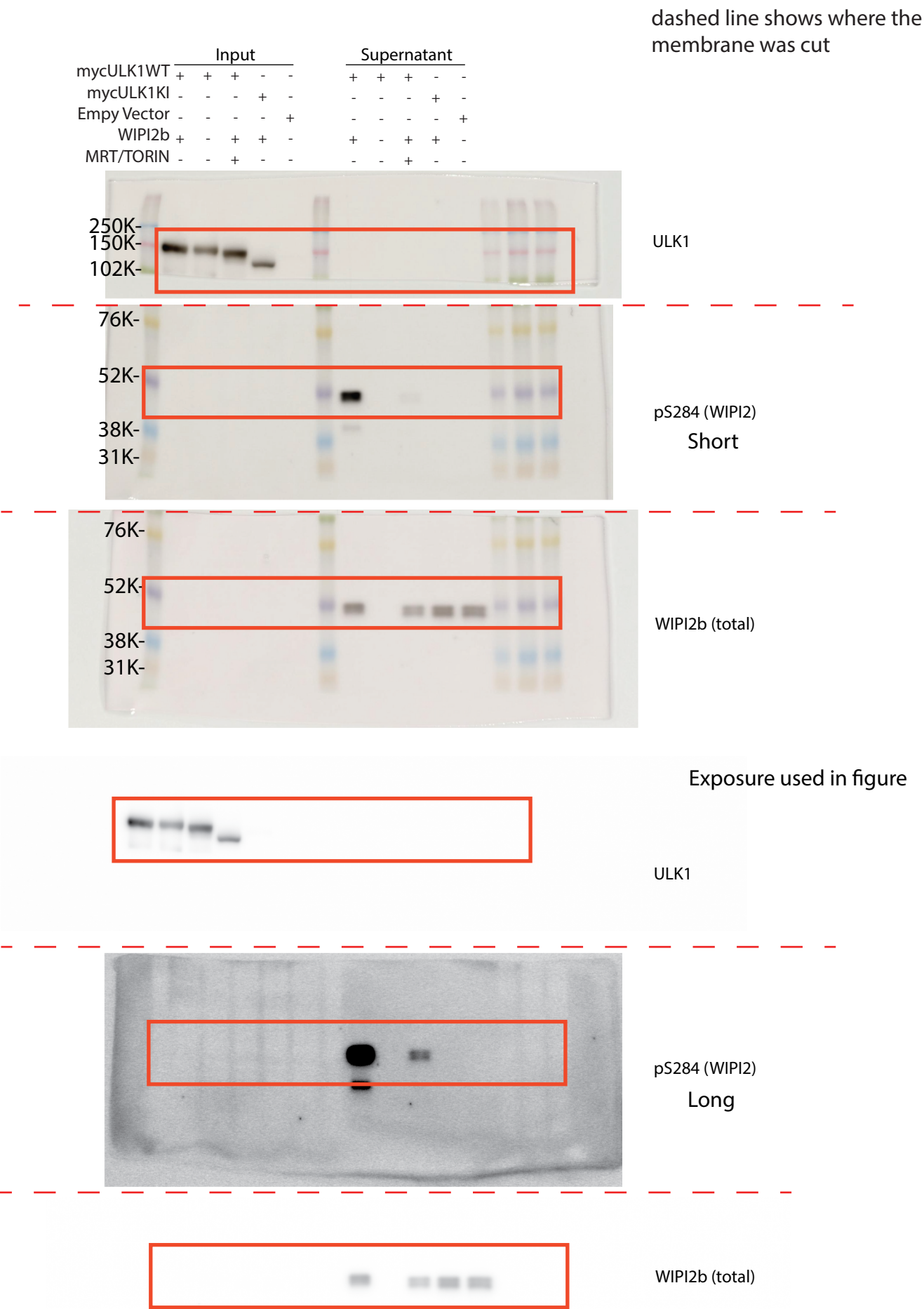

Supplement: Supplementary file 7 — Source data Fig. 5 [file 44319_2024_215_MOESM7_ESM.zip › Figure 5/5D/F5D.pdf]

Figure 5B

dashed line shows where the membrane was cut

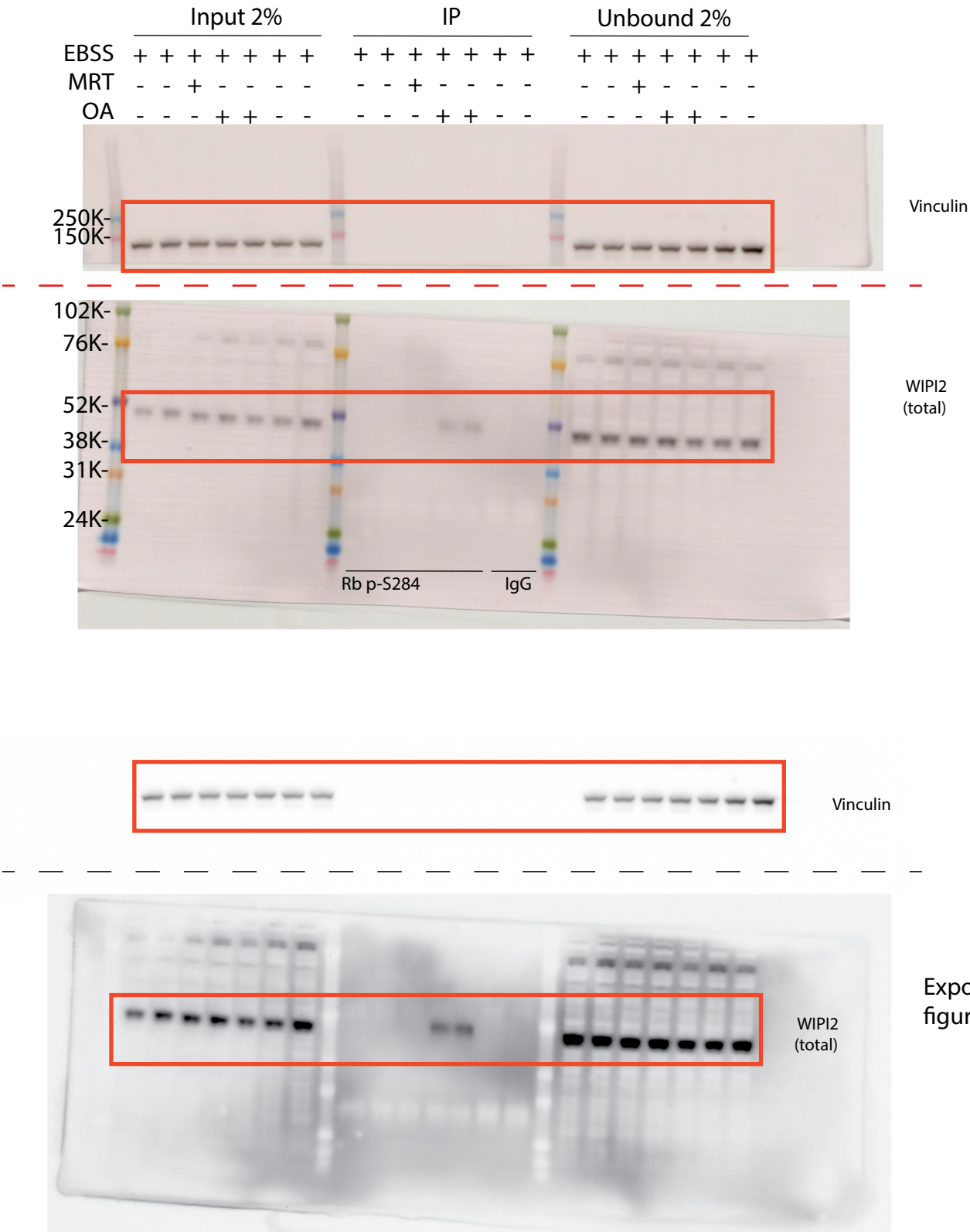

Supplement: Supplementary file 7 — Source data Fig. 5 [file 44319_2024_215_MOESM7_ESM.zip › Figure 5/5B/F5B.pdf]

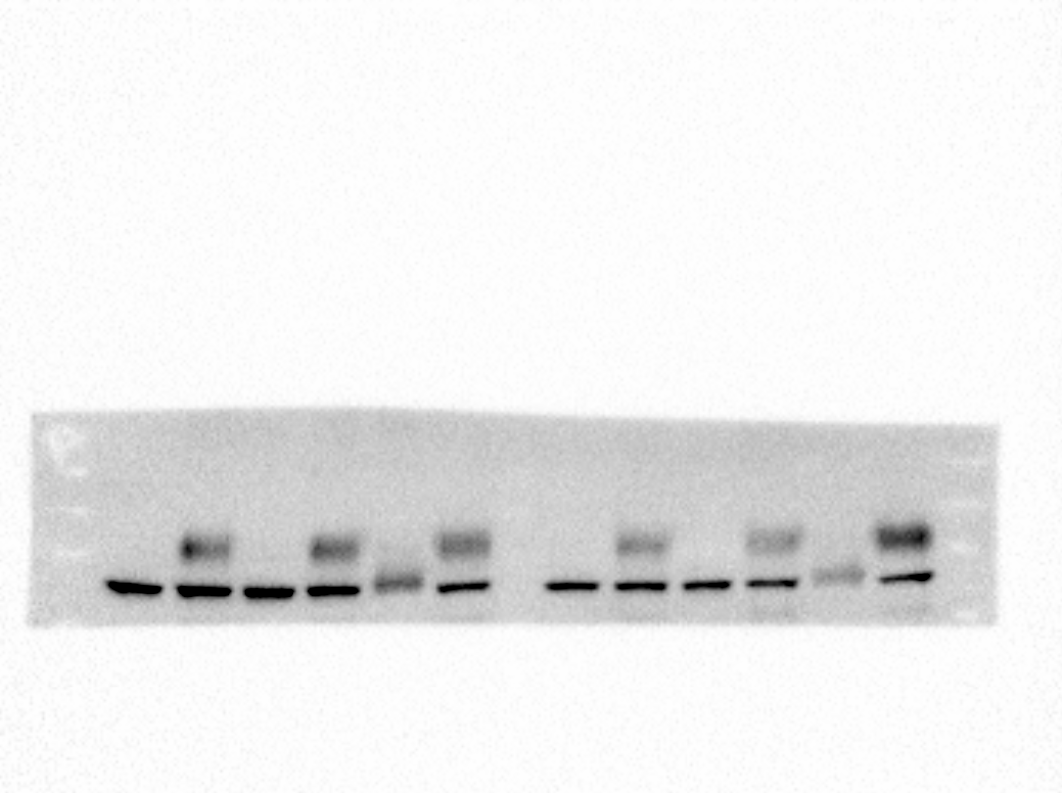

Supplement: Supplementary file 7 — Source data Fig. 5 [file 44319_2024_215_MOESM7_ESM.zip › Figure 5/5A/Images/western vinuclin.tif]

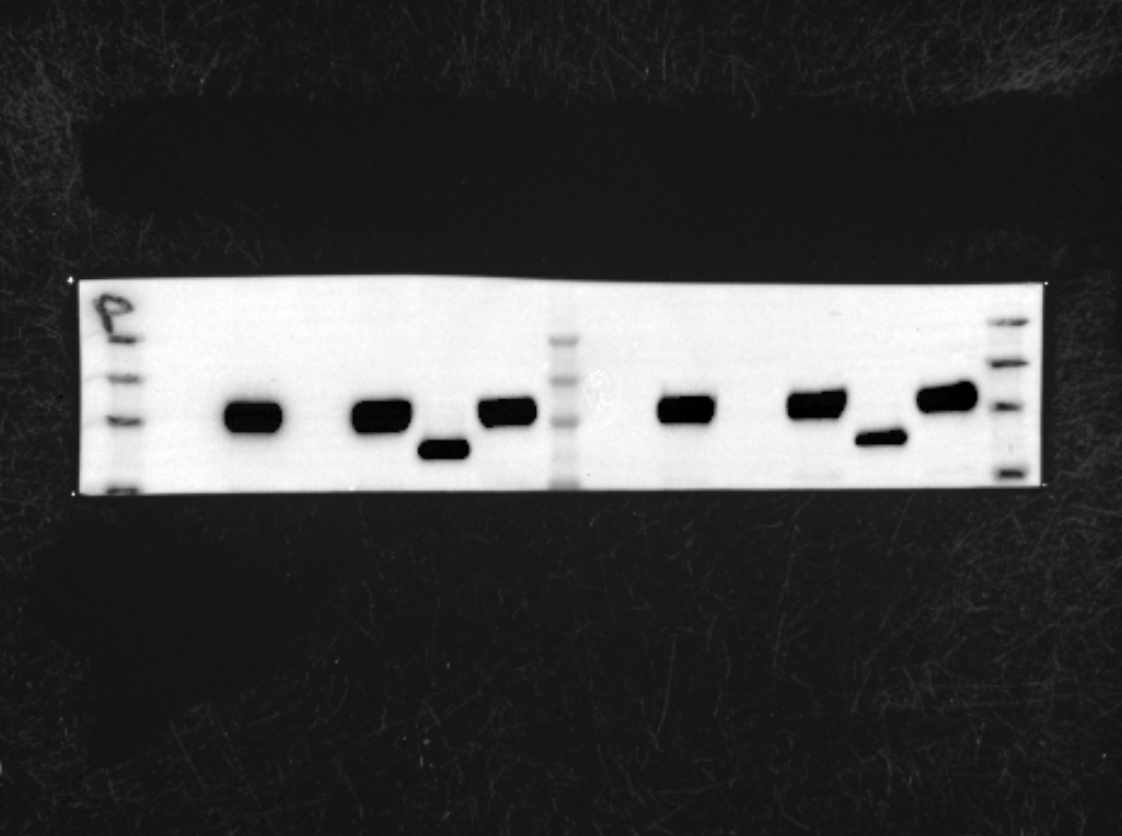

Supplement: Supplementary file 7 — Source data Fig. 5 [file 44319_2024_215_MOESM7_ESM.zip › Figure 5/5A/Images/western myc with marker.tif]

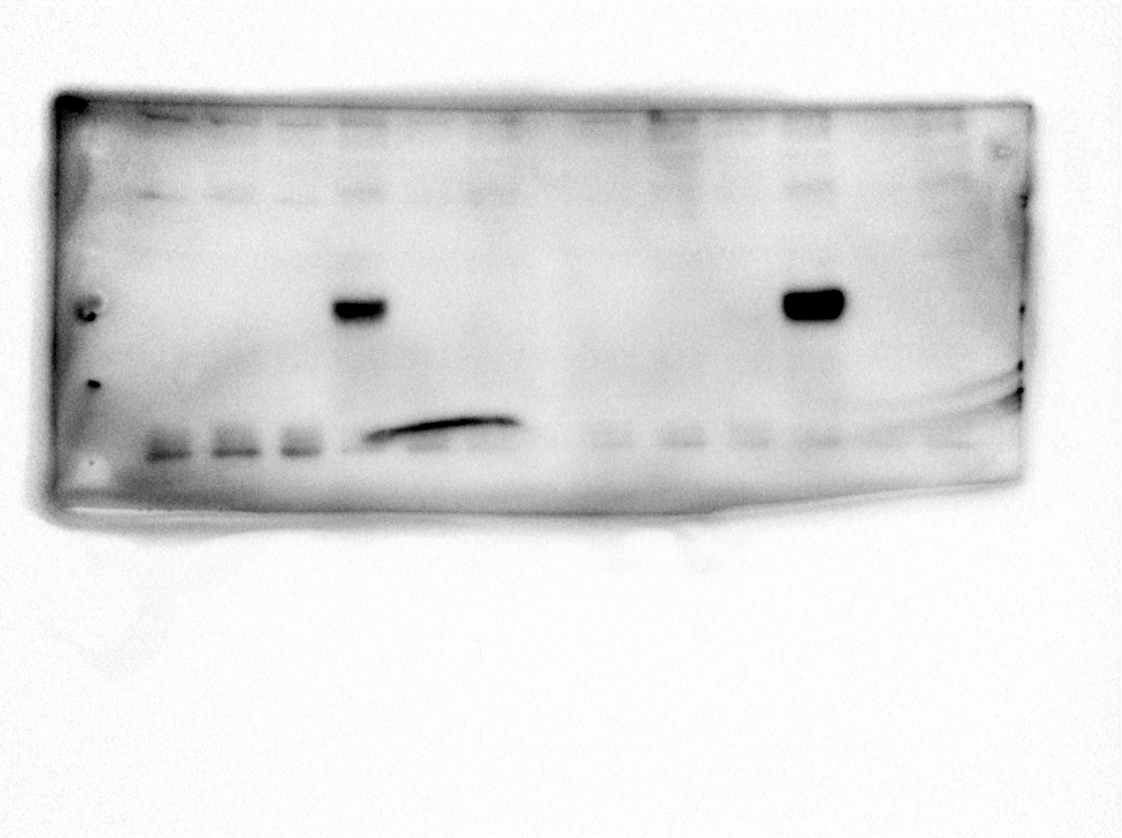

Supplement: Supplementary file 7 — Source data Fig. 5 [file 44319_2024_215_MOESM7_ESM.zip › Figure 5/5A/Images/western phospho S284.tif]

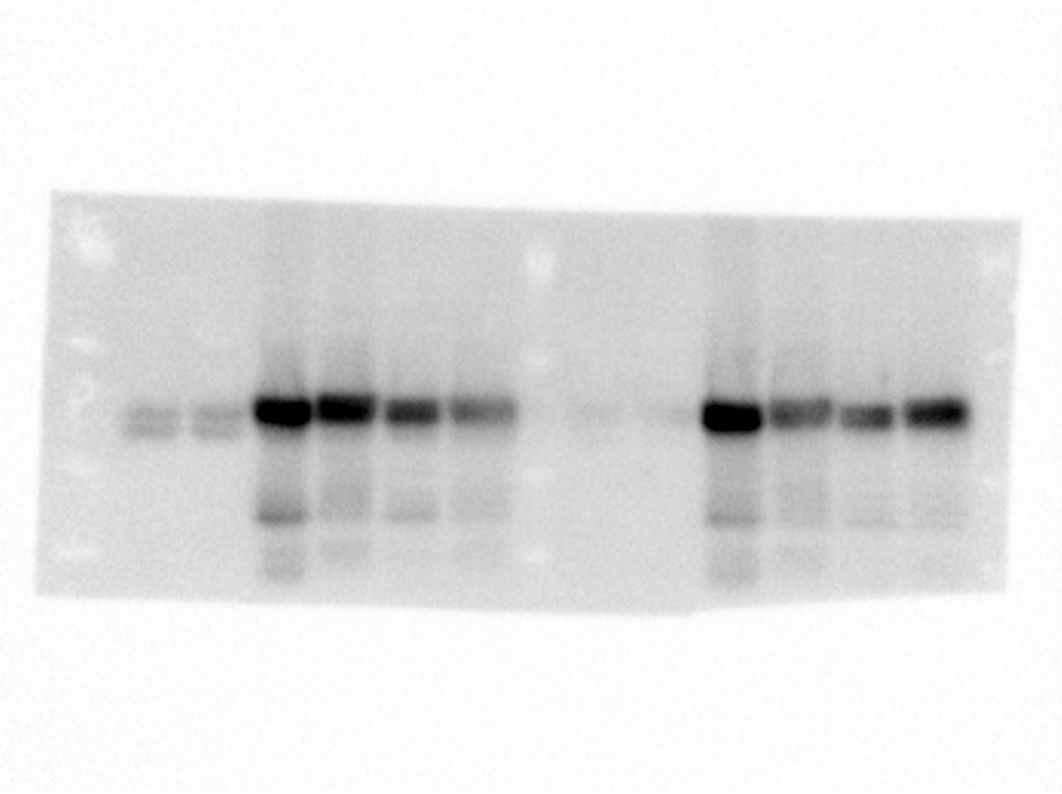

Supplement: Supplementary file 7 — Source data Fig. 5 [file 44319_2024_215_MOESM7_ESM.zip › Figure 5/5A/Images/western WIPI2.tif]

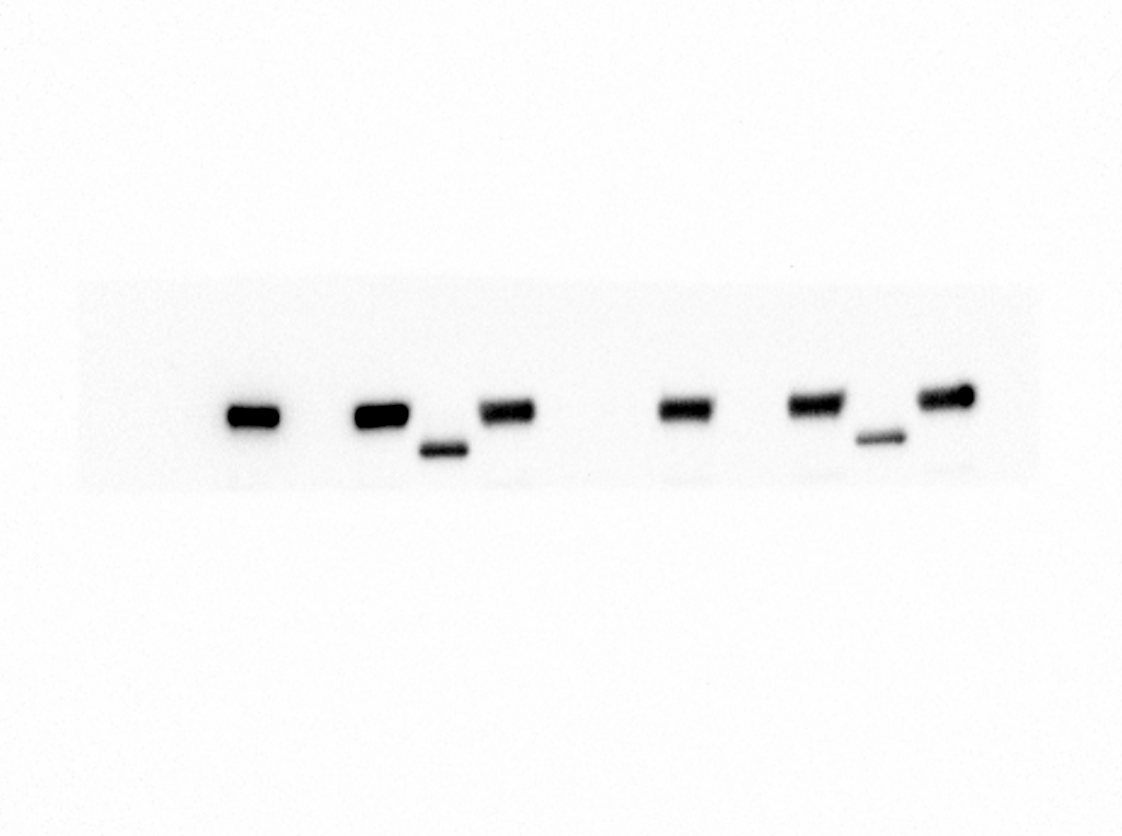

Supplement: Supplementary file 7 — Source data Fig. 5 [file 44319_2024_215_MOESM7_ESM.zip › Figure 5/5A/Images/western myc.tif]

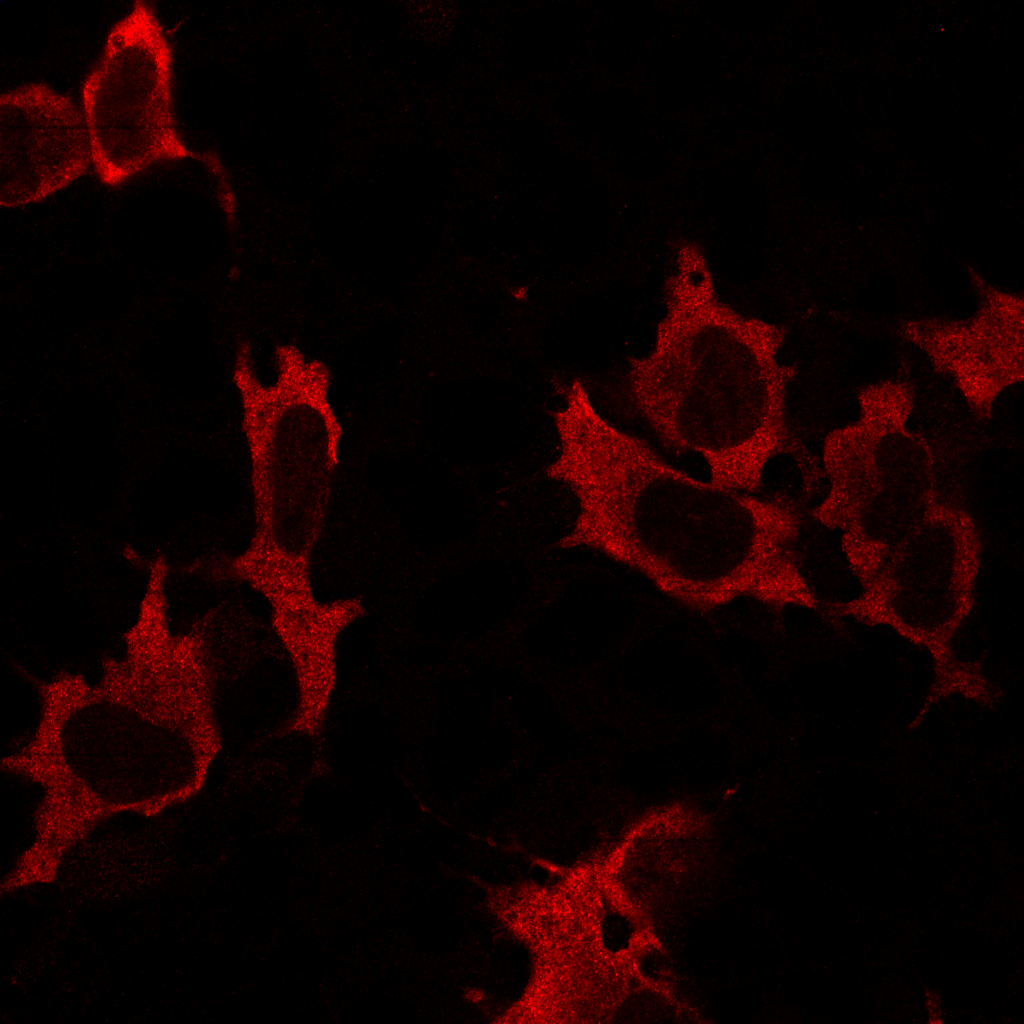

Supplement: Supplementary file 7 — Source data Fig. 5 [file 44319_2024_215_MOESM7_ESM.zip › Figure 5/5F/Images/S284D_StarvedBaf_HA.tif]

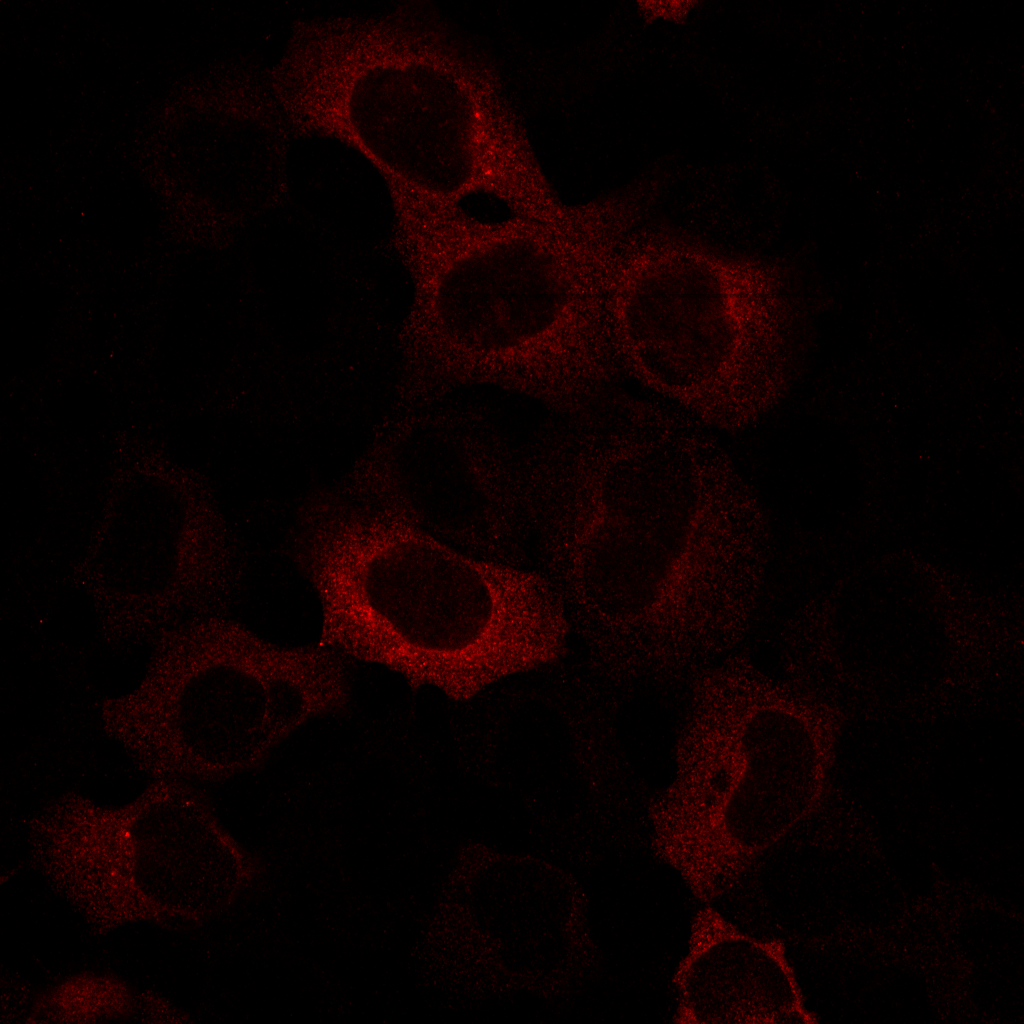

Supplement: Supplementary file 7 — Source data Fig. 5 [file 44319_2024_215_MOESM7_ESM.zip › Figure 5/5F/Images/S284A_Fed_HA.tif]

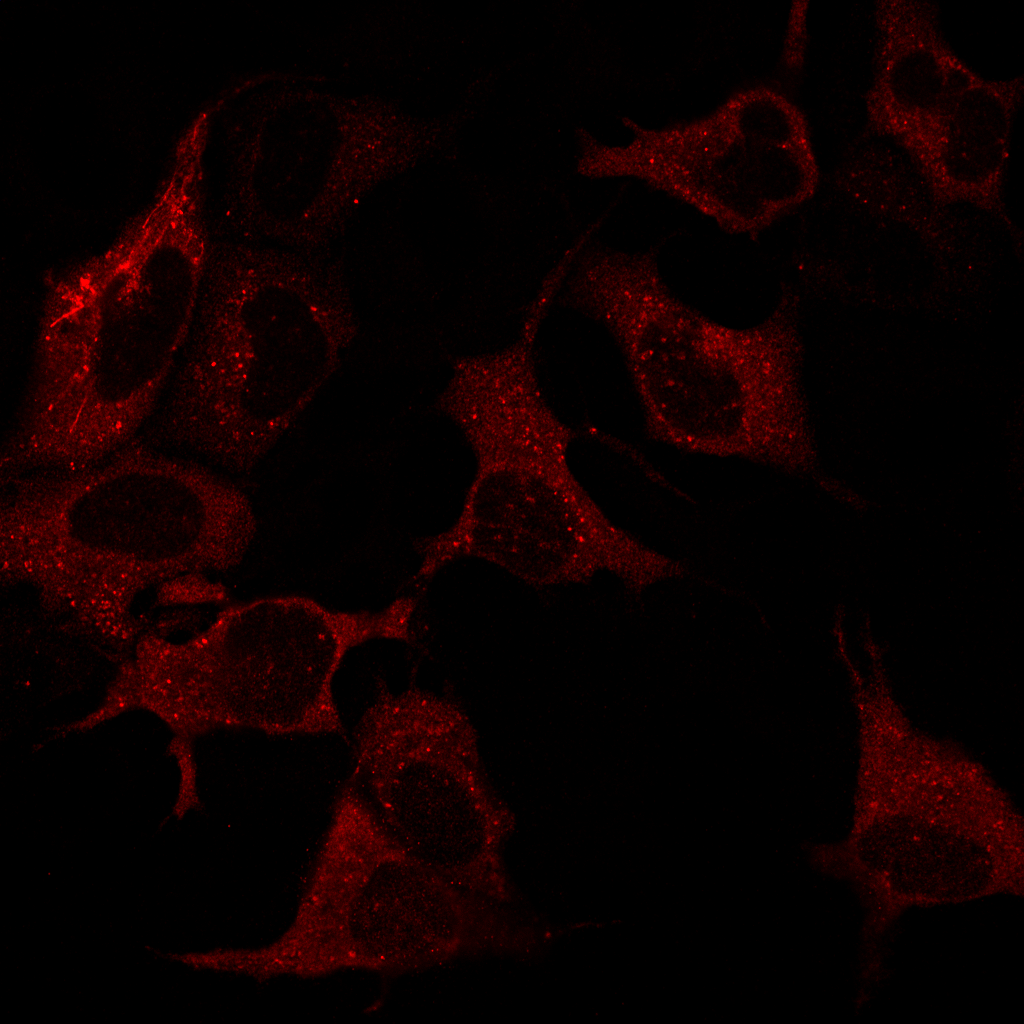

Supplement: Supplementary file 7 — Source data Fig. 5 [file 44319_2024_215_MOESM7_ESM.zip › Figure 5/5F/Images/S284A_StarvedBaf_HA.tif]

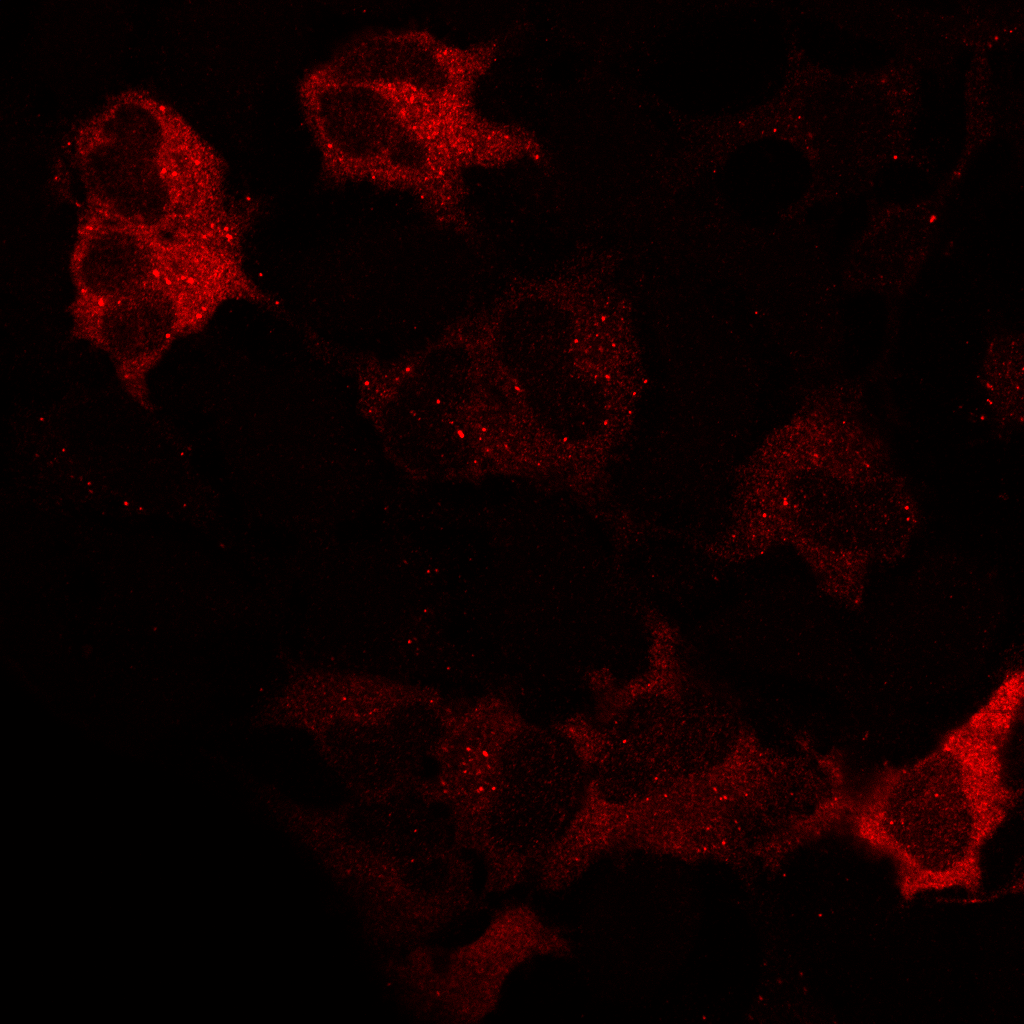

Supplement: Supplementary file 7 — Source data Fig. 5 [file 44319_2024_215_MOESM7_ESM.zip › Figure 5/5F/Images/WT_Starved_HA.tif]

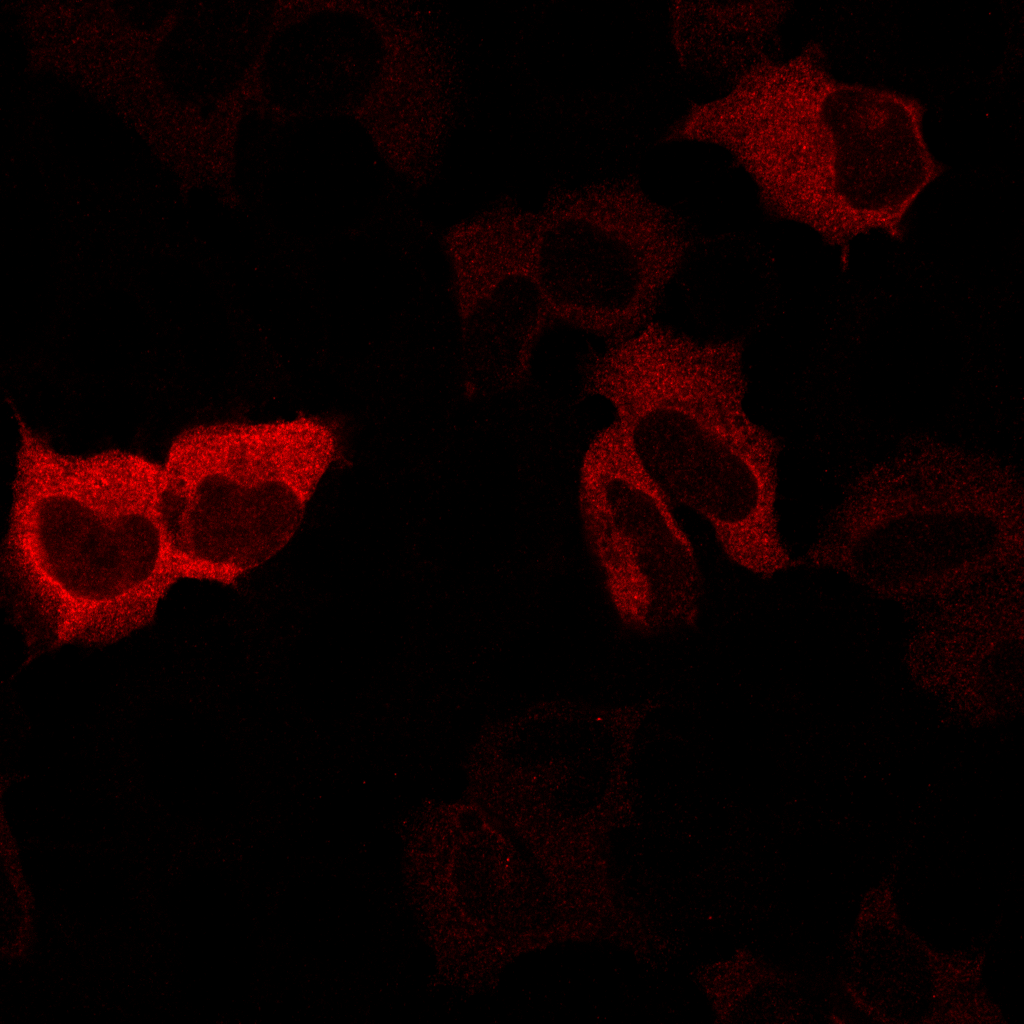

Supplement: Supplementary file 7 — Source data Fig. 5 [file 44319_2024_215_MOESM7_ESM.zip › Figure 5/5F/Images/WT_Fed_HA.tif]

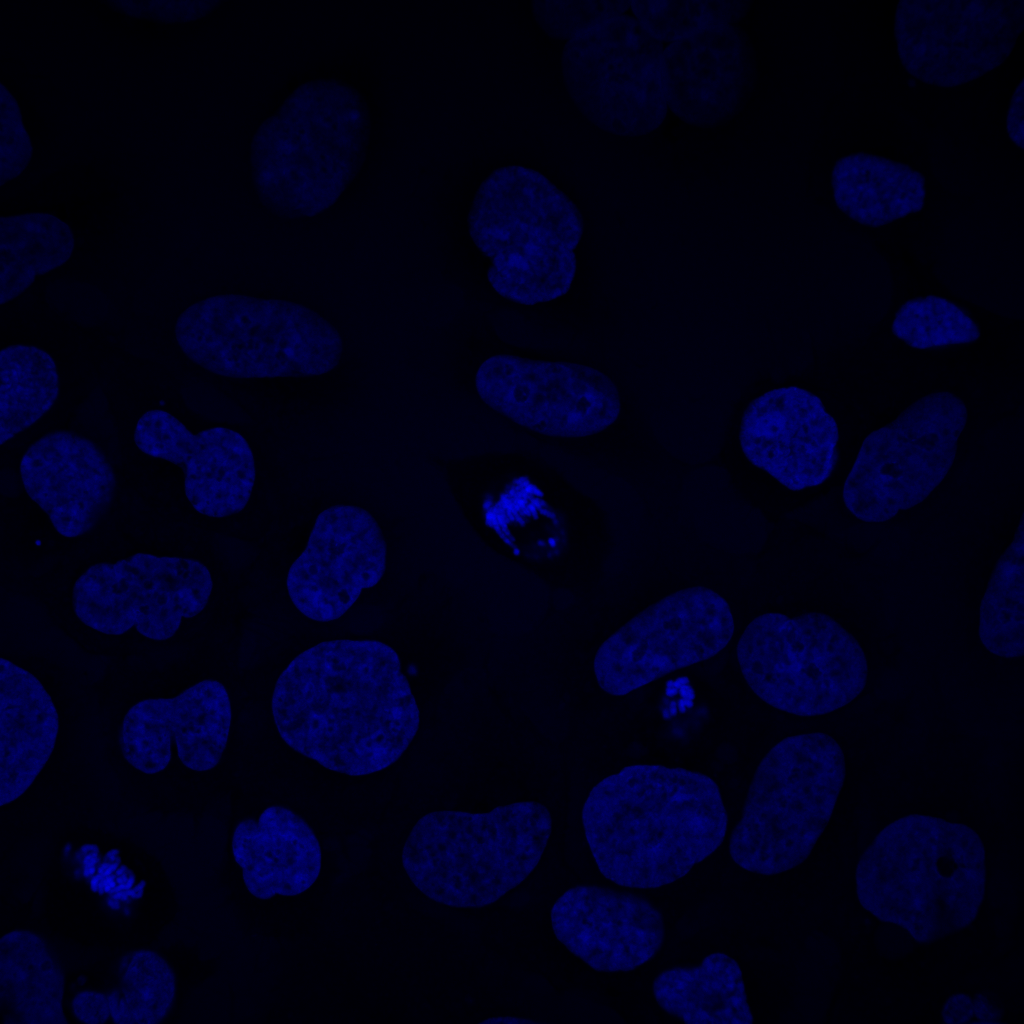

Supplement: Supplementary file 7 — Source data Fig. 5 [file 44319_2024_215_MOESM7_ESM.zip › Figure 5/5F/Images/S284D_Fed_hoechst.tif]

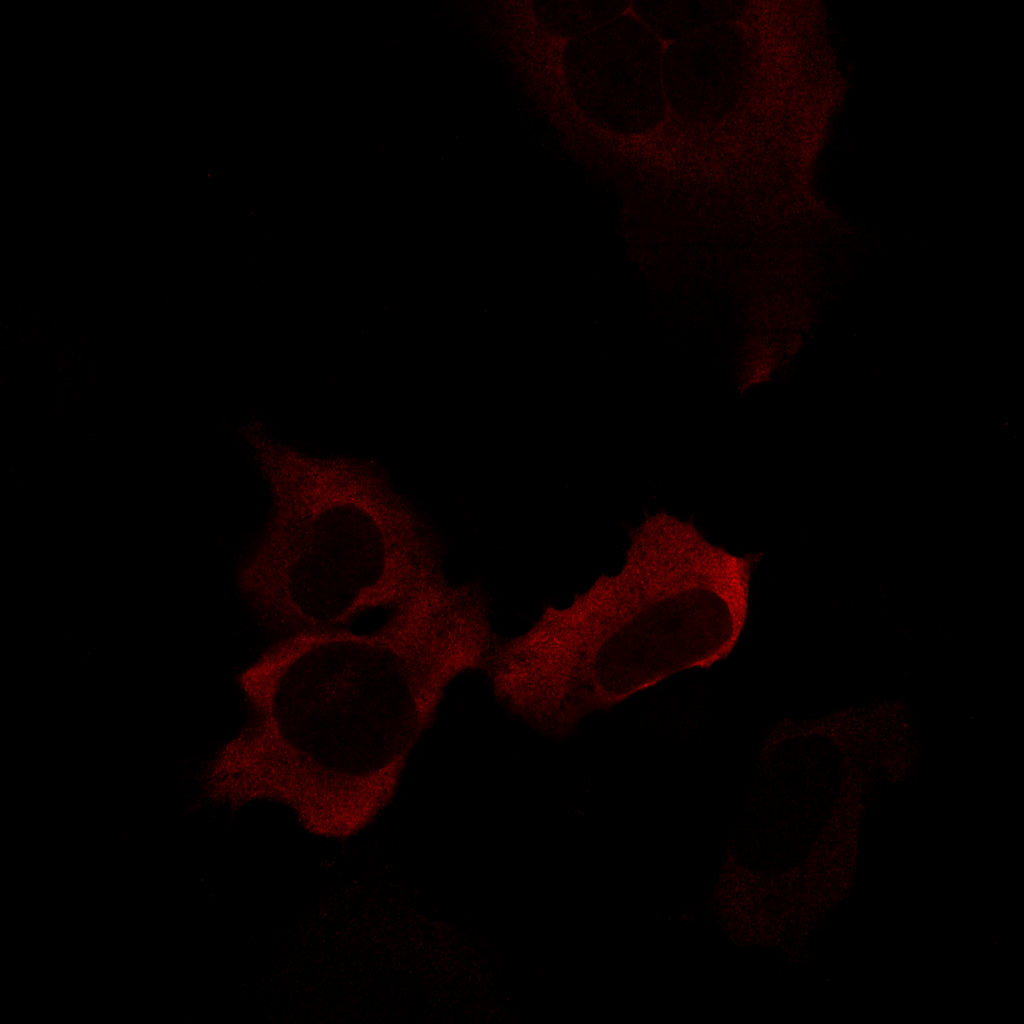

Supplement: Supplementary file 7 — Source data Fig. 5 [file 44319_2024_215_MOESM7_ESM.zip › Figure 5/5F/Images/S284D_Fed_HA.tif]

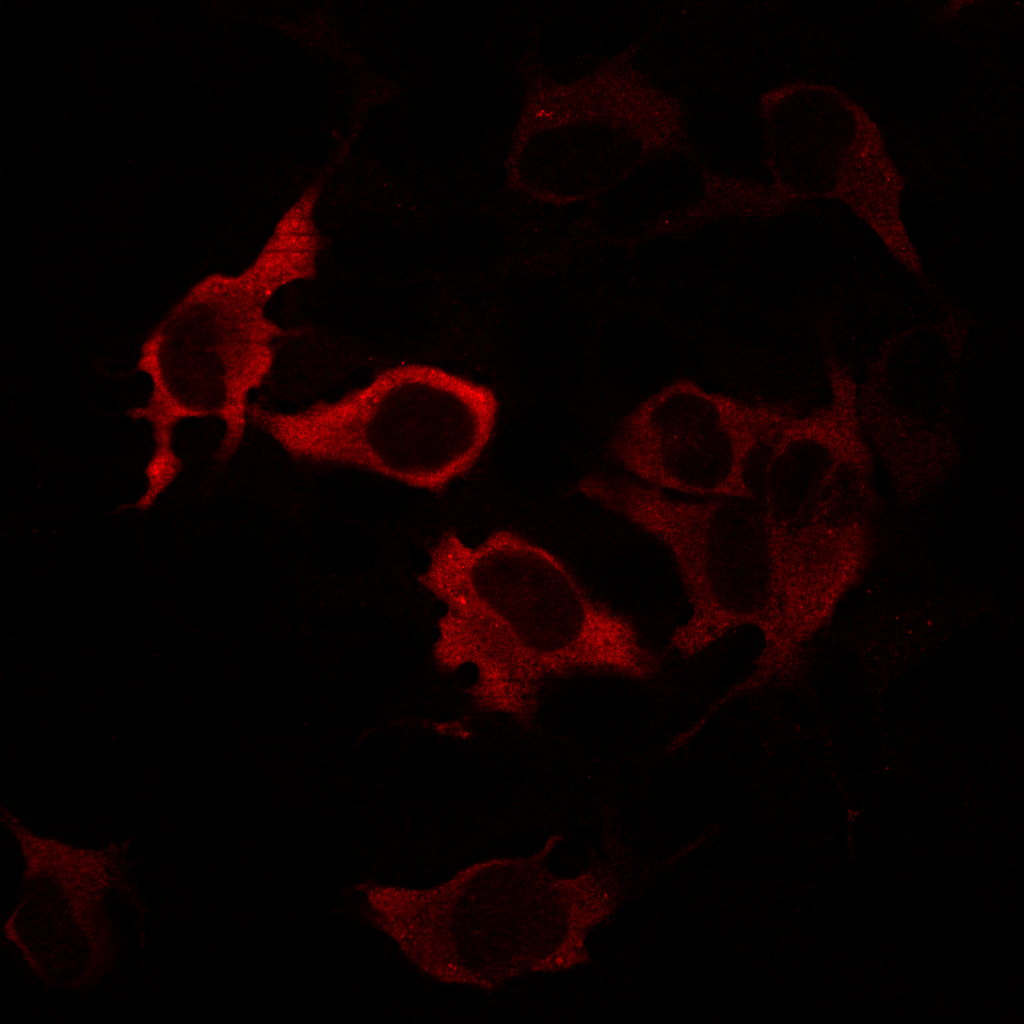

Supplement: Supplementary file 7 — Source data Fig. 5 [file 44319_2024_215_MOESM7_ESM.zip › Figure 5/5F/Images/WT_StarvedBaf_HA.tif]

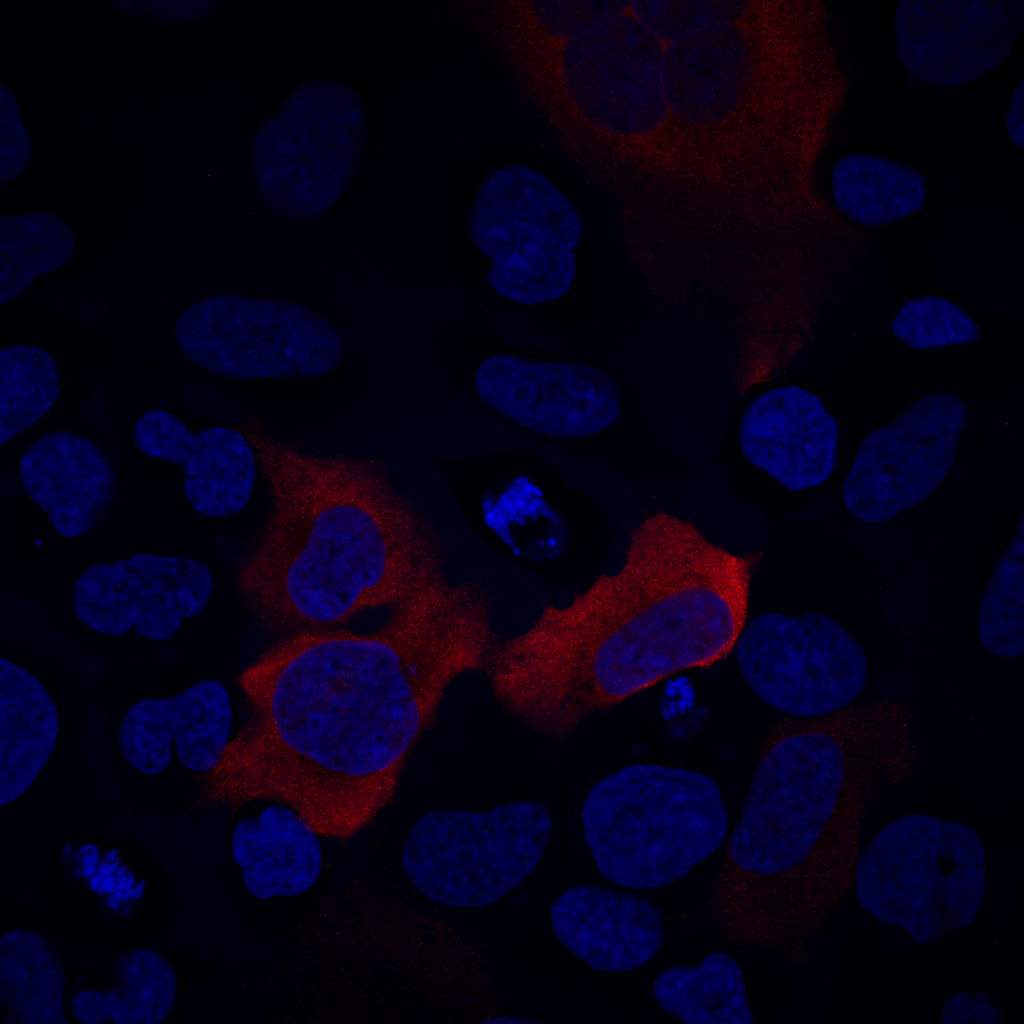

Supplement: Supplementary file 7 — Source data Fig. 5 [file 44319_2024_215_MOESM7_ESM.zip › Figure 5/5F/Images/S284D_Fed_merge.tif]

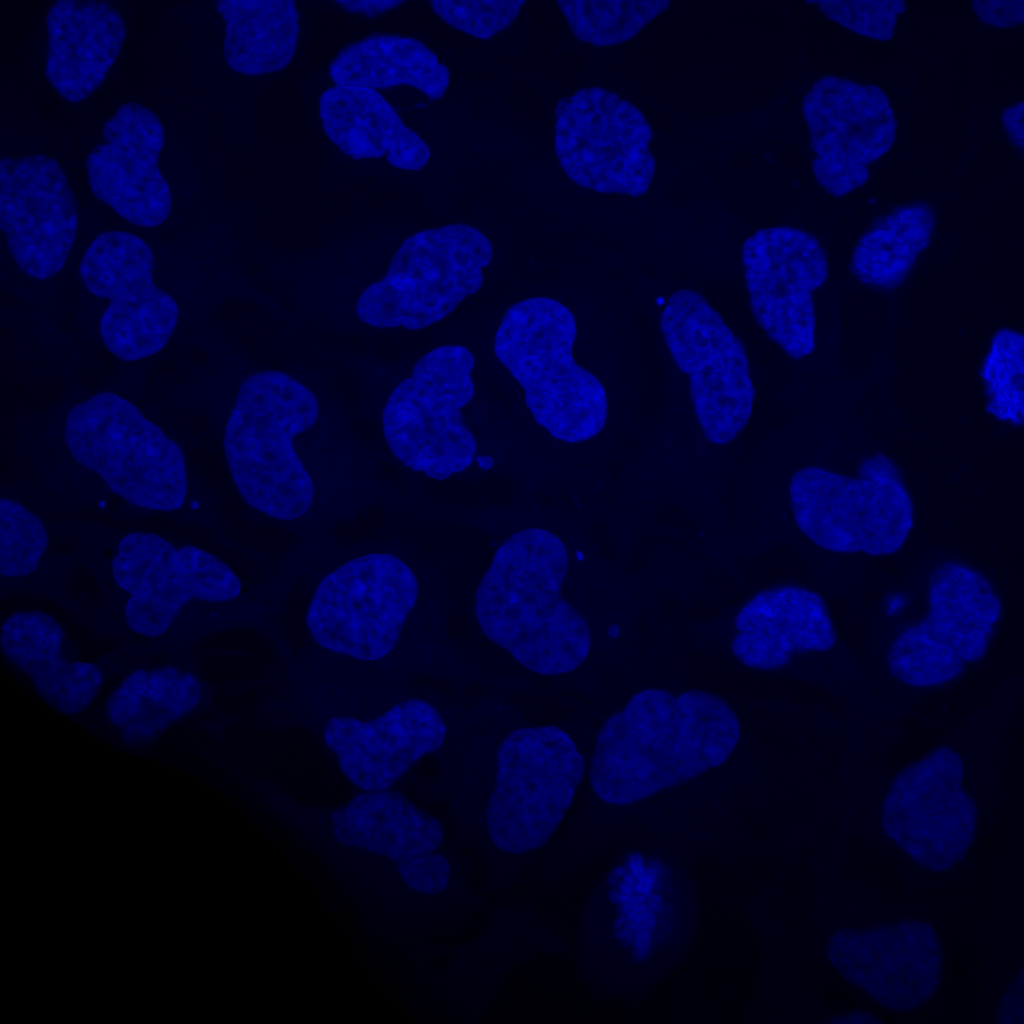

Supplement: Supplementary file 7 — Source data Fig. 5 [file 44319_2024_215_MOESM7_ESM.zip › Figure 5/5F/Images/WT_starved_hoechst.tif]

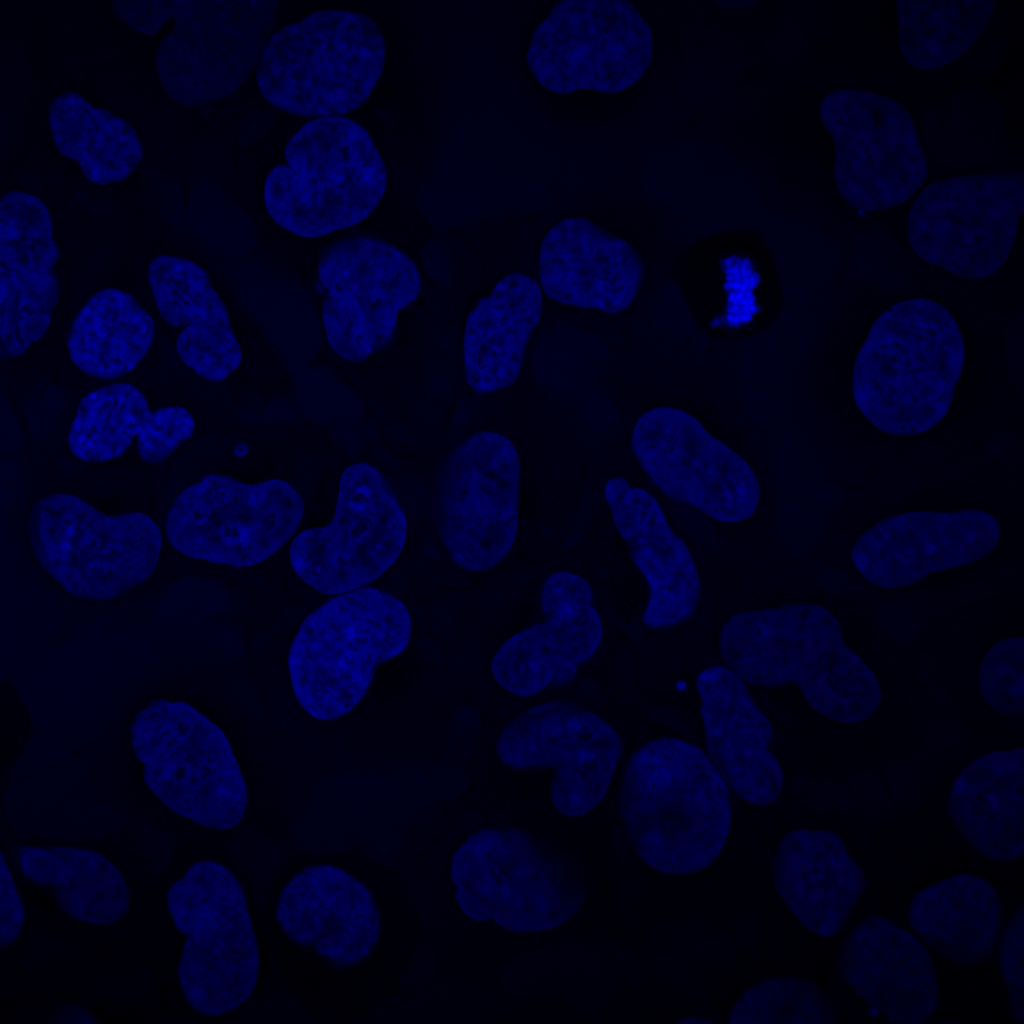

Supplement: Supplementary file 7 — Source data Fig. 5 [file 44319_2024_215_MOESM7_ESM.zip › Figure 5/5F/Images/WT_Fed_hoechst.tif]

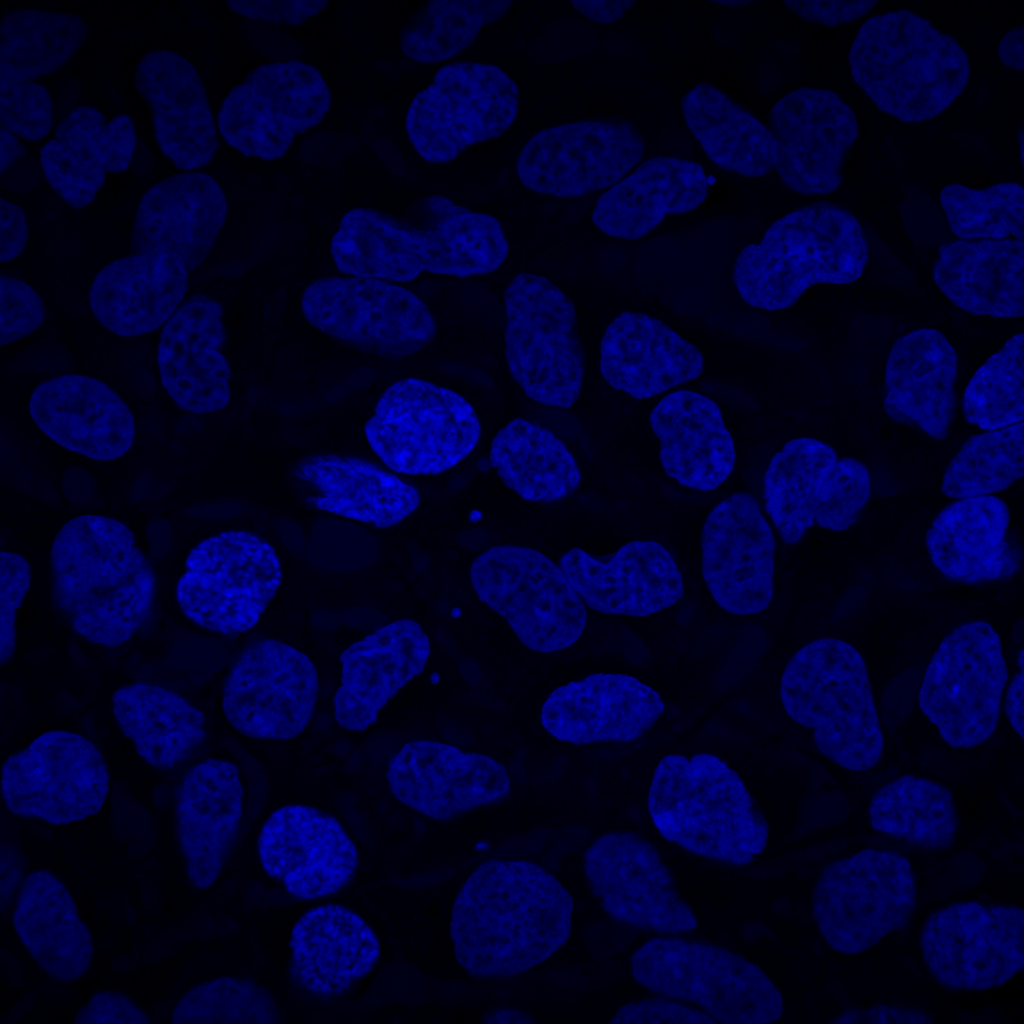

Supplement: Supplementary file 7 — Source data Fig. 5 [file 44319_2024_215_MOESM7_ESM.zip › Figure 5/5F/Images/WT_StarvedBaf_hoechst.tif]

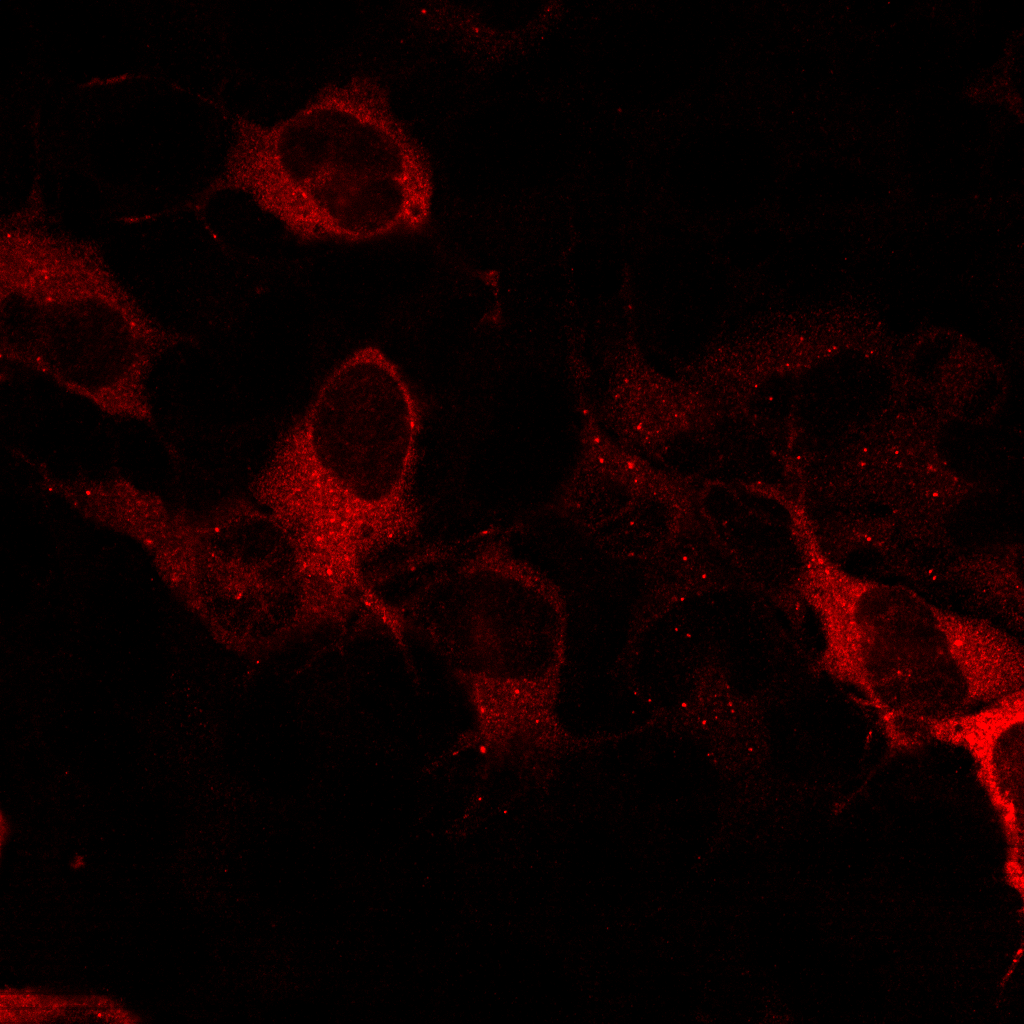

Supplement: Supplementary file 7 — Source data Fig. 5 [file 44319_2024_215_MOESM7_ESM.zip › Figure 5/5F/Images/S284A_Starved_HA.tif]

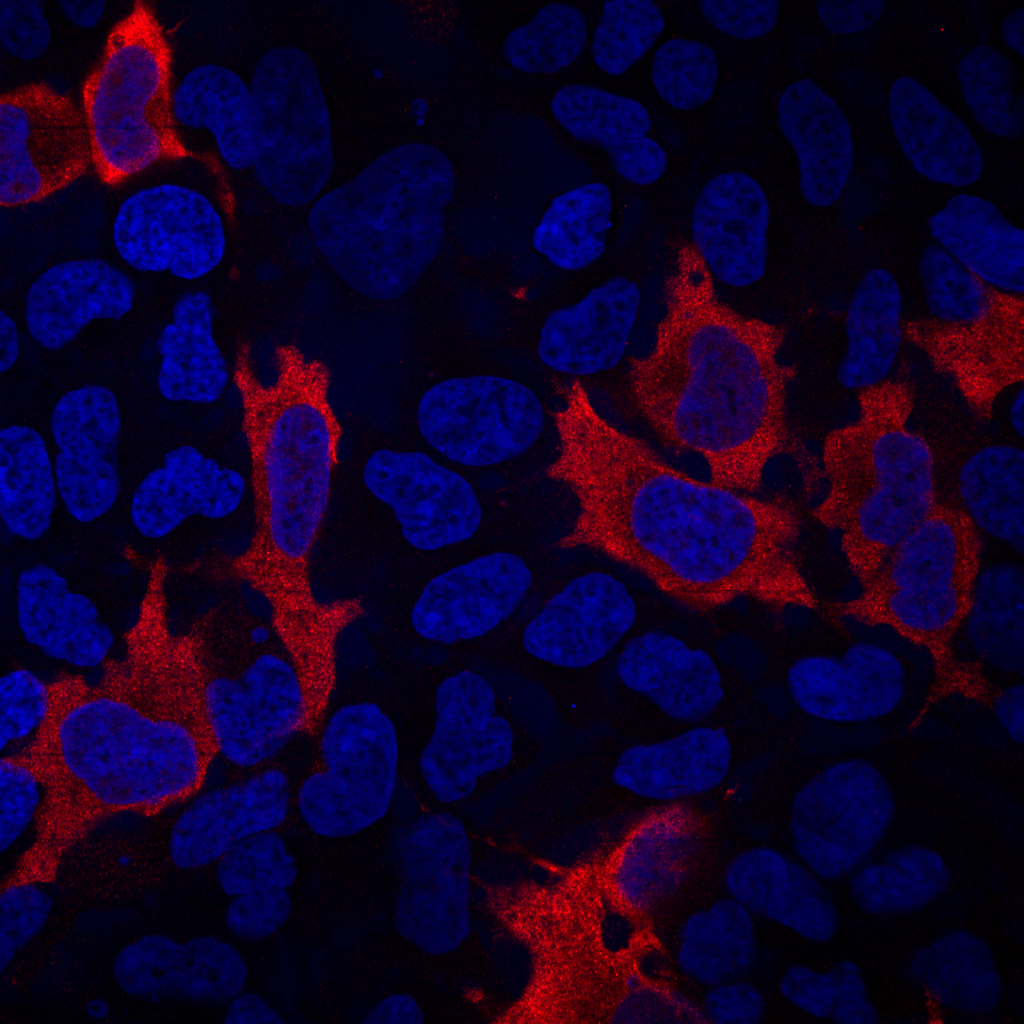

Supplement: Supplementary file 7 — Source data Fig. 5 [file 44319_2024_215_MOESM7_ESM.zip › Figure 5/5F/Images/S284D_StarvedBaf_merge.tif]

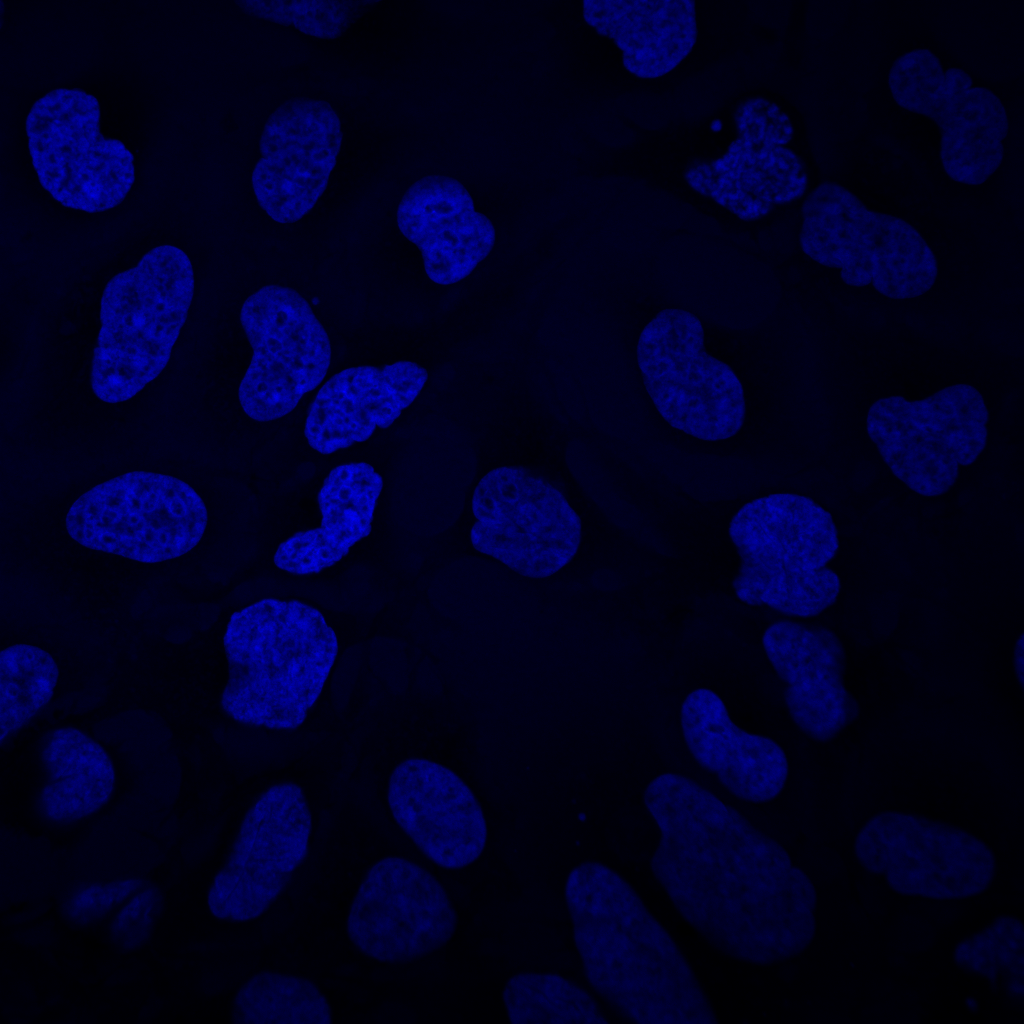

Supplement: Supplementary file 7 — Source data Fig. 5 [file 44319_2024_215_MOESM7_ESM.zip › Figure 5/5F/Images/S284A_StarvedBaf_hoechst.tif]

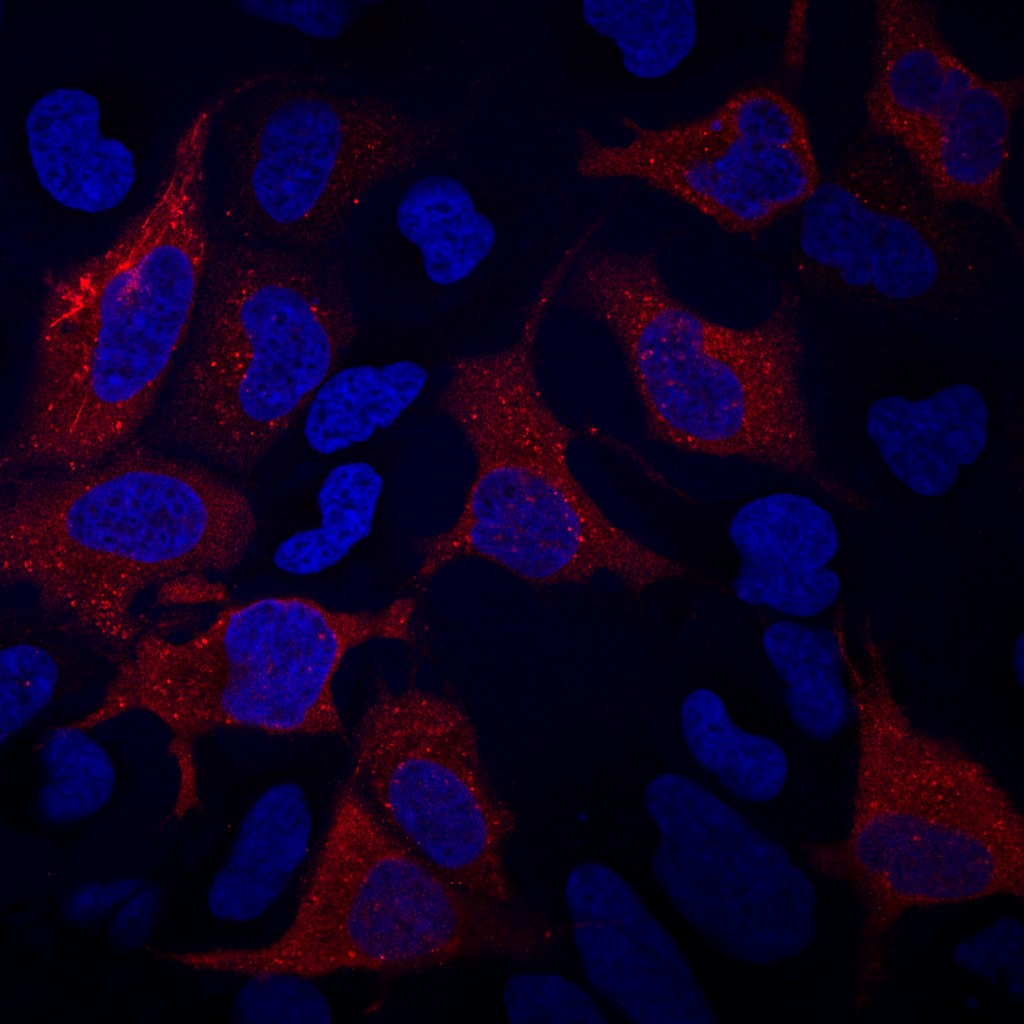

Supplement: Supplementary file 7 — Source data Fig. 5 [file 44319_2024_215_MOESM7_ESM.zip › Figure 5/5F/Images/S284A_StarvedBaf_merge.tif]

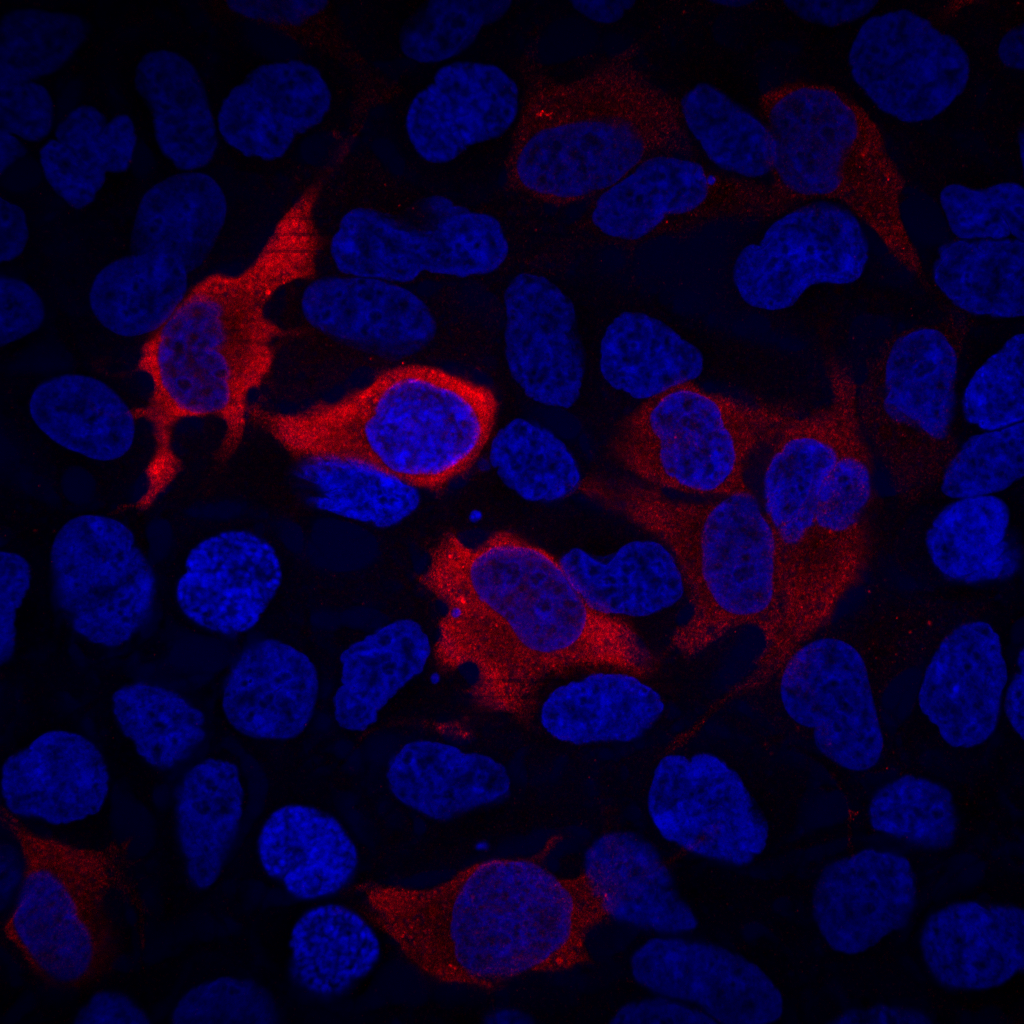

Supplement: Supplementary file 7 — Source data Fig. 5 [file 44319_2024_215_MOESM7_ESM.zip › Figure 5/5F/Images/WT_StarvedBaf_merge.tif]

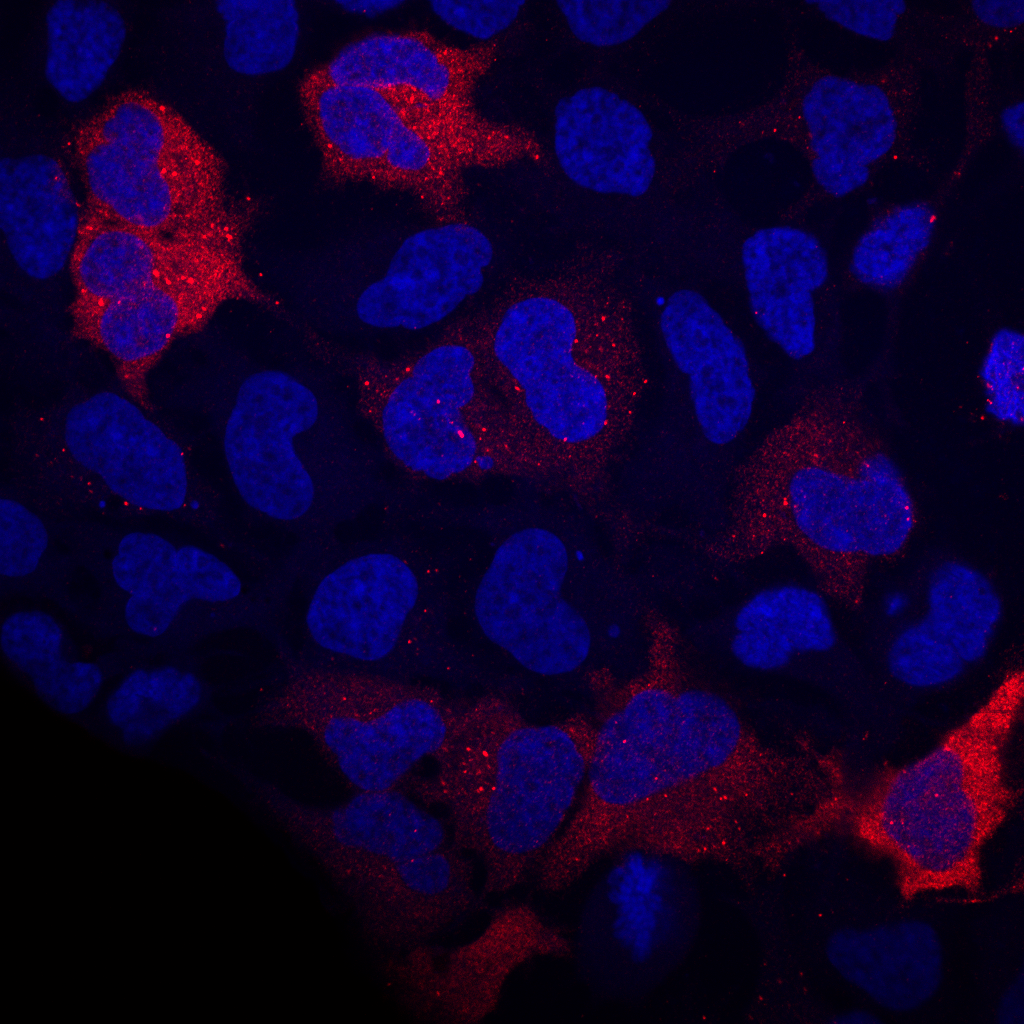

Supplement: Supplementary file 7 — Source data Fig. 5 [file 44319_2024_215_MOESM7_ESM.zip › Figure 5/5F/Images/WT_starved_merge.tif]

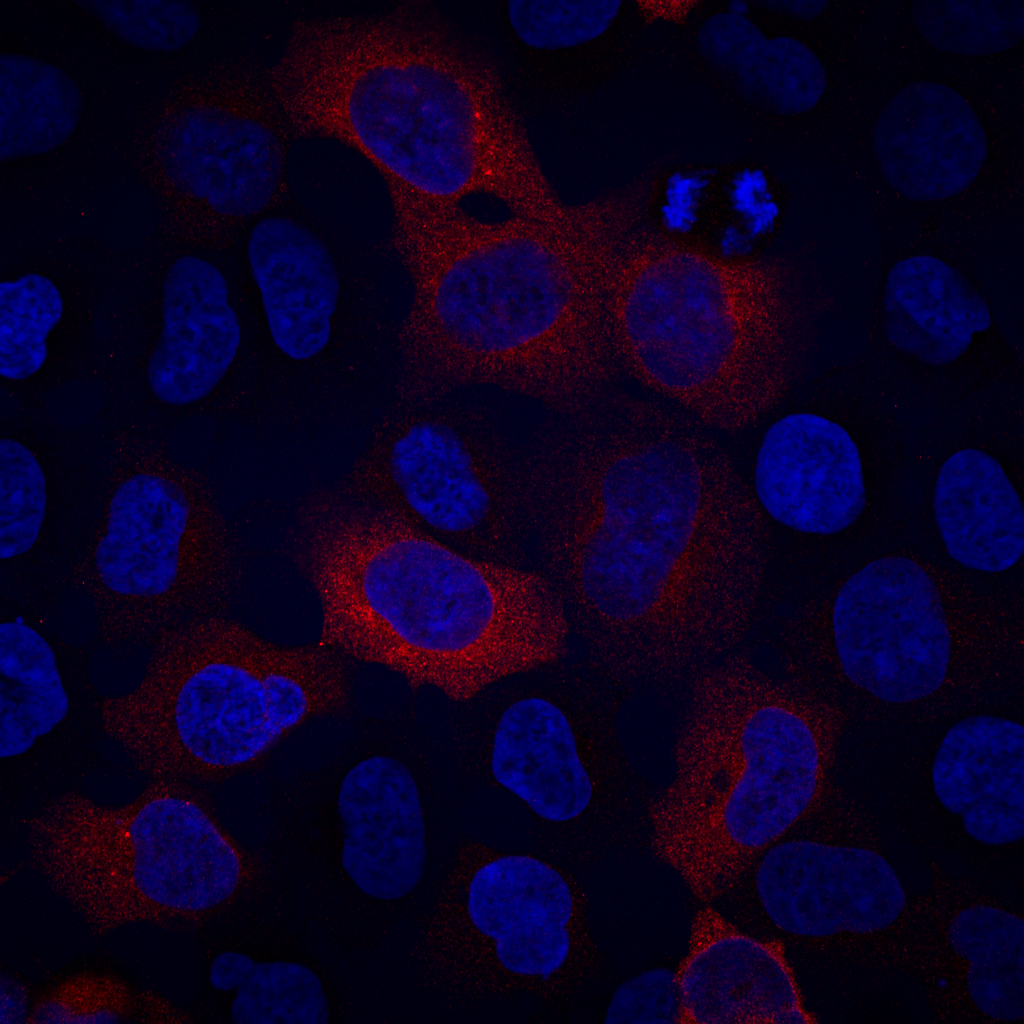

Supplement: Supplementary file 7 — Source data Fig. 5 [file 44319_2024_215_MOESM7_ESM.zip › Figure 5/5F/Images/S284A_Fed_merge.tif]

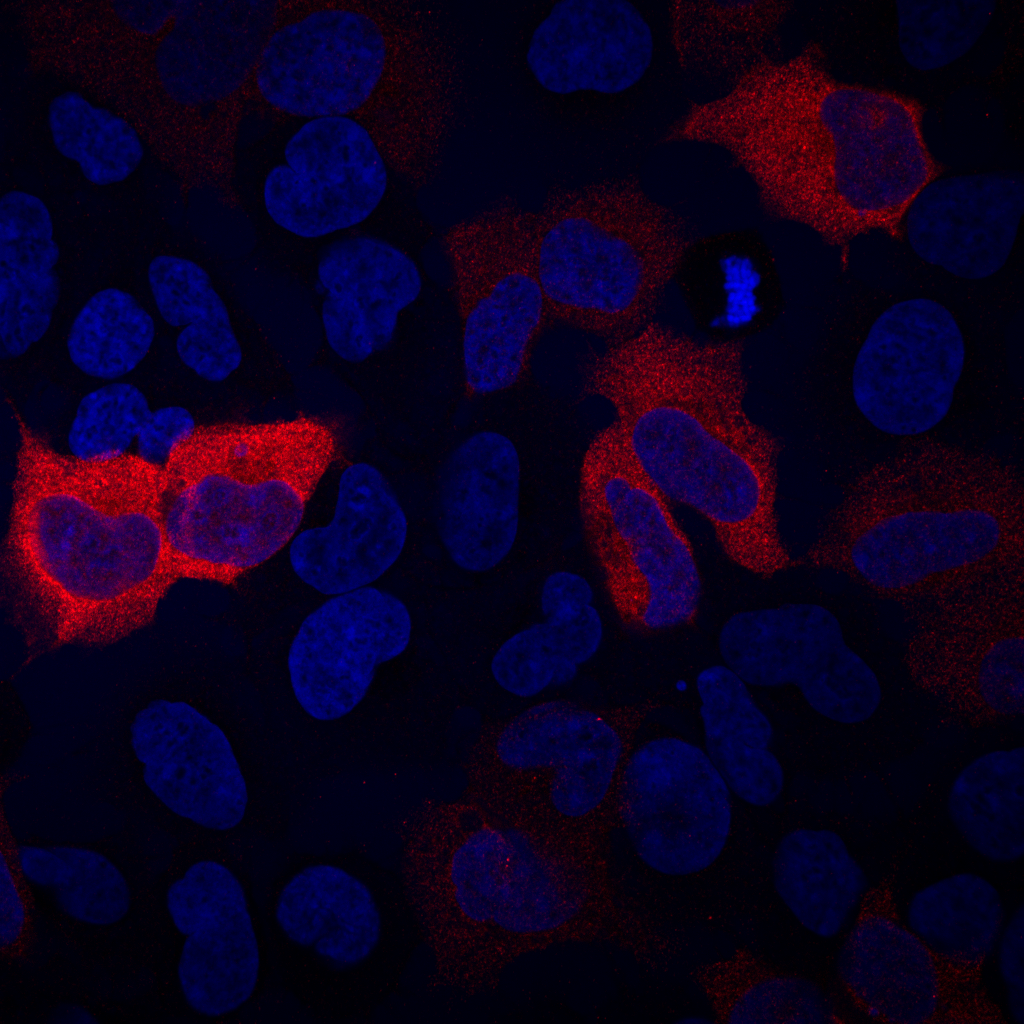

Supplement: Supplementary file 7 — Source data Fig. 5 [file 44319_2024_215_MOESM7_ESM.zip › Figure 5/5F/Images/WT_Fed_merge.tif]

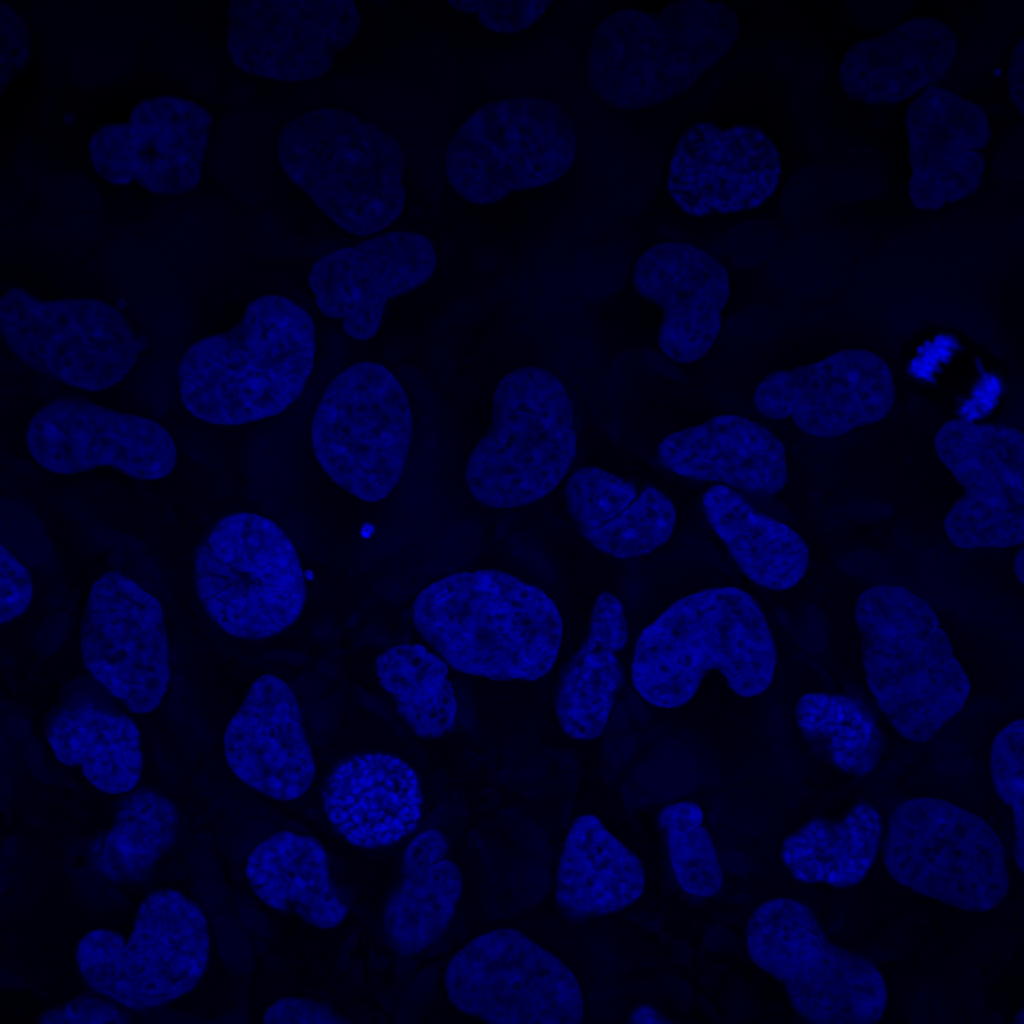

Supplement: Supplementary file 7 — Source data Fig. 5 [file 44319_2024_215_MOESM7_ESM.zip › Figure 5/5F/Images/S284A_Starved_hoechst.tif]

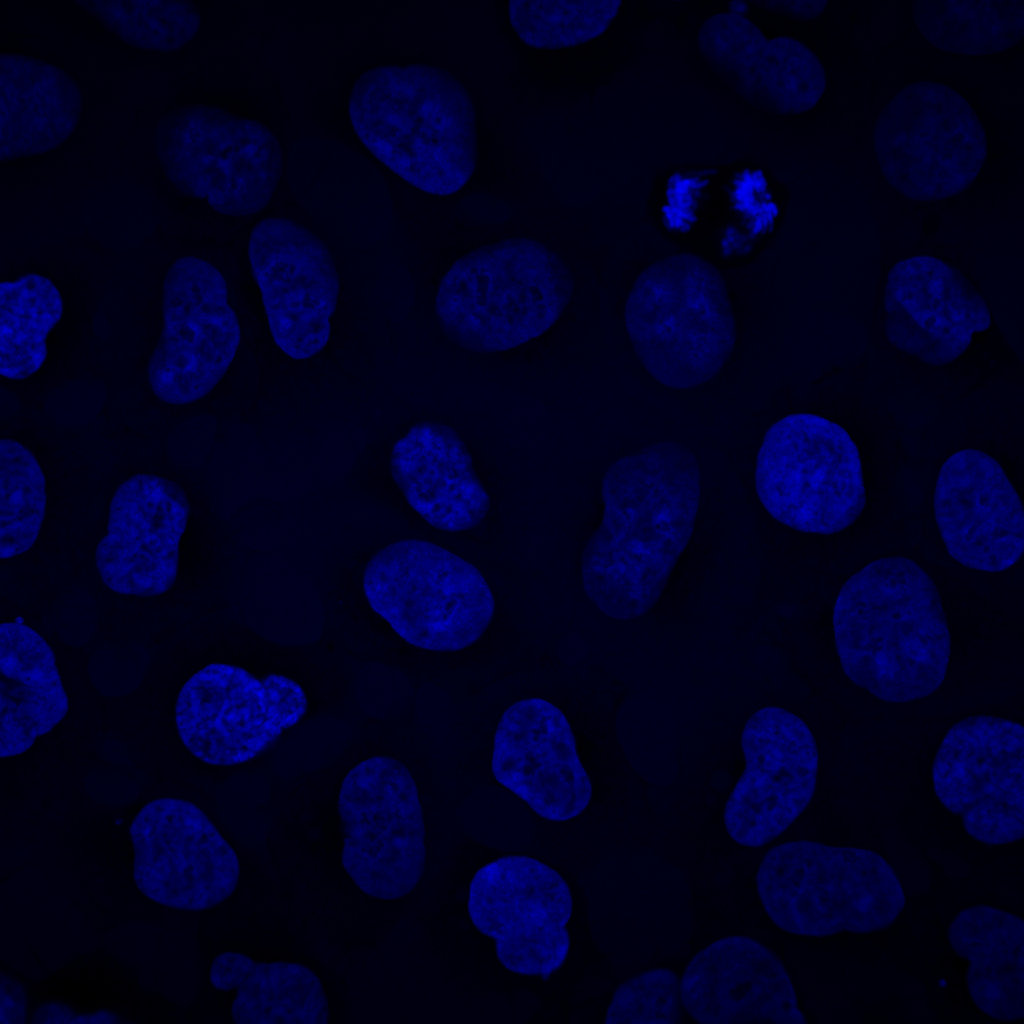

Supplement: Supplementary file 7 — Source data Fig. 5 [file 44319_2024_215_MOESM7_ESM.zip › Figure 5/5F/Images/S284A_Fed_hoechst.tif]

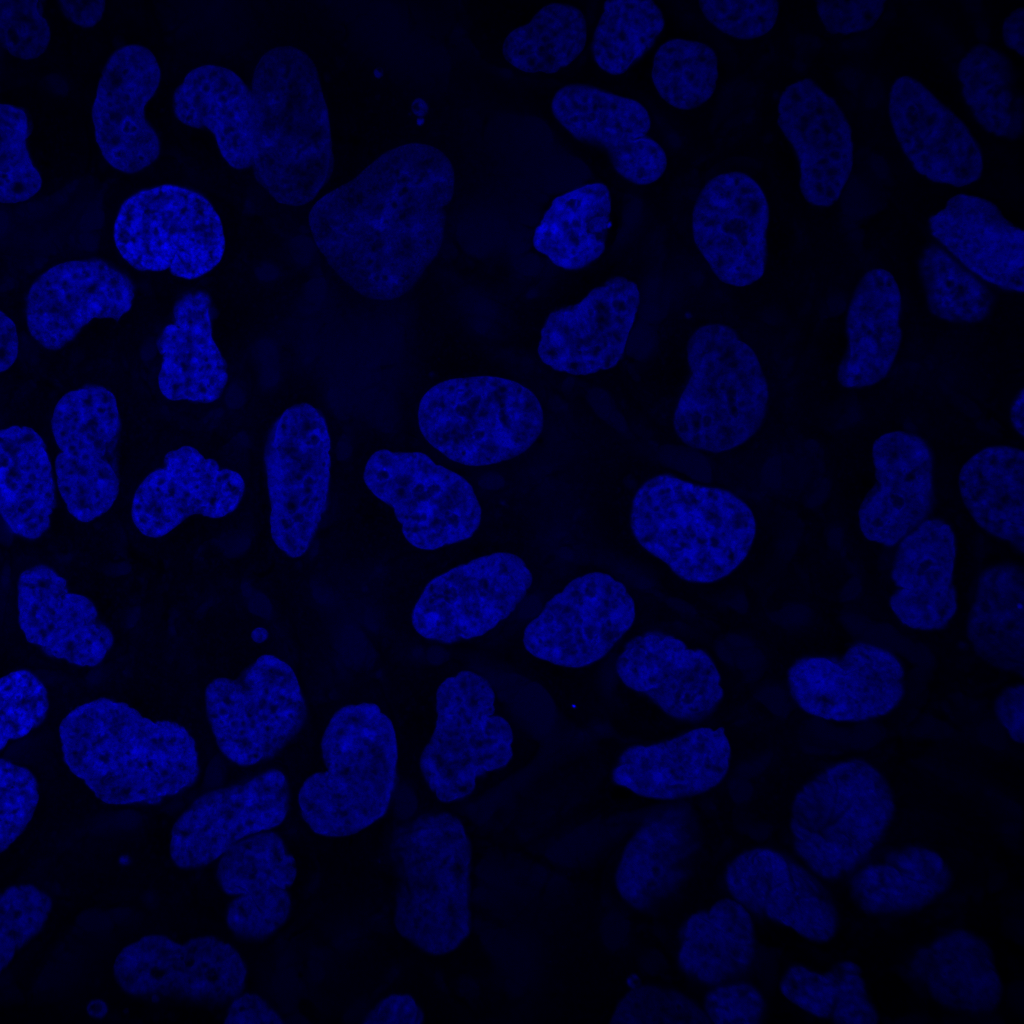

Supplement: Supplementary file 7 — Source data Fig. 5 [file 44319_2024_215_MOESM7_ESM.zip › Figure 5/5F/Images/S284D_StarvedBaf_hoechst.tif]

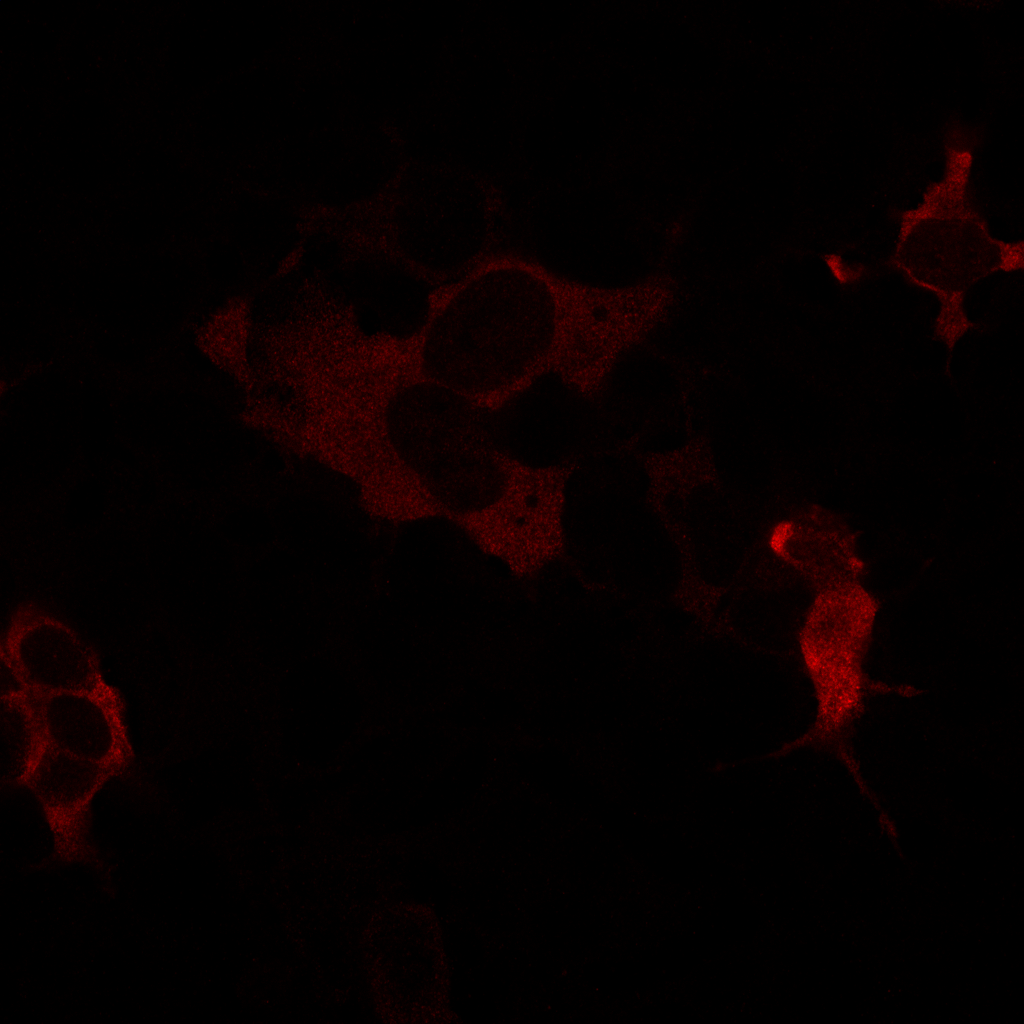

Supplement: Supplementary file 7 — Source data Fig. 5 [file 44319_2024_215_MOESM7_ESM.zip › Figure 5/5F/Images/S284D_Starved_HA.tif]

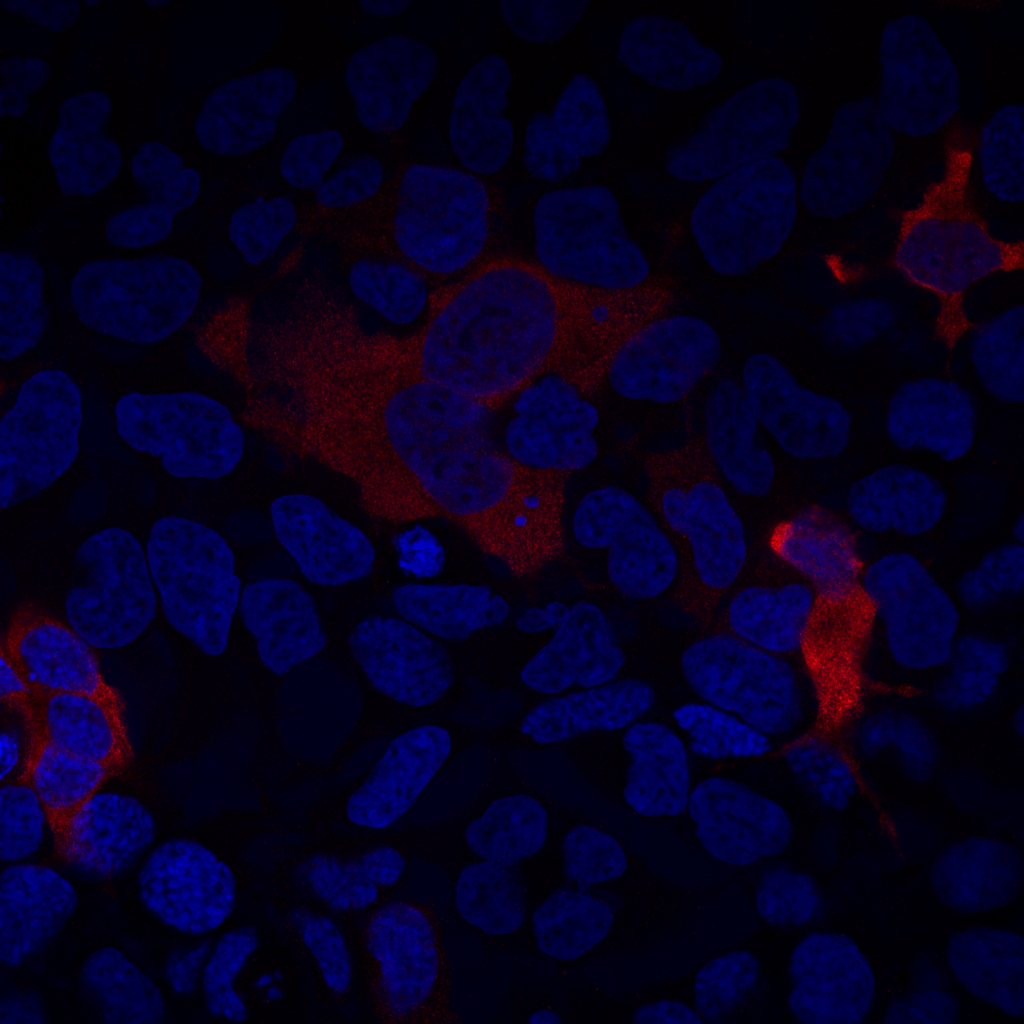

Supplement: Supplementary file 7 — Source data Fig. 5 [file 44319_2024_215_MOESM7_ESM.zip › Figure 5/5F/Images/S284D_Starved_merge.tif]

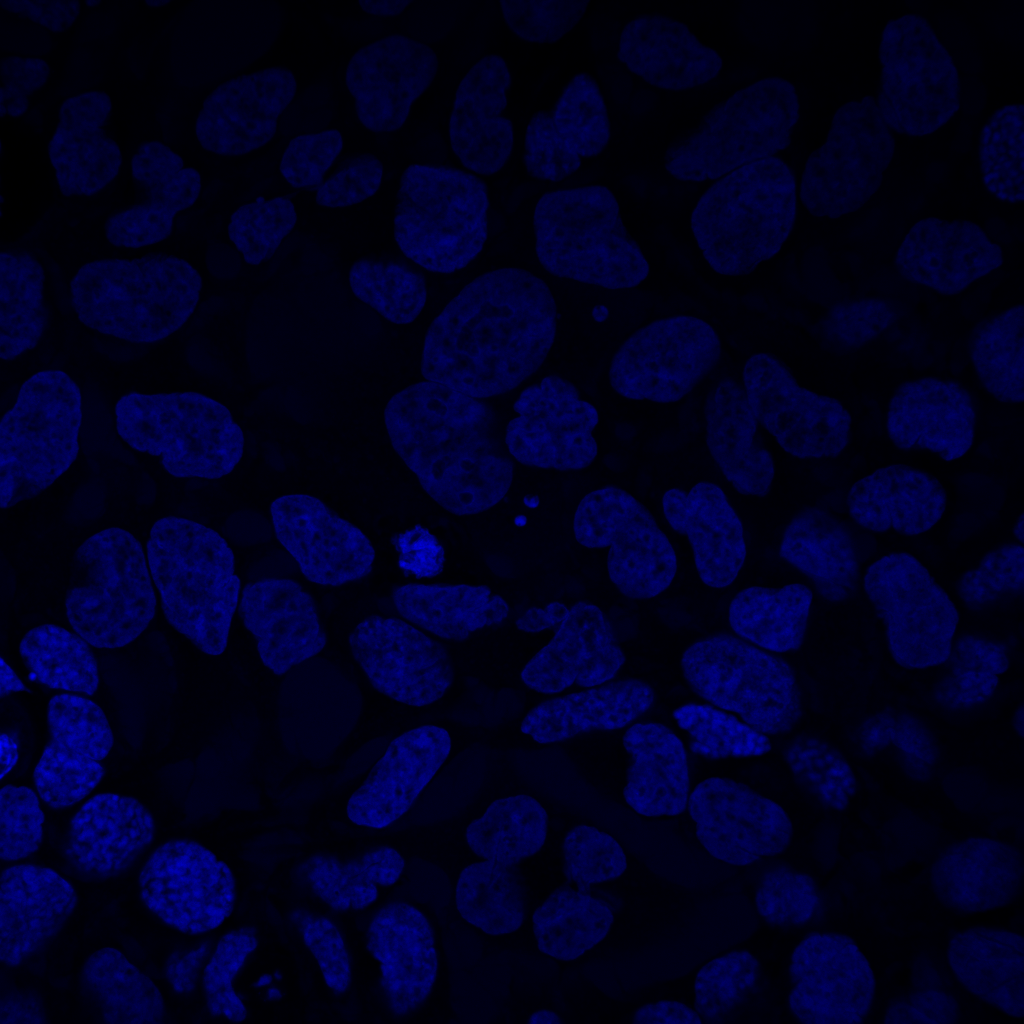

Supplement: Supplementary file 7 — Source data Fig. 5 [file 44319_2024_215_MOESM7_ESM.zip › Figure 5/5F/Images/S284D_Starved_hoechst.tif]

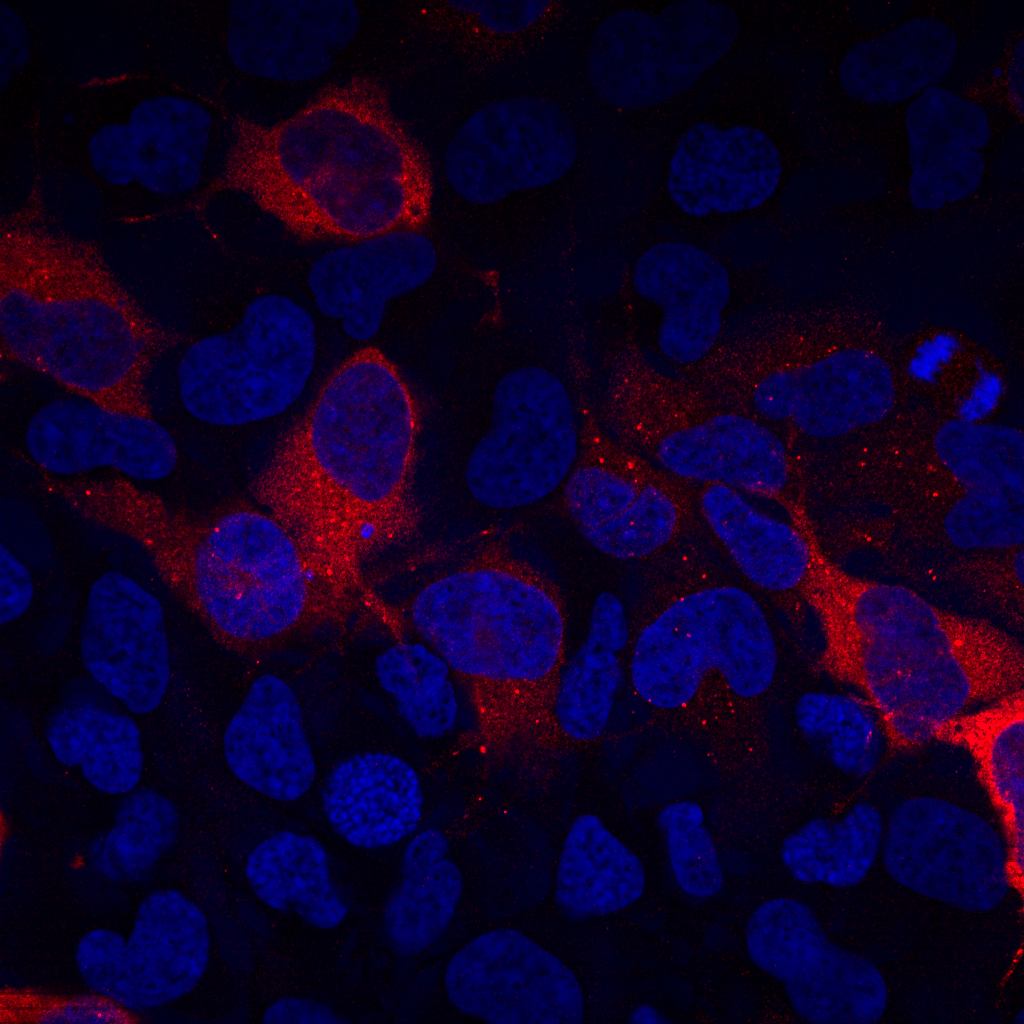

Supplement: Supplementary file 7 — Source data Fig. 5 [file 44319_2024_215_MOESM7_ESM.zip › Figure 5/5F/Images/S284A_Starved_merge.tif]

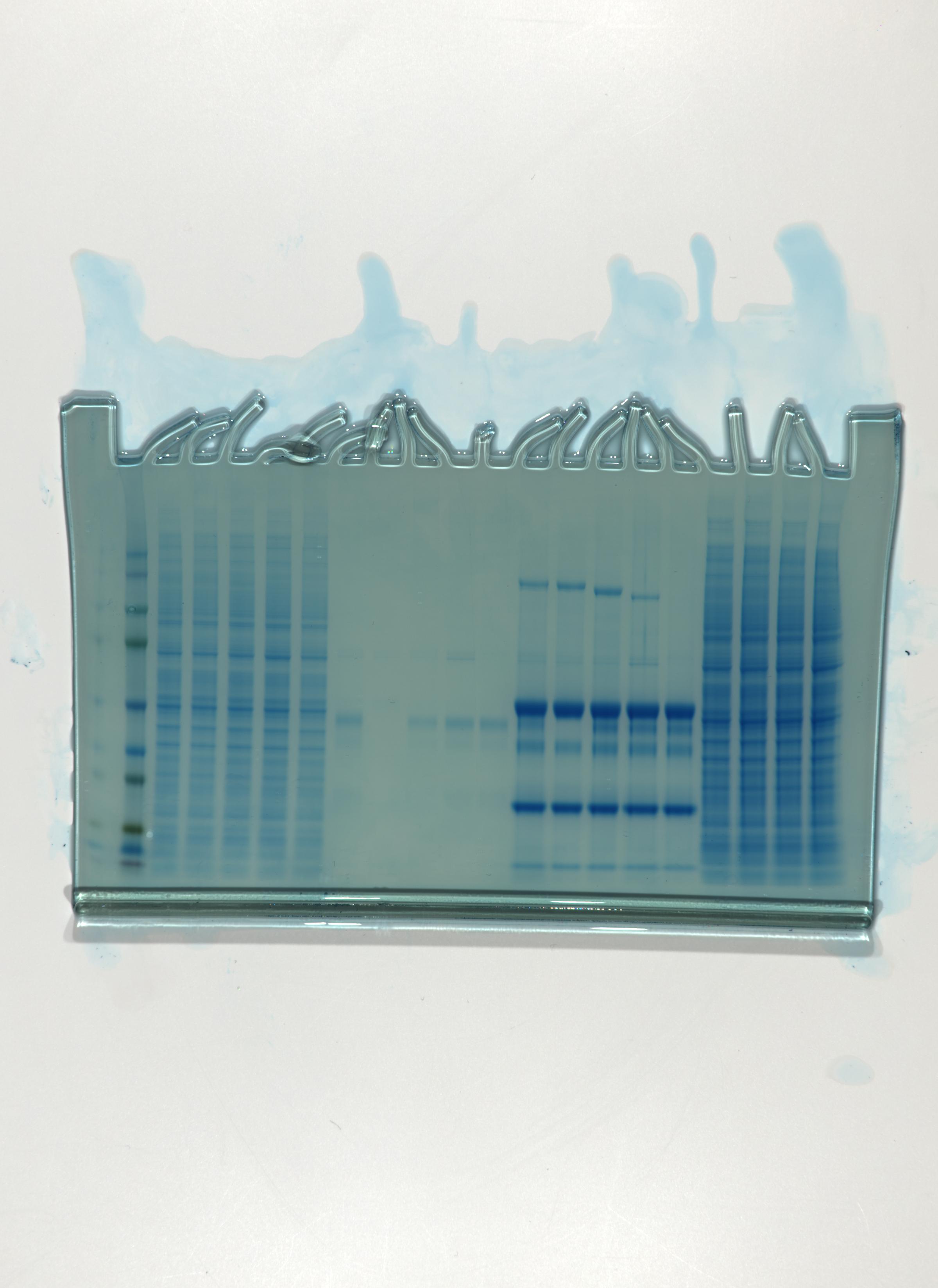

Supplement: Supplementary file 7 — Source data Fig. 5 [file 44319_2024_215_MOESM7_ESM.zip › Figure 5/5C/Image/Coomassie.jpg]

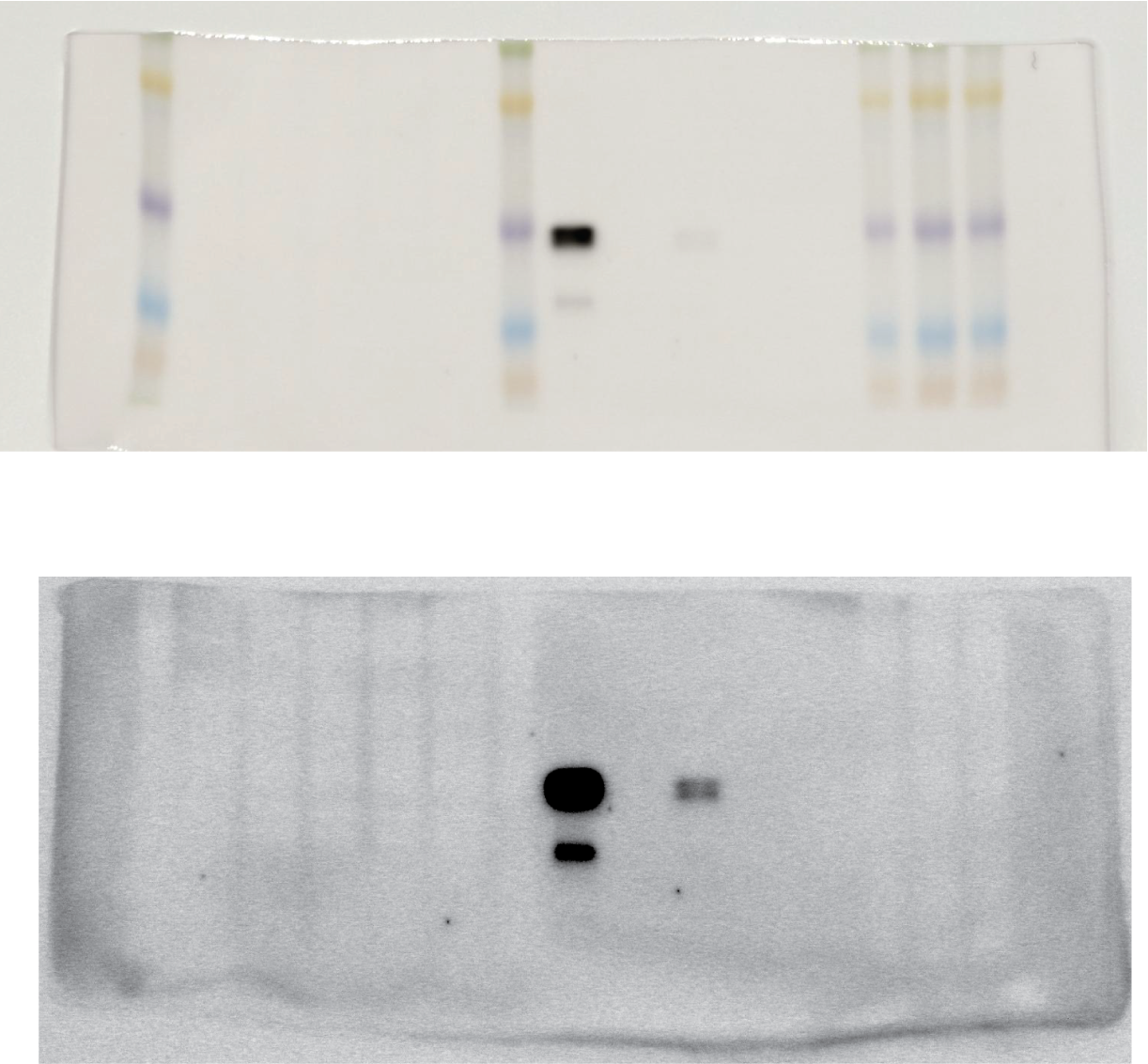

Supplement: Supplementary file 7 — Source data Fig. 5 [file 44319_2024_215_MOESM7_ESM.zip › Figure 5/5D/Images/western WIPI2 (pS284).tif]

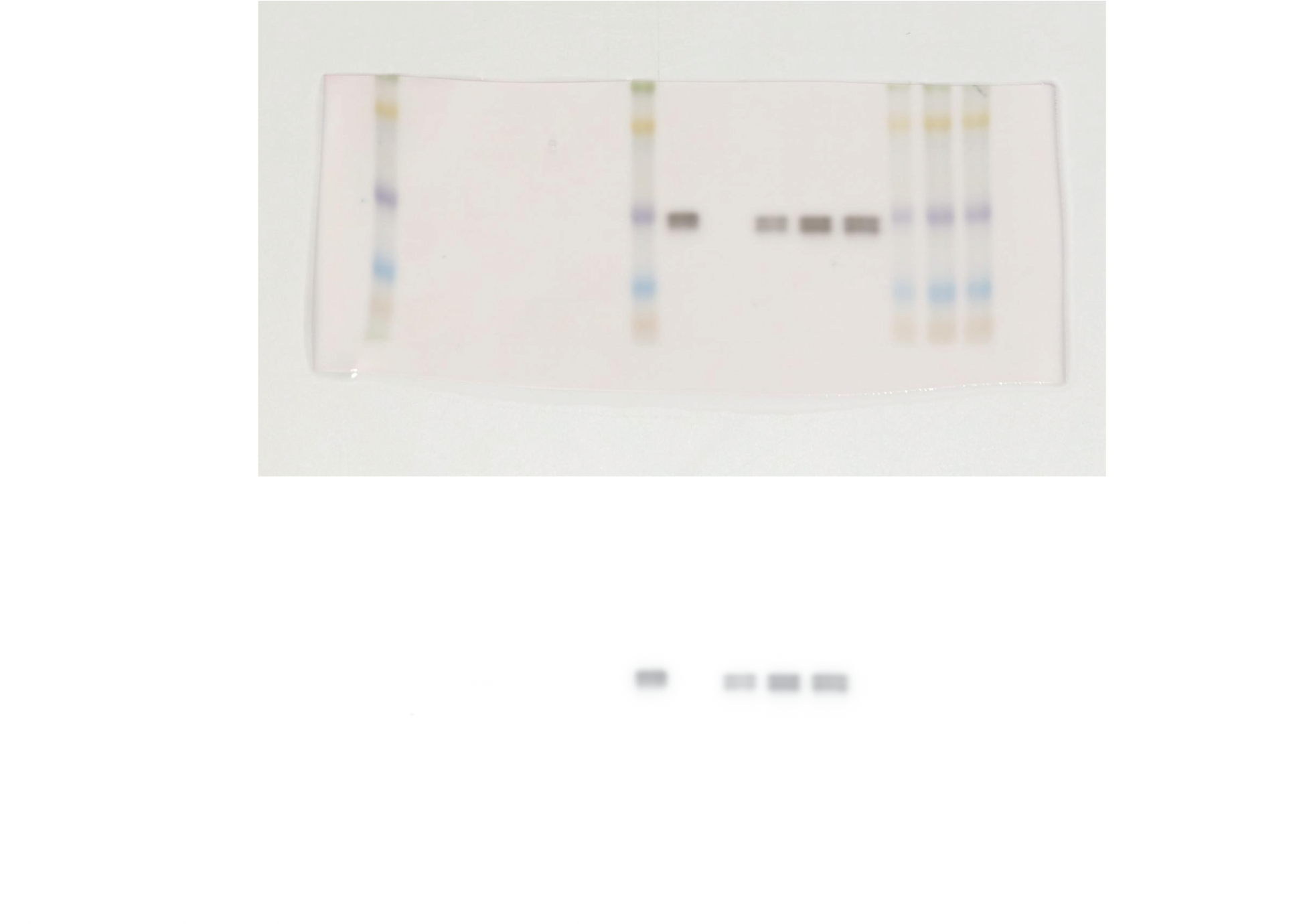

Supplement: Supplementary file 7 — Source data Fig. 5 [file 44319_2024_215_MOESM7_ESM.zip › Figure 5/5D/Images/western WIPI2 total.tif]

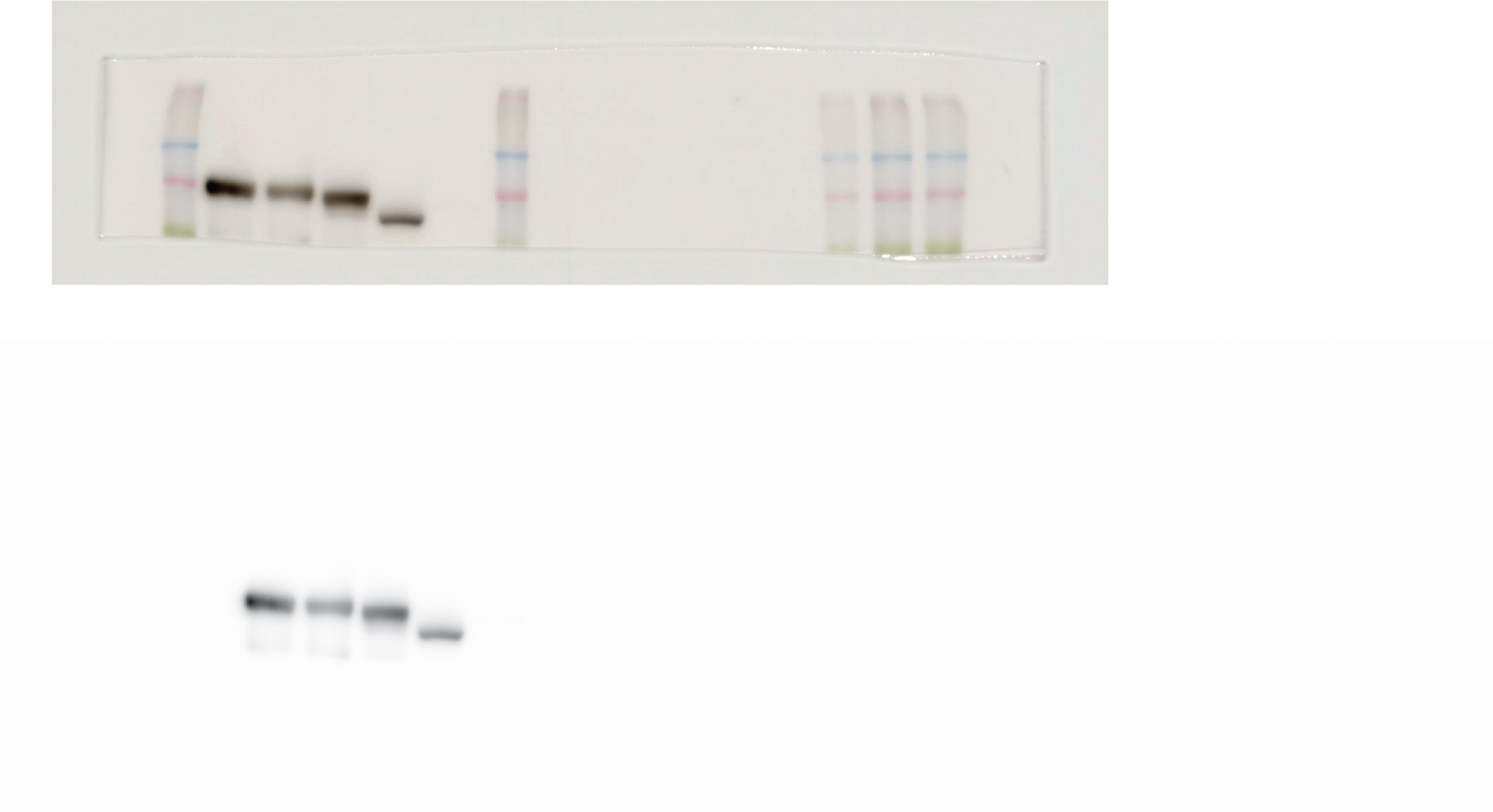

Supplement: Supplementary file 7 — Source data Fig. 5 [file 44319_2024_215_MOESM7_ESM.zip › Figure 5/5D/Images/western ULK1.tif]

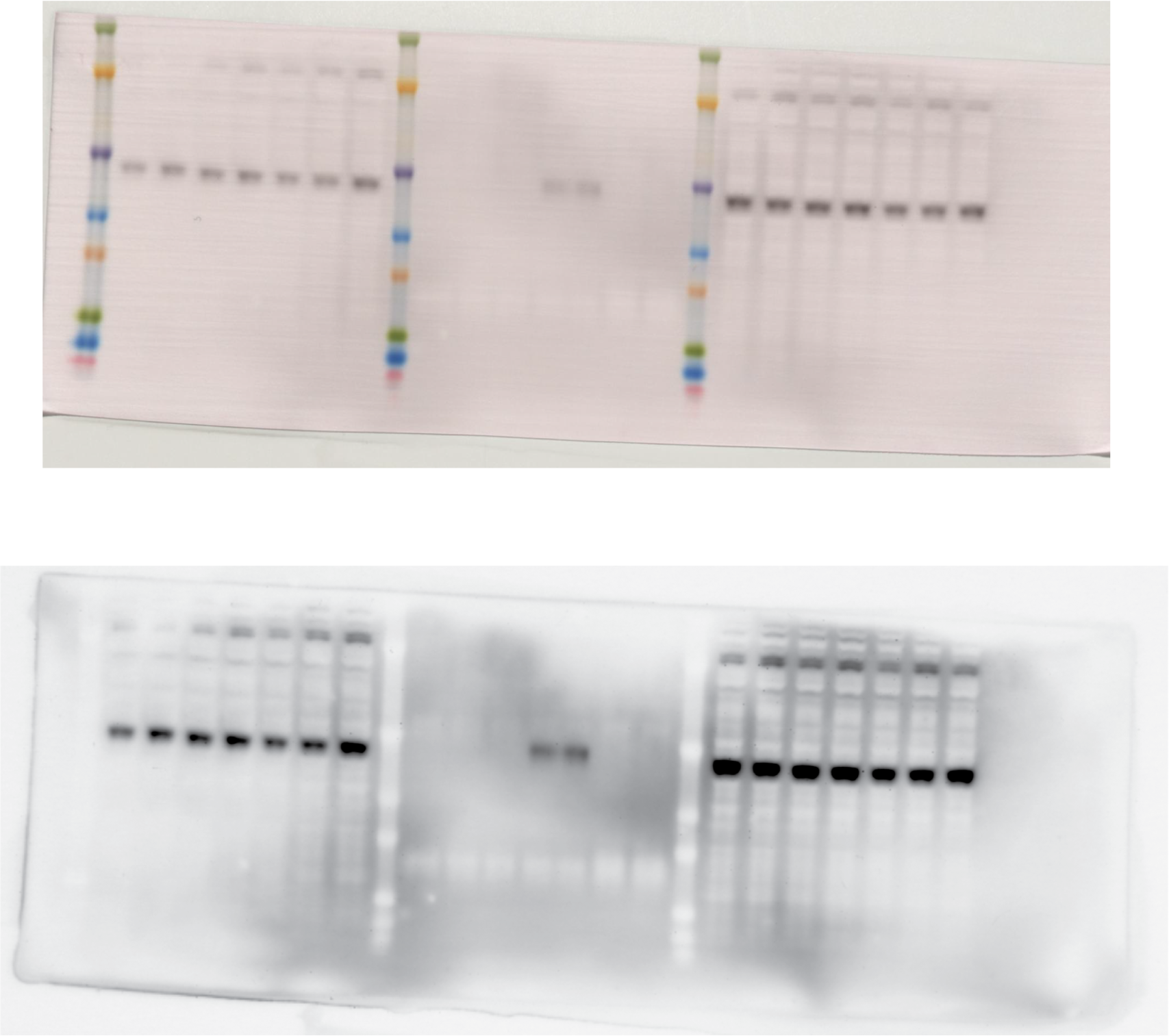

Supplement: Supplementary file 7 — Source data Fig. 5 [file 44319_2024_215_MOESM7_ESM.zip › Figure 5/5B/Images/western WIPI2 total.tif]

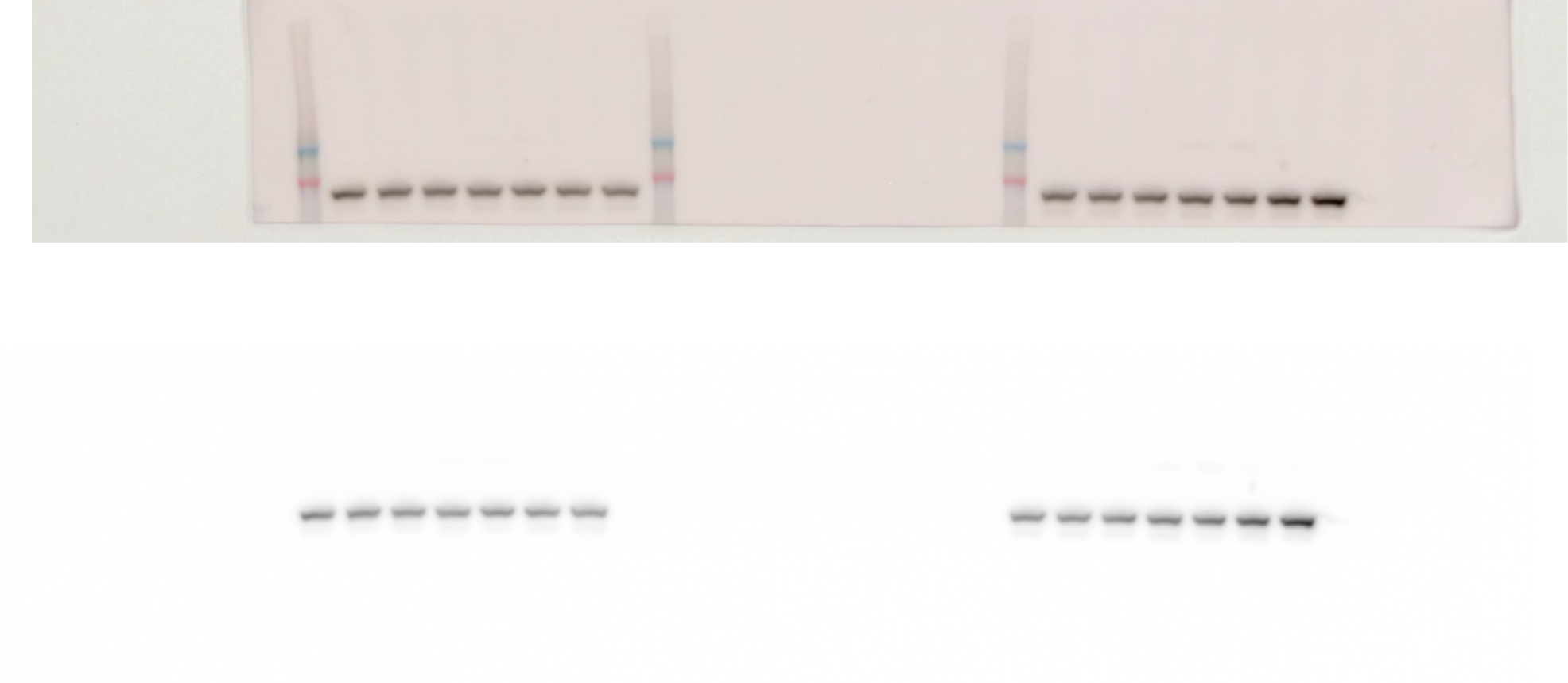

Supplement: Supplementary file 7 — Source data Fig. 5 [file 44319_2024_215_MOESM7_ESM.zip › Figure 5/5B/Images/western Vinculin.tif]
