# Supplementary material for: WIPI2b recruitment to phagophores and ATG16L1 binding are regulated by ULK1 phosphorylation
Source: EMBO Rep. 2024 Aug 16;25(9):8. doi: 10.1038/s44319-024-00215-5 (PMC11387628; doi:10.1038/s44319-024-00215-5)
Supplement: Supplementary file 8 — Expanded View Figures [file 44319_2024_215_MOESM8_ESM.pdf]

Expanded View Figures

A

Clone	Mutation	STOP codon	Mw of synthesised product
**G12C11**	68 nt insertion	exon 3	~7 kDa
	8 nt deletion	exon 5	~10 kDa
**G41C11**	97 nt insertion	exon 3	~7 kDa
	53 nt insertion	exon 3	~7 kDa

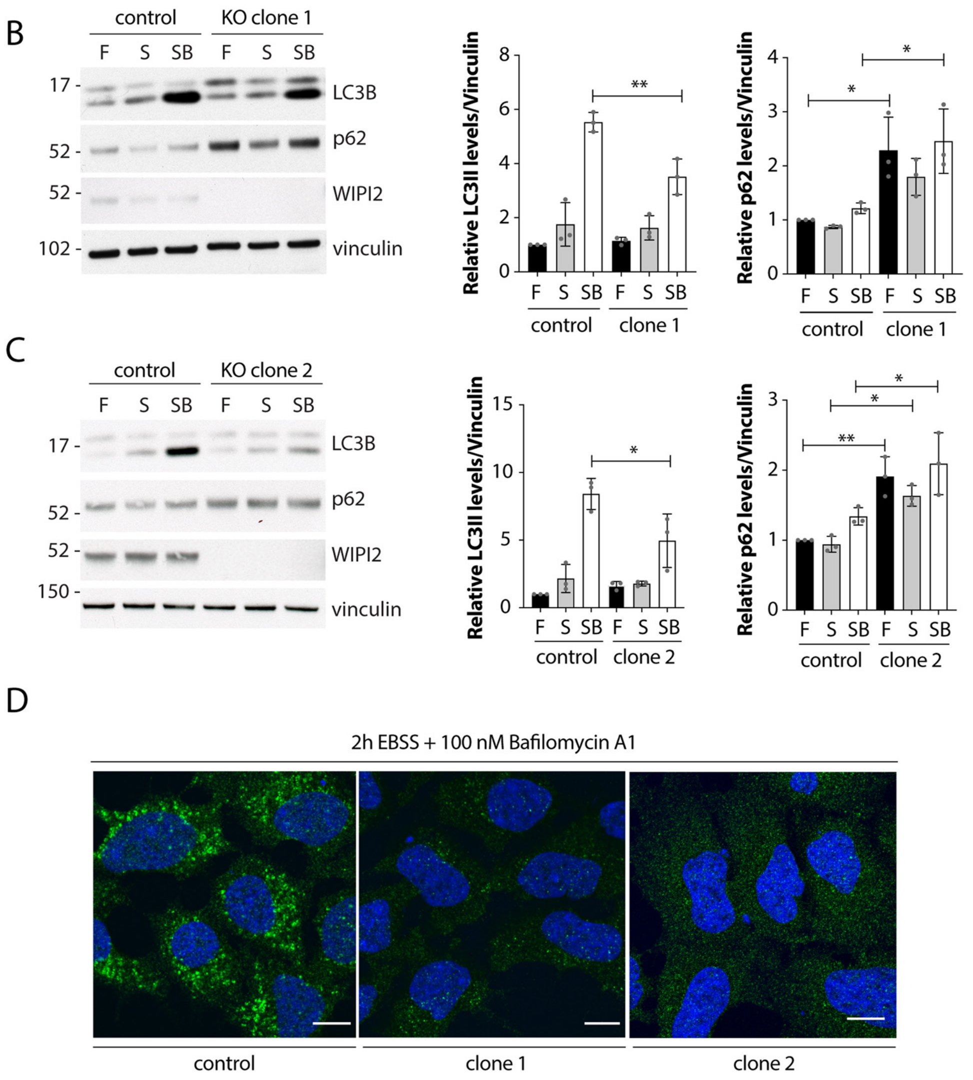

Figure EV1. Validation of WIPI2KO cells.

(**A**) WIPI2 CRISPR KO clones and gene mutations. (**B**) WIPI2 control and KO clone G12C11 (clone 1) were incubated 2 h in full medium (F), EBSS (S) or EBSS supplemented with Bafilomycin A1 (SB) and analysed by western blot. LC3-II levels from 3 independent experiments were quantified using one-way ANOVA with Tukey's post test. $^{**}P = 0.0029$. p62 levels from 3 independent experiments were quantified using one-way ANOVA with Tukey's post test. F vs F $^{*}P = 0.0129$. (**C**) WIPI2 control and KO clone G41C11 (clone 1) were treated as in (**B**). LC3-II levels from 3 independent experiments were quantified using one-way ANOVA with Tukey's post test. $^{*}P = 0.015$. p62 levels from 3 independent experiments were quantified using one-way ANOVA with Tukey's post test. F vs F $^{**}P = 0.0045$, S vs S $^{*}P = 0.0312$, SB vs SB $^{*}P = 0.0183$. (**D**) WIPI2 control, G12C11 (clone 1) and G41C11 (clone 2) cell lines were incubated in EBSS with Bafilomycin A1 for 2 h and analysed by confocal microscopy after staining with LC3 antibody. Scale bar = 10 µm.

© The Author(s)

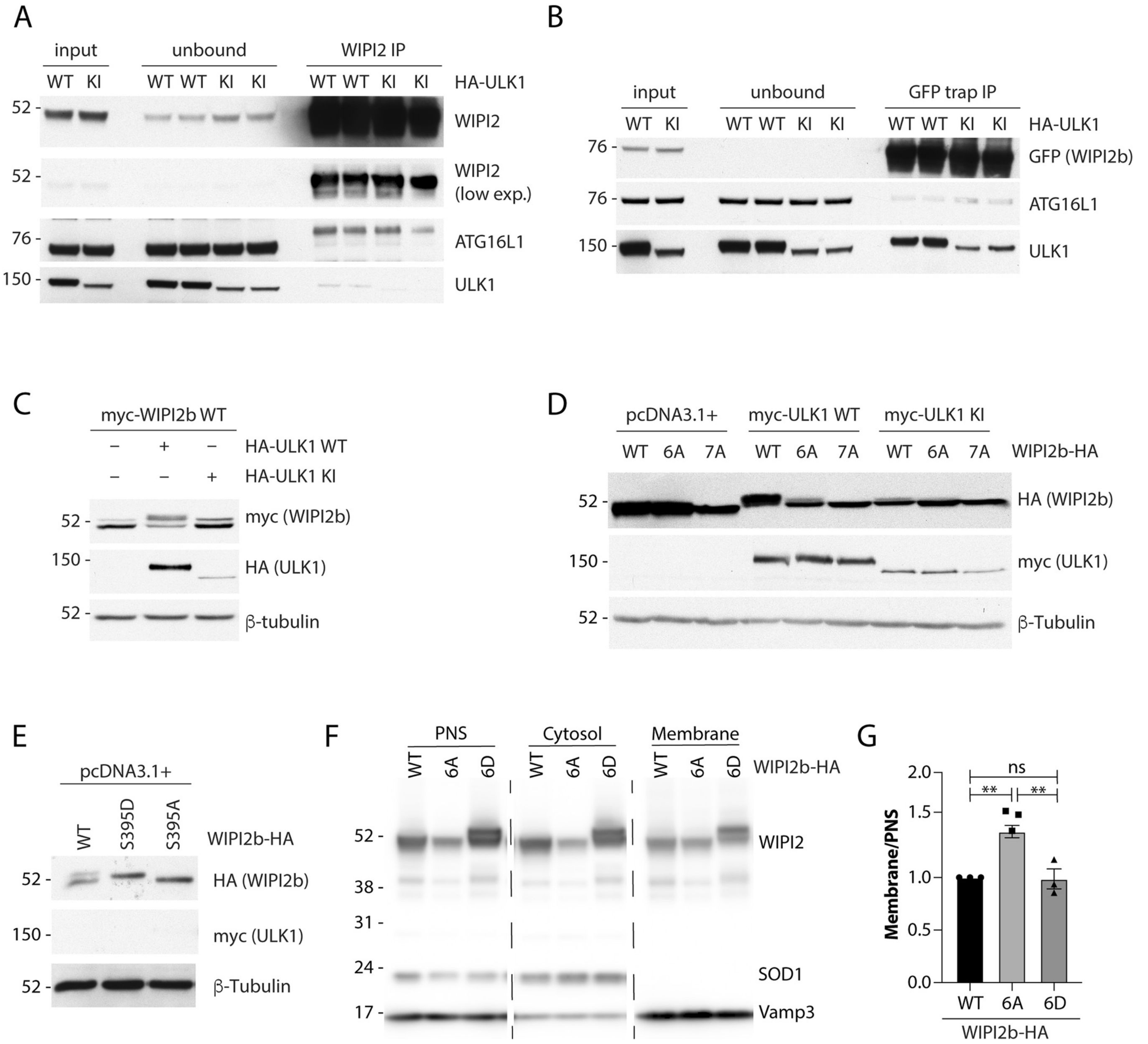

Figure EV2. WIPI2b is phosphorylated by ULK1 at 6 sites.

(**A**, **B**) Samples used for mass spectrometry for detection of phosphorylation sites on endogenous WIPI2 (**A**) and transiently expressed GFP-WIPI2b WT (**B**) with transient expression of HA-ULK1 WT or KI before and after immunoprecipitation were analysed by SDS-PAGE and western blot. (**C**) HEK293A cells were transiently transfected with empty vector, HA-ULK1 WT or HA-ULK1 KI and myc-WIPI2b WT and starved for 2 h in EBSS before being analysed by western blot. (**D**) HEK293A cells transiently co-expressing WIPI2b-HA WT, WIPI2b-HA 6A or WIPI2b-HA 7A and empty vector, myc-ULK1 WT or myc-ULK1 KI were treated for 2 h in EBSS and analysed by western blot with indicated antibodies. Representative experiment from $n = 3$. (**E**) HEK293A cells co-expressing WIPI2b-HA WT, WIPI2b-HA S395D or WIPI2b-HA S395A and incubated for 2 h in EBSS, lysed and analysed by western blot. Representative experiment from $n = 3$. (**F**) Crude cellular fractionation was performed on WIPI2 KO cells transiently expressing WIPI2b-HA WT, 6A or 6D. Membrane fraction was solubilised to a 1/5th of the volume of the cytosol. Equal volumes of each fraction were analysed by western blot. SOD1 was used as a marker for the cytosolic fraction, and Vamp3 as a marker for the membrane fraction. (**G**) Quantification of WIPI2b-HA membrane levels in (**F**). Membrane fraction over post-nuclear supernatant input (PNS). Mean with SEM using one-way ANOVA with Tukey's post test from 3 independent experiments. WT vs 6A $^{**}P = 0.0069$ 6 A vs 6D $^{**}P = 0.0060$.

 © The Author(s)

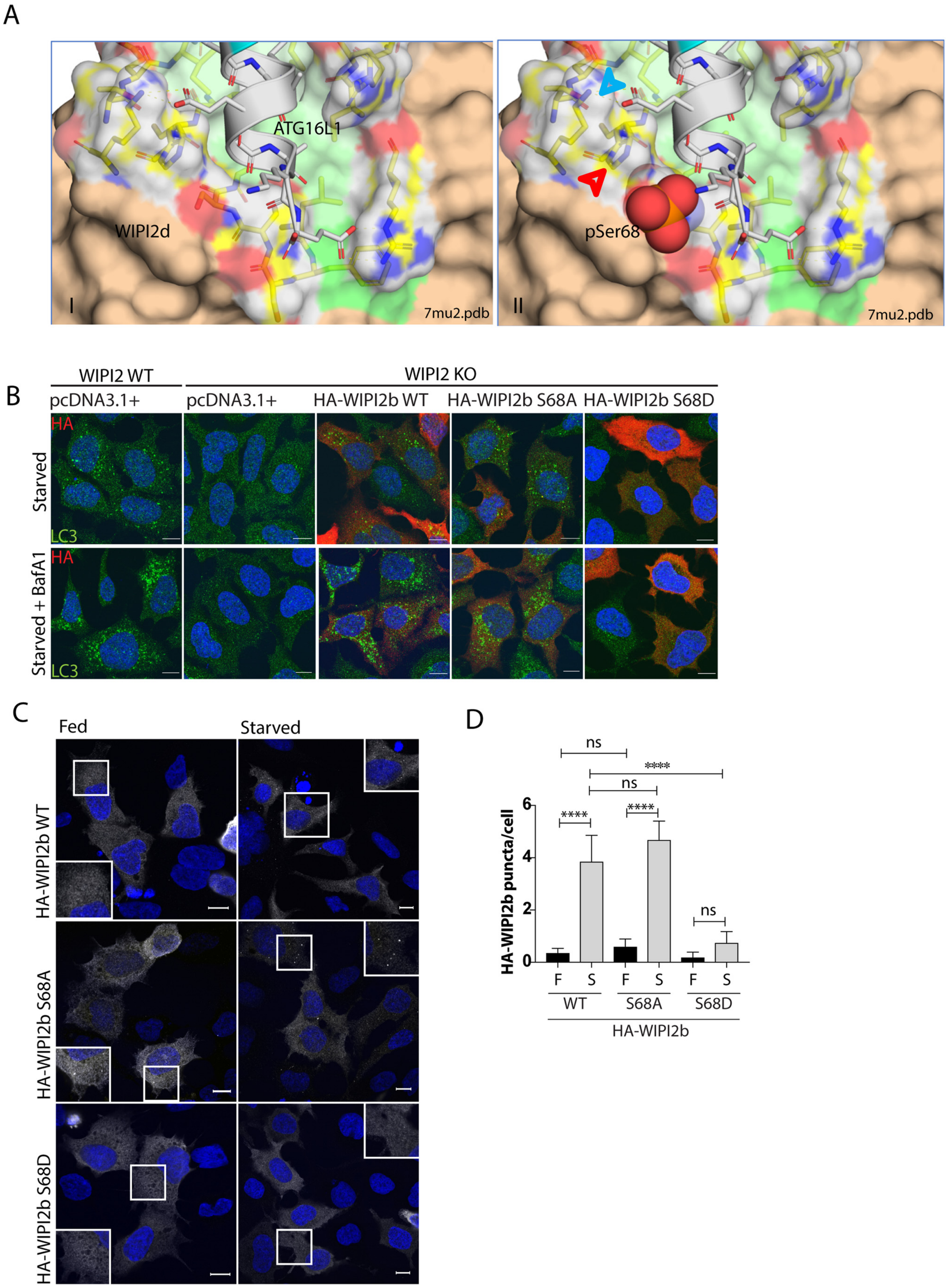
A
ATG16L1
WIPI2d
I
7mu2.pdb
pSer68
II
7mu2.pdb
B
WIPI2 WT
WIPI2 KO
pcDNA3.1+
pcDNA3.1+
HA-WIPI2b WT
HA-WIPI2b S68A
HA-WIPI2b S68D
Starved
Starved + BafA1
HA
LC3
HA
LC3
C
Fed
Starved
HA-WIPI2b WT
HA-WIPI2b S68A
HA-WIPI2b S68D
D
HA-WIPI2b puncta/cell
ns
****
ns
****
****
ns
0
2
4
6
F
S
F
S
F
S
WT
S68A
S68D
HA-WIPI2b

Figure EV3. Phosphorylation at WIPI2b S68 reduces WIPI2 and LC3 puncta formation.

(**A**) (I) Model of human WIPI2d bound to ATG16L1 α-helix (Strong et al, 2021). PDB 7mu2.pdb. Atoms of contact sites coloured as follows – oxygen, red; nitrogen, blue; carbon WIPI2d yellow, ATG16L1 grey; hydrophobic residues shaded green according to hydrophobicity scale (White and Wimley, 1998). (II) Phosphoserine 68 is represented as a red molecule in the bottom image. Residues H85 and K88 are marked by a red and blue arrowhead respectively. (**B**) WIPI2 control cells expressing empty vector and WIPI2 KO cells expressing empty vector, HA-WIPI2b WT, HA-WIPI2b S68A and HA-WIPI2b S68D were incubated 2 h in EBSS with or without Bafilomycin A1. Cells were analysed by confocal microscopy after immunostaining with LC3B and HA antibodies. Scale bar = 10 µm. (**C**) WIPI2 KO cells were transiently transfected with HA-WIPI2b WT, HA-WIPI2b S68A and HA-WIPI2b S68D, and starved for two h or left untreated. Cells were analysed by immunostaining with anti-HA antibody. Scale bar = 10 µm. (**D**) Quantification of (**C**) HA-positive puncta. SEM from at least 120 cells per condition from two experiments. One-way ANOVA with Tukey's post test $^{****}P < 0.0001$.

© The Author(s)

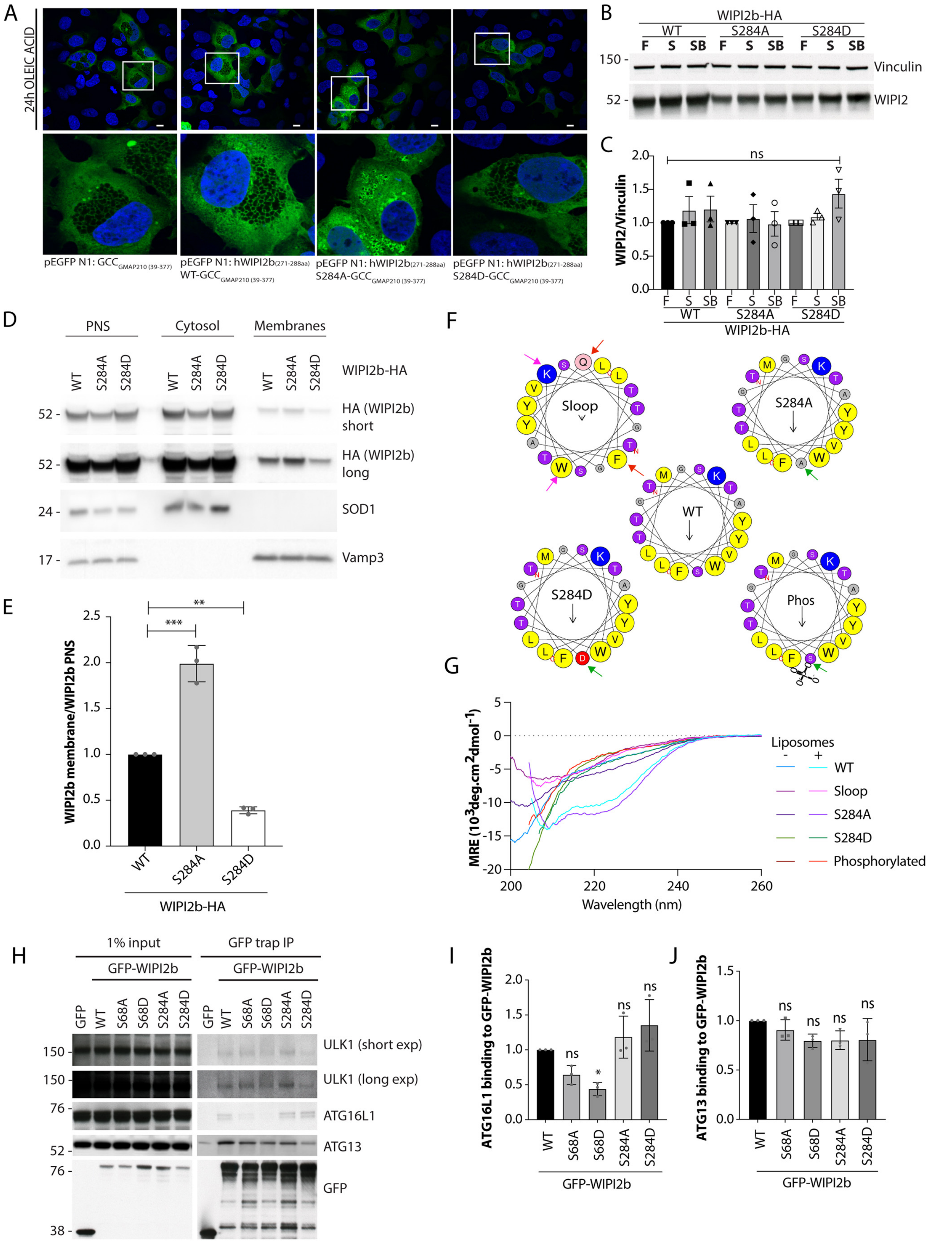
A
24h OLEIC ACID
pEGFP N1: GCC GMAP210 (39-377)
pEGFP N1: hWIPI2b(271-288aa) WT-GCC GMAP210 (39-377)
pEGFP N1: hWIPI2b(271-288aa) S284A-GCC GMAP210 (39-377)
pEGFP N1: hWIPI2b(271-288aa) S284D-GCC GMAP210 (39-377)
B
WIPI2b-HA
WT S284A S284D
F S SB F S SB F S SB
150 -
52 -
Vinculin
WIPI2
C
WIPI2/Vinculin
ns
F S SB F S SB F S SB
WT S284A S284D
WIPI2b-HA
D
PNS Cytosol Membranes
WT S284A S284D WT S284A S284D WT S284A S284D
WIPI2b-HA
HA (WIPI2b) short
HA (WIPI2b) long
SOD1
Vamp3
E
WIPI2b membrane/WIPI2b PNS
***
**
WT S284A S284D
WIPI2b-HA
F
Sloop
S284A
WT
S284D
Phos
G
MRE (10³deg.cm²dmol⁻¹)
Wavelength (nm)
Liposomes
- +
WT
Sloop
S284A
S284D
Phosphorylated
H
1% input GFP trap IP
GFP-WIPI2b GFP-WIPI2b
GFP WT S68A S68D S284A S284D GFP WT S68A S68D S284A S284D
ULK1 (short exp)
ULK1 (long exp)
ATG16L1
ATG13
GFP
I
ATG16L1 binding to GFP-WIPI2b
ns * ns ns
WT S68A S68D S284A S284D
GFP-WIPI2b
J
ATG13 binding to GFP-WIPI2b
ns ns ns ns
WT S68A S68D S284A S284D
GFP-WIPI2b

Figure EV4. Phosphorylation at WIPI2b S284 affects amphipathic helix formation.

(**A**) WIPI2 CRISPR KO cells were transiently transfected cells with pEGFP N1:GCC-GMAP210 (39-377) (negative control), pEGFP N1:WIPI2b WT-GMAP210 (39-377), pEGFP N1:WIPI2b S284A-GMAP210 (39-377), or pEGFP N1:WIPI2b S284D-GMAP210 (30–377) were treated with oleic acid for 24 h and fixed in 4% PFA. Scale bar = 10 µm. (**B**) WIPI2 KO HEK293A cells transiently expressing WIPI2b-HA WT, WIPI2b-HA S284A or WIPI2b-HA S284D were left untreated (Fed, F) incubated in EBSS for 2 h with (SB) or without Bafilomycin A1 (S). (**C**) Quantification of WIPI2 levels in (**B**) normalised to vinculin. SEM from $n = 3$ biological replicates. Statistical analysis was performed by one-way ANOVA with Tukey's post test, ns, $P > 0.05$. (**D**) Crude cell fractionation was performed in HEK293A cells transiently expressing WIPI2b-HA WT, WIPI2b-HA S284A and WIPI2b-HA S284D. Equal volumes of post-nuclear supernatant (PNS), cytosol and membranes were analysed by western blot. Representative experiment of $n = 3$. (**E**) Quantification of WIPI2b-HA membrane levels in (**D**). SEM from $n = 3$ biological replicates. Statistical analysis was performed by one-way ANOVA with Tukey's post test. $^{**}P = 0.0017$, $^{***}P = 0.001$. (**F**) Helical wheel representations of WT and amphipathic helix peptides used for CD spectra (made in Heliquest (Gautier et al, 2008)). Coloured arrows indicate where mutations/modifications have been made. Residues are colour coded as follows; blue, basic; red, acidic; yellow, hydrophobic; purple, serine and threonine; pink, asparagine and glutamine; grey, alanine and glycine. (**G**) Far UV CD spectra of individual peptides in the absence and presence of 3 mM 100 nm liposomes. Raw CD data is converted to mean residue ellipticity and the percentage helicity is calculated from the value at 222 nm. $N = 3$. (**H**) HEK293A cells transiently expressing GFP alone, GFP-WIPI2b WT, GFP-WIPI2b S68A, GFP-WIPI2b S68D, GFP-WIPI2b S284A or GFP-WIPI2b S284D were lysed and subjected to GFP-TRAP co-immunoprecipitation. Protein complexes were resolved by SDS-PAGE and analysed by western blot. (**I**) ATG16L1 binding to GFP-WIPI2b quantified using one-way ANOVA with Tukey's post test. SEM from $n = 3$ biological replicates. $^{*}P = 0.0381$. (**J**) ATG13 binding to GFP-WIPI2b quantified using one-way ANOVA with Tukey's post test. SEM from $n = 3$ biological replicates. ns, $P > 0.05$.

 © The Author(s)

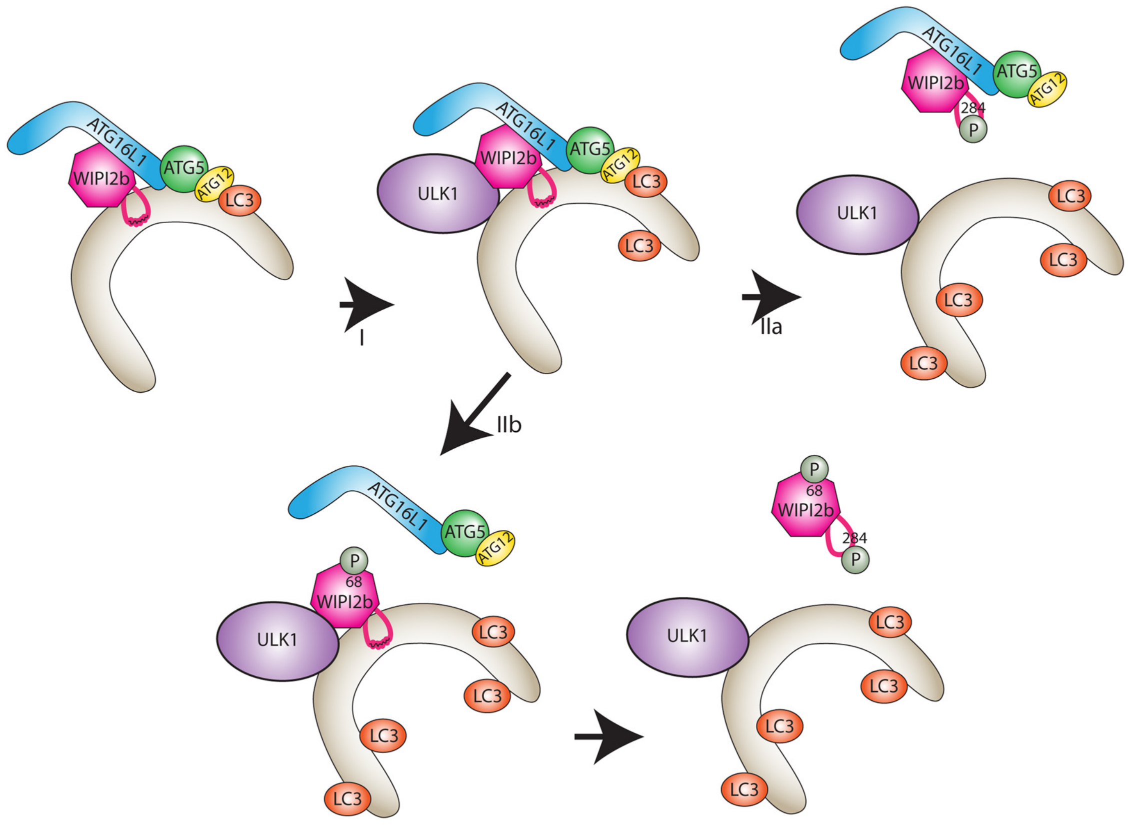

Figure EV5. Working model.

Upon autophagy initiation WIPI2b is recruited to PI3P-positive membranes, which in turn recruits ATG16L1 for LC3 lipidation. (I) ULK1 binds WIPI2b and (IIa) phosphorylates it at S284 and (IIb) S68. Phosphorylation at S284 reduces WIPI2bs membrane association, likely by disrupting amphipathic helix formation.
